# Expanding the repertoire of imine reductases by mining divergent biosynthetic pathways for promiscuous reactivity

**DOI:** 10.1016/j.checat.2024.101160

**Published:** 2024-12-19

**Authors:** Godwin A. Aleku, Florian Hollfelder

**Affiliations:** 1 Department of Biochemistry University of Cambridge, 80 Tennis Court Road, CB2 1GA Cambridge, UK; 2 Institute of Pharmaceutical Science, Franklin-Wilkins Building, King’s College London, 150 Stamford Street, SE1 9NH London, UK

**Keywords:** imine reductases, enzyme discovery, biocatalysis, imine reduction, reductive amination, biosynthetic enzymes, functional genomics, enzyme promiscuity

## Abstract

Imine reductases (IREDs) are invaluable catalysts for enantioselective imine reduction and reductive amination of carbonyl compounds. Their synthetic versatility is, however, limited by their substrate scope, and new IREDs are needed. Current IREDs are closely related to the initially characterized enzymes, as their discovery has been driven by sequence homology searches. Here, we demonstrate a *functional* genomics approach based on biosynthetic promiscuity, guided by the identification of C=N reducing enzymes acting on large, complex substrates in biosynthetic pathways. These substrate-promiscuous biocatalysts share low homology to existing IREDs and fall into distinct functional enzyme families, yet they catalyze the hydrogenation of non-native imines as well as the reductive amination of simple ketones. Venturing further into sequence space without the constraints of close homology, but instead guided by functional promiscuity, has thus led us to distinct, previously unrecognized and unexplored areas of sequence space for mining IREDs for synthesis.

## Highlights

• A functional genomics approach identifies new imine reductases (IREDs) for biocatalysis
• 44 enzymes from 6 non-homologous C=N bond reducing biosynthetic enzyme families screened
• 46% of these reduced synthetic imines, and some catalyze reductive carbonyl amination
• Prospecting unexplored sequence space is a route to imine reductases for synthesis

## The bigger picture

Making enzymatic synthesis of active pharmaceutical ingredients (APIs) sustainable and attractive for industrial applications requires a repertoire of enzymes with activities and selectivities that surpass those of chemical synthesis methods. Reductive amination (RA) is one of the most frequently employed methods to synthesize amine-based APIs, so the use of imine reductases (IREDs) for this transformation is extremely attractive: catalytic reactions are performed with high efficiency and high stereoselectivity under mild conditions. Secondary and tertiary amines that are not accessible by other enzymatic methods can be synthesized in this way. However, the versatility of IRED-catalyzed reductive amination is limited by their substrate scope, so new IREDs to bridge gaps in specificity will increase the broad application of IREDs. Here, we explore a functional genomic approach to retrieve and characterize enzymes catalyzing unrelated physiological imine reduction reactions across several metabolic routes, revealing their promiscuity for the reductive amination of synthetic substrates. Our work has uncovered six useful enzyme families for imine reduction in previously unannotated sequence space, where their functional assignment creates “bridgeheads” for further searches for IREDs.

## Graphical abstract

**Figure:**
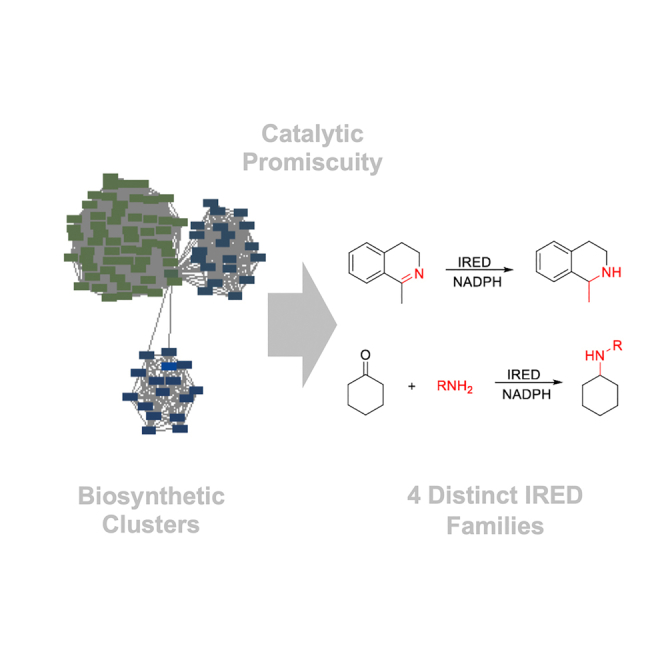

## Teaser

To identify useful biocatalytic enzymes in the vastness of sequence space, biosynthetic clusters with potential promiscuous activities for prospecting imine reductases (IREDs) like enzymes were defined. This approach guided the screening of 44 biosynthetic enzymes from six distinct non-homologous enzyme families, of which 46% showed activity and useful substrate scope for potential synthetic applications. These functional annotations based on experimental observations provide useful bridgeheads in previously unknown sequence space, provide practical guidelines for where to look for this type of activity, and increase confidence in further sequence-based annotations that are now more firmly anchored to actual observations.

## Introduction

Imine reductases (IREDs) have emerged as important members of the catalyst toolkit for the synthesis of chiral amine building blocks. IREDs (including the closely related subclass of reductive aminases [RedAms]) catalyze the NAD(P)H-dependent enantioselective reduction of imines and reductive amination of carbonyl compounds, allowing access to primary, secondary, and tertiary chiral amines.^1^^,^^2^ Compared with other conventional methods (e.g., chemo-catalytic and transaminase-based routes), IRED-catalyzed reductive amination reactions often enable greener and shorter synthetic routes to 2° and 3° chiral amines by providing a direct and selective pathway to these amines without a further *N*-alkylation step.^3^^,^^4^ A transaminase route, for instance, can only form 1° amines, and further *N*-alkylation step(s) and toxic reagents are required to access the corresponding 2° and 3° amine derivatives.^5^ Abiotic reductive amination reactions, on the other hand, suffer from poor stereoselectivity and often yield the target products as racemic mixtures or in sub-optimal optical purity that require a further and tedious chiral resolution of the enantiomers. In addition, chemical reductive amination methods employing stoichiometric amounts of reducing reagents (such as NaBH_4_, NaBH(OAc)_3_, and NaBH_3_CN) produce large quantities of undesired boronic or cyanide by-products.^6^ Specialized and potentially hazardous equipment setups and high catalyst loadings are usually necessary in transition-metal-catalyzed reductive amination reactions where hydrogenation is performed with dihydrogen gas (using H_2_/transition metal systems).

Since chiral amines are among the most important building blocks in the synthesis of active pharmaceutical ingredients (APIs), asymmetric amine-forming catalytic systems such as those afforded by IREDs are highly sought after. Recent examples of successful industrial exploitation of IRED/RedAm technology^7^^,^^8^^,^^9^^,^^10^ demonstrate considerable interest in this enzyme family. This translational success fuels the motivation to establish the biocatalytic IRED route as a mainstream method of choice for the asymmetric reductive amination of carbonyl compounds. To realize this ambition, the narrow substrate scope of IREDs must be significantly expanded, as several frequently used reductive amination substrates, such as aromatic/bulky ketones and amines, are only poorly tolerated by existing IRED toolkits.^1^^,^^11^^,^^12^

**Figure 1:**
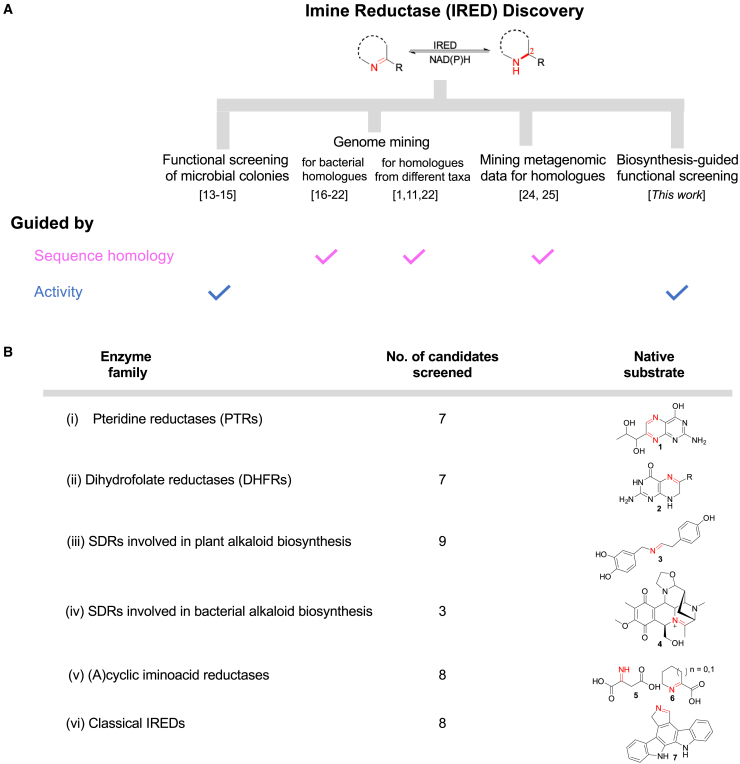
Past and present enzyme discovery approaches to identify IREDs (A) Approaches are classified by exploratory principle: either by searching for sequences that are homologous to the few known IREDs or by testing candidates experimentally (regardless of homology to known IREDs), with references to exemplify these. (B) In this work, a systematic functional exploration of potentially promiscuous biosynthetic C=N bond-reducing enzymes toward the reduction of non-native imine substrates was performed. The summary shows enzyme classes as named by their diverse known reactions with their native substrates. Compounds: pteridine **1**, dihydropterin **2,** norcraugsodine **3**, naphthyridinomycin iminium **4**, imminosuccinate **5**, (1)-piperideine/pyrroline-2-carboxylate **6,** and pyrrole indolocarbazole core **7**. See also the supplemental information, Table S1, and Figure S1.

This problem notwithstanding, the effort devoted to the characterization of synthetically useful IREDs has been remarkable, with several academic and industry groups contributing to the field. These efforts have, over the past decade, yielded hundreds of experimentally characterized IREDs (Figure 1A). The availability of *Streptomyces*’ IREDs as query sequences, identified through the screening of microbial cultures,^13^^,^^14^^,^^15^ paved the way for several genome mining IRED discovery projects in which putative bacterial homologs were identified by sequence homology and experimentally validated.^16^^,^^17^^,^^18^^,^^19^^,^^20^^,^^21^^,^^22^ Through sequence homology search, several homologs of the characterized enzymes were assembled to construct the IRED Engineering Database.^23^ Others have assessed the distribution of IREDs across different taxa, opening up fungal, plant, and metazoan IRED sequence space.^1^^,^^11^^,^^22^ Lastly, a sequence-based metagenomic approach has recently been explored to further expand the IRED toolbox^24^^,^^25^ (Figure 1A). The aforementioned IRED discovery strategies have relied on sequence homology to the extent that the vast majority of known IREDs are closely related, homologous proteins. The narrowly defined sequence window implies that they share similar functional, catalytic, or synthetic properties and limitations or potential for directed evolution. It is, therefore, not surprising that the hugely expanded IRED toolbox has not yet translated to a significant extension of the substrate scope of this enzyme class, highlighting the need for a discovery effort that is not driven by sequence homology alone but instead primarily by function. Exploration of alternative enzyme families that catalyze imine reduction has been considered with some promising results,^22^^,^^26^^,^^27^ but an extensive functional investigation of multiple distinct enzyme families for biocatalytic application as IREDs is not on record.

This work addresses whether IREDs can be found using a sequence-independent strategy to reveal functional proteins in phylogenetically distinct sequence space that transcends the currently narrow sequence definition of contemporary IREDs. Specifically, our strategy explores enzymes that have been shown to reduce theC=N bond of biosynthetic intermediates in different biosynthetic/metabolic pathways across almost all clades of life. Even though biosynthetic enzymes are often perceived to display strict substrate specificity toward their native substrates,^28^^,^^29^^,^^30^^,^^31^ catalytic and substrate promiscuity is a recognized, intrinsic property of many enzymes that allow them to act on alternative substrates, as an evolutionary “head start” en route to the acquisition of a new function after gene duplication.^32^^,^^33^^,^^34^ Here, we systematically explore whether such C=N bond-reducing enzymes are promiscuous toward non-native imine substrates relevant for IRED applications and whether this search, only driven by a functional but not sequence-defined search profile, would uncover novel IREDs in areas of sequence space that is remote from the current cluster of known IREDs. Our approach involves (1) the selection of functionally and phylogenetically divergent enzyme families based on their recorded physiological role as NAD(P)H-dependent C=N reductases in the literature, regardless of sequence homology, (2) a preference for reductases that work on relatively large substrate molecules in the center of which the C=N reduction occurs (to bias for a large binding site able to accept bulky IRED substrates, for which few catalysts exist), and (3) experimental testing of recruited sequences per each selected functional family to determine the number of hits for each selected functional family, their activity, and their stereoselectivity. For each selected family, an average of seven sequences with varying degrees of sequence identity (35%–80%) were screened.

## Results and discussion

### Construction of a diverse enzyme collection

We identified a panel of 44 phylogenetically diverse C=N reducing enzymes covering a wide range of functional families, each with distinct native imine substrate specificity (Figures 1B and S1; Table S1; supplemental information). Specifically the panel comprises enzymes acting on pterin **1** and pteridine **2** such as (1) dihydrofolate reductases (DHFRs) and (2) pteridine reductases (PTR1s; PruAs), respectively; (3) short-chain dehydrogenases (SDRs) catalyzing C=N bond (instead of C=O) reduction in the biosynthesis of plant alkaloids, e.g., norcraugsodine **3**^35^^,^^36^^,^^37^^,^^38^^,^^39^; and (4) bacterial enzymes including naphthyridinomycin biosynthetic enzyme (NAPW) and homologs catalyzing C=N reduction of the iminium precursors of tetrahydroisoquinoline (THIQ) antibiotics, e.g., naphthyridinomycin iminium **4**.^40^ Others include (5) imino acid reductases such as those catalyzing C=N reduction in the metabolism of acyclic imino acids, e.g., iminosuccinate **5**,^28^^,^^41^ as well as those catalyzing the reduction of cyclic imino acids, e.g., 1-peperideine/pyrroline-2-carboxylate **6**.^42^^,^^43^^,^^44^ (6) Lastly, representatives of the “classical” IRED family catalyzing imine reduction in biosynthetic pathways. For example, RedE, an uncharacterized metagenomic IRED-like biosynthetic enzyme acting on pyrrole/pyrrolinium indolocarbazole core **7**, a precursor of tryptophan dimer natural product,^45^ as well as related homologs from Antarctica (from *Mortierella antarctica* [*Ma*RedAm]) and a tropical habitat (from a metagenomic bacterium [*Bac*RedAm]).

**Figure 2:**
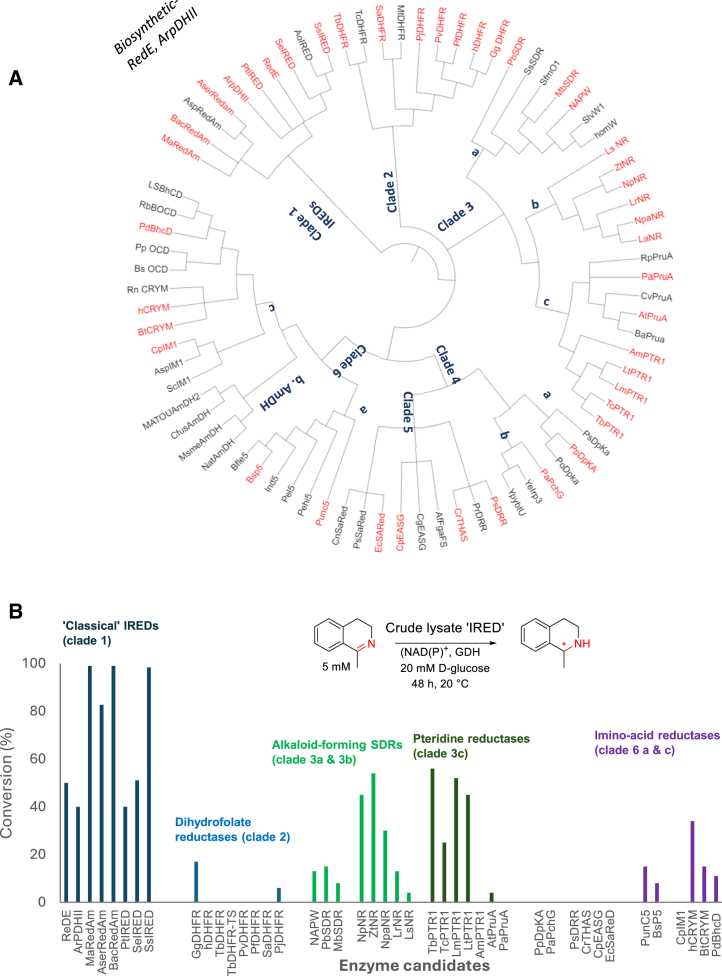
Systematic evaluation of NAD(P)H-dependent C=N bond-reducing (biosynthetic) enzymes for promiscuous IRED activity (A) A cladogram showing highly divergent (biosynthetic) enzymes recruited for this study (in red) from distant or unrelated clades. (B) HPLC conversion obtained from analysis of biotransformation reactions using recombinantly expressed (in *E. coli*) (biosynthetic) C=N bond-reducing enzyme/homolog against a model non-native isoquinoline imine substrate. For NAPW, *Pb*SDR, and *Mb*SDR, conversion values for the reduction of the imine myosmine are presented. Hit rate (%) = (number of enzyme candidates affording detectable product formation as monitored by reverse phase-HPLC/total number enzymes screened in that family) × 100. Hit rate (%) for clade 1 = 100%; clade 2 = 25%; clade 3 = 87%; clades 4 and 5 = 0%; and clades 6a and 6c = 66%.

A cladogram of these divergent sequences reveals distinct clusters (Figure 2A), namely clades 1–6, with each clade containing further monophyletic group(s) (e.g., clades 3a, 3b, and 3c and clades 6a, 6b, and 6c). A sequence similarity network analysis^46^^,^^47^ showed a similar pattern of clustering (Figure S2; supplemental information). Using clade 1 (containing classical IREDs) as a reference clade, generated percent sequence identity (PID) matrices clearly show each clade as, indeed, a unique sequence space with significantly low PIDs of 10%–17% to known IREDs (Figures S3–S5; supplemental information).

### Screening for NADPH-dependent imine-reducing activity on non-native substrates

Representative sequences for each clade were chosen and cloned into pET-based vectors (pET28a, pET15b, and pET22b) (see supplemental information for gene sequences). These plasmids were each expressed in *E. coli* BL21(DE3) to produce recombinant whole-cell biocatalysts (supplemental information; Figures S20 and S21). A preliminary screening for NAD(P)H-dependent imine-reducing activity was performed by monitoring the high-performance liquid chromatography (HPLC) conversion of the imine 1-methyl-3,4-dihydroisoquinoline **8** to the corresponding amine **9** and/or bioreduction of myosmine **10** to nornicotine **11** following 48 h incubation with lysates of recombinant IRED-expressing *E. coli* cells in lysozyme-based lysis buffer (pH 7.0). These substrates have been chosen for an initial affirmation of IRED function because, empirically, the majority of synthetically useful IREDs have been found to reduce isoquinoline and pyrroline imines. In addition, isoquinolines and pyrrolidines are prevalent motifs in many pharmaceuticals.

Analysis of biotransformation reactions revealed that ∼60% of the biosynthetic enzymes from functionally diverse classes displayed imine-reducing activity on one or both non-native imine substrates **8** and **10** (Figure 2B), although group-specific hit rates ranged from 0% to 100%. Of the eight members investigated from clade 1 (REDE homologs, the classical IREDs), all displayed imine-reducing activity toward **8** or **10**, representing a 100% hit rate. In contrast, only two members, *Pj*DHFR from *Pneumocystis jirovecii* (fungal) and *Gg*DHFR from *Gallus gallus* (avian), of the eight screened from clade 2 (DHFRs) displayed weak imine-reducing activity toward **8**, equivalent to a 25% hit rate. 87% (13) of the 15 members of clade 3 (PTR1s, norcraugsodine reductases [NRs], and NAPW-like enzymes) converted either **8** or **10** to the corresponding amine. The protozoan PTR1s and NRs from plants afforded moderate conversions, with the other hits in this clade only displaying weak activity (Figure 2B).

Six members of clades 4 and 5 were screened, but no detectable product was observed under the biotransformation conditions based on HPLC analysis. For clades 6a and 6c (imino acid reductases), six members were also screened, of which 66% displayed weak imine-reducing activity toward **8**. These include the acyclic imino acid reductase PunC5/BsP5 from *Paenibacillus* sp.*/Bacillus* sp., iminosuccinate reductase *Pb*BcHD from *Paracoccus denitrificans*, and the mammalian ketimine reductase mu-crystallin (CRYM). It is worth mentioning that some of these enzymes (e.g., DHFRs, NAPW, PunC5, BsP5) were unstable and enzymatic imine-reducing activity appears to vary with each enzyme batch. Native amine dehydrogenases (NatAmDHs) that form a distinct branch (6b) within clade 6 were excluded from this study, as significant effort has recently been devoted to their biocatalytic exploitation.^24^^,^^48^

Using semi-purified enzyme preparations, we examined the stereoselectivity of selected representatives of clades 1, 2, 3, and 6 toward the reduction of imines **8** and **10** (Table 1). RedE displayed weak activity toward **8** but efficiently reduced myosmine **10** to the corresponding *S*-nornicotine **11** (90% conv., >99% enantiomeric excess [*e.e.*]), while related homologs *Ma*RedAm, *Bac*RedAm, and and the reductive aminase from *Aspergillus sergii (Aser*RedAm) efficiently reduced **8** to the (*R*)- amine **9** (>99% conv., 48%–92% *e.e.*). *Bac*RedAm and *Ma*RedAm also efficiently converted **10** to the (*S*)-amine (>99% conv.), whereas *Aser*RedAm displayed weaker activity toward this substrate.

**Table 1:** Biotransformation for the enantioselective reduction of non-native imines catalyzed by a panel of highly divergent (biosynthetic) enzymes


Entry	Enzymes	8		10	
Enzymes	Functional family	Conv. (%)	*e.e.*% (Abs. conf.)	Conv. (%)	*e.e.%* (Abs. conf.)
ReDE	classical IRED (clade 1)	<5	N/D	79	70 (*S*)
*Ma*RedAm	classical IRED (clade 1)	>99	48 (*R*)	>99	31 (*S*)
*Bac*RedAm	classical IRED (clade 1)	>99	92 (*R*)	>99	98 (*S*)
*Aser*RedAm	classical IRED (clade 1)	>99	89 (*R*)	15	95 (S)
*Gg*DHFR	dihydrofolate reductase (clade 2)	48	>98 (*R*)	N/D	N/D
*Lt*PTR1	pteridine reductases (clade 3a)	>99	96 (*R*)	79	90 (*R*)
*Tb*PTR1	pteridine reductases (clade 3a)	56	13 (*S*)	58	84 (*R*)
*Lm*PTR1	pteridine reductases (clade 3a)	78	54 (*S*)	>99	98 (*R*)
*Tc*PTR1	pteridine reductases (clade 3a)	67	94 (*R*)	n.t.	n.t.
*Zt*NR	alkaloid-forming SDR (clade 3b)	>99	25 (*R*)	75	30 (*R*)
*Np*NR	alkaloid-forming SDR (clade 3b)	89	20 (*R*)	60	29 (*S*)
*Lr*NR	alkaloid-forming SDR (clade 3b)	65	93 (*R*)	45	96 (*R*)
*Pb*SDR	alkaloid-forming SDR (clade 3b)	<3	n.d.	68	91 (*S*)
*Hs*Crym	imino acid reductase (clades 6a and 6c)	68	>98 (*R*)	<3	n.d.
PunC5	imino acid reductase (clades 6a and 6c)	76	>98 (*R*)	<3	n.d.
*Pd*BhcD	imino acid reductase (clades 6a and 6c)	85	>98 (*R*)	8	n.d.


N.B. Reaction conditions for scheme **a**: 5 mM imine, 20 mM D-glucose, 0.5 mM NADP^+^, 0.25–1 mg mL^−1^ IRED (reactions were performed with semi-purified IRED preparation), 0.25 mg mL^−1^ glucose dehydrogenase (GDH; cell-free extract), in 0.5 mL phosphate buffer (100 mM with 100 mM NaCl, pH 7.0). The reaction was incubated at 20°C for 12–48 h. Reaction conditions for scheme **b**: 10–25 mM imine, 30–50 mM glucose, 1 mM NADP^+^, 1–2 mg mL^−1^ IRED, 0.5 mg mL^−1^ GDH (lyophilized cell-free extract), in phosphate buffer (100 mM with 100 mM NaCl, pH 7.0). The reaction was incubated at 25°C for 48 h. The absolute configurations of biotransformation products were determined by comparing the selectivity observed here to previously characterized *Ao*IRED^49^ and *Asp*RedAm^1^ under the same reaction conditions and using the same HPLC chiral columns and screening methods. n.t., not tested; N/D, product not detected; n.d., not determined; Abs. conf., absolute configuration.

Protozoan PTR1s from kinetoplastid parasites, including from *Trypanosoma brucei* (*Tb*PTR1), *T. cruzi* (*Tc*PTR1), *Leishmania major* (*Lm*PTR1), and *L. tarentolae* (*Lt*PTR1), reduced both **8** and **10.** While *Lm*PTR1 and *Tb*PTR1 reduced **8** to the corresponding (*S*)-**9**, albeit in moderate to low *e.e.* (13%–54% *e.e*.), *Tc*PTR1 and *Lt*PTR1 produced the corresponding (*R*)-amine in excellent enantioselectivity (up 96% *e.e*.). The PTR1s all yielded the (*R*)-**11** from the reduction of **10,** irrespective of their selectivity toward amine **9**. The avian DHFR (*Gg*DHFR) reduced **8** to (*R)****-*9** (97% conv., >98% *e.e.*), but no conversion of **9** to **10** could be detected.

The plant alkaloid-forming biosynthetic enzyme NR (*Np*NR from *Narcissus pseudonarcissus*), which has been previously investigated for imine reduction of other cyclic imine substrates,^19^ and homologs from *Zephyranthes treatiae* (*Zt*NR) and *Lycoris radiata* (*Lr*NR) afforded high conversion of **8** to (*R*)-**9,** albeit with poor *e.e.* values. However, *Zt*NR and *Np*NR displayed stereo-complementary selectivity for the reduction of imine **10**, yielding (*S*)-**11** (30% *e.e.*) and (*R*)-**11** (28% *e.e.*), respectively. An alkaloid-forming bacterial dehydrogenase, NAPW-like protein from *Paenibacillus* sp. (*Pb*SDR), formed (*S*)-**11** from **10** (68% conv., 91% *e.e.*). Representatives of (a)cyclic imino acid reductase, including *Homo sapiens ketimine reductase mu-crystallin* (*hs*CRYM), Punc5, and imminosuccinate reductase (β- hydroxyaspartate cyclodeaminase from *Paracoccus denitrificans, Pd*BhCD), yielded (*R*)-**9** with near-perfect selectivity (>98% *e.e.*); however, substrate **10** was barely converted by *hs*CRYM and PunC5 (<3% conv.). Control reactions with stoichiometric amounts of NAD(P)H confirmed the IRED activity and showed a similar trend to the observed activity when using a glucose dehydrogenase (GDH)-recycling system (see supplemental information and Table S2).

All the enzymes investigated in this work displayed a preference for NADPH, although NADH was also accepted, and in some cases, e.g., *Ma*RedAm, *Lt*PTR1, *Zt*NR, and *Am*IRED, showed comparable conversion with both cofactors. The pH profile for the imine-reducing activity of selected enzymes showed optimal activity at pH between 6 and 7, with most of the enzymes also maintaining high activity at pH 5 (see supplemental information and Figure S6). Activity at weakly acidic pH can be useful when handling substrates that are labile at basic pH^7^ and in cascade reactions involving (de)carboxylases.^50^^,^^51^

To further examine the synthetic potential of members of PTR1 and NR families and benchmarking against members of the classical IRED family, we performed biotransformations at higher substrate loading for the reduction of cyclic imines **8** (100 mg preparative scale, 25 mM, 28 mL reaction) and **10** (25 mM, 1 mL reaction), as well as for bulkier imines, salsolidine imine **12** (10 mM, 1 mL reaction) and harmaline **14** (10 mM, 1 mL reaction) (Table 1B). At this elevated substrate loading, the PTR1s, NRs, and IREDs retained moderate to high conversion values for imines **8** and **10** (32% to >99% conv.) to the corresponding enantioenriched products **9** and **11** (Table 1B). Isolated yields and *e.e.* values for the preparative-scale reactions for the reduction **8** to (*R*)-**9** were 92% yield, 95% *e.e.*; 25% yield, 98% *e.e.*; and 30% yield, 24% *e.e.* for *Ma*RedAm-catalyzed, *Lt*PTR1-catalyzed, and *Zt*NR-catalyzed reactions, respectively. For the reduction of imines **12** and **14**, a difference in substrate tolerance could be observed within members of the same family. For example, *Lt*PTR1 showed moderate conversion (41%) toward **12**, yielding (*R*)-**13** (>98% *e.e*.), but only weak or trace activity could be detected with *Tb*PTR1 and *Lm*PTR1 with the same substrate. Harmaline **14** was reduced to the corresponding amine (*S*)-**15,** albeit in low conversion values (6%–18%).

The plant enzyme Z*t*PTR1 was efficient toward the reduction of salsolidine imine **12** (>99% conv., 38% e.e. [*S*]) and harmaline **14** (97% conv., 89% *e.e.* [*R*]), while *Np*NR displayed a slower conversion rate with these substrates. Similar trends were observed with the classical IREDs. *Ma*RedAm efficiently reduced imine **12,** affording the corresponding amine (*R*)-**13** (>99% conv., >98% *e.e*.), but displayed poor activity toward imine **25** (6% conv.). In contrast, *Bac*RedAm reduced **14** to form (*S*)-**15** in excellent conversion and *e.e.* (93% conv., 98% *e.e*.) but only showed weak activity toward the reduction of imine **12.**

The protein sequence space shown to harbor enzymes with imine-reducing activity from this work extends far beyond the classical definition of the IRED sequence space. For example, sequences included in the IRED Engineering Database (www.ired.biocatnet.de), which provides the most extensive coverage of currently known IREDs (>1,400 sequences), fall in their entirety under clade 1 (Figure 2A).^23^ Indeed, a BLAST search against this database using any member of clade 1 as a query sequence returns hits with significant homology scores. However, a similar homology search using representative sequences of all other clades 2, 3, 4, 5, and 6 scanned against the IRED Engineering Database did not return any hits, indicating that the five other distinct enzyme families (clades) described here cover new ground: i.e., are sequence diverse and phylogenetically distinct from known IREDs. This test suggests that the new clades will provide the basis for a significant extension of IRED diversity by functional annotation of proteins in sequence space that act on synthetic imine substrates as IREDs or provide a starting point for their directed evolution.

### Application of representative members in reductive amination

Encouraged by the activity of members of clade 3 on isoquinoline and pyrroline imines and the high imine-reducing hit rate of this group, we became interested in the prospect of these biosynthetic enzymes for the reductive amination of ketones with alkylamines, given the synthetic usefulness of this transformation. Hence, alongside members of clade 1, we examined the performance of representative members of clade 3 toward the reductive amination of simple cyclic ketones (cyclohexanone **16**, cycloheptanone **17**, cyclopentanone **18**, cyclobutanone **19**, 4-phenylcyclohexanone **20**) with alkylamines (propargylamine **a**, cyclopropylamine **b**, methylamine **c)** and ammonia, **d** (Table 2).

**Table 2:** Reductive amination of carbonyl compounds catalyzed by (biosynthetic) IREDs



Enzyme	Conversion (%)							
	16a	16b	16c	17a	17b	17c	18a	18b
*Ma*RedAm	>99	95	>99	98	61	88	94	53
*Bac*RedAm	>99	>99	>99	86	50	66	80	66
*Lt*PTR1	78	62	55	87	29	81	82	15
*Tb*PTR1	28	5	23	20	n.t.	16	30	N/D
*Lm*PTR1	41	24	24	22	n.t.	15	25	8
*Zt*NR	22	N/D	6	21	n.t.	n.t.	18	n.t.
*Np*NR	16	N/D	N/D	20	n.t.	n.t.	15	n.t.


Enzyme	Conversion (%)						
	18c	19a	19c	20a	20c	16d	20d
*Ma*RedAm	80	94	90	93	71	90	30
*Bac*RedAm	62	83	74	89	80	15	32
*Lt*PTR1	46	45	23	53	56	8	14
*Tb*PTR1	11	15	n.t.	n.t.	n.t.	N/D	n.t.
*Lm*PTR1	5	21	n.t.	n.t.	n.t.	N/D	n.t.
*Zt*NR	n.t.	N/D	N/D	n.t.	8%	N/D	N/D
*Np*NR	n.t.	N/D	N/D	n.t.	N/D	N/D	N/D

N.B. Reaction conditions: [carbonyls]: **16**, **17**, **18**, and **19** (50 mM) and **20** (10 mM); [amine nucleophile]: **a** and **b** (2 equiv) and **c** and **d** (4 equiv). 100 mM glucose, 0.5 mM NADP^+^, 0.5–1 mg mL^−1^ (semi-)pure IRED, 0.5 mg mL^−1^ glucose dehydrogenase (GDH; cell-free extract), 7.5 mM MgCl_2_ in 0.5 mL 100 mM Tris-HCl (100 mM NaCl, pH 9.0). The reaction was incubated at 20°^C^ for 12–48 h. n.t., not tested; N/D, product not detected.

*Ma*RedAm-catalyzed reductive amination of carbonyls **16**–**19** (50 mM) with alkylamines **a**, **b**, and **c** (2 equiv.) afforded high conversion (**16a**–**16c,** 95% to >99% conv.; **17a**–**17c,** 50%–98% conv., **18a**–**18c,** 53%–94% conv., **19a** and **19c**, 74%–89% conv.), and comparable conversion values were obtained with *Bac*RedAm (Table 2). Similar conversion values were also observed with 4-phenylcyclohexanone **20** (10 mM), with *Ma*RedAm and *Bac*RedAm efficiently coupling amines **a** and **c** to form the corresponding amine products **20a** (89%–93% conv.) and **20c** (71%–80% conv.), respectively.

Members of the PTR1s (clade 3c) displayed similar substrate tolerance to *Ma*RedAm and *Bac*RedAm. *Lt*PTR1, in many cases, afforded comparable conversion values with the prototype RedAms (e.g., **16a**, 78% conv.; **17a**, 87% conv.; **18a**, 82% conv.; **19a**, 45% conv.; **20a**, 53% conv.), whereas *Tb*PTR1 and *Lm*PTR1 yielded these products but in lower conversion values. Other alkylamines, such as cyclopropylamine **b** and methylamine **c**, were also accepted by *Lt*PTR1 as amine nucleophiles, furnishing the corresponding amine products in modest conversion values. *Ma*RedAm-catalyzed amination of **16** (50 mM) with NH_3_ **d** (supplied as 200 mM NH_4_Cl) afforded 90% conv. to the primary amine **16d**, while conversion values were significantly lower with *Bac*RedAm (**16d**, 15% conv.) and *Lt*PTR1 (**16d**, 8% conv.). These enzymes also formed 4-phenylcyclohexylamine **20d** from 4-phenylcyclohexanone **20** and NH_3_, **d** (**20d**, *Ma*RedAm, 32% conv.; *Bac*RedAm, 30% conv.; and *Lt*PR1, 14% conv.).

The plant enzymes (clade 3b) *Zt*NR and *Np*NR displayed activity toward the amination of **16**–**18** with propargylamine **a**, affording the corresponding *N*-alkylated propargylamine products **16a**–**18a**, albeit with significantly lower conversion values. However, both enzymes could not use ammonia or the small alkylamine MeNH_2_ and showed weak activity toward amination of **16** with cyclopropylamine **b** (*Zt*NR, **16b**, 5% conv.) (Table 2).

Preparative-scale biotransformation reactions for the amination of cyclohexanone **16** (100 mg, 1.02 mmol, total reaction volume, 41 mL) with 2 equiv of propargylamine **a** were performed, yielding the corresponding secondary amine **16a** (84% yield for *Ma*RedAm-catalyzed reaction and 63% yield for *Lt*PTR1-catalyzed reaction).

To examine the pattern of substrate specificity for both carbonyl and amine coupling partners, we carefully constructed a small substrate panel containing ketones **16** and **22**, aldehyde **21**, α-keto ester **23**, and α-keto acids **24** and **25** as carbonyl acceptors, and propargylamine **a**, cyclopropylamine **b**, methylamine **c,** and ammonia **d** were included as the amine coupling partners (Table 3). Using purified enzyme preparation, representative members of clades 1, 2, 3, 5, and 6 were each screened against the various substrate combinations of this substrate panel, monitoring the NADPH-dependent reductive amination initial rate at 340 nm using a microtiter plate reader (Table 3).

**Table 3:** Comparison of substrate specificity of representative members from distinct enzyme families using a small substrate panel


Specific activity (U mg^−1^)							
Amine nucleophile	Carbonyl acceptor	*Ma*RedAm (clade 1)	*Lt*PTR1 (clade 3c)	*Pp*Dpka (clade 4)	*h*CRYM (clade 6c)	Avian DHFR (clade 2)	PunC5 (clade 6a)
	16	7.726	0.021	–	–	–	–
	**21**	9.794	0.041	–	–	–	–
	**22**	0.266	0.015	–	–	–	–
	**23**	0.052	–	3.975	–	–	–
	**24**	0.029	–	4.326	0.055	0.048	0.133
	**25**	0.041	–	0.216	–	–	–
	**16**	10.300	0.034	–	–	–	–
	**21**	11.629	0.034	–	–	–	–
	**22**	0.732	0.012	–	–	–	–
	**23**	0.079	–	4.602	–	–	–
	**24**	0.018	–	4.713	0.039	0.047	0.097
	**25**	0.044	–	0.215	–	–	–
	**16**	6.976	0.025	–	–	–	–
	**21**	1.972	0.013	–	–	–	–
	**22**	0.127	0.005	–	–	–	–
	**23**	0.044	–	4.770	0.022	0.005	0.024
	**24**	–	–	4.752	0.113	0.097	0.168
	**25**	0.031	–	0.015	–	0.023	0.096
	**16**	0.434	0.007	–	–	–	–
	**21**	0.324	0.009	–	–	–	–
	**22**	–	–	–	–	–	–
	**23**	–	–	2.799	–	–	–
	**24**	–	–	2.906	–	–	0.028
	**25**	–	–	0.554	–	–	–

N.B. Cells with en dashes mean that activity was not detected under the screening conditions. The following screening conditions were used: reactions contain 5–10 mM ketones, 60 mM amine nucleophile (100 mM for ammonia) added to reaction mixture from 1 M pH adjusted stock solution (pH 9), and 0.5 mM NADPH. The reaction was performed in Tris-HCl buffer (100 mM, pH 9, supplemented with 7.5 mM MgCl_2_), and 0.05–0.6 mg mL^−1^ IRED was added to start the reaction. The initial reaction rate was monitored at 340 nm using a microtiter plate reader.

*Ma*RedAm (clade 1) was the most versatile of these catalysts, displaying high to moderate activity for the ketone/aldehyde and alkylamine combinations, with specific activity of up to 11.6 U mg^−1^. *Ma*RedAm-catalyzed reductive amination activity was one order of magnitude lower when ammonia was used instead of alkylamines (Table 3). *Ma*RedAm also exhibited reductive amination activity toward the α-keto ester **23** and α-keto acids, **24** and **25**, albeit two orders of magnitude slower relative to the rates observed with cyclohexanone/hydrocinnamaldehyde and alkylamines. *Lt*PR1, a representative from clade 3c, showed similar amine specificity to *Ma*RedAm. However, reaction velocities were 10- to 100-fold slower when compared with *Ma*RedAm for the same substrate combinations. Activity toward α-keto acids was not detected under the screening conditions. Pseudomonas putida ketimine reductase (*Pp*Dpka) showed similar amine substrate specificity to *Ma*RedAm but distinct specificity for the carbonyl acceptor. *Pp*Dpka exhibited high specific activity toward the amination of α-keto ester **23** and α-keto acids **24** and **25** with alkylamines (up to 5 U mg^−1^); activity toward ketones **16** and **22** and aldehyde **21** was not detected under the conditions of this assay. *Hs*CRYM (clade 6c), PunC5 (clade 6a), and the avian DHFR (*Gg*DHFR, clade 6a) displayed activity for α-keto acids/esters with alkylamines and, in some cases, ammonia (e.g., PunC5); however, methylamine **c** was the preferred alkylamine nucleophile for these substrates.

The screening against this carefully designed, albeit small, substrate panel has revealed that several non-homologous enzyme families investigated can catalyze reductive amination with primary amines to form *N*-alkylamines, *N*-alkylamino esters, and *N*-alkylamino acids (Table 3). Hence constructing IRED panels to contain representative members from these distinct and non-homologous enzyme families should significantly extend the amine product scope that can be accessed compared to conventional IRED kits.

To demonstrate the utility of observed activities for biotransformation reactions, we focused on PTR1s and NRs (again benchmarking against the classical IREDs in clade 1) for the reductive amination of hydrocinnamaldehyde **21** (30 mM), benzaldehyde **26** (10 mM), and a prochiral ketone, 4-phenyl-2-butanone **22** (10 mM). PTR1s efficiently catalyzed the amination of hydrocinnamaldehyde with propargylamine **a** to form the corresponding secondary amine coupling product **21a** (54%–95% conv.) as well as the coupling of benzaldehyde **17** with **a**, yielding norpargyline **26a** (up to >99% conv.). However, PTR1s only showed low conversion (up to 6%) when tasked with the amination 4-phenyl-2-butanone **22** with propargylamine **a** or allylamine **e**. Similarly, NRs catalyzed the amination benzaldehyde **26** with **a** to afford the corresponding amine **26a** in high conversion (up >99%) but displayed weak amination for hydrocinnamaldehyde **21** and only trace activity for the amination of **22**. In contrast, the classical IREDs/RedAms performed well across these substrates, affording high conversion of up to >99%. For the reductive amination of ketone **22** with amines **a** or **e**, the (*R*)-configured amine products ((*R*)**-22a**, and (*R*)-**22e**) were generated with moderate to high enantioselectivity of up to 95% *e.e.* Although this preliminary screen shows that PTR1s and NRs are suitable for the amination of aromatic aldehydes and simple cyclic ketones and less efficient for the amination of (aromatic) ketones, an extensive substrate profiling study is needed to map out the distinctive substrate specificities of PTR1s and NRs, as well as the other enzyme families described in this work.

The classical IREDs emerged as the most versatile of these enzyme families for synthetic application in the reductive amination of ketones. Hence, we further investigated the efficiency of the novel IREDs/RedAms identified in this work (Table S1) toward the amination of difficult-to-aminate bicyclic aromatic ketones such as 1-indanone **27** and 1-tetralone **28**. The synthesis of α-secondary amines from bicyclic aromatic ketones represents one of the most challenging reactions for existing IREDs/RedAms.^52^ Achieving this transformation remains hugely attractive, as corresponding amine products formed from this reaction are prevalent in pharmaceutical drugs. To this end, we screened 9 members of clade 1 (classical IREDs) for the amination of **27** with methylamine **c**, revealing *Bac*RedAm, *Ma*RedAm, and *Aser*RedAm as the best-performing members of this clade for these substrates.

**Figure 3:**
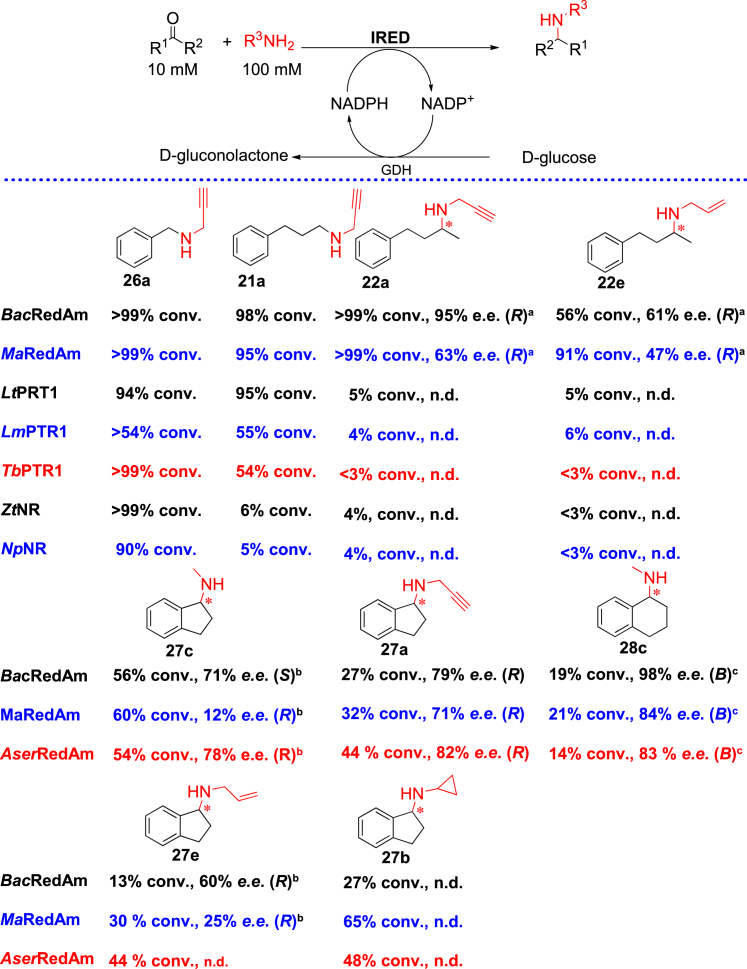
Investigation of the performance of PTR1s, NRs, and classical IREDs for the reductive amination of aromatic ketones and aldehydes Pteridine reductases (PTR1s) and norcraugsodine reductases (NRs) were able to catalyze the amination of aromatic aldehydes but showed only weak/trace activity toward the amination of aromatic ketones. Novel reductive aminases identified from this work catalyzed the stereoselective reductive amination of aromatic ketones for the synthesis of α-chiral secondary amines. A glucose dehydrogenase (GDH)-based cofactor recycling system was employed, with glucose used as a sacrificial oxidant. Reaction conditions: ketone substrate (10 mM), amine nucleophile (100 mM), 0.5 mM NAD(P)^+^, 20 mM D-glucose, 0.3 mg mL^−1^ GDH lyophilized cell-free extract, purified RedAm/IRED 1 mg mL^−1^, 2% v/v DMSO. The reaction was performed in Tris-HCl buffer (100 mM, pH 9) at 30°C and 230 rpm for 24 h. Conversion and enantiomeric excess (*e.e*.) values were determined by normal-phase HPLC analysis using chiral columns. ^a^Absolute configuration assigned based on comparison of the elution pattern of the *N-*methylated analog. ^b^Absolute configurations were assigned based on comparisons of the elution pattern of the *N*-propargyl analog. ^c^Absolute configuration not determined; designation of “A” or “B” represents the order of the elution of the enantiomers on chiral HPLC, with enantiomer “A” eluting first.

The amination of 1-indanone with methylamine **c** formed the corresponding α-secondary amine product **27c** with modest conversion values (54%–60%). *Ma*RedAm and *Aser*RedAm produced the (*R*)-**27c** with *e.e.* values of 12% and 78%, respectively, while *Bac*RedAm generated (*S*)-**27c** (71% *e.e*.) (Figure 3; see also supplemental information and Figure S7). Interestingly, *Bac*RedAm-catalyzed reductive amination of **27** with propargylamine **a** afforded the (*R*)-configured amine product rasagaline **27a** (27% conv., 79% *e.e*.), indicating that the amine nucleophile can play a role in the stereochemical outcome of RedAm-catalyzed reductive amination. Both *Ma*RedAm and *Aser*RedAm also afforded rasagiline (*R*)-**27a** (*Ma*RedAm: 32% conv., 71% *e.e*.; *Aser*RedAm: 44% conv., 82% *e.e*.), retaining the selectivity observed with *N*-methylated product **27c** (Figure 3). The RedAm-catalyzed amination with other amine nucleophiles, namely cyclopropylamine **b** and allylamine **e**, yielded the corresponding amine products **27a** and **27e** in moderate conversions of up to 65%. These enzymes also catalyzed the reductive amination of 1-tetralone **28** with methylamine to yield **28c**, a key intermediate of sertraline, albeit in low conversion values (14%–19%) but with good to excellent *e.e.* values of up to 98% (Figure 3).

### Conclusion

In summary, the functional exploration of highly divergent, sequence-unrelated enzyme families based on their C=N reducing biosynthetic roles has identified promiscuous catalysts with IRED activity toward non-native substrates. While accidental enzyme discovery in biosynthetic pathways has been the source of many useful catalysts in the past, here we provide a rationale for IRED mining with high hit rates (average: ∼46%; successful in 4 of 6 clades) via the substrate promiscuity of C=N reducing enzymes. Specifically, we have mapped and annotated hitherto unexplored sequence space by uncovering distinct, unrelated enzyme families for prospecting biocatalysts for enantioselective imine reduction and reductive amination. Clade 3, which comprised PTR1s and alkaloid-forming plants and bacterial SDRs, represents a unique and promising functional annotation of sequence space to find novel enzymes for enantioselective imine reduction and reductive amination with a hit rate >87% for imine-reducing activity for this clade. We showed that members of PTR1s and NRs were able to catalyze the reductive amination of simple cyclic ketones and aromatic aldehydes.

Clade 6 is another promising group to retrieve novel IRED-like biocatalysts; members of this group such as the mammalian ketimine reductase mu-crystallin (CRYM) and iminosuccinate reductases (e.g., *Pb*BhCD) exhibit promiscuous (albeit weak) imine-reducing activity toward non-native cyclic imines, e.g., isoquinoline imine. Importantly, clade 6 also features NatAmDHs, which have been shown to catalyze the IRED-like reductive amination reaction.^7^ Our approach allows the identification of alternative sets of evolutionarily unrelated, non-homologous “isofunctional” enzymes.

A comparative evaluation of the performance of these non-homologous enzyme families in the reductive amination of carbonyl compounds with primary amines using a small panel of carbonyl compounds including ketones, aldehydes, α-keto esters, and α-keto acids highlights differences in substrate specificity. Classical IREDs emerged as the most versatile enzyme class, displaying activity across all the investigated substrate groups and enabling the synthesis of α-secondary amines from difficult-to-aminate aromatic ketones.

Several of the other enzyme classes characterized in this work act on native substrates that can be considered large and hydrophobic, providing spacious hydrophobic binding sites, which should warrant future evaluation of their usefulness in the reductive amination of bulky substrates. Even more desirable is an extensive substrate profiling study using large and structurally diverse carbonyl and amine substrate panels to map out the unique synthetic scope of each of these diverse enzyme families. Catalytic efficiency can further be optimized through protein engineering to provide a versatile toolbox for biocatalytic imine reduction and reductive amination of challenging substrates. In this context, sequence network models^53^ and convolutional neural networks using deep learning models^54^ can serve as useful tools to investigate the sequence and structural features peculiar and common to these divergent enzyme families. Such studies may provide useful mechanistic insights into enzyme promiscuity to allow (semi)-rational enzyme engineering of these enzymes.

## Experimental procedures

### Materials and methods

For more details on the experimental procedures, including materials and chemicals, procedures for the synthesis of chemical standards, procedures for cloning, enzyme expression and purification, and biotransformation reactions, see the supplemental information.

## Resource availability

### Lead contact

Requests for further information and resources should be directed to and will be fulfilled by the lead contact, Godwin Aleku (godwin.aleku@kcl.ac.uk).

### Materials availability

All materials generated in this study are available from the lead contact without restriction.

### Data and code availability

This study did not generate any datasets.

## Acknowledgments

This work received support from the 10.13039/501100000275Leverhulme Trust through a Leverhulme Early Career Fellowship to G.A.A. (ECF-2020-694). G.A.A. also received support through the Isaac Newton Trust Early Career Fellowship and 10.13039/501100000288The Royal Society (RGS\R1\231514). Further support was provided by the 10.13039/501100000268BBSRC (BB/T003545/1). F.H. is an ERC Advanced Investigator (695669) and member of the EU Horizon consortium BlueRemediomics (101082304) with support from UKRI. The authors would like to thank Dr. Melanie A. Higgins for kindly providing us with Bsp5 and Punc5 plasmids and Dr. Liisa van Vliet for kindly providing us with the *Pv*DHFR and *Pf*DHFR plasmids.

## Author contributions

G.A.A. conceived and designed the experiments with input and guidance from F.H. G.A.A. performed all experiments and analyzed the data. G.A.A. and F.H. wrote the paper.

## Declaration of interests

The authors declare no competing interests.

## Supplemental information

Document S1. Figures S1–S21, Tables S1–S4, supplemental experimental procedures, and supplemental references Supplemental information contains detailed experimental procedures, chromatograms for product analysis (Figures S7–S19), gene sequences, protein sequence accession numbers (Table S1) and network and sequence analyses (Figures S2-S5), pH rate profiles (Figure S6), and protein expression trials (Figures S20 and S21). The Supplemental information also contains a list of enzymes and their physiological roles and their native substrates (Table S1; Figure S1), biotransformation data using stoichiometric amounts of NADPH and NADH (Table S2) and a list of substrates tested and HPLC/GC-MS analysis methods (Tables S3 and S4), and can be found online at https://doi.org/10.1016/j.checat.2024.101160. The authors have cited additional references within the supplemental information.^S1,S2^

Data S1. *Chem Catalysis* checklist

Document S2. Article plus supplemental information
