# Supplementary material for: Expanding the repertoire of imine reductases by mining divergent biosynthetic pathways for promiscuous reactivity
Source: Chem Catal. 2024 Dec 19;4(12):101160. doi: 10.1016/j.checat.2024.101160 (PMC11876095; doi:10.1016/j.checat.2024.101160)
Supplement: Document S1. Figures S1–S21, Tables S1–S4, supplemental experimental procedures, and supplemental references — Supplemental information contains detailed experimental procedures, chromatograms for product analysis (Figures S7–S19), gene sequences, protein sequence accession numbers (Table S1) and network and sequence analyses (Figures S2-S5), pH rate profiles (Figure S6), and protein expression trials (Figures S20 and S21). The Supplemental information also contains a list of enzymes and their physiological roles and their native substrates (Table S1; Figure S1), biotransformation data using stoichiometric amounts of NADPH and NADH (Table S2) and a list of substrates tested and HPLC/GC-MS analysis methods (Tables S3 and S4), and can be found online at https://doi.org/10.1016/j.checat.2024.101160. The authors have cited additional references within the supplemental information.S1,S2 [file mmc1.pdf]

Chem Catalysis, Volume *4*

Supplemental information

Expanding the repertoire of imine reductases by mining divergent biosynthetic pathways for promiscuous reactivity

Godwin A. Aleku and Florian Hollfelder

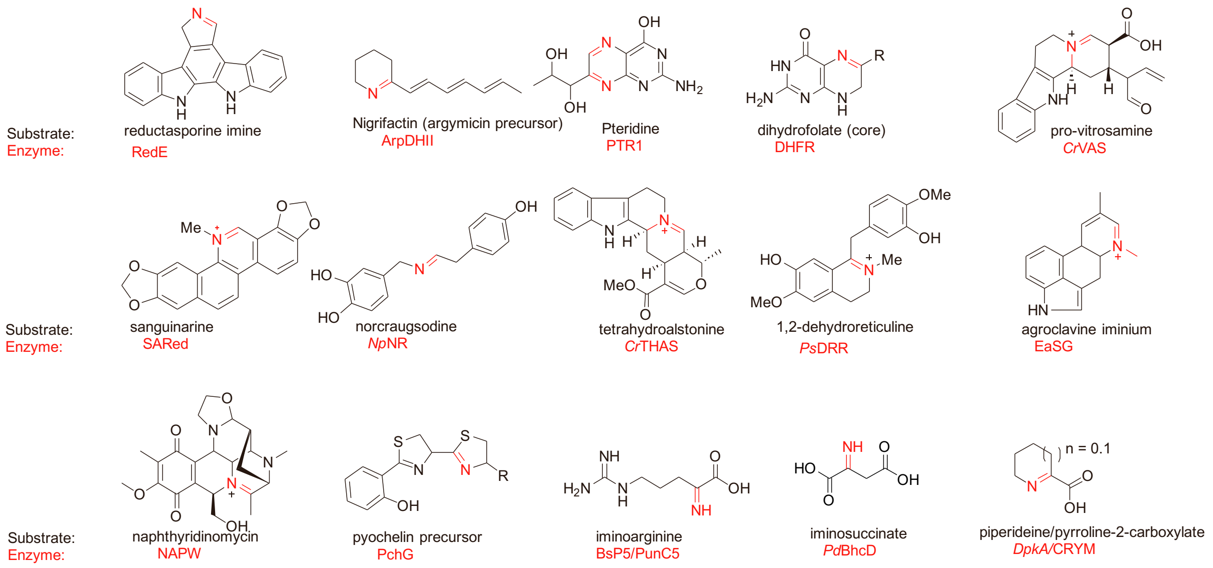

Figure S1. Examples of structurally diverse native imine/iminium substrates and their respective biosynthetic C=N reducing enzymes. RedE, tryptophan dimer biosynthetic enzyme; ArpDHII, Argimycins P dehydrogenase; PTR1, pteridine reductase 1; DHFR, dihydrofolate reductase; *Cr*VAS vitrosamine synthase from *Catharanthus roseus*; SaRed, sanguinarine reductase; NpNR noroxomaritidine/norcraugsodine reductase from *Narcissus pseudonarcissus*; *Cr*THAS, tetrahydroalstonine synthase from C. roseus; *Ps*DRR, 1,2-dehydroreticuline reductase from *Papaver sp*; EaSG agroclavine synthase; NAPW, naphthyridinomycin dehydrogenase; PchG, pyochelin biosynthesis thiazoline reductase; BsP5/PunC5 acyclic imino acid reductase; *Pb*BhcD, Imminosuccinate reductase from *Paracoccus denitriicans.* DpkA/CRYM-ketimine reductase.

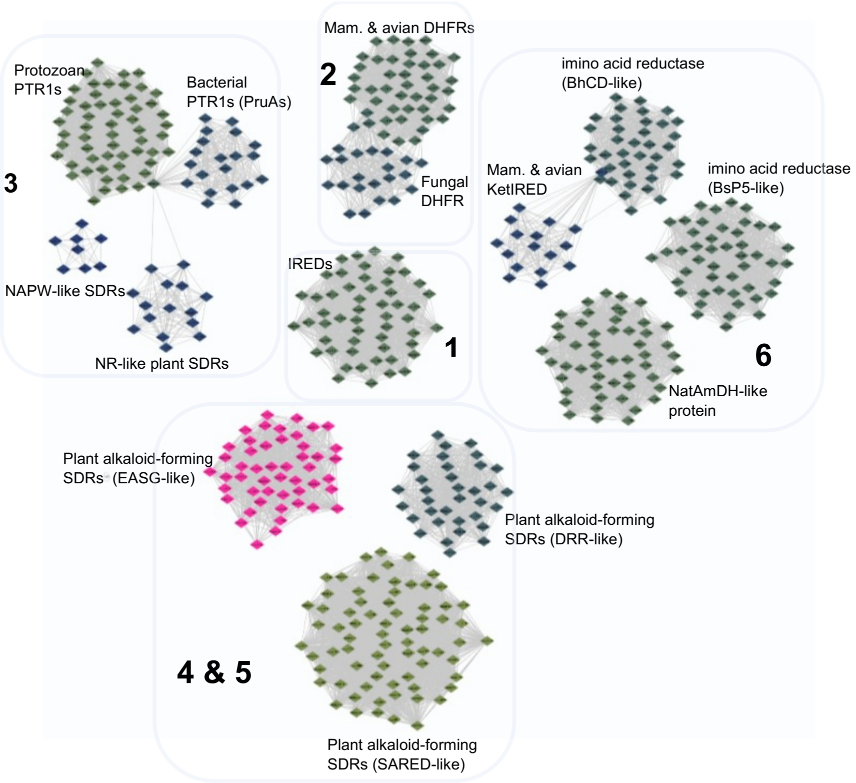

Figure S2. Sequence similarity networks of biosynthetic C=N reducing enzymes from different functional families. Networks were generated using the Enzyme Similarity Tool (EFI-EST)[1] and visualised with Cytoscape.[2] Edges displayed correspond to a median of 27% identity over an alignment length of 350 amino acids. Representatives' clades. IREDs, imine reductases; DHFR, dihydrofolate reductase; PTR1, pteridine reductases; PruAs, bacterial pteridine reductase; SDR, short-chain dehydrogenase/reductase; KetIRED, ketimine reductases/CRYMs; NR, *norcraugsodine reductase, NAPW,* naphthyridinomycin dehydrogenase; NatAmDH, native amine dehydrogenase; DRR, dihydroreticuline reductase; EaSG, agroclavine synthase (ergot alkaloid biosynthesis). A comprehensive list of the enzymes included in this study is presented in Table S1 and a phylogenetic tree (cladogram) is presented in main manuscript, Figure 2.

		IREDs						DHFRs							
		ArpDHII	RedE	SelRED	BacRedAm	MaRedAm	AserRedam	TbDHFR	PjDHFR	hDHFR	GgDHFR	SaDHFR	PvDHFR		
IREDs	ArpDHII	100	39	43	42	36	39	16	15	13	13	16	12	ArpDHII	IREDs
	RedE	39	100	45	44	34	35	12	12	14	14	16	13	RedE	
	SelRED	43	45	100	45	37	38	15	13	13	13	15	11	SelRED	
	BacRedAm	42	44	45	100	53	51	14	16	16	16	14	10	BacRedAm	
	MaRedAm	36	34	37	53	100	53	16	13	16	16	16	12	MaRedAm	
	AserRedam	39	35	38	51	53	100	15	10	13	13	15	10	AserRedam	
DHFRs	TbDHFR	16	12	15	14	16	15	100	30	31	32	27	24	TbDHFR	DHFRs
	PjDHFR	15	12	13	16	13	10	30	100	36	36	31	27	PjDHFR	
	hDHFR	13	14	13	16	16	13	31	36	100	75	30	31	hDHFR	
	GgDHFR	13	14	13	16	16	13	32	36	75	100	31	30	GgDHFR	
	SaDHFR	16	16	15	14	16	15	27	31	30	31	100	32	SaDHFR	
	PvDHFR	12	13	11	10	12	10	24	27	31	30	32	100	PvDHFR	
		ArpDHII	RedE	SelRED	BacRedAm	MaRedAm	AserRedam	TbDHFR	PjDHFR	hDHFR	GgDHFR	SaDHFR	PvDHFR		
		IREDs						DHFRs							

Figure S3. Percent sequence identity matrix comparing classical imine reductases (IREDs)/reductive aminases (RedAms) with dihydrofolate reductases (DHFRs, clade 2). The percent identity matrix was generated using ClustalW.

		IREDS							NAPW-like SDRs			NR-like SDRs						PTR1s				
		ArpDHII	RedE	SsIRED	SeIRED	BacRedAm	MaRedAm	AserRedam	MbSDR	PbSDR	NAPW	LsNR	NpaNR	LaNR	LrNR	NpNR	ZtNR	AmPTR1	LtPTR1	LmPTR1	TbPTR1	TcPTR1
	ArpDHII	100	38	40	42	41	35	38	12	12	12	11	12	12	12	11	17	15	15	14	11	16
	RedE	38	100	43	45	43	33	34	16	14	15	13	10	11	10	11	12	14	14	12	13	14
	SsIRED	40	43	100	57	46	39	38	14	12	12	12	10	10	12	12	11	15	14	13	15	16
**IREDS**	SeIRED	42	45	57	100	45	37	38	12	10	10	11	11	12	13	12	12	14	13	12	11	16
	BacRedAm	41	43	46	45	100	53	50	14	11	11	16	12	12	11	11	13	17	15	14	14	17
	MaRedAm	35	33	39	37	53	100	52	14	12	12	11	11	12	11	12	12	17	14	11	14	15
	AserRedam	38	34	38	38	50	52	100	10	11	12	13	14	14	12	13	14	14	13	12	14	14
	MbSDR	12	16	14	12	14	14	10	100	55	51	22	23	24	22	24	23	22	21	20	19	21
**NAPW-like SDRs**	PbSDR	12	14	12	10	11	12	11	55	100	74	23	22	23	22	23	22	21	21	21	21	21
	NAPW	12	15	12	10	11	12	12	51	74	100	22	21	22	22	22	22	21	19	19	20	19
	LsNR	11	13	12	11	16	11	13	22	23	22	100	45	46	**45**	48	49	32	27	28	29	26
	NpaNR	12	10	10	11	12	11	14	23	22	21	45	100	90	70	75	70	33	27	26	29	26
**NR-like SDRs**	LaNR	12	11	10	12	12	12	14	24	23	22	46	90	100	71	73	68	33	27	26	32	25
	LrNR	12	10	12	13	11	11	12	22	22	22	45	70	71	100	83	75	34	27	24	27	23
	NpNR	11	11	12	12	11	12	13	24	23	22	48	75	73	83	100	85	36	27	26	30	26
	ZtNR	17	12	11	12	13	12	14	23	22	22	49	70	68	75	85	100	36	29	27	30	26
	AmPTR1	15	14	15	14	17	17	14	22	21	21	32	33	33	34	36	36	100	45	44	44	41
	LtPTR1	15	14	14	13	15	14	13	21	21	19	27	27	27	27	27	29	45	100	80	52	48
**PTR1s**	LmPTR1	14	12	13	12	14	11	12	20	21	19	28	26	26	24	26	27	44	80	100	51	48
	TbPTR1	11	13	15	11	14	14	14	19	21	20	29	29	32	27	30	30	44	52	51	100	56
	TcPTR1	16	14	16	16	17	15	14	21	21	19	26	26	25	23	26	26	41	48	48	56	100

Figure S4. Percent sequence identity matrix comparing classical imine reductases (IREDs)/reductive aminases (RedAms) with members of clade 3 (pteridine reductases, PTR1s; naphthyridinomycin dehydrogenases, NAPW; norcraugsodine reductases, NRs. The percent identity matrix was generated using ClustalW.

		CRYMs							BHCDs									IREDs									
		GcCRYM	SvCRYM	AcCRYM	CmCRYM	RnCRYM	CRYM	BtCRYM	VsBHCD	GaBhCD	PaBhCD	PsBHCD	OtBhCD	AeBhCD	RbBOCD	LSBhCD	RaBhCD	MaRedAm	BacRedAm	AdRedAm	AtRedAm	AspRedAm	AsRedam	SeIRED	AoIRED	SpRedAm	RedE
	GcCRYM	100	91	91	92	80	80	79	29	30	27	28	27	28	27	29	29	10	11	10	12	13	13	14	14	14	10
	SvCRYM	91	100	94	95	79	80	78	29	29	27	29	27	28	27	29	30	10	11	11	11	13	15	14	13	14	11
	AcCRYM	91	94	100	98	77	78	78	28	28	27	28	26	27	27	29	29	10	11	11	12	13	14	14	13	14	11
CRYMs	CmCRYM	92	95	98	100	81	80	81	29	29	28	29	27	28	28	30	30	10	11	11	12	13	13	14	13	14	11
	RnCRYM	80	79	77	81	100	88	89	28	27	26	27	25	26	27	27	27	10	10	10	11	13	13	13	12	12	10
	HsCRYM	80	80	78	80	88	100	90	27	26	26	27	25	25	26	27	27	10	11	10	12	13	13	11	14	12	10
	BtCRYM	79	78	78	81	89	90	100	27	26	26	26	24	25	27	27	27	10	11	10	11	12	12	12	13	12	10
	VsBHCD	29	29	28	29	28	27	27	100	42	38	39	38	39	39	38	38	12	11	12	13	14	14	13	13	14	12
	GaBhCD	30	29	28	29	27	26	26	42	100	61	61	60	66	64	63	62	12	14	12	14	13	13	13	13	15	11
	PaBhCD	27	27	27	28	26	26	26	38	61	100	79	64	70	69	70	70	13	13	12	13	13	12	13	13	15	12
	PsBHCD	28	29	28	29	27	27	26	39	61	79	100	63	68	68	68	68	13	14	14	14	14	14	14	16	16	12
BHCDs	OtBhCD	27	27	26	27	25	25	24	38	60	64	63	100	72	67	67	66	10	14	12	11	11	11	11	11	12	10
	AeBhCD	28	28	27	28	26	25	25	39	66	70	68	72	100	74	71	70	13	15	13	15	14	13	12	12	16	11
	RbBOCD	27	27	27	28	27	26	27	39	64	69	68	67	74	100	75	74	12	13	12	13	12	12	14	11	15	11
	LSBhCD	29	29	29	30	27	27	27	38	63	70	68	67	71	75	100	99	12	12	12	11	13	13	12	14	14	12
	RaBhCD	29	30	29	30	27	27	27	38	62	70	68	66	70	74	99	100	12	12	12	12	14	14	13	14	14	12
	MaRedAm	10	10	10	10	10	10	10	12	12	13	13	10	13	12	12	12	100	53	50	54	51	53	37	38	39	34
	BacRedAm	11	11	11	11	10	11	11	11	14	13	14	14	15	13	12	12	53	100	53	54	50	50	45	45	49	45
	AdRedAm	10	11	11	11	10	10	10	12	12	12	14	12	13	12	12	12	50	53	100	54	52	54	39	41	39	38
	AtRedAm	12	11	12	12	11	12	11	13	14	13	14	11	15	13	11	12	54	54	54	100	59	60	42	42	42	37
	AspRedAm	13	13	13	13	13	13	12	14	13	13	14	11	14	12	13	14	51	50	52	59	100	92	37	36	37	34
IREDs	AsRedam	13	15	14	13	13	13	12	14	13	12	14	11	13	12	13	14	53	50	54	60	92	100	37	38	37	35
	SeIRED	14	14	14	14	13	11	12	13	13	13	14	11	12	14	12	13	37	45	39	42	37	37	100	57	49	46
	AoIRED	14	13	13	13	12	14	13	13	13	13	16	11	12	11	14	14	38	45	41	42	36	38	57	100	48	44
	SpRedAm	14	14	14	14	12	12	12	14	15	15	16	12	16	15	14	14	39	49	39	42	37	37	49	48	100	55
	RedE	10	11	11	11	10	10	10	12	11	12	12	10	11	11	12	12	34	45	38	37	34	35	46	44	55	100

Figure S5. Percent sequence identity matrix comparing classical imine reductases (IREDs)/reductive aminases (RedAms) against members of clade 6c (imino acid reductases, CRYMs and BhCD-like enzyme). The percent identity matrix was generated using ClustalW.

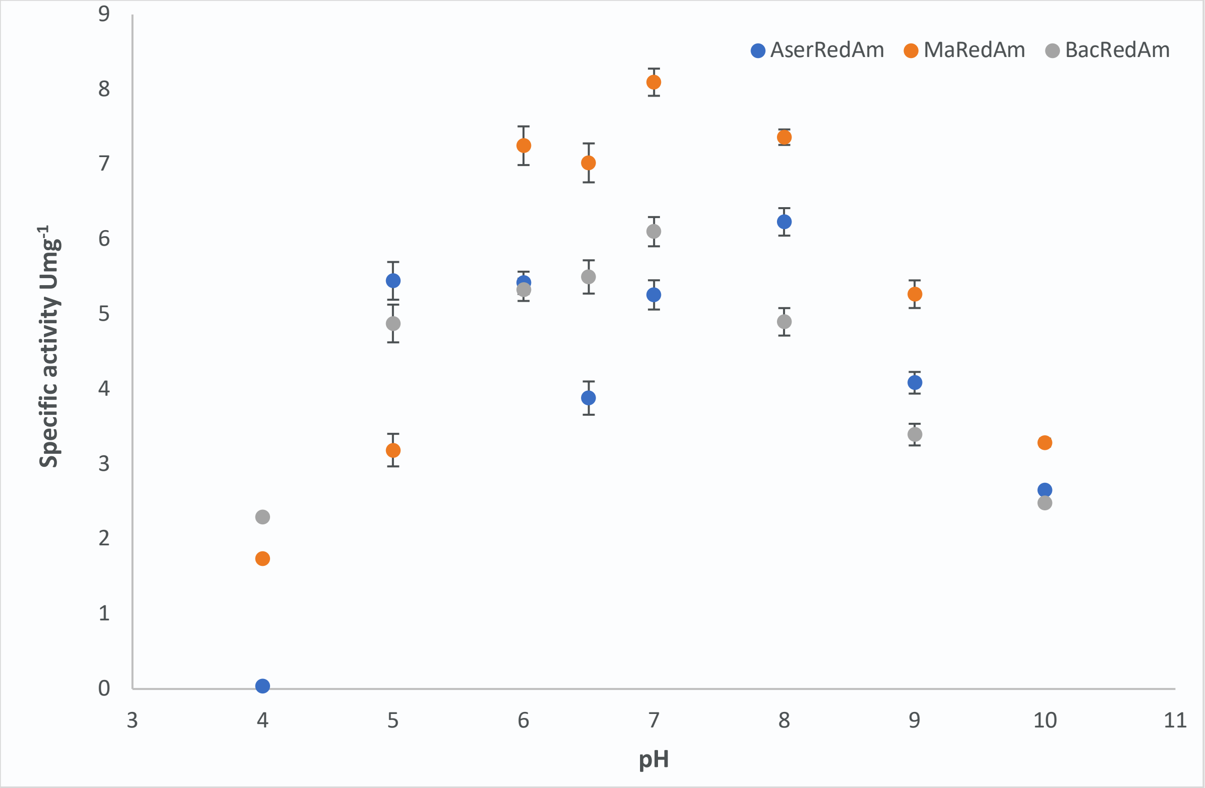

(b)

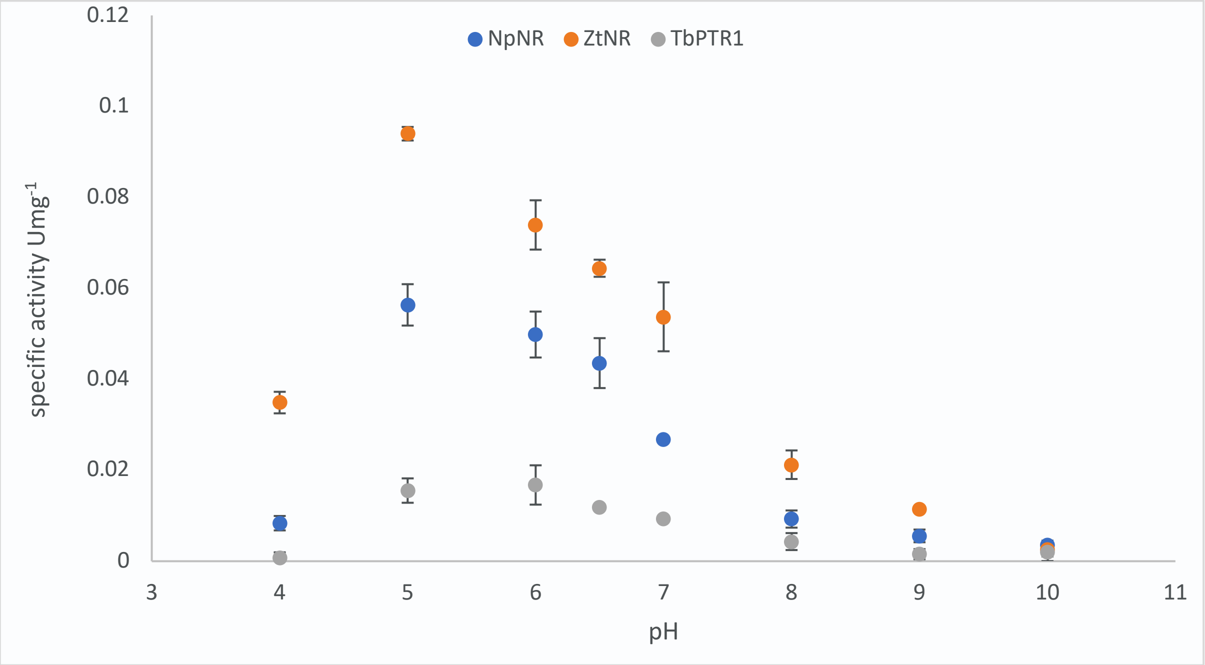

pH	Buffer	pH	Buffer
pH4-6	Citrate-Phosphate Buffer	pH 8-9	Tris-HCl
pH 6.5-7.5	Phosphate buffer	pH 10-11	Glycine-NaOH

Figure S6. pH profiles for the NADPH-dependent reduction of 1-methyl-3,4-dihydroisoquinoline imine for selected representatives of imine reducing enzymes.

Supplemental Representative chromatograms

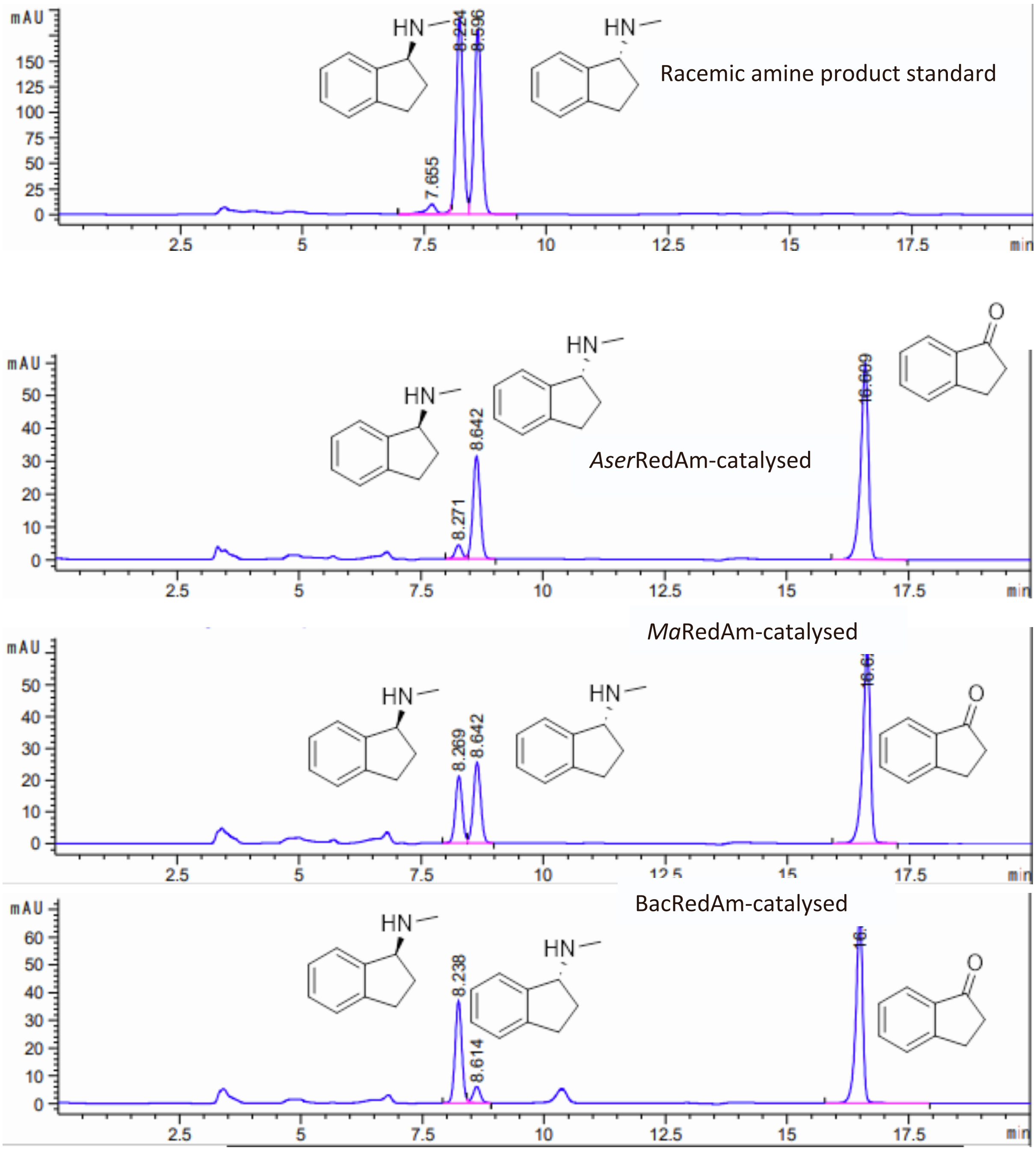

Figure S7. An example of products obtained from IRED-catalysed amination of aromatic ketones: Chiral HPLC analysis of IRED-catalysed reductive amination of 1-indanone **27** with methylamine **c** to afford enantioenriched alpha secondary amine product **27c**.

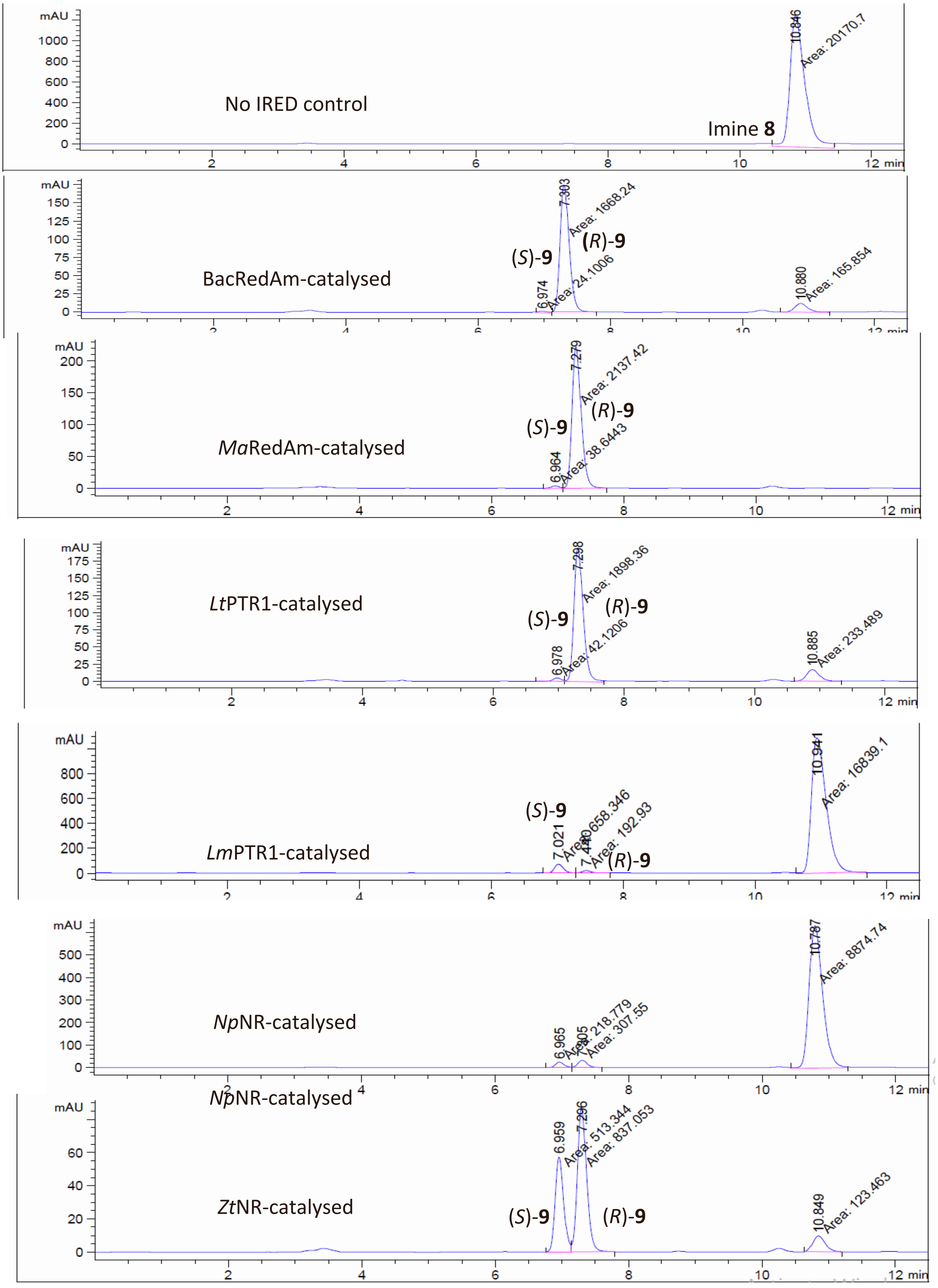

Figure S8. Chiral HPLC analysis of biotransformation reaction for the reduction of 1-methyl-3,4-dihydroisoquinoline imine **8** to yield.

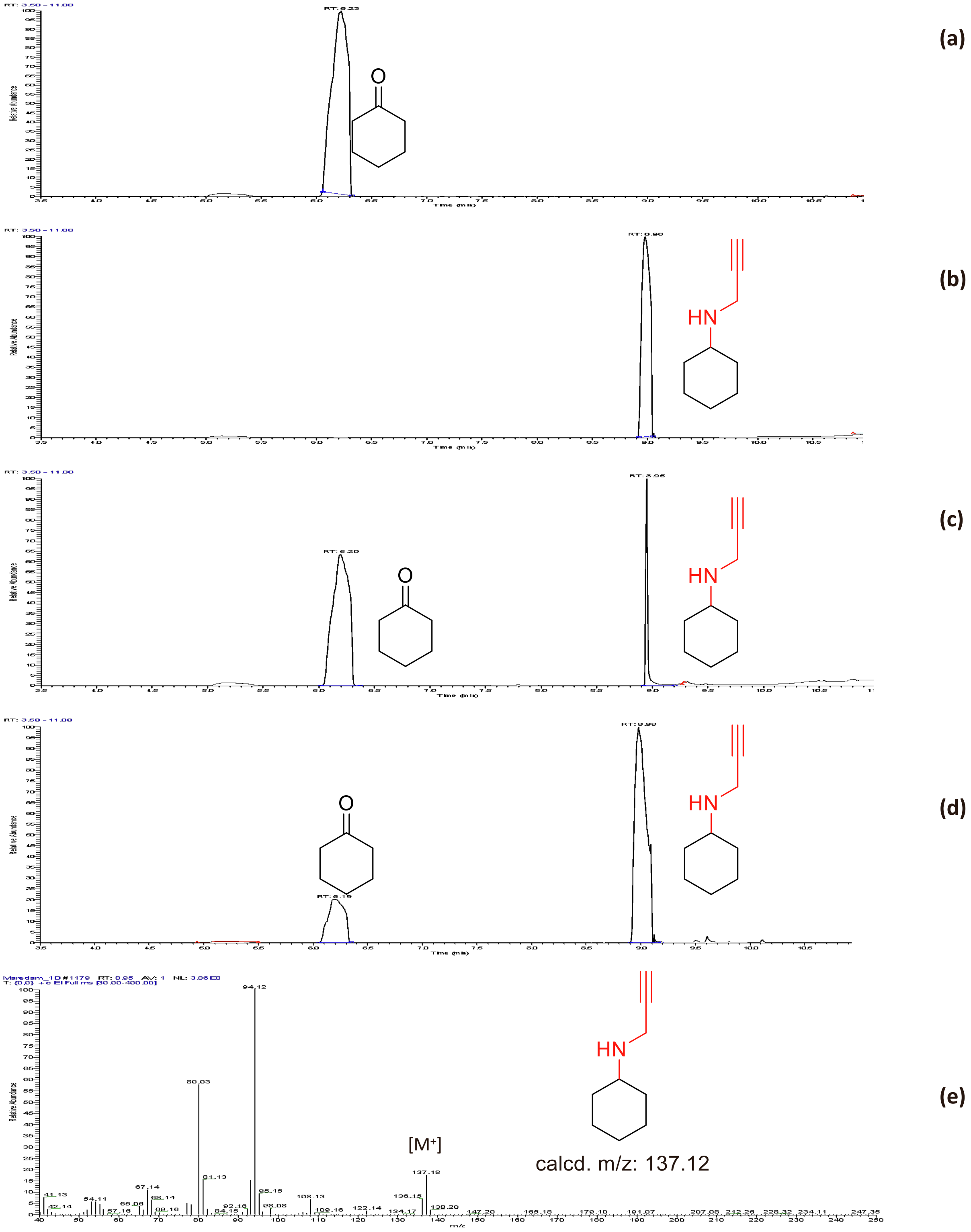

Figure S9. IRED-catalysed Reductive amination of cyclohexanone and propargylamine. (a) Control reaction containing all reaction components but lacking IRED. (b) Biotransformation catalysed by *Ma*RedAm. (c). Biotransformation catalysed by *Zt*NR (d). Biotransformation catalysed by *Lt*PTR1. (e) EI spectrum of the product obtained from MaRedAm-catalysed biotransformation. GCMS (EI) m/z = 137.18.

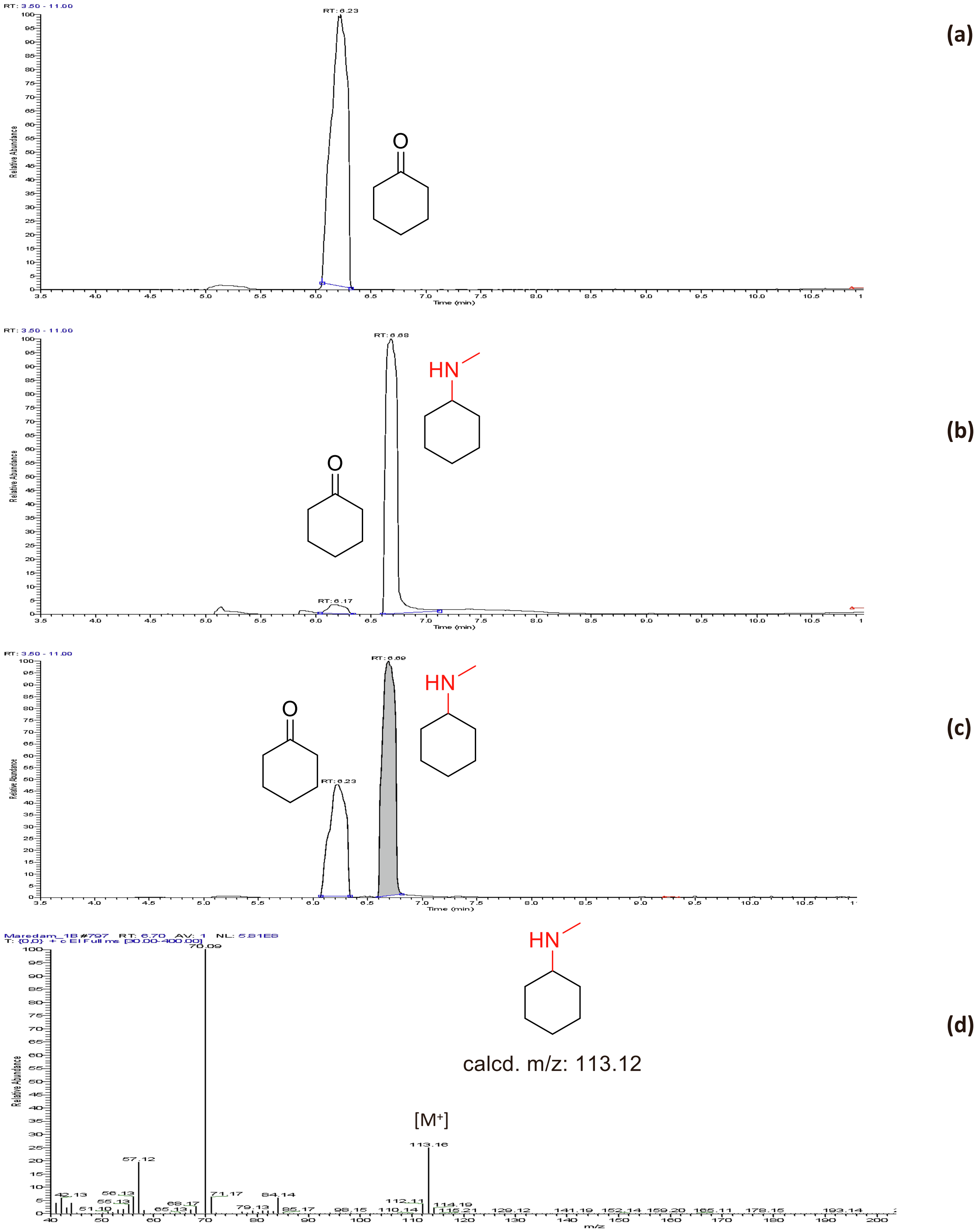

Figure S10. IRED-catalysed reductive amination of cyclohexanone and methylamine. (a) Control reaction containing all reaction components but lacking IRED. (b) Biotransformation catalysed by *Ma*RedAm. (c). Biotransformation catalysed by *Lt*PTR1, (d) EI spectrum of product obtained from MaRedAm-catalysed biotransformation. GCMS (EI) m/z found= 113.16.

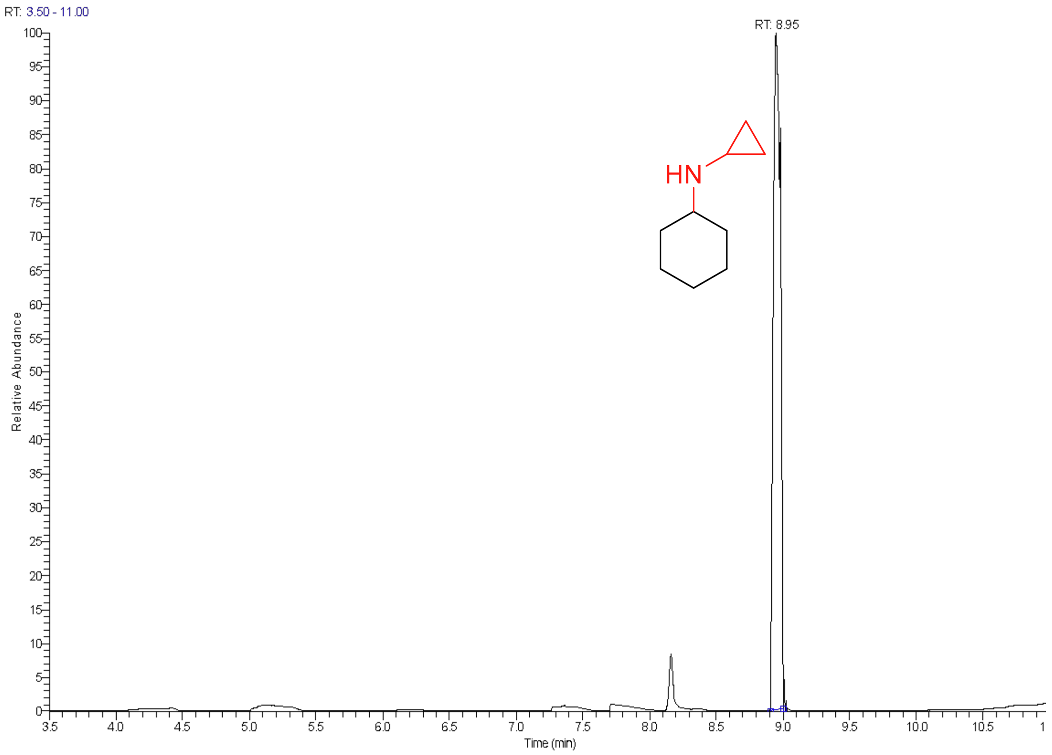

(a)

(b)

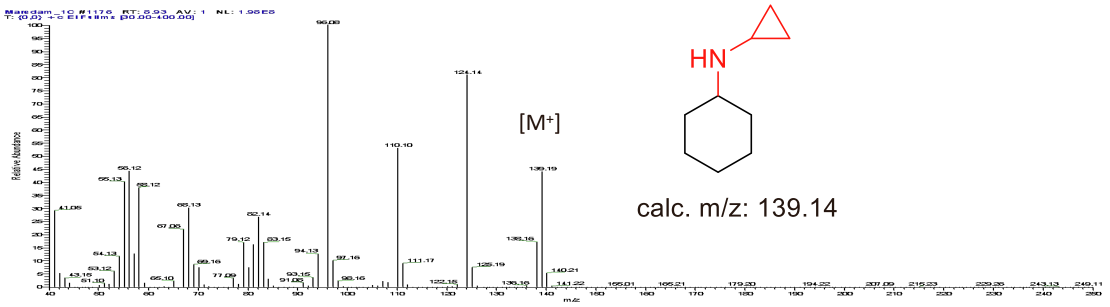

Figure S11. IRED-catalysed reductive amination of cyclohexanone and cyclopropylamine. (a) Biotransformation catalysed by *Ma*RedAm. (b) EI spectrum of the product obtained from MaRedAm-catalysed biotransformation. GCMS (EI) m/z found = 139.19.

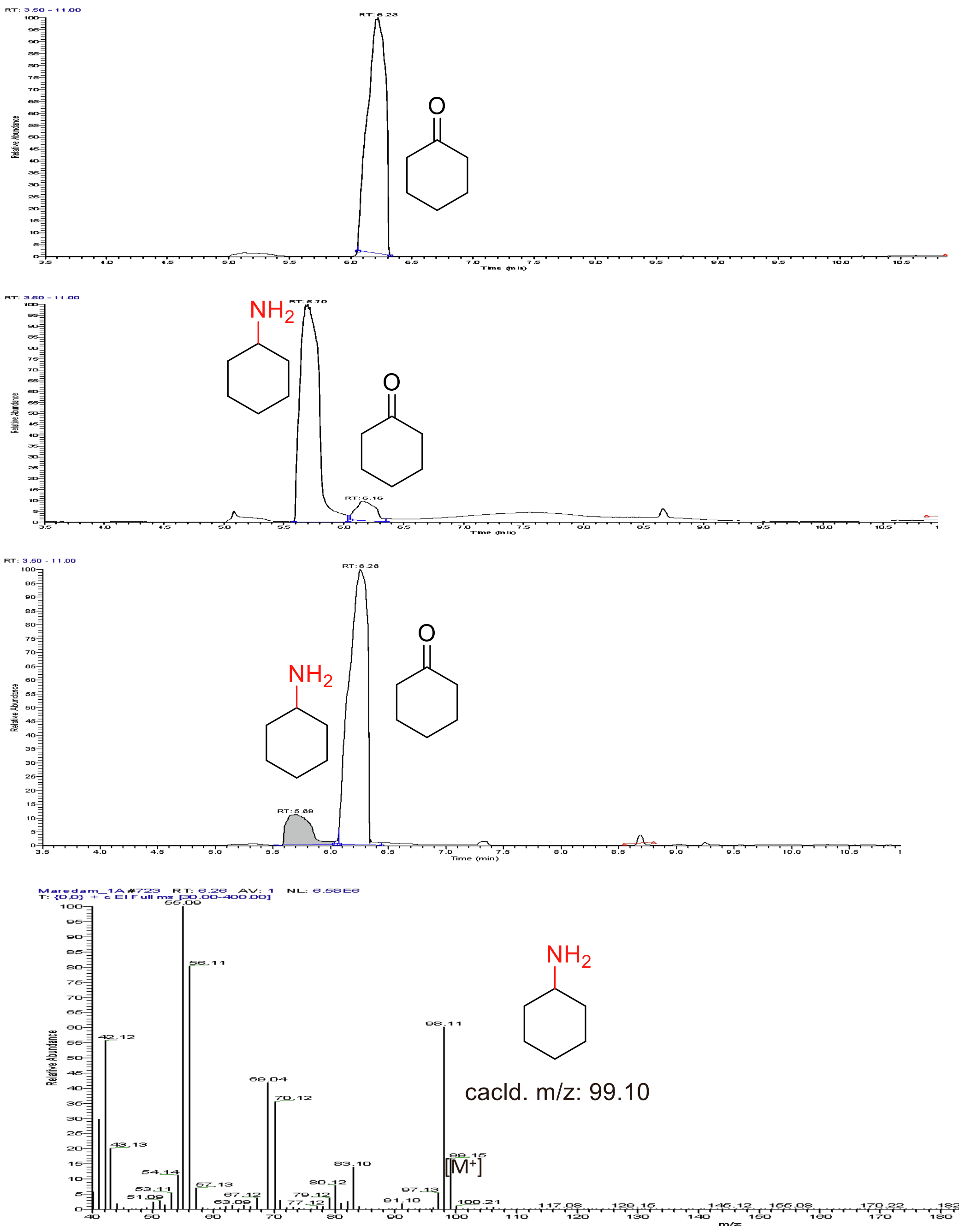

(a)

(b)

(c)

(d)

Figure S12. IRED-catalysed reductive amination of cyclohexanone and methylamine. (a) Control reaction containing all reaction components but lacking IRED. (b) Biotransformation catalysed by *Ma*RedAm. (c). Biotransformation catalysed by BacRedAm, (d) EI spectrum of product obtained from MaRedAm-catalysed biotransformation. GCMS (EI) m/z found= 99.15

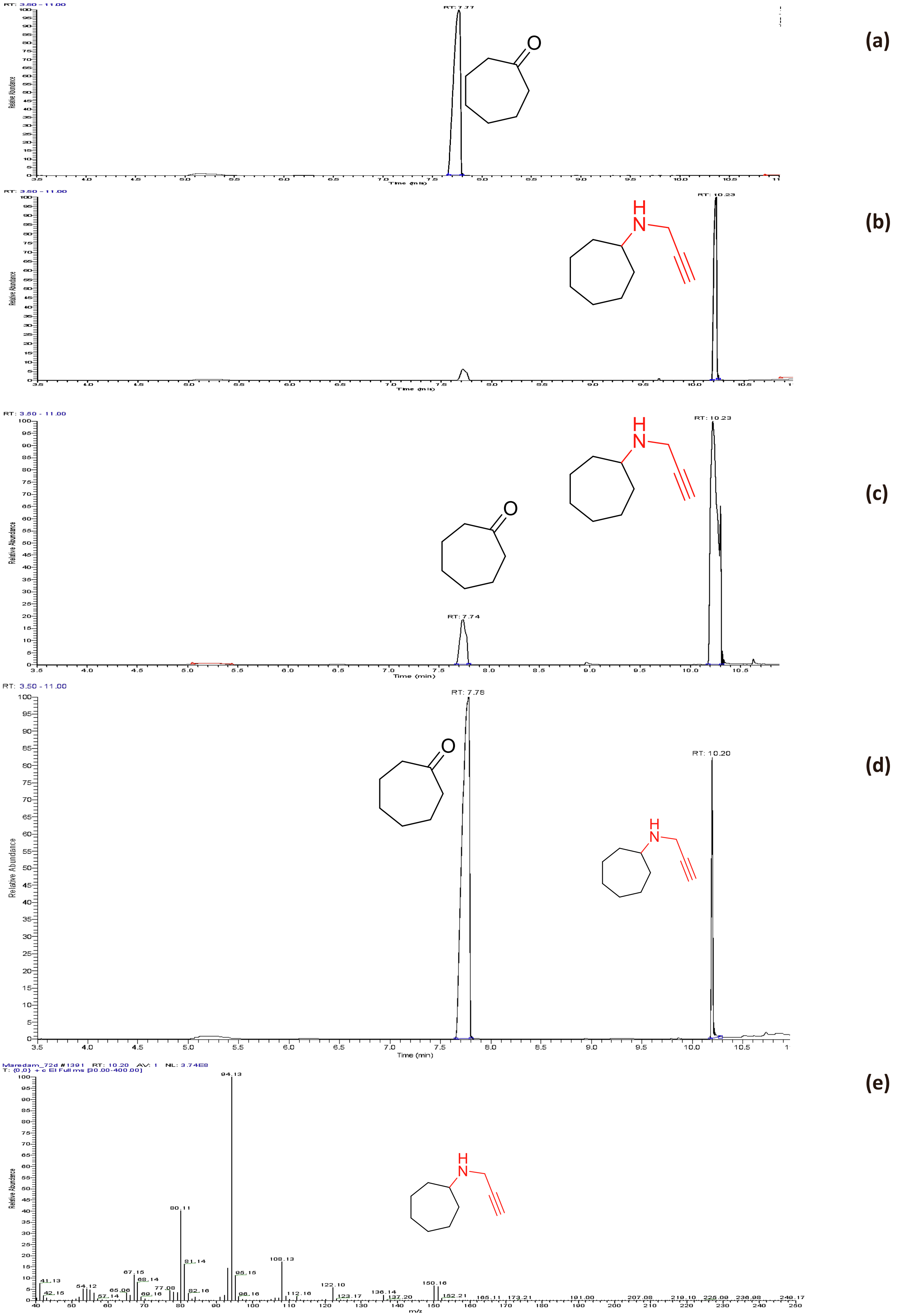

Figure S13. IRED-catalysed reductive amination of cycloheptanone and propargylamine. (a) Control reaction containing all reaction components but lacking IRED. (b) Biotransformation catalysed by

*Ma*RedAm. (c). Biotransformation catalysed by *Lt*PTR1 (d). Biotransformation catalysed by *Zt*NR. (e) EI spectrum of product obtained from MaRedAm-catalysed biotransformation.

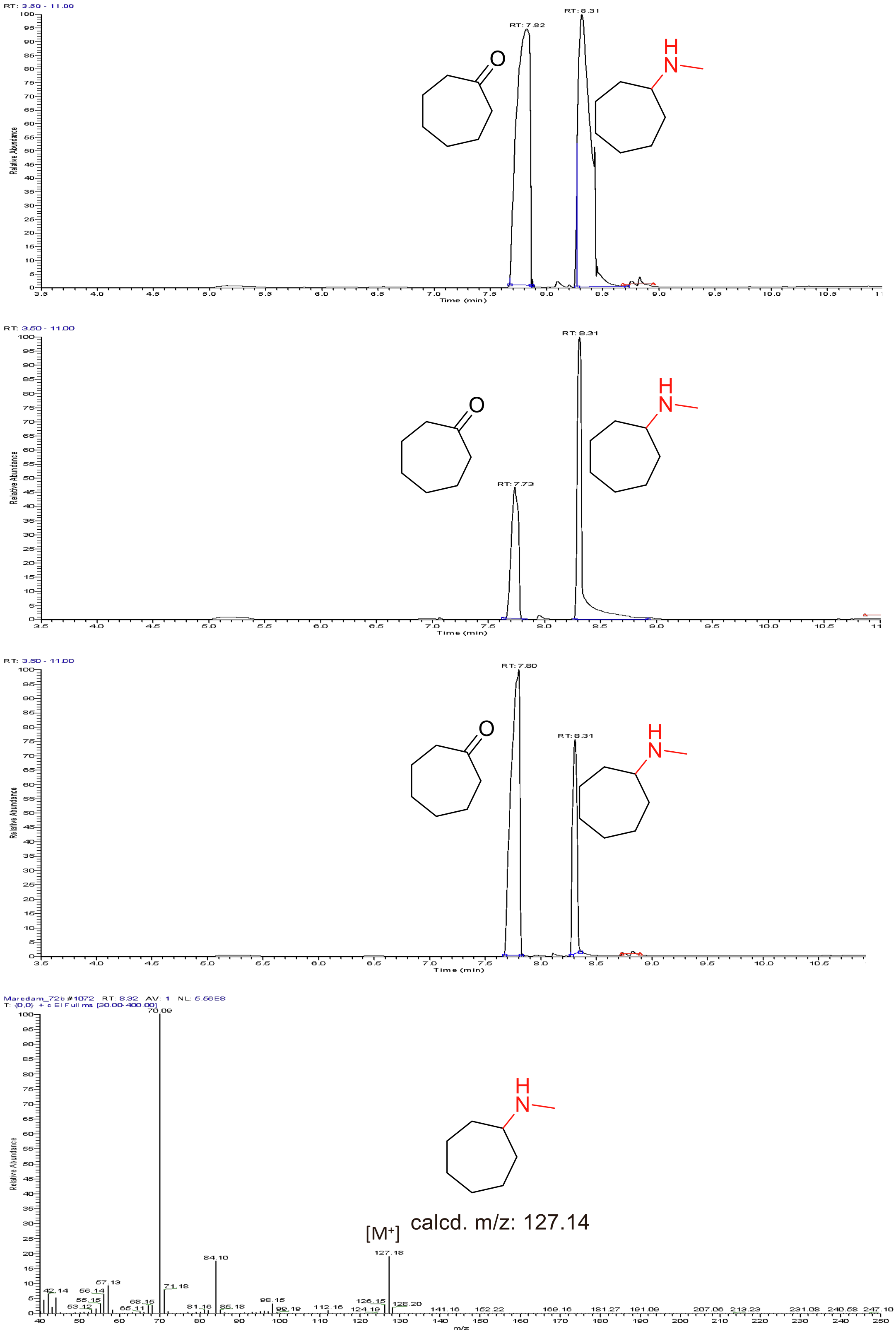

(a)

(b)

(c)

(d)

Figure S14. IRED-catalysed reductive amination of cycloheptanone and methylamine. (a) Biotransformation catalysed by *Ba*RedAm. (a) Biotransformation catalysed by *Ma*RedAm. (c).

Biotransformation catalysed by *Lt*PTR1, (d) EI spectrum of product obtained from MaRedAm-catalysed biotransformation. GCMS (EI) m/z found= 127.18

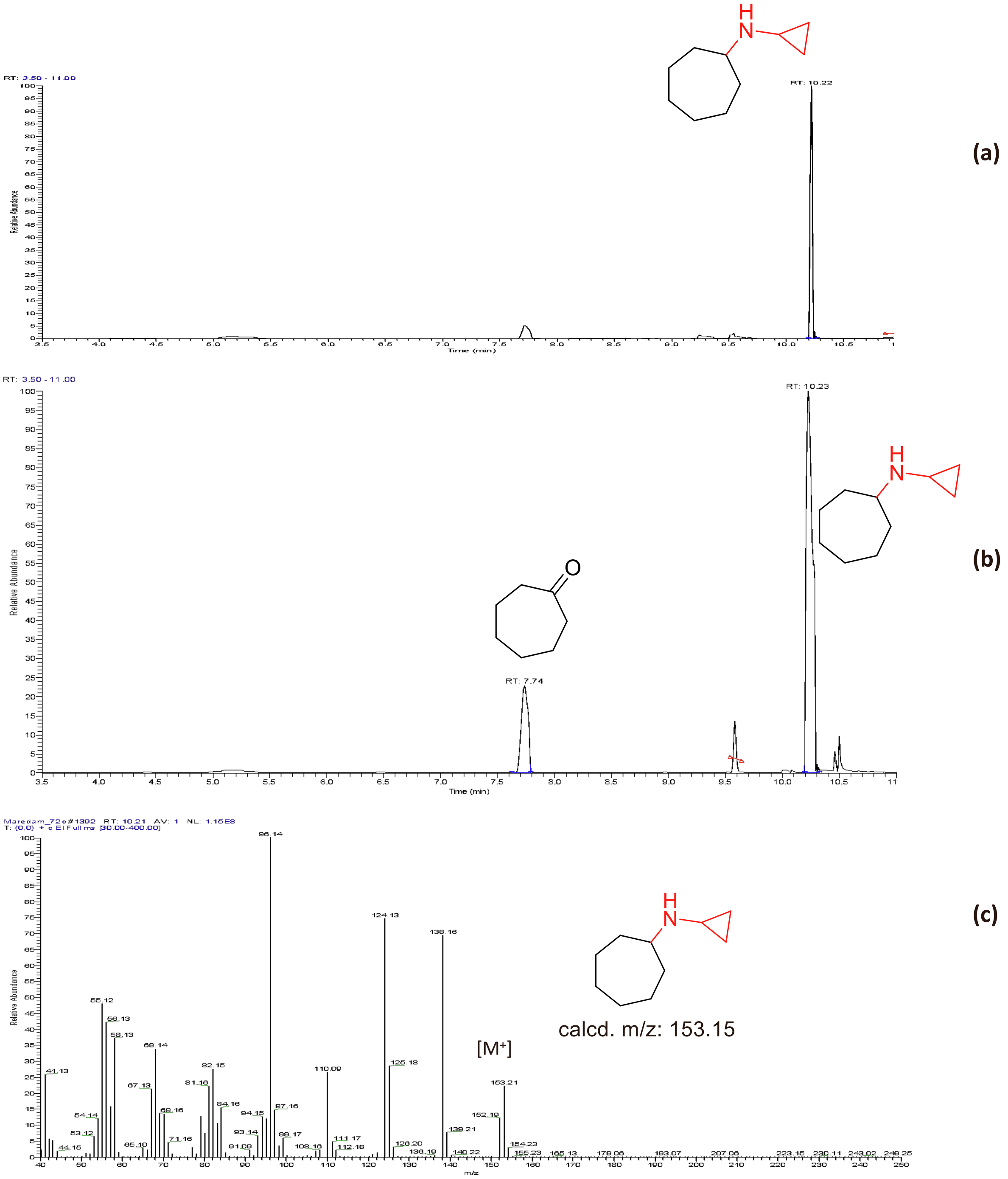

Figure S15. IRED-catalysed reductive amination of cycloheptanone and cyclopropylamine. (a) Biotransformation catalysed by *Ma*RedAm. (b). Biotransformation catalysed by *Lt*PTR1, (d) EI spectrum of product obtained from MaRedAm-catalysed biotransformation. GCMS (EI) m/z found= 153.2.

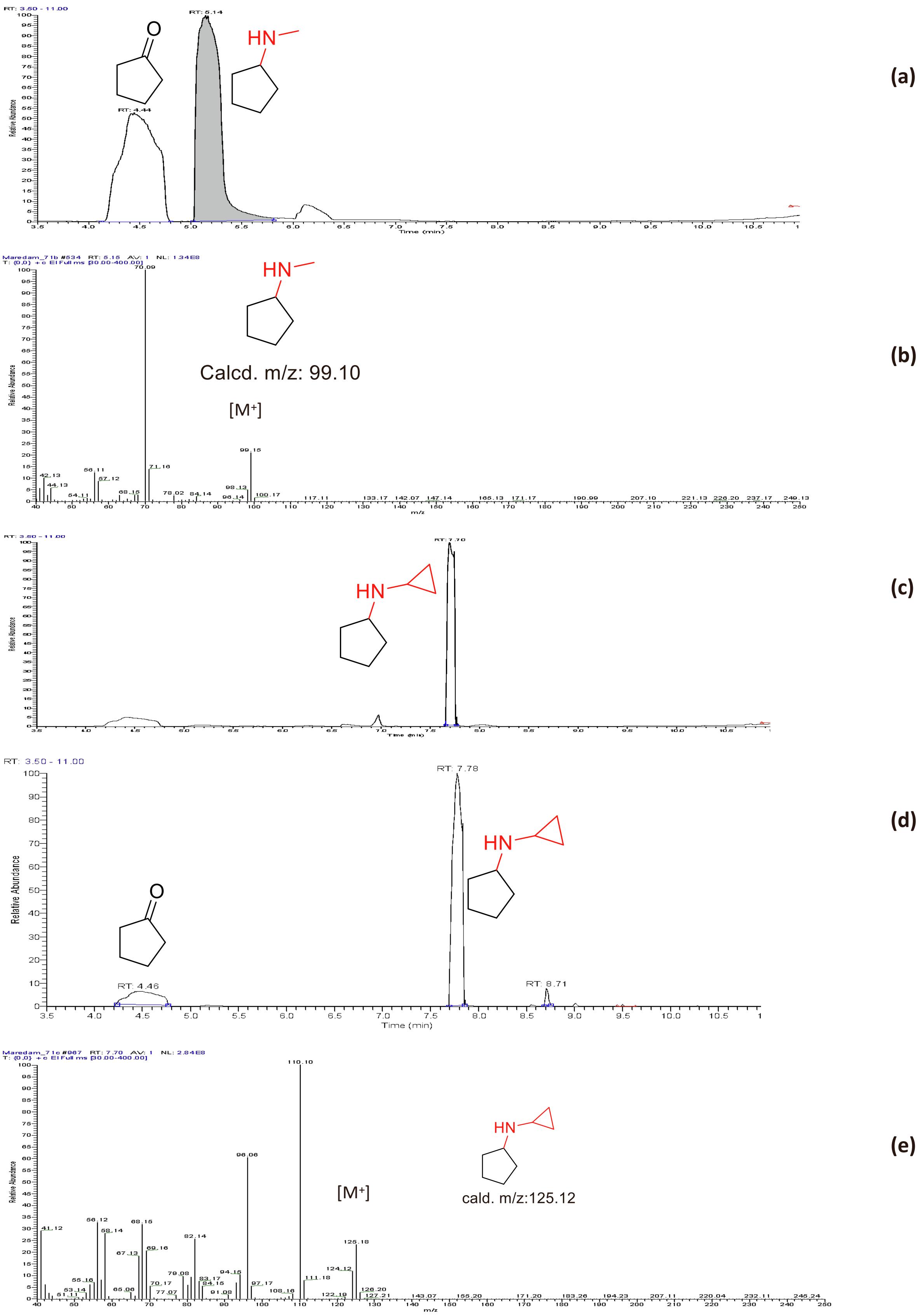

Figure S16. IRED-catalysed reductive amination of cyclopentanone. (a, b) Bioamination of cyclopentanone with methylamine catalysed by *Ma*RedAm and the associated EI spectrum of the biotransformation product, GCMS (EI) m/z found= 99.15. (c-e), Bioamination of cyclopentanone with propargylamine catalysed *Ma*RedAm (row c) and LtPTR1 (row d) and the associated EI spectrum of

the biotransformation product obtained from *Ma*RedAm-catalysed reaction (row e). GCMS (EI) m/z found= 125.18.

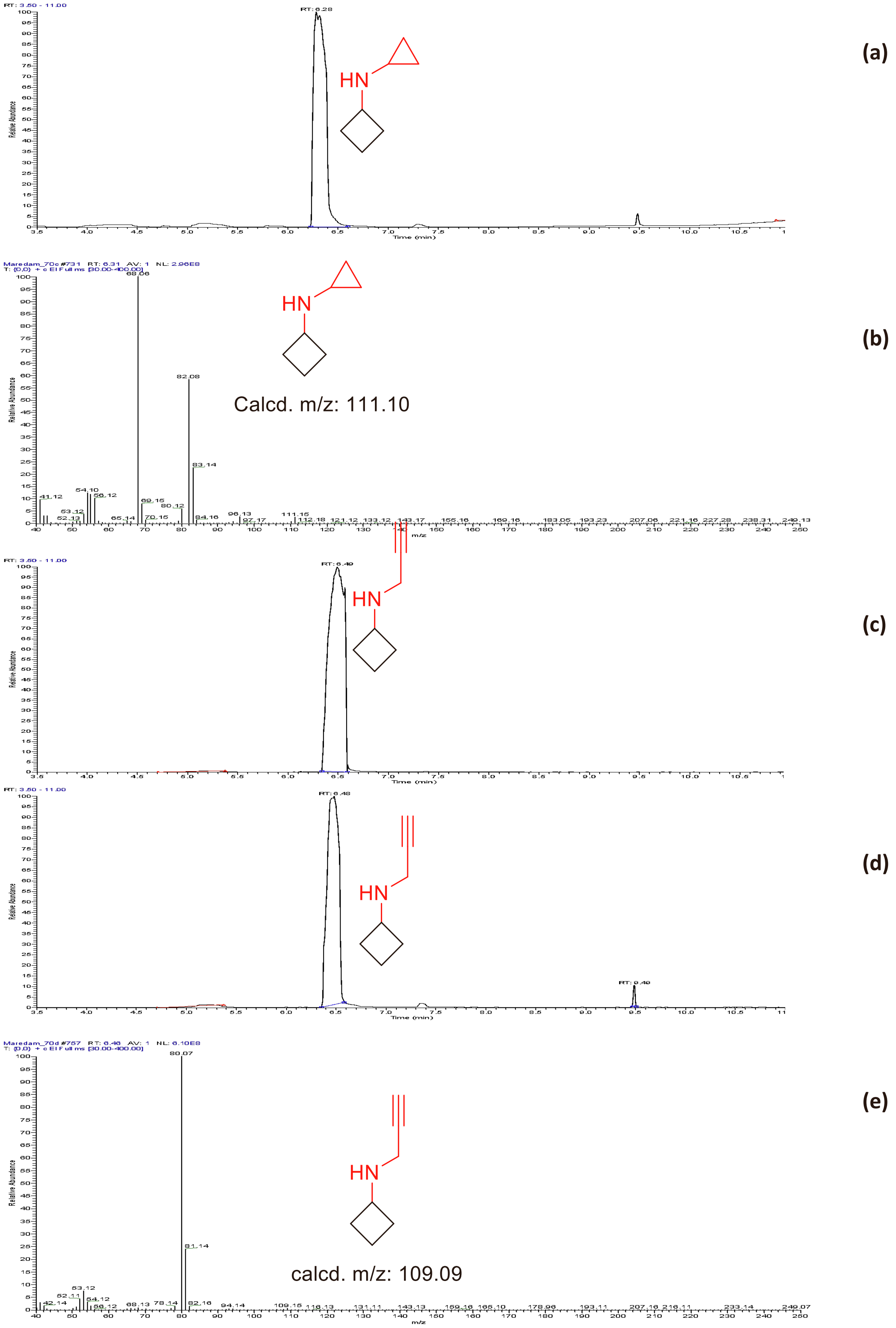

Figure S17. IRED-catalysed reductive amination of cyclobutanone. (a, b) Bioamination of cyclobutanone with cyclopropylamine catalysed by *Ma*RedAm and (b) the associated EI spectrum of the biotransformation product, GCMS (EI) m/z found= 111.15. (c-e), Bioamination of cyclobutanone

with propargylamine catalysed *Ma*RedAm (row c) and LtPTR1 (row d) and the associated EI spectrum of the biotransformation product obtained from *Ma*RedAm-catalysed reaction (row e). GCMS (EI) m/z found= 109.15

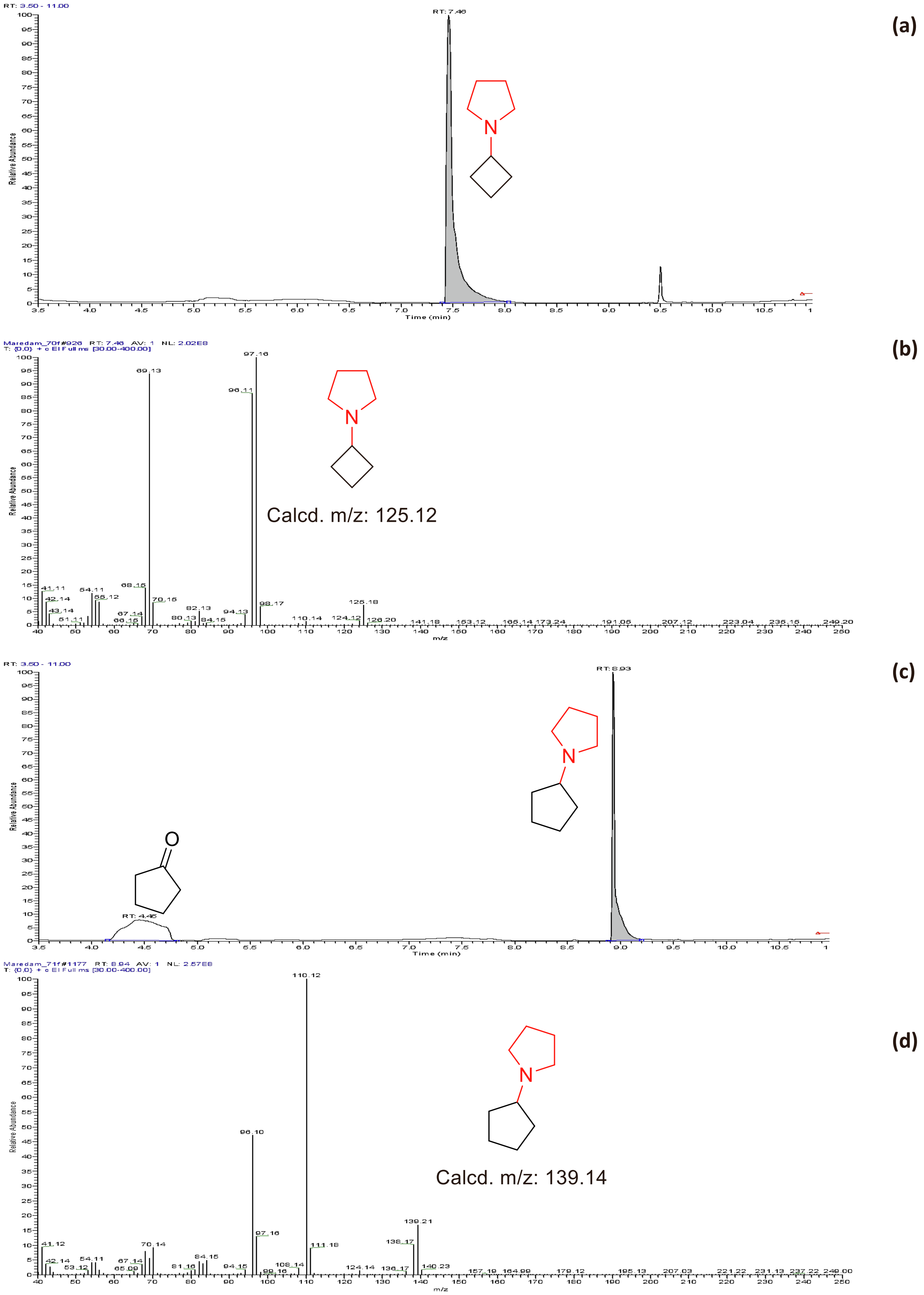

Figure S18. IRED-catalysed reductive amination of ketones with pyrrolidine. (a) Bioamination of cyclobutanone with pyrrolidine catalysed by *Ma*RedAm and (b) the associated EI spectrum of the

biotransformation product, GCMS (EI) m/z found= 125.18. (c-e), Bioamination of cyclopentanone with propargylamine catalysed *Ma*RedAm (row c) and the associated EI spectrum of the biotransformation product obtained from MaRedam-catalysed reaction. GCMS (EI) m/z found= 139.21.

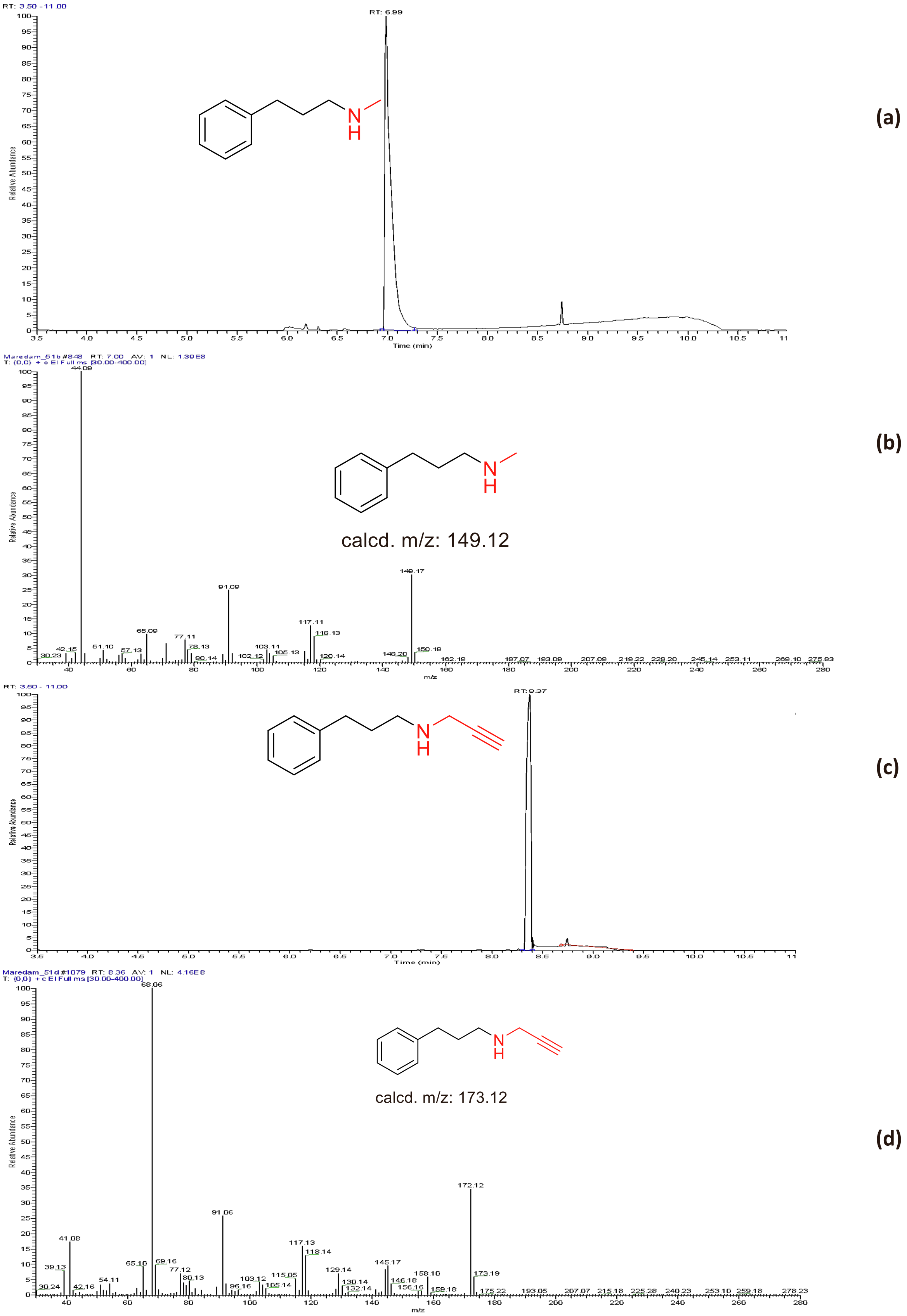

Figure S19. IRED-catalysed reductive amination of hydrocinnamaldehyde. (a) Bioamination of

hydrocinnamaldehyde with methylamine catalysed by *Ma*RedAm and (b) the associated EI spectrum of the biotransformation product, GCMS (EI) m/z found= 149.17. (c) Bioamination of hydrocinnamaldehyde with propargylamine catalysed by *Ma*RedAm and (b) the associated EI spectrum of the biotransformation product, GCMS (EI) m/z found= 173.19.

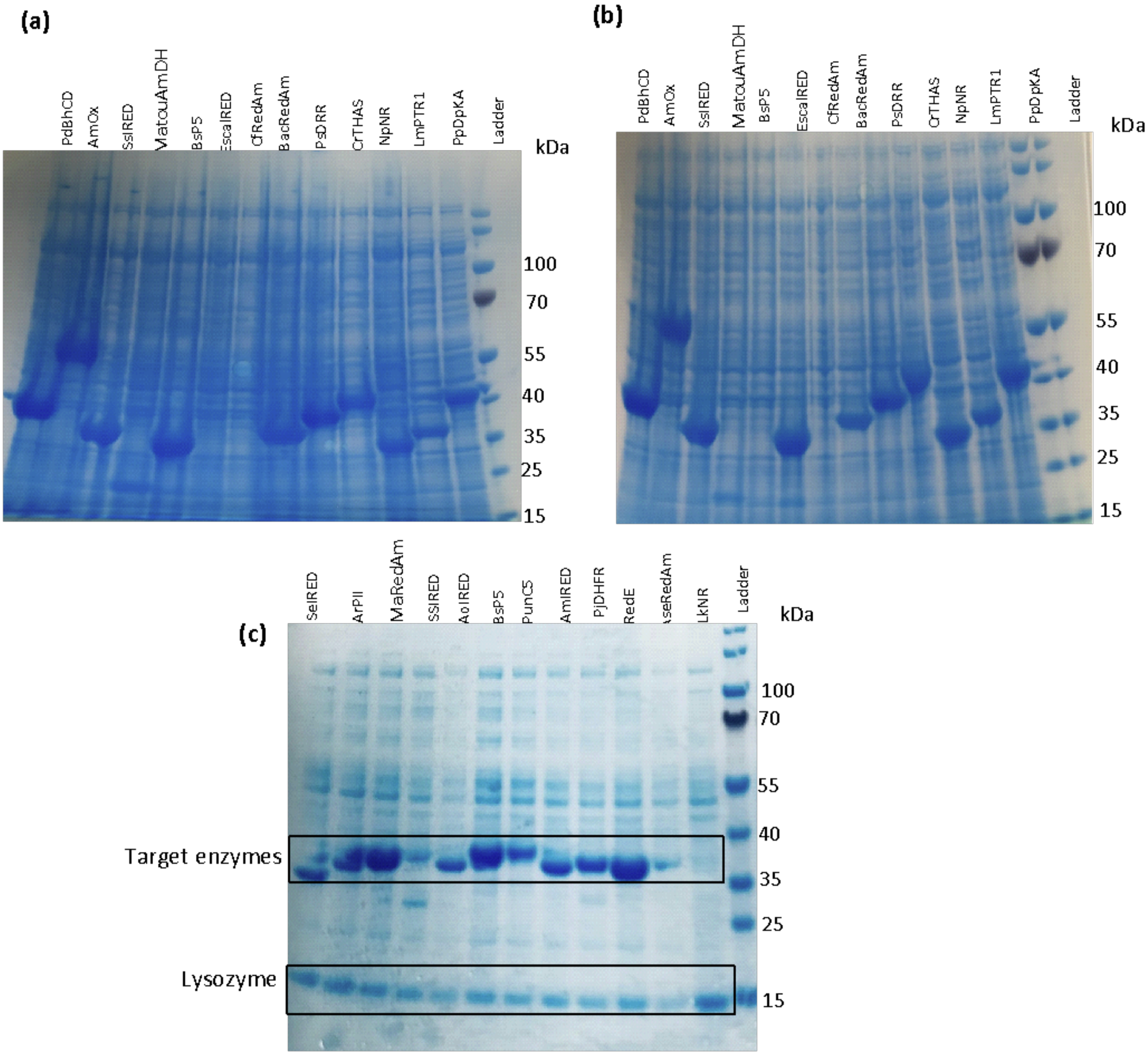

Figure S20. SDS page gels to detect soluble expression by analysis of clarified lysates obtained from recombinant *E. coli* expression. Expression was trialled with **(a)** Lysogeny broth (LB) autoinduction media (initial cultivation for 5 h at 37 °C then continued for 40 h at 24 °C) *versus* **(b,c)** LB media with IPTG induction (initial cultivation at 37 °C until OD_{600} of between 0.6 and 0.8, then induction with IPTG (0.4 mM), cultivation continued at 22°C). For gels (a) and (b), cell disruption was performed using ultrasonication. For gel (c), lysis was performed with lysozyme-based lysis buffer (100 mM Tris-HCl, 100 mM NaCl, pH 7.5, 1 mg ml^{-1} lysozyme, 0.3 mg ml^{-1} polymyxin B), incubated at 20 °C for 1h 30 min. Both IPTG induction and autoinduction conditions were suitable for the expression of most of the investigated enzymes. Similarly, cell disruption with either ultrasonication or lysozyme-based cell lysis was both effective. The enzymatic lysis was employed for small-volume cultures, while ultrasonication was used for larger-volume cultures.

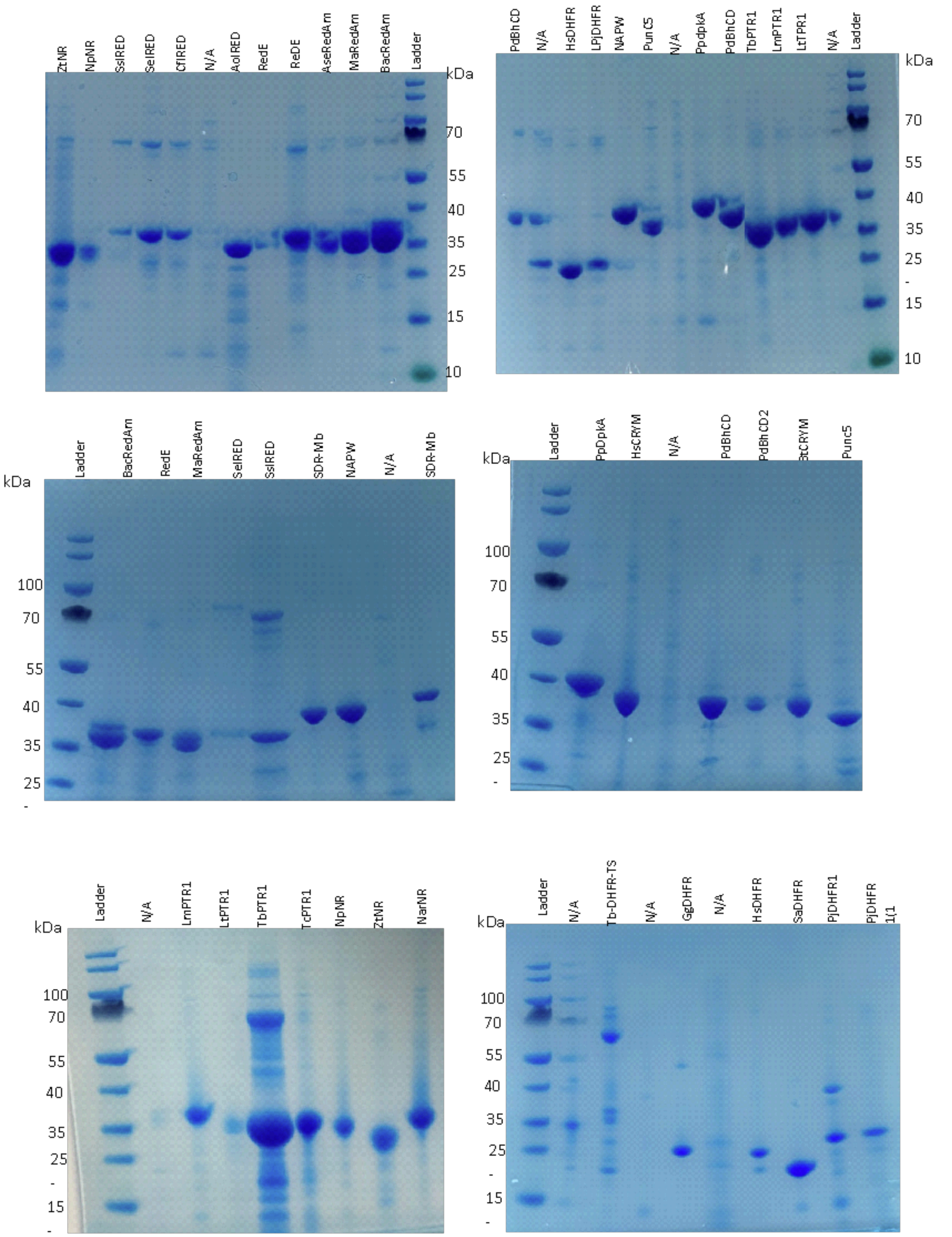

Figure S21. Protein gel (SDS page) analysis of semi-purified IREDs. For quick (semi)-purification, cells were resuspended in buffer A (50 mM Tris-HCl buffer, 300 mM NaCl, 25 mM imidazole pH 7.5) and were disrupted by ultrasonication at 4°C employing 5 × 20 s bursts with 40 s intervals at 4 °C. Following centrifugation of the lysed cells, the clarified lysate was purified by Ni-affinity chromatography using gravity columns. Clarified lysate was loaded onto the column, followed by a single-step wash with buffer A, and eluted with buffer B (50 mM Tris-HCl buffer, 300 mM NaCl, 300 mM imidazole, pH 7.5). The elute was desalted using 10 kDa cut-off Centricon filters (15 ml) and the buffer was exchanged to Tris-HCL buffer (50 mM Tris, 100 mM NaCl, pH 7.5) or HEPES (30 mM, 100 ml NaCl).

Table S1. A panel of highly divergent/non-homologous (biosynthetic) imine-reducing enzymes investigated in this study

Entry		Organismal source	*Physiological function (Pathway)*	*Genbank ID*
Classical IREDs involved in biosynthesis and homologues				
1	RedE	*Metagenomic*	tryptophan dimer (TD) biosynthesis	>AKG47111.1
2	ArpDHII	*Streptomyces argillaceus*	Argimycins P dehydrogenase	SCO70303.1
3	*Bac*RedAm	Metagenomic (bacterial)	Not known	PZN88780.1
4	*Mared*Am	*Mortierella antarctica*	Not known	KAF9989289.1
5	*Aser*RedAm	*Aspergillus sergii*	Not known	KAE8332418.1
6	*Pt*IRED	*Pyxidicoccus trucidator*	Not known	WP_164011897.1
7	*Ss*IRED	*Streptomyces sp. WAC 05977*	Not known	RSN12190.1
8	*Se*IRED	*Saccharopolyspora erythraea*	Not known	WP_211898848.1
9	*ArIRED*	*Amycolatopsis roodepoortensis*	Not known	WP_257488618.1
Pteridine reductases (PTR1s)				
10	*Lm*PTR1	*Leishmania major*	Protozoan Pteridine reductase (pterins salvage)	sp|Q01782.2
11	*Lt*PTR1	*Leishmania tarentolae*	Protozoan Pteridine reductase (pterins salvage)	sp|P42556.1
12	*Tc*PTR1	*Trypanosoma cruzi*	Protozoan Pteridine reductase (pterins salvage)	AAC38850.1
13	*Tb*PTR1	*Trypanosoma brucei equiperdum]*	Protozoan Pteridine reductase (pterins salvage)	RHW70915.1
14	*Am*PTR1	*Alteromonas mediterranea*	PTR1-like protein	WP_071968609.1
15	*At*PruA	*Agrobacterium tumefaciens*	Bacterial pteridine reductase	WP_006312872.1
16	*Pa*PruA	*Pseudomonas aeruginosa*	Bacterial pteridine reductase	WP_003091895.1
Dihydrofolate reductases (DHFRs)				
17	*Pj*DHFR/PcDHFR	*Pneumocystis jirovecii*	Dihydrofolate reductase (folate metabolism)	1VJ3_A
18	*Gg*DHFR	*Gallus gallus*	Dihydrofolate reductase (folate metabolism)	NP_001006584.2
19	*h*DHFR	*Homo sapien*	Dihydrofolate reductase (folate metabolism)	NP_000782.1
20	*Tb*DHFR-TS	*Trypanosoma brucei*	Bifunctional dihydrofolate reductase-thymidylate synthase	Q27783.1
22	*Tb*DHFR	*Trypanosoma brucei*	Dihydrofolate reductase	3QFX_A
23	*PvDHfR*	*Plasmodium vivax*	Dihydrofolate reductase	WAN11905.1

24	*PfDHFR*	*Plasmodium falciparum*	Dihydrofolate reductase	PDB: 1J3I_A
Short-chain dehydrogenases/reductases *(SDRs) involved in plant alkaloid biosynthesis*				
25	*Np*NR	*Narcissus pseudonarcissus*	noroxomaritidine/norcraugsodine reductase (NR)	AUG71944.1
26	*NpaNR*	*Narcissus papyraceus*	noroxomaritidine/norcraugsodine reductase	AXU39908.1
27	*Zt*NR	*Zephyranthes treatiae*	NR-like plant protein	6Y4D_A
28	LrNR	*Lycoris radiata*	NR-like plant protein	QFQ50502.1
29	LsNR	*Larkinella sp. BK230*	NR-like bacterial protein	WP_208327519.1
30	*Cr*THAS	*Catharanthus roseus*	tetrahydroalstonine synthase	AKF02528.1
31	*EcSaRed*	*Eschscholzia californica*	Sanguinarine reductase (benzophenanthridine detoxification)	>sp|D5JWB3.1
32	*Ps*DRR	*Papaver Sp*	1,2-dehydroreticuline reductase (morphine biosynthesiss)	ACM44068.1
33	*Cp*EaSG	*Claviceps gigantea*	agroclavine synthase (ergot alkaloid biosynthesis)	ATW01297.1
SDRs in bacterial alkaloids biosynthesis				
34	NAPW	*Streptomyces sp.*	naphthyridinomycin biosynthetic	WP_121719702.1
35	*Mb*SDR	*Myxococcales bacterium*	NAPW-like protein	OJY30843.1
36	*Pb*SDR	*Paenibacillus sp.*	NAPW-like protein	WP_076164526.1
(A)cyclic imino-acid reductases				
37	PunC5	*Paenibacillus sp.*	Imino acid reductase	WP_090636782.1
38	*BsP5*	*Bacillus sp.*	Imine acid reductase	pdb|6P2I|A
39	*Pd*BhcD	*Paracoccus denitrificans*	Imminosuccinate reductase (β-hydroxyaspartate cycle)	pdb|6RQA|A
40	*Pp*DpKA	Pseudomonas putida	Amino acid biosynthesis	Q5FB93.1
41	*Hs*CRYM	*Human sapiens*	ketimine reductase mu-crystallin	NP_001363185.1
42	*Bt*DHFR	*Bos taurus*	ketimine reductase mu-crystallin	Leucojum aestivum
43	*Cp*IM1	*Candida parapsilosis*	Ketimine reductase-like protein	XP_036665050.1
44	PchG	*Pseudomonas aeruginosa*	pyochelin biosynthesis thiazoline reductase	: WP_250024055.1

Table S2. Biotransformation for the imine reduction of 1-methyl-3,4-dihydroisoquinoline **8** using stoichiometric amounts of NAD(P)H.

Enzyme	Cofactor	Conversion (%)	e.e. (%)	Absolute conf.
*Am*IRED	NADPH	98	71	(S)
	NADH	>99	79	(S)
*Bac*Redam	NADPH	>98	94	(*R*)
	NADH	93	89	(*R*)
MaRedAm	NADPH	98	86	(*R*)
	NADH	>99	90	(*R*)
*Lt*PTR1	NADPH	>99	92	(*R*)
	NADH	>99	92	(*R*)
*Lm*PTR1	NADPH	>99	49	(S)
	NADH	59	70	(S)
*Tb*PTR1	NADPH	79	1	(S)
	NADH	45	11	(S)
*Np*NR	NADPH	>99	15	(*R*)
	NADH	91	19	(*R*)
*Np*NR	NADPH	>99	19	(*R*)
	NADH	>99	20	(*R*)

Reaction condition: 5 mM imine, 10 mM NAD(P)H, 0.25-1 mg ml^{-1} IRED in 0.5 ml phosphate buffer (100 mM with 100 mM NaCl, pH 7.0). The reaction was incubated at 25 °C for 24 h.

Table S3. HPLC methods and retention times

Method A: *n*-hexane/isopropanol/diethylamine (90/10/0.1).
Method B: *n*-hexane/isopropanol/diethylamine (80/20/0.1).
Method C: *n*-hexane/isopropanol/diethylamine (98/02/0.1).
Method D: *n*-hexane/isopropanol/diethylamine (97/03/0.1).
Flow rate: 1 ml min^{-1}, monitored at wavelength of 265 nm.
Columns: CHIRALPAK®IC 250 mm × 4.6 mm, 5 μm; and CHIRALPAK® IB N-5, 250 mm × 4.6 mm, 5 μm.

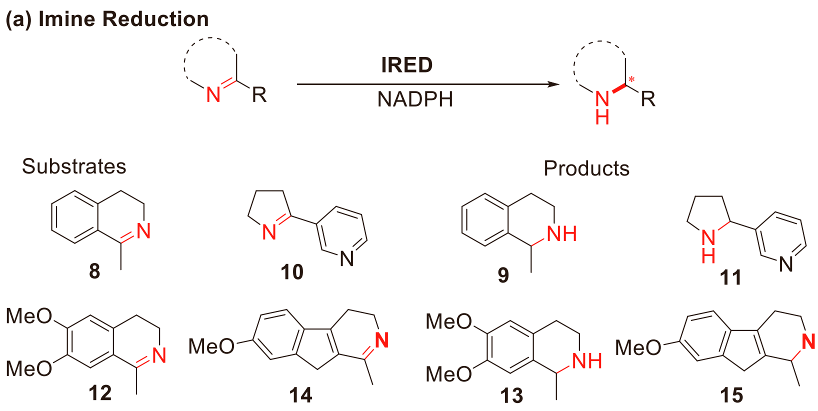

Imine Substrate	Amine product	Column	n-hexane/IPA/DEA	Retention time		
				Imine	Amine product	
					T1	T2
8	9	CHIRALPAK®IC	Method A	11.0	7.0 (*S*)	7.4 (*R*)

**10**	**11**	CHIRALPAK®IB N-5	Method A	12.7	16.2 (*R*)	19.4 (S)
**12**	**13**	CHIRALPAK®IB N-5	Method A	12.4	16.2 (*R*)	19.4 (*S*)
**14**	**15**	CHIRALPAK®IC	Method B	6.7	12.0 (S)	14.6 (*R*)

(b) Reductive Amination

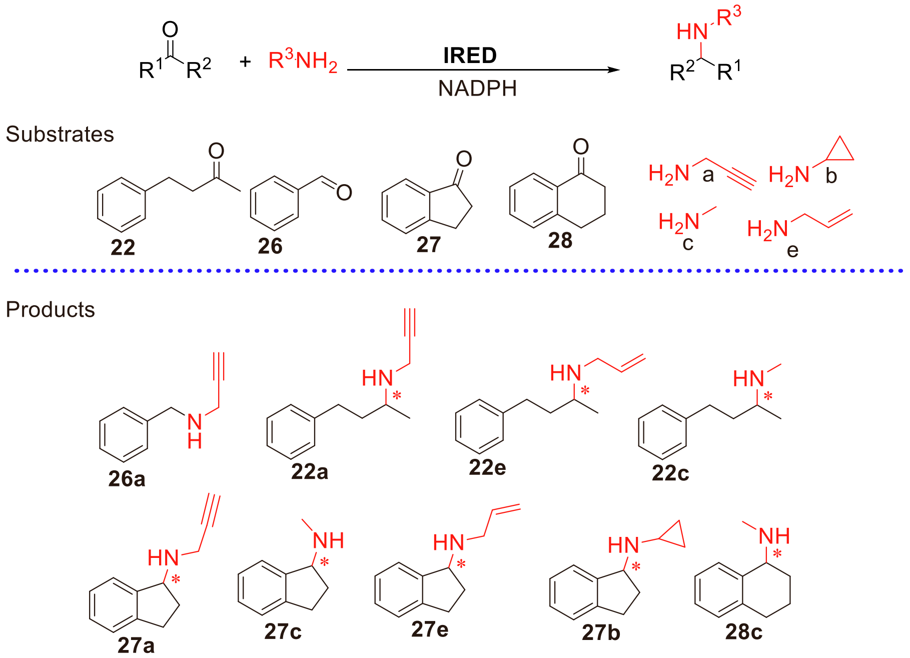

Substrates		Amine product	Column	n-hexane/IPA/DEA	Retention time		
					Ketone/aldehyde	Amine product	
Carbonyl	Amine					T1	T2
**22**	**c**	**22c**	CHIRALPAK®IB N-5	Method C	6.3	5.4 (*R*)	5.9 (*S*)
**22**	**b**	**22b**	CHIRALPAK®IB N-5	Method C	6.3	3.9 [a]	
**22**	**e**	**22e**	CHIRALPAK®IB N-5	Method C	6.3	4.2 (*R*)	4.4 (*S*)
**22**	**a**	**22a**	CHIRALPAK®IB N-5	Method C	6.3	5.2 (*R*)	5.4 (*S*)
**26**	**a**	**26a**	CHIRALPAK®IC	Method C	7.8	6.3[b]	
**27**	**c**	**27c**	CHIRALPAK®IC	Method C	21.4	8.2 (*S*)	8.6 (*R*)
**27**	**c**	**27c**	CHIRALPAK®IC	Method D	17	8.2 (*S*)	8.6 (*R*)
**27**	**b**	**27b**	CHIRALPAK®IC	Method C	21.4	4.2[a]	
**27**	**e**	**27e**	CHIRALPAK®IC	Method C	21.4	4.7 *S*)	5.0 (*R*)
**27**	**a**	**27a**	CHIRALPAK®IC	Method C	21.4	6.1 *S*)	6.7 (*R*)
**28**	**c**	**28c**	CHIRALPAK®IC	Method C	14.0	6.5 (*S*)	7.2 (*R*)

[a] HPLC method did not resolve enantiomers. [b] product is non-chiral.

Table S4. GC-MS analysis: retention times for ketones/aldehydes and reductive amination products

Column: CD-5MS Capillary Column 30 m x 0.25 mm x 0.25µm (P/N MOD-GC-CD5MSU-5, S/N CD17506. Method: inlet temperature = 250°C, detector temperature = 250°C, MS source= 230 °C, gas flow = 1.0 ml min^{-1}; oven temperature between 60 - 280°C, 20°C min^{-1} or 15 °C min^{-1}

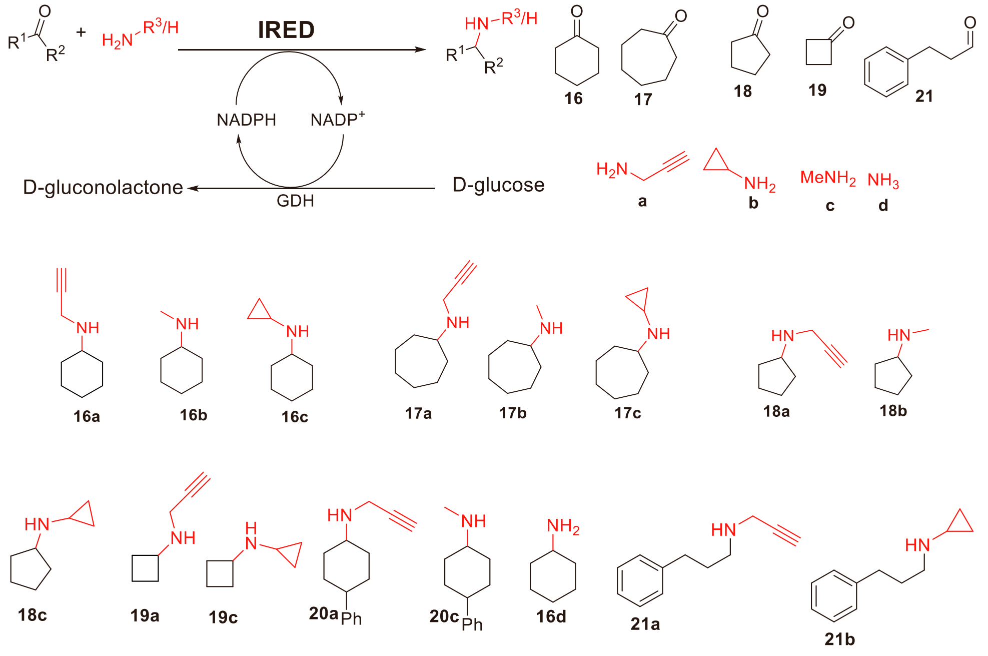

Ketone	Amine donor	Amine product	Ketone retention time [min]	Amine retention time [min]
**16**	**a**	**16a**	6.2	9.0
**16**	**b**	**16b**	6.2	8.9
**16**	**c**	**16c**	6.2	6.7
**16**	**d**	**16d**	6.2	5.7
**17**	**a**	**17a**	7.7	10.2
**17**	**b**	**17b**	7.7	10.2
**17**	**c**	**17c**	7.7	8.3
**17**	**d**	**17d**	7.7	-
**18**	**a**	**18a**	4.4	7.7
**18**	**b**	**18b**	4.4	7.7
**18**	**c**	**18c**	4.4	5.1
**18**	**d**	**18d**	4.4	-
**19**	**a**	**19a**	n.d.	6.5
**19**	**b**	**19b**	n.d.	6.3
**21**	**a**	**21a**	7.7	8.7
**21**	**b**	**21c**	7.7	8.3
**21**	**c**	**21d**	7.7	7.0

Supplemental Experimental Procedures

Chemicals

Commercially available chemicals and reagents of the highest purity were purchased from Sigma-Aldrich (Poole, Dorset, UK), Fluorochem (Hadfield, Derbyshire, UK), or Thermo Fisher Scientific unless stated otherwise. Enzyme nicotinamide cofactors $NAD(P)^+$ and NAD(P)H were purchased from Cambridge Bioscience (Cambridge, UK). Media were purchased from Formedium (Hunstanton, UK).

General Procedure for chemical reductive amination. Secondary amine product standards were prepared as previously reported.[3] Briefly, 2 equiv. of $Ti(OiPr)_4$ was added to a solution containing 1 mmol ketone and 4 mmol amine in EtOH, and the reaction was stirred under Argon at room temperature for 18 h. The reaction was cooled on ice, and 2 equiv. $NaBH_4$ was added and stirred at room temperature for 1 h, after which 3 M HCl was added to quench the reaction. Then, 5 ml of water was added, and the solution was basified to pH 12 with 10 M aqueous NaOH. 10 ml of EtOAc was added to the basified solution and mixed vigorously, and the organic layer was collected. The aqueous fraction was further extracted with EtOAc, and the organic fractions were combined. 20 ml of water was added to the organic fraction, acidified to pH 1. The aqueous fraction was collected, basified to pH 12 with 10 M NaOH, and the amine product was extracted twice into dichloromethane (DCM). The DCM fraction was dried with anhydrous $MgS0_4$, and DCM was removed to recover the amine product. NMR data are in agreement with previously reported data.[3]

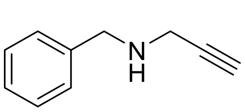

26a

Yellow oil isolated, 70% yield. **^{1}H NMR** (400 MHz, $CDCl_3$) δ 7.30 – 7.13 (m, 4H), 3.80 (s, 2H), 3.35 (d, *J* = 2.4 Hz, 3H), 2.18 (t, *J* = 2.4 Hz, 1H), 0.84 – 0.71 (m, 1H). **^{13}C NMR** (101 MHz, $CDCl_3$) δ 139.40, 128.46, 128.43, 127.19, 82.07, 71.58, 52.29, 37.35.

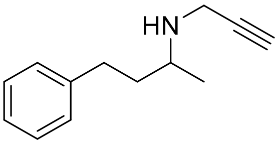

22a

Brownish-yellow oil isolated, 62% yield. **^{1}H NMR** (400 MHz, $CDCl_3$) δ 7.35 – 7.23 (m, 2H), 7.19 (ddt, *J* = 10.8, 6.3, 1.6 Hz, 3H), 7.16 (s, 1H), 3.53 – 3.35 (m, 2H), 2.99 – 2.86 (m, 1H), 2.79 – 2.58 (m, 2H), 2.19 (t, *J* = 2.4 Hz, 1H), 1.84 – 1.56 (m, 2H), 1.11 (d, *J* = 6.3 Hz, 3H). **^{13}C NMR** (101 MHz, $CDCl_3$) δ 142.29, 128.38, 128.36, 125.79, 82.37, 71.15, 51.15, 38.47, 35.61, 32.16, 19.81.

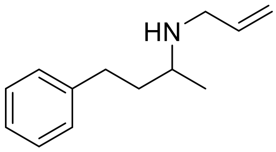

22e

Yellowish oil isolated, 73% yield. **^{1}H NMR** (400 MHz, $CDCl_3$) δ 7.41 – 7.28 (m, 2H), 7.28 – 7.19 (m, 3H), 5.97 (ddt, *J* = 17.1, 10.2, 6.0 Hz, 1H), 5.22 (dq, *J* = 17.2, 1.7 Hz, 1H), 5.20 – 5.09 (m, 1H), 3.35 (ddt, *J* = 13.8, 5.9, 1.5 Hz, 1H), 3.27 (ddt, *J* = 13.8, 6.2, 1.4 Hz, 1H), 2.84 – 2.63 (m, 4H), 1.86 (dddd, *J* = 13.5, 9.8, 6.4, 5.6 Hz, 1H), 1.78 – 1.63 (m, 1H), 1.17 (d, *J* = 6.3 Hz, 3H). **^{13}C NMR** (101 MHz, $CDCl_3$) δ 142.71, 137.48, 128.67, 128.65, 126.05, 115.98, 52.41, 50.13, 39.05, 32.65, 20.57.

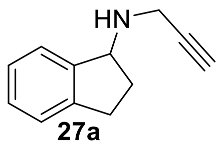

27a

Brow oil isolated, 58% yield. **^{1}H NMR** (400 MHz, $CDCl_3$) δ 7.40 (ddt, *J* = 6.7, 1.4, 0.8 Hz, 1H), 7.33 – 7.18 (m, 3H), 4.50 – 4.43 (m, 1H), 4.17 (q, *J* = 7.1 Hz, 1H), 3.57 (t, *J* = 2.6 Hz, 2H), 3.17 – 3.03 (m, 1H), 2.94 – 2.80 (m, 1H), 2.59 – 2.38 (m, 1H), 2.31 (t, *J* = 2.5 Hz, 1H), 2.04 – 1.85 (m, 1H), 1.35 – 1.22 (m, 1H). **^{13}C NMR** (101 MHz, $CDCl_3$) δ 144.48, 143.84, 126.69, 126.28, 124.89, 124.21, 82.46, 71.44, 61.89, 36.15, 33.31, 30.47

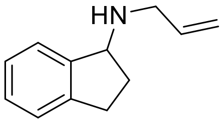

27e

Brown oil isolated, 67% yield. **^{1}H NMR** (400 MHz, $CDCl_3$) δ 7.40 – 7.30 (m, 1H), 7.27 – 7.14 (m, 3H), 5.98 (ddt, *J* = 17.1, 10.2, 5.9 Hz, 1H), 5.32 – 5.18 (m, 1H), 5.11 (dq, *J* = 10.2, 1.4 Hz, 1H), 4.27 (t, *J* = 6.6 Hz, 1H), 4.12 (q, *J* = 7.1 Hz, 1H), 3.45 – 3.27 (m, 2H), 3.01 (ddd, *J* = 15.9, 8.5, 4.9 Hz, 1H), 2.87 – 2.75 (m, 1H), 2.41 (dddd, *J* = 12.9, 8.3, 7.0, 4.9 Hz, 1H), 1.84 (dddd, *J* = 12.8, 8.5, 7.1, 6.1 Hz, 1H). **^{13}C NMR** (101 MHz, $CDCl_3$) δ 145.62, 144.05, 137.52, 127.84, 126.65, 125.19, 124.55, 116.23, 60.80, 50.47, 34.06, 30.78

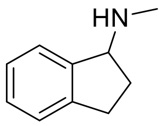

27c

Brown oil isolated, 56% yield. **^{1}H NMR** (400 MHz, $CDCl_3$) δ 7.29 – 7.20 (m, 1H), 7.20 – 7.03 (m, 3H), 4.11 – 3.95 (m, 1H), 2.90 (ddd, *J* = 16.0, 8.5, 5.2 Hz, 1H), 2.77 – 2.61 (m, 1H), 2.38 (s, 3H), 2.35 – 2.21 (m, 1H), 1.85 – 1.68 (m, 1H**).** ^{13}C NMR (101 MHz, $CDCl_3$) δ 144.19, 143.47, 127.30, 125.99, 124.53, 124.00, 64.37, 33.23, 32.29, 30.08

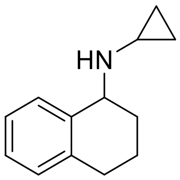

28a

Yellow oil isolated, 65% yield. **^{1}H NMR** (400 MHz, $CDCl_3$) δ 7.44 – 7.35 (m, 1H), 7.25 – 7.14 (m, 2H), 7.17 – 7.08 (m, 1H), 3.92 (t, J = 4.5 Hz, 1H), 2.93 – 2.73 (m, 2H), 2.36 – 2.27 (m, 1H), 2.12 – 1.92 (m, 3H), 1.88 – 1.75 (m, 1H), 0.65 – 0.38 (m, 5H). **^{13}C NMR** (101 MHz, $CDCl_3$) δ 139.73, 137.52, 129.37, 129.31, 126.93, 125.96, 56.08, 29.77, 29.08, 29.06, 19.32, 7.85, 6.59

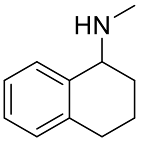

28c

Brownish-yellow isolated 52% yield**. ^{1}H NMR** (400 MHz, $CDCl_3$) δ 7.38 – 7.29 (m, 1H), 7.23 – 7.11 (m, 2H), 7.14 – 7.05 (m, 1H), 3.69 (t, J = 4.8 Hz, 1H), 2.89 – 2.66 (m, 3H), 2.51 (s, 3H), 2.07 – 1.92 (m, 1H), 1.92 (ddd, J = 6.3, 3.0, 1.7 Hz, 1H), 1.92 – 1.79 (m, 1H), 1.83 – 1.69 (m, 1H). **^{13}C NMR** (101 MHz, $CDCl_3$) δ 139.12, 137.72, 129.97, 129.45, 129.22, 127.09, 126.02, 57.40, 34.24, 29.68, 19.19.

Gene synthesis, cloning, expression, and protein purification.

Each gene sequence was optimised for *E. coli* expression, synthesised by Twist Biosciences, (South San Francisco, USA), and cloned into pET28a+ unless otherwise stated. Plasmid constructs of Bsp5 and Punc5 in pET22b have been kindly given to us by Dr Melanie A. Higgins. *Pv*DHFR, *Pf*DHFR, and *Sa*DHFR were each cloned into pET15b and were sourced from our in-house gene collections. A single colony of recombinant *E. coli* BL21 (DE3) containing pET28a/pET22b/pET15b-IRED was inoculated into 10 ml lysogeny broth (LB) (1% tryptone, 0.5% yeast extract, 1% NaCl, containing kanamycin 30 µg ml.−1 or ampicillin 50 µg ml−1) and incubated overnight at 37 °C in an orbital shaker at 200 r.p.m. This starter culture was used as the inoculum for subsequent growth.

Expression trial. Soluble expression of the target protein was trialled with isopropyl β-D-1-thiogalactopyranoside (IPTG) induction and LB autoinduction media (Formedium, Hunstanton, UK). A 250 ml flask containing 50 ml LB was supplemented with (30 µg ml.−1) or ampicillin (50 µg ml−1), depending on the construct, and inoculated with 0.5 ml of starter culture. For expression in LB media using IPTG induction, initial cultivation was performed at 37 °C in an orbital shaker with shaking at 180 r.p.m. At an optical density (OD600) of between 0.6 and 0.8, IPTG was added to a final concentration of 0.4 mM to induce protein expression. Incubation was continued at 22 °C with shaking at 180 r.p.m. for 18 h. For expression using autoinduction media, cultivation was initially performed at 37 °C in an orbital shaker with shaking at 180 r.p.m for 5 h, after which further cultivation was continued at 24°C for a further 40 h. Soluble expression was comparable for most of the investigated enzymes when using IPTG induction or autoinduction conditions.

Cell lysis was performed either by ultrasonication (5 × 20 s bursts with 40 s intervals at 4 °C) or incubation in lysozyme-based lysis buffer (100 mM Tris-HCl, 100 mM NaCl, pH 7.5, supplemented with 1 mg ml^{-1} lysozyme from chicken egg white, 0.3 mg ml^{-1} polymyxin B) incubated at 20 °C for 1h 30 min. Cell debris was removed by centrifugation (12,000 r.p.m, 4 °C, 30 min), and the clarified soluble cell-free extract was analysed by SDS-page gel electrophoresis, purified, or used for biotransformation.

Protein production and (semi)-purification. A 2-l flask containing 500 ml LB was supplemented with kanamycin (30 µg ml.−1) or ampicillin (50 µg ml^{-1}) and inoculated with 5 ml of starter culture. Cultivation was performed at 37 °C in an orbital shaker with shaking at 180 r.p.m. At an optical density (OD600 nm) between 0.6 and 0.8, isopropyl β-D-1-thiogalactopyranoside (IPTG) was added to give a final

concentration of 0.4 mM to induce protein expression. Incubation was continued at 22 °C and 180 r.p.m. for 18 h. Cells were then harvested by centrifugation and washed in Tris-HCl buffer (100 mM, pH 7.5).

For quick (semi)-purification, cells were resuspended in buffer A (50 mM Tris-HCl buffer, 300 mM NaCl, 25 mM imidazole, pH 7.5) and were disrupted by ultrasonication at 4°C employing 5 × 20 s bursts with 40 s intervals at 4 °C. Following centrifugation of the lysed cells (12,000 r.p.m, 4 °C, 1 h), the clarified lysate was purified by Ni-affinity chromatography using gravity columns. Clarified lysate was loaded onto the column, followed by a single-step wash with buffer A, and eluted with buffer B (50 mM Tris-HCl buffer, 300 mM NaCl, 300 mM imidazole, pH 7.5). The elute was desalted using 10 kDa cut-off centricon filters (15 ml) and the buffer was exchanged to Tris-HCL buffer (50 mM Tris, 100 mM NaCl, pH 7.5) or HEPES (30 mM, 100 ml NaCl, for BsP5, Punc5, NAPW).

Biotransformation Reactions. *For imine reduction using crude lysed cells*. An initial biotransformation reaction to assess imine-reducing activity was performed using crude lysed *E. coli* cells expressing an IRED. Biotransformation reactions typically contained *E. coli* BL21(DE3) resting cells containing expressed IRED (OD600 nm of 50), 0.3 mM NADP+/0.3 mM NAD+, 1-2% (v/v) DMSO, 5 mM imine substrate, 20 mM D-glucose, 0.3 mg ml^{-1} glucose dehydrogenase (GDH) as lyophilised cell free extract (CFE), 0.5 mg ml^{-1} lysozyme, and 0.25 mg ml-1 polymyxin B in sodium phosphate buffer (100 mM, pH 7.0, 100 ml NaCl). Reactions were incubated in an orbital shaker at 20 °C with shaking at 200 rpm for 48 h.
Biotransformation reactions using cell-free extract or (semi)purified enzyme preparation were performed employing glucose dehydrogenase (GDH)/NADP+ as a cofactor recycling system. A typical 500 µl reaction mixture contained 20 mM D-glucose, 0.3 mg ml^{-1} GDH (lyophilised CFE), 0.3 mM NADP+, 0.1-1 mg ml^{-1} IRED, 5 mM imine and 2% (v/v) DMSO. The reaction volume was made up to 500 µl with sodium phosphate buffer (100 mM, pH 7, 100 mM NaCl). Reactions were incubated at 25°C with shaking at 200 r.p.m. for 24 h.

For IRED-catalysed reductive amination of ketones or aldehydes. A 500 µl reaction mixture contained 50 mM ketone (30 mM for hydrocinnamaldehyde, and 10 mM for other carbonyl substrates), 30-100 mM D-glucose, 0.3 mg ml^{-1} GDH (lyophilised cell-free extract), 0.5 mM NADP+ in Tris-HCl buffer (100 mM, pH 9.0) containing 2% (v/v) DMSO. 2-10 equivalents of amine (from 1M buffered amine nucleophile solution stock solution, pH 9) were then added, and the reaction was initiated with the addition of 0.2-1 mg ml^{-1} IRED. The reaction volume was made up to 500 µl with Tris-HCl buffer (100 mM, pH 9.0). Reactions were incubated at 25 °C with shaking at 200 r.p.m. for 24 h.

For analysis, reactions were quenched by adding 60 µl of 5 M NaOH and extracted twice with 500 µl tert-butyl methyl ether. The organic fractions were combined and dried over anhydrous MgSO4 and analysed on HPLC using chiral columns (CHIRALPAK®IC 250 mm × 4.6 mm, 5 µm; and CHIRALPAK® IB N-5, 250 mm × 4.6 mm, 5 µm). Samples were run at 1 ml min^{-1} and monitored at 265 nm or by GC-MS (CD-5MS Capillary Column 30 m x 0.25 mm x 0.25µm (P/N MOD-GC-CD5MSU-5, S/N CD17506).

Preparative Biotransformation for the imine reduction of 1-methyl-3.4-dihydroisoquinoline (using MaRedAm, ZtNR, and LtPR1 as representative IREDs). Preparative biotransformation reactions for the reduction of the imine, 1-methyl 3,4-dihydroisoquinoline **8** (100mg, 0.69 mmol, total reaction volume, 28 ml) were performed in a 250 ml glass flask. The reaction mixture contained 100 mg imine **8** (25 mM), D-glucose (2.0 equiv., 50 mM), 0.6 mM NADP$^+$, and 2% v/v DMSO. The mixture was dissolved in 18 ml NaP*i* buffer (100 mM, pH 7), and the pH of the reaction mixture was adjusted to pH 7. The enzymatic components, including purified IRED (1 mg ml^{-1} *Ma*RedAm or 2 mg ml^{-1} *Zt*NR, or 2 mg ml^{-1} *Lt*PR1) and GDH as CFE (0.5 mg ml^{-1}), were then added to this reaction mixture. The reaction volume was then made up to 28 ml with NaP*i* buffer (100 mM, pH 7.0). The reaction flask was sealed and incubated at 25 °C in an orbital shaker with 140 rpm shaking for 24 h (*Ma*RedAm) or 48 h (for *Lt*PTR1 and *Zt*NR).

The biotransformation reaction was quenched by the addition of 5 M NaOH (basified to pH ~12) and extracted twice into EtOAc (2 x 20 ml) with intermediate centrifugation (4000 r.p.m, 5min, 10 °C). To the combined organic fractions, 20 ml of water was added, the pH was adjusted to pH 1 with 3 M HCl, and the aqueous layer was collected. The pH of the aqueous fraction was then readjusted to pH 12 with 5 M aqueous NaOH. To extract the amine product from the organic layer, 20 ml of dichloromethane (DCM) was added to the basified aqueous layer; this step was repeated. The combined DCM fractions were dried with anhydrous MgSO4, and the clarified DCM solution was recovered. DCM was then removed under reduced pressure to afford the corresponding amine **9.** Data from isolated product were

compared to commercial racemic amine standard **9** and *e.e.* values were determined using chiral HPLC, See Figure S8. *R*, *S*-configurations were assigned from comparison of isolated product with the enantiomer product obtained with IREDs of known selectivity (*Ao*IRED for the *S*-amine product and AspRedAm for the *R*-amine product.

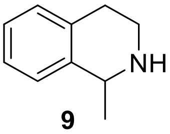

9

Yellow oil isolated. **^{1}H NMR** (400 MHz, $CDCl_3$) δ 7.41 – 7.22 (m, 3H), 4.35 – 4.25 (m, 1H), 3.45 (dt, *J* = 12.5, 5.1 Hz, 1H), 3.33 – 2.98 (m, 2H), 2.98 – 2.86 (m, 1H), 2.84 (s, 1H), 1.65 (d, *J* = 6.7 Hz, 3H), 1.03 (td, *J* = 13.7, 7.6 Hz, 1H). **^{13}C NMR** (101 MHz, $CDCl_3$) δ 138.43, 134.64, 127.53, 127.28, 125.93, 125.91, 51.55, 41.66, 27.86, 22.60.

Preparative biotransformation for the reductive amination of cyclohexanone with propargylamine (using MaRedAm, and LtPR1 as representative IREDs). Preparative scale biotransformation reactions for amination of cyclohexanone **1** (100mg, 1.02 mmol, total reaction volume, 41 ml with 2 equiv. of propargylamine **a,** were performed in 250 ml glass flasks. The 41 ml reaction mixture contained 100 mg cyclohexanone (25 mM), amine (2 equiv., 50 mM), D-glucose (2.0 equiv., 50 mM), 0.6 mM NADP+ and 2% v/v DMSO. The mixture was dissolved in 30 ml Tris-HCl buffer (100 mM, pH 8), and the pH of the reaction mixture was adjusted to pH 8.

The enzymatic components, including purified IRED (0.3 mg ml^{-1} MaRedAm or 1 mg ml^{-1} *Lt*PR1) and GDH as CFE (0.3 mg ml^{-1}), were then added to this reaction mixture. The reaction volume was then made up to 41 ml with Tris-HCl buffer (100 mM, pH 8.0). The reaction flask was sealed and incubated at 25 °C in an orbital shaker with 140 rpm shaking for 24 h or 48 h.

The biotransformation reaction was quenched by the addition of 5 M NaOH (basified to pH ~12) and extracted twice into EtOAc (2 x 20 ml) with intermediate centrifugation (4000 r.p.m, 5min, 10 °C). To the combined organic fractions, 20 ml of water was added, and the pH was adjusted to pH 1 with the addition of 3 M HCl, and the aqueous layer was collected. The pH of the aqueous fraction was then readjusted to pH 12 with 10 M aqueous NaOH. To extract the amine product from the organic layer, 20 ml of dichloromethane (DCM) was added to the basified aqueous layer; this step was repeated. The combined DCM fractions were dried with anhydrous $MgSO_4$, and the clarified DCM solution was recovered. DCM was then removed under reduced pressure to afford amine **16a.**

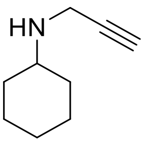

16a. Yellow oil isolated, (84% yield for MaRedAm-catalysed reaction and 63% yield for *Lt*PTR1-catalysed reaction) **^{1}H NMR** (400 MHz, $CDCl_3$) δ 3.42 (d, *J* = 2.5 Hz, 2H), 2.62 (tt, *J* = 10.3, 3.7 Hz, 1H), 2.16 (t, *J* = 2.4 Hz, 1H), 1.87 – 1.76 (m, 2H), 1.76 – 1.63 (m, 2H), 1.63 – 1.53 (m, 1H), 1.33 – 0.97 (m, 6H). **^{13}C NMR** (101 MHz, $CDCl_3$) δ 82.52, 70.96, 54.95, 35.07, 33.00, 26.07, 24.78. GCMS (EI) m/z = 137.18.

Supplemental: Gene Sequences

>LmPTR1
ATGACAGCTCCCACTGTACCAGTTGCACTAGTGACCGGCGCTGCTAAACGTCTGGGCAGAAGCA
TTGCTGAGGGCTTGCACGCCGAGGGCTACGCAGTTTGTCTGCACTACCATCGTAGCGCGGCGG
AGGCGAACGCGCTGTCTGCGACCTTGAATGCGCGTCGTCCGAATAGCGCGATCACCGTTCAAG
CAGACCTGAGCAACGTGGCCACGGCTCCGGTGAGCGGTGCGGACGGCTCTGCTCCGGTCACCT
TATTCACCCGTTGCGCAGAGCTGGTCGCCGCGTGCTATACCCATTGGGGTCGCTGCGACGTGCT
GGTGAACAACGCTAGCTCGTTCTATCCGACCCCGTTGCTGCGTAATGATGAAGATGGTCACGAA
CCGTGTGTTGGTGATCGTGAAGCCATGGAAACCGCGACCGCGGACCTGTTCGGCTCTAACGCC
ATCGCACCGTATTTTCTGATTAAAGCGTTTGCACATCGCTTCGCTGGTACACCGGCGAAACACCG
AGGTACGAACTATTCCATCATCAACATGGTTGATGCTATGACCAACCAGCCGCTGCTGGGCTACA
CGATTTATACCATGGCAAAGGGTGCGCTTGAGGGCTTGACTCGCAGCGCGGCGCTGGAGCTGG
CACCACTGCAAATTCGTGTTAATGGTGTCGGTCCGGGTTTGTCCGTGTTGGTAGACGACATGCC
GCCTGCGGTGTGGGAAGGCCACCGCAGTAAGGTTCCGCTGTACCAGCGTGATAGCTCCGCGGC
TGAAGTTTCCGATGTTGTTATTTTTCTTTGCAGCTCAAAGGCAAAGTACATCACTGGTACGTGCGT
CAAAGTGGACGGCGGTTACAGCCTGACCCGCGCATAA

>*Tb*PTR1
ATGGAAGCCCCTGCCGCAGTAGTAACGGGTGCCGCAAAACGTATTGGTCGTGCCATCGCGGTTA
AATTACATCAGACGGGTTATCGCGTCGTCATCCATTATCATAACTCGGCGGAAGCAGCCGTCTCG
CTGGCTGATGAACTCAATAAAGAACGTTCCAATACCGCCGTTGTTTGCCAAGCAGATCTTACCAA
TTCCAACGTTTTACCGGCATCCTGTGAGGAGATCATCAATTCGTGCTTTCGCGCGTTTGGTCGTT
GTGATGTCTTAGTGAATAACGCGTCCGCGTTTTACTCAACGCCCCTGGTGCAGGGTGATCACGA
AGACAACTCTAACGGCAAGACGGTGGAGACACAAGTCGCGGAATTAATCGGTACGAATGCAATT
GCCCCCTTTCTGCTCACCATGTCTTTTGCTCAGCGTCAGAAAGGGACGAATCCAAATTGCACATC
TTCAAACCTGTCTATCGTAAACCTGTGTGACGCGATGGTCGACCAACCTTGTATGGCGTTTTCTT
TATATAATATGGGCAAGCATGCGCTGGTCGGGCTTACCCAAAGCGCCGCCTTAGAACTCGCTCC
CTATGGTATCCGCGTAAATGGCGTAGCCCCGGGAGTTTCGTTACTGCCGGTAGCTATGGGCGAG
GAAGAGAAAGACAAGTGGCGTCGCAAAGTGCCACTGGGCCGCCGCGAAGCGTCTGCCGAGCA
GATTGCGGACGCCGTGATCTTCCTCGTGTCAGGCTCGGCACAATATATTACAGGCAGTATTATTA
AGGTTGATGGCGGCCTGTCATTAGTCCACGCGTAA

>*Tc*PTR1
ATGAACGAGACGTCTCACGAAGCAAGTGAATGCCCGGCCGCCATCATTACGGGTGGTGCCCGT
CGCATCGGCCATAGCATTGCGGTTCGTCTTCACCAACAGGGCTTTCGTGTGGTGGTTCACTACC
GCCACTCAGAGGGCGCTGCCCAACGTCTCGTGGCAGAGCTGAATGCCGCACGCGCTGGATCCG
CGGTTCTGTGCAAAGGAGATCTGAGCCTTGGATCATCGTTGTTAGATTGCTGTGAAGATATCATC
GATTGTAGTTTCCGTGCGTTTGGTCGCTGCGACGTACTGGTTAATAACGCCTCGGCGTACTATCC
CACTCCGCTGCTCCCAGGTGATGATACTAATGGAGCCGCAGATGCAAAGCCCATTGATGCTCAA
GTGGCCGAACTCTTCGGCAGCAATGTCGTGGCCCCTCTTTTCCTGATCCGTGCTTTTGCGCGCC
GTCAAGGCGAAGGCGATTCTTGGCGCAGTCGCAACCTTTCAGTGGTTAATCTGTGCGATGCGAT
GACCGACCTGCCTTTACCTGGATTTTGTGTATATACAATGGCGAAACACGCCCTGGGTGGGCTTA
CTCGCGCAGCAGCGTTAGAGTTGGCGCCGCGTCATATTCGCGTAAACGCGGTTGCGCCAGGCC
TGTCATTATTTCCGCCGGCAATGCCACAAGAAACGCAAGAGGAATATCGCCATAAAGTTCCACTG
GGACAAAGTGAAGCGAGCGCGGCGCAGATCGCTGATGCTATTGCCTTTCTGGTCAGCAAGGAC
GCAGGGCACATCACTGGGATCACCCTGAAAGTTGATGGCGGTTTGATTTTGGCTCGCGCGTAA

>*Lt*PTR1
ATGACGACCAGCCCGACGGCCCCTGTAGCACTTGTGACTGGAGCAGCTAAGCGTCTGGGTTCTA
GTATTGCGGAAGCGCTGCACGCTGAGGGATATACCGTATGCTTGCACTATCATCGTTCAGCGGC
CGACGCCAGCACGTTAGCAGCAACCTTAAATGCCCGTCGCCCTAATAGCGCCATCACCGTCCAG
GCCGACTTAAGTAATGTCGCAACGGCATCGTTTTCTGAGACGGATGGCTCCGTCCCAGTTACAC
TCTTCTCGCGCTGTAGCGCGTTGGTTGACGCCTGTTATATGCACTGGGGTCGTTGCGACGTTCT
TGTTAATAACGCATCAAGTTTCTACCCAACGCCGTTGCTGCGTAAGGACGCAGGTGAGGGCGGT
TCGAGCGTAGGTGACAAAGAAAGCCTGGAAGTAGCCGCTGCGGATCTCTTTGGCTCGAATGCCA
TTGCACCGTACTTCCTGATCAAGGCCTTCGCACAGCGCGTGGCCGACACCCGCGCAGAACAAC
GTGGAACGTCATACTCGATCGTGAATATGGTAGATGCCATGACCAGCCAGCCATTATTGGGCTA
CACCATGTACACCATGGCGAAAGAGGCACTTGAGGGCCTTACTCGCTCAGCAGCGTTGGAGCTT
GCAAGTCTTCAGATCCGTGTAAATGGTGTCTCGCCGGGCTTAAGTGTTTTACCTGATGATATGCC

TTTCTCGGTTCAAGAGGATTATCGTCGCAAGGTCCCCCTTTACCAACGCAACTCGTCGGCCGAG
GAAGTGAGCGACGTAGTTATCTTCCTGTGTTCACCGAAGGCCAAGTACATTACCGGCACTTGCAT
CAAGGTGGACGGCGGGTATTCGCTCACACGTGCGTAA

>*Am*PTR1
ATGACTAACTCGGGTCCGGTTGCTTTAATCACTGGTGCGGCGAAACGTATTGGCGCGGCCATGG
CCATGAAGCTCCATAATGAGGGCTACCGCGTTATCATCCACTATGGCCATAGCGAGAACGATGC
TCTTTCCCTGGCGGCTCGCTTAAACCAGAAACGTGCGAATTCAGCATTCTGTTTGCAGGCCGAC
CTGTGTGACACGCATGCCGTCAGTGCGCTGGGTGAAGAGGCTGTGAATGTGTGGGGTCGCTTG
GACGTCCTTGTAAACAATGCTTCCTCATTTTATCCAACGCCGGTGGGAGACATTACAGAAGAGGA
TTGGACTAGCTTAGTAGGTTCAAACGTTAAGGGGCCACTGTTCCTCAGTCAGGCCCTGACCCCC
GCCTTAAAGAAGTCAAACGGGTGCATTGTTAACATGGTAGACATGCATATTGATCGTCCCCTGCC
GAAGCATTCTGTGTATCTCCTTGCCAAGTCAGGCCTGGCTTCTCTGACACAGTCACTCGCAATTG
ACCTTGCGCCAAACATTCGTGTGAACGGTATTGGACCAGGGGCGATTCTCTGGCCTGAACGCGA
GATGGAAGATGCAGAGAAGGACACTCTGCTGTCGTCTATCCCTCTGGGAGAGCTTGGCACACCG
GATGACATCGCCAATACACTGTGGTTCCTGATCAGCGCGCCCTACATTACGGGCCAGATCATCTT
CGTGGACGGCGGCCGCTCACTGCACACAGGAGCGTCAGCATAA

>NpNR
ATGAGTTTAGAAAAAAGGTGGTCACTAGAGGGAACCACGGCTCTGGTTACCGGTGGCACCAAGG
GTATTGGCCGTGCGATCGTGGAAGAGTTGGTGGGTTTTGGTGCTCGTGTCTATACGTGCAGCCG
TAACGAAGCTGAGCTGCGTAAGTGCCTCCAAGAGTGGGAAAATCTGAAATACGACGTGACAGGC
TCCGTGTGTGATGTTTCGAGCCGCACCGAGCGCGAGAAGTTGGCCGAGGACGTCTCCTCTGTTT
TTAACGGCAAATTGAACATCCTGATTAATAACGCGGGTGGTTATGTGAACAAACCGATTGACGGC
TTCACCGCGGAGGACTTCAGCTTCCTGGTGGCCGTTAACTTGGAAAGCGCGTTTCATCTGTGCC
AGCTGGCCCACCCGATGCTGAAGGCCTCAGGCACGGGTTCTATTGTGCACATCAGCTCCAGCTG
CGCACAAATTGCGATTCCGGGTCATAGCATCTACAGTAGCACCAAAGGTGCGATCAACCAGCTG
ACCCGTAACTTGGCGTGTGAATGGGCAAAGGACAACATCCGCACCAATAGCATCGCACCGGGTG
CGATCCGTACCCCGGGCACCGAACCATTTGTTAATGATAAAGATGCGCTGGATCGTGAGGTTAG
CCGTGTTCCGAGCGGTCGCATTGGTGAACCGGAAGAGGTCGCGTCTCTGGCGGCTTTCTTATGC
ATGCCGTCCGCAAGCTACATCACTGGCCAGGTAATTTGTGTTGATGGTGGGAGAGCTATCAATG
GCTAA

>*NpaNR*
ATGAGACGGCGCGAAGAGGAATCACTGTCAATGCCAATGGAGAAAAAACGCTGGTCGCTGGTG
GGTGCAACGGCGCTTGTAACGGGGGGAACAAAGGGAATTGGCAGAGCTATCGTCGAGGAACTG
GCCGGATTTGGGGCGAGAGTGCATACGTGCTCGCGCAATAAAGCGGAATTGAACGTGTGCCTG
CAGGAATGGGAGAAACTGAAGTTGGATGTGACAGGATCAGTCTGCGATGTCTCTTCTCGTACAG
AACGAGAAAAACTGATGGAAGAGGTCTCTTCGGTATTTAATGGTAAATTGAATATTCTGATAAACA
ATGCCGGCACAGCGATCCTGAAGCCAATTCTTGATTTCACCGATGAAGATTGTTCCTTTTTAGTA
GCGACTAACTTTGAATCTGCGTTTCACTTGAGTCAGCTGTCGCATCCAATGCTGAAGGCGTCCG
GAGTTGGCAGCATTGTGCATATCAGTACAGTATGCACATTTATTGGCCTCGAAGGCCACTGTATT
TACAGCGCCACCAAAGGTGCTATGTCGCAATTGACGAGAGATTTTGCTTGTGAATGGGCACGTG
ATGGTATTCGTACGAACTGCATCGCTCCGGGTATTACCCGCACCGTACAGGTCCAACCGTTCCT
GGACGATAAAGACGCTGCGGCGAAGGAAATGAGCCGCATCCCGAATGGTCGCCCAGGTGAGCC
AGAAGAAATGGCTTCTCTTGCTGCGTTTTTATGCATGCCAGCCGCTTCATATATAAATGGACAGG
TTATTTGCGTCGATGGTGGGCGAGGAATCAATGGTTAA

>*Lr*NR
ATGCGTTGGAGTCTTCGCGGGCGCACTGCCTTAGTTACATCAGGTACGAAGGGTATCGGCCACG
CGATCGTTGAGGAGCTGGCGGGCTTAGGCGCTGTGGTATATACGTGTTCGCGCAATGAGACCG
AACTGAATAAGTGCCTGCAAGAATGGAAGAATCTTAAACTGAATATTAGCGGCAGCGTATGCGAC
GTTTCATCACGCCCCGAACGTAAGAAGTTAATGGAAAACGTGACGTCAATCTTCGACGGAAAAGT
TAATATTCTGATCAATAATGCCGCGTCTACACTGTATAAGGCGGTGGTGGACTGTACGGCTGAGG
ATTACAGCTTTATTATGGCGGCAAATTTTGAAAGCGCCTTTCACCTGTGTCAGCTGGCGCATCCG
ATGCTGAAAGCCAGTGGTACCGGGTCCATTGTGCATATTTCCAGCGCGTGTGCGGGTATCGCGA
TTCCTGGACATACGATCTACTCCAGCACCAAAGGGGCCATTAACCAACTGACCCGCAACCTCGC
GTGCGAATGGGCCAAGGACAACATTCGCACGAATAGCATTGCTCCTGGCGCCATTCGCACTCCC
GGGACTGAGAGCTTCGTCGAAGACAAAGACGCCCTGGACCGCGAAGTGTCGCGCATCCCTTTT

GGTCGTATTGGGGAACCCGAGGAAGTGGCATCGCTGGCAGCCTTTCTGTGTATGCCGAGCGCG
AGCTATATTACCGGCCAGGTGATTTGTGTTGACGGTGGCCGCACGATTAACGGGTAA

>*Zt*NR
ATGCGTTGGAGTCTTAAAGGTACTACAGCGCTTGTTACGGGCGGCACGAAGGGGATCGGTCAC
GCGATCGTGGAAGAATTGGCTGGTTTTGGGGCTCGCGTGTATACTTGTAGCCGCAACGAAGCCG
AGCTGACCAAATGCTTGCAAGAATGGGAAAATTTAAATTTCGACGTGGCAGGAAGCGTGTGTGA
CATTGCCTCACGTACCGAACGCGAGGAGCTGATGGAGCGTGTATCAAGCGTGTTCAACGGAAAC
CTGAATATCTTAATTAATAATGCGGGTGGTTACGTGAACAAACCAATCGACGATGTTACCGCCGA
AGATTTTAGTTTTCTGGTGGCGGTAAATCTCGAAAGCGCATTTCATCTCTGCCAGCTCGCGCACC
CTATGCTCAAAGCATCGGGTCGTGGTAGCATTGTCCATGTGAGCTCGTGCTGCGCTCAGATCGC
GTTACCTGGGCATTCGATGTATTCTGCCACCAAGGGAGCAATTAATCAGTTGACCCGTAATCTCG
CGTGTGAGTGGGCGAAAGATAACATTCGCACCAACACCGTAGCTCCGGGAGCCATTCGCACGC
CGAGTTCGGAACCGTTTGTCAATGACAAAGATGCGGTGGCTAAGGAAGTCGCGCGCGTACCGCT
CGGCCGTATTGGGGAGCCGGAAGAGGTAGCTGCCATCACGGTATTTCTGTGTATGCCGGCTGC
CAGTTATATTACAGGTCAGGTCATTTGTGTCGATGGCGGCCGTACGATCAACGGCTAA

>*Ls*NR-like
ATGGATTTCAGCTGGCAGTTAACCCATCAACGCGCCCTGGTCACGGGTGGCACCAAAGGTATCG
GTCGTGCAATCGTCCAGCAATTGCTTCAGTTCGGTGCTTCTATCTTTATCGTTGCACGCGATAAC
GCCCTTCTGCAACAGCAATTACAGGAATACCGTCAAAACGGCTTTTCGGTCGACGGCCTTGCCA
CGGATATTTCGCAGCCTGGAGCCGCACAACGCTTAGTTGAAACCGTCCAAGACCGTTGGGGTTC
ATTAGACATCCTGATCAACAACGCGGGAACCAACATCCGCAAACCTACCACCGACTACCGCCCG
GATGAATTTGACCAGATTCTGAACACTAACCTTCGCAGCGCCTACGAGCTGTGCCAGGCTGCGT
ATCCACTGCTGAAAAGTAGCGGAAAATCTCGCATTGTGTTTGTGAGCTCAGTAAGCGGCCTCAC
GCACACCTCTTCTGGTTCGATCTACGGGATGACTAAGGCAGCCCTGCACCAACTGACTCGCAAC
CTGGCCGTGGAGTGGGCACCGGACGGAATTCGTGTAAATGCAGTGGCACCCTGGTACATCCGT
ACGCCGCTTGCCGAACCGGTTCTTTCAGACCCGGAACGTCTCAATCGCATTCTGAGTCGCACGC
CGCTGGGCCGCATCGGCGAGCCAGAAGAGGTCGCAGCAACCGTCGCGTTTCTGTGTCTTCCCG
CAGCAGGTTACATCACCGGTCAGACACTGGCTGTAGACGGTGGAATGACTGCTTGGGGAATGTA
A

>*Pt*IRED
ATGTAAACCCGCTATAGCAGTACTAGGAGTTGGTCGTATGGGCAGCGCGCTGGTCAGCGCATTC
CTGAAACAGGGCTACGGCGTTGATATCTGGAACCGTACCCGTGCGAAGTGCGAACCGCTGGCG
GCTCAAGGCGCTCGCATTGCAGCGACGGTAAGAGATGCGGTGGCAGCTGCGGACATCGTGGTG
GTCAACGTAAACGATTATGGTACTTCCGACTCTCTGTTACGTCCGGACGAAGTTACCCGTGCATT
GCGCGGTAAACTGCTCGTCCAGCTGACCTCCGGCTCCCCGAGCCAGGCCCGTGAGCAAGCGAC
GTGGGCACGCCAGCACGGCATTCCATACCTGGACGGCGCGATTATGGGTACTCCGGACTTCAT
CGGACAGCCGGGTGGCACCCTGCTGTACAGCGGTCCGTCGGAACTGTTCGAGCAATATAAACC
GGTGTTGTTGACCTTAGGTGGTAATACCCAGCACGTGGGCGCGGACGTTGGCCACGCAGCGGC
TCTCGACAGCGCGCTGCTGGTGTATTTATGGGGTGCTATGTTCGGCGTTTTGCAAGGCGCGGCT
GTGTGTGAAGCGGAAAAGGTGCCGCTGGAGAGCTTTATGGGTTACGTCAAGGCTACCAGCCCT
GTGGTTGAAGGTGCCGTGACCGATGTTCTTATGCGTGTTCAGCAAGGCCGTTTTGGTTCTGACG
CGACCACCCTGGCCACGTTGGAGATCCACCATGGTGCGTTGCGCCACCTGCTGGAGCTGTGCC
GTGAGCGCGGTCTGCATCGTGAGATGCCGGAAGCATTTGATCGTCTGTTCCAGAAAGCGCTGCA
AGCCGGTCATGCCCAAGATGATTTTGCAGTTCTATCACGTTTTATGCGCTAA

>*Bac*RedAm
ATGAGGGAACCCATAGTAAGTGCTCACACAGAGCGCGCAGTCGAGTCTCGTGGCGCGGACCGT
GGTTCTGCGGTTACCGTCATCGGCTTGGGTTCCATGGGTTCAGCCCTCGCCGGCGCGGTGCTG
GAAGCGGGCTATCCGACGACCGTTTGGAACCGCACGGCTGGTAAGGCAGAACCATTGGTGCGT
AGAGGCGCGGCTCGCGCGGCGACGGTGGCGGAGGCGGTGAGCGCGTCCCCGACCGTGATCG
CCTGCGTGCTGGATTATCGTGCGTTACGTGAGATCCTGAGCACCGCGGGCGACGCACTCGCTG
GCCGTACCGTTGTTAATCTGACCAACGGTACACCGACCGAGGCCCGTGAAACCGCTGCTTGGGT
CGAGGGTCATGGTGCTCGTTACCTGGACGGCGGCATCATGGCAGTTCCGGAAATGATTGGTGG
CGCGGAAAGCCTTGTTCTGTATAGCGGTAGCGCCGAGGCGTTTGAAACCGTAGAGCCGGTTCTG
CGTCGCTTCGGCAGCGCTATGTACCTGGGTGCGGACCCGGGTTTGGCCTCGCTGCACGATCTG
GCATTGCTGGCGGGGATGTACGGCCTGTTTGCAGGCTTCCTGCACGCAGTGGCCTTAGTGGGT
ACGGAAGGTGTTCGTGCCACCGAGTTCACCAGCTCCCTGCTGATTCCGTGGCTGCAGGCCATGA

CCGCGACTCTGCCTGAAGCTGCGGCGCAAATTGATGCAGGCGACTACGCAGCGACTGGTTCTC
GCCTGGACATGCAAGCGGTCGCGTTGGCGAACATTGTTGAGGCGAGCAGAAGCCAGGGTATCC
GTCCGGATCTTATGCTGCCGATTCAGGCATTGGTTGAGCGCCGTGTGGCAAAAGGTGGTGGCG
GTGAAGATATCGCAGCGGTGGTGGAGGAAGTTCGCGGATAA

>*Ma*RedAm
ATGACTTCTTCCTCCACTGTTAGCATTATCGGCCTTGGCGCCATGGGCTTGGCCCTTGCTGCCAA
GTTTGTGGAGAAGGGCTACACGACCACTGTTTGGAACAGATCCACCGAGAAGGCACTCAAGTTT
GCTGCAGAGCACGAGAACGCGCATGCCGCAACCACCGTGGCTCAAGGTCTAGAGGCTAGCAAC
CTGGTGATCATCTGTCTTCTCGACAACAAGGCCGTTCGCGATACCATTGATCAAGCCCTTCCCTC
CTTGGCGGGACGCATCGTTGTCAACCTGACTAACGGTACCCCTGATGAGGGACGAGAGACTGG
GGCCCTTGTGGCGGCTCAGGAAGGATCCAAGTACGTGCACGGCGGGATCATGGCAACTCCTTC
GATGGTCGGCTCTCCGGCATCGGTGCTGCTGTACAGCGGATCTCTAGAAGCTTACACAGCGGTG
GAGAAGGATCTCGAGATCCTGGGTGCTGGCAAGTACCTCGGAGCCGACTCTGGATCGGCTTCG
CTGCATGATCTGGCGCTCCTGAGCGGGATGTATGGCCTCTTCTCTGGCTTTACGCATGCAGTGT
CGCTGGTGCGGAACGAGAAGCGGTCGACCACGGAATTCCTGTCGCTTTTGGTGCCCTGGCTGA
CGGCGATGACGGGCTACCTGCACGTGCTGGGCAAGCAGATTGATGAGGGCGACTTCTCGTCTC
TTGGGTCGAGTATTGAGATGCAGGTGCCTGCGATCAACAACATTGTGAAGACAAGCGAGGCACA
GGGCGTGTCTGCGGATCTCATCCGGCCCATCCAAGGCTTGCTGGAGCGTGCGGTGGCGGTCG
GACGAGGTGGCGAGGAGATCTCGGCGCTGGTGGGCCTGAATGTGCTGGCGAGGAAGGCAGAG
TAA

>AserRedAm
ATGTCGAAGCACATAAGCATATTCGGCCTCGGTGCTATGGGAACAGCTCTCGCAGCCAAGTATC
TCAGACATGGCTATAACACAACCGTTTGGAATCGAACCACTGCAAAGGCAACTCCGCTCGTTGA
GCAGGGTGCCAAGCTAGCCTCTACAATCTCGAAGGGGGTAGACGCCAGCGACCTCATCGTTATA
TGCCTCCTTAACAATCAAGTTGTCGAGAACACTCTACGGGATGCATTACACATTCTGTCCAGTAA
GACCATTGTCAACCTTACCAATGGGACACCAAACCAGGCTCGTAAGCTCGCAGATTTCGTCACCT
CCCACGGAGCACGGTACATCCACGGCGGCATTATGGCGGTTCCCACCATGATTGGCTCTCCACA
CGCTGTCCTGCTTTACAGTGGAGAGTCTCTTGAGCTATTCAAGAGCATCGAAAGTCATCTGTCTC
TCCTTGGGATGTGCAAATATCTCGGCACCGATGCAGGGTCTGCCTCTTTGCATGACTTGGCTCT
GTTGTCGGGAATGTACGGTCTCTTCTCCGGGTTCCTGCATGCGGTTGCTCTGATCAGGTCCGAG
CAGGATACCAGTACTGCCGCTACCGGGTTATTGCCGCTTCTGACTCCGTGGTTGTCGGCAATGA
CGGGATATCTTAGTTCTATCGCGAAACAGATCGACGAGGGTGACTATGCCACACAGGGATCTAA
CCTGGGAATGCAATTGGCCGGAATGGAAAACATAATCAGAGCTGGCGAGGAGCAGGGGGTCTC
CTCACAGATGATCCTTCCGATAAAAGCGTTGATAGAGCAAGCAGTGGGTGAAGGGCATGGGGGT
GAGGATCTATCAGCGCTGATTGAATACTTTGAGGCGGGAAAAAATGTGGATTAA

>RedE
ATGGGCGCGAAAGTAACCGTCCTGGGTCTCGGCCCGATGGGCGCGGCCCTGGCCGGAGCGTT
CCTCGCGGCTGGTCATCGTACGACCGTTTGGAACCGTACGCCGGGTAAAGGTGGCTCTCTGGC
TGGTGAAGGGGCAACCGAAGTTGCGAGCGCCGCAGAAGCGGTGGCAGCGAGCCCGCTGGTCG
TAGTCTGTTTGGCAACGTACGAAGCAGTACATGAGGTACTCGACCCTCTTGCTGATGAACTGGC
CGGTCGCACTGTTGTCAACCTTACCTCGGGTTCCCCGGTCCACGCCCGTGAGACCGCCAACTG
GGCTCAGCAACACGGCGCCGAATATCTGGACGGTGTTATTATGACGACGCCAAGTGGAATTGGG
AAGCCGGATTACCTGCTGCTGTATAGTGGCAGCCAGGCAGCGTTCGATGGCTCACGTGGTACG
CTGTGTGCATTGGGCGAACCAATGAATCTGGGTACTGATGCAGCGATGGCCAGCGTGTACGATA
CCGCATTACTCGGCTTAATGTGGGGTACTCTGACGGGCTGGCTTCATGGTGTGGCCTTGATGGG
TGCCGATGGGCCAGGCGGTAATGTCACTGCGACTGCGTTCACCGAAGTCGCTAATCGCTGGAT
GAAGACGGTGGGTGTGTTTATGAACACTTACGCACCCCATGTAGACGCGGGCCACTATCCCGGG
GATGAATTTACCTTACATCTGCATCATCGGACTATGAACATTCTGGCCCACGCATCGGAACTGCG
CGGTGTAGTCAGCGGTTTACCGGAGTTACTGACCGAGTTGACGGGGCGCGCAATCACGGCGGG
TCACGGCAATGACTCTTATGCGCGGCTGGTCGAATTCATTCGCAAAGATGGTAGTCCTATTTAA

>SeIRED
ATGAATAACGGCTTCGCTGCTCCTGTTACCGTCGTTGGACTCGGACCCATGGGGTGTGCGCTTG
CAGATGCGTTTCTCGCCGCTGGTCACCCGACTACAGTGTGGAATCGTTCGGCCCATAAGGCAGA
CCCGCTTGTGGCAAAGGGAGCCGTTCGTGCGGCTACCGCTGCTGAAGCACTCGCCGCCAGCGA
CTTAGTGGTGGTTTGTGTCGCGGATTATGCTGCGATGCGTGCAGCCCTCGATCGCACCGGCGCA
GAACTGAGTGGTAAAGTTCTCGTCAACCTCTGTTCCGGTACACCTCGCGAGGCCCGCGAGGCTC

TTACGTGGGCGACCGCGCACGGGGCTGGATATCTTGACGGTGCTATCATGGTGCCGGTTGAAG
TGATTGGTACCCCTAGTAGTGTTGTGTTTTATTCCGGTGCCCGTGAGCCATTTGACGCACACCGC
TCAACGTTAGATGCCCTGGGTGGGGTGCCTCGCTACCTGGGAGATGACGCTGGGCTGGCAGTA
TTACACAATACCGCGCTTTTGGGCTTGATGTGGGCTACGGTAAATGGGTTTCTTCACGCAGCCG
CGCTCGTGGAAAGCGCGGGTGTCGGAGTGGCTGACTTCGCCGAGACGGCAGTGGATTGGTTTC
TGCCCTCGGTTATTGGCGAGATCTTACGTGCTGAGGCCGCTCGCATTGACCGCGGTGAGTTCCC
TGGCGATGGCGGGACCCTTGCGATGTGTTTAACAGCAATTGAACATATTGTTCGTACCTCGCGC
GATGCTGGAATCAGCGACGAGGTGCCGAGCCAGTTGAAGACATTGGGCGATCGTGCCGTAGCG
GCAGGTCACGGCGACGAGAACTACATGTCACTTATCAAGGTACTGCGCGTGCCTTCGACTGGTG
CTCATCGTTAA

>*Ss*IRED
ATGAGTGAGAAGAAGCTGGCAGTTACCGTAGCAGGACTGGGGCCAATGGGTTCAGCCCTGGCC
GCGGCACTGCTTGATCACGGCCACGAAGTCACGGTATGGAATCGTAGTCCTGGTAAGGCTGCG
CCGTTAGTAGCACGCGGTGCCCGCGAAGCGGATGGCGTGGCTAGCGCAGTGAGCGCGTCTGA
AGTAACTGTGGTGTGCCTTGCCGACTACAACGCGTTATACTCGGCGTTACGCCCAGCAGAGGCG
GCTCTTCGTGGACGTGTGGTTGTGAATCTTAATAGCGGCACCCCCAAAGAGGCACACGAAGCAG
TCCGTTGGGCGGAAGACCGTGGTATCGGTTACTTGGACGGAGCTATTATGGTTCCACCCGCGAT
GGTCGGACGCCCAGGCAGCGTATTCCTGTATTCTGGTGCAGAGGATGTATTTGATGCACACAAG
GCGACCCTGGCGGTTCTGGGTGAGGCAACGTACCTTGGTGCAGACCCAGGCCTTGCCGTACTT
TACAATACGGCACTTCTGTCTTTGATGTACAGCAGTATGAACGGCTTTCTGCACGCTGCGGCGCT
GGTTGGCTCGGCGGGAGTTGCAGCGACGGACTTCACAAAGCTGGCTGTTGACTGGTTCTTACCT
AGTGTGGTCGGCGGCATTTTAGAAGTTGAGGCTCCAGCGATCGACAATGGTGTTTACCCTGGTG
ATTTAGGCTCATTAGAGATGAACTTAACGGCCTTGACCCATATTGTAGGTACATCCGGCGAACAA
GGCGTAGACACGGAAATTCCTGCCCGCAATAAAGAGTTAGCGGAGCGTGCAATTGCGGCCGGT
TTCGGTAAGTCTAGCTACTCTTCAATTATCGAAGTGCTGAAGAAATAA
>*Ar*IRED
ATGAGTGAGAAGAAGCTGGCAGTTACCGTAGCAGGACTGGGGCCAATGGGTTCAGCCCTGGCC
GCGGCACTGCTTGATCACGGCCACGAAGTCACGGTATGGAATCGTAGTCCTGGTAAGGCTGCG
CCGTTAGTAGCACGCGGTGCCCGCGAAGCGGATGGCGTGGCTAGCGCAGTGAGCGCGTCTGA
AGTAACTGTGGTGTGCCTTGCCGACTACAACGCGTTATACTCGGCGTTACGCCCAGCAGAGGCG
GCTCTTCGTGGACGTGTGGTTGTGAATCTTAATAGCGGCACCCCCAAAGAGGCACACGAAGCAG
TCCGTTGGGCGGAAGACCGTGGTATCGGTTACTTGGACGGAGCTATTATGGTTCCACCCGCGAT
GGTCGGACGCCCAGGCAGCGTATTCCTGTATTCTGGTGCAGAGGATGTATTTGATGCACACAAG
GCGACCCTGGCGGTTCTGGGTGAGGCAACGTACCTTGGTGCAGACCCAGGCCTTGCCGTACTT
TACAATACGGCACTTCTGTCTTTGATGTACAGCAGTATGAACGGCTTTCTGCACGCTGCGGCGCT
GGTTGGCTCGGCGGGAGTTGCAGCGACGGACTTCACAAAGCTGGCTGTTGACTGGTTCTTACCT
AGTGTGGTCGGCGGCATTTTAGAAGTTGAGGCTCCAGCGATCGACAATGGTGTTTACCCTGGTG
ATTTAGGCTCATTAGAGATGAACTTAACGGCCTTGACCCATATTGTAGGTACATCCGGCGAACAA
GGCGTAGACACGGAAATTCCTGCCCGCAATAAAGAGTTAGCGGAGCGTGCAATTGCGGCCGGT
TTCGGTAAGTCTAGCTACTCTTCAATTATCGAAGTGCTGAAGAAATAA

>*Ps*DRR
ATGGAATCAAATGGAGTACCCATGATAACACTATCCAGCGGTATCCGCATGCCGGCTCTGGGTA
TGGGTACTGTTGAAACCATGGAGAAGGGCACTGAACGCGAGAAGCTCGCGTTTCTGAAGGCGAT
CGAGGTGGGCTATCGTCACTTCGATACCGCGGCCGCCTACCAGACCGAAGAGTGCCTTGGCGA
GGCGATCGCTGAAGCTCTGCAACTGGGTCTGATCAAAAGCCGTGAAGAGCTGTTCATCGCGTCG
AAGTTGTGGTGTACCGATGCGCACGCAGACCTGGTGCTGCCGGCACTGCAGAATTCTCTGCGCA
ATCTGAAGCTGGAATACTTGGACCTGTATTTGATTCATTTTCCGGTGTCCCTTAAGCCGGGTCGT
ATTGTTAGCGATATTCCAAAAGACCAGATGCTGCCTATGGACTACAAATCTGTTTGGGCTGCTAT
GGAGGAGTGCCAGACGCTGGGCTTTACCCGTGCAATTGGCGTTTCTAATTTCAGCTGCAAGAAA
TTGCAAGAGCTGATGGCTACCGCGAACAGCCATCCGGTTGTTAACGAAGTGGAAATGTCCCCGG
TCTTTCAGCAAAAAAACTTGCGTGCGTACTGCAAGGCCAACAACATTATGATTACCGCATACAGC
GTTTTAGGCGCGCGTGGTGCGGCGTGGGGTAGCAACGCAGTAATGGACAGTAAAGTGCTGCAC
GAAATCGCGGTCGCCAGAGGCAAAAGCGTGGCACAGGCATCCATGCGTTGGGTTTATCAACAAG
GTGCGTGTTTGGTCGTGAAATCGTTCAACGAGGAGCGCATGAAAGAAAATTTGAAAATCTTCGAT
TGGGAATTAAGCGCGGAAGATATGGAGAAGATCAGCGAAATCCCGCAGTGCCGTACCTCAAGCG
CTGACTTCCTGCTGAGCCCGACGGGTCCGTTTAAAACGGAGGAGGAGTTCTGGGATGAAAAGGA
CTAA

>BsP5
ATGAAGATAACATATATTGATAAACCCACTTACTTGCCAAGCTGGGTCATCAACAAGATTAACGAA
TATGGTGATTTCGAGGTATTCTACGACTTCCCGAACGAAGAAGAGGCGATTAATAGACTGTCGAG
CACCGACATCGCGATTGTTGAATGGACCAGCATCACGAAAGAAATGATTGAGAAGATCAGTCGT
CTGAAGTACCTGATAACCATTACCACCAGCTACGATTATATCGACGTGAACAGCCTAAAGGACAA
CGAAATCATGGTTAGCAACTGCCCGCAGTATTCCAAACAGGCTGTGGCGGAGCACGTGTTTGCA
CTGTTGTTTGCGGTGAATCGCAAAATCCTGCAGGCCGATGAGACGTGCCGTAAAGGTTTGTCCC
ATATCTACCCGCCTTTTCTGTGTAGCGAGATTCGTGATAAAACCATTGGTCTTATCGGCATTGGTC
AGATTGGCCAAACTGTTGCCGAAATCGCTAATGCGTTCCAAATGAAAGTTATTGGTCTCAACAAG
TCCAAGCGCAACGTGAAAGGTATTCAACAGGTTGATATCACGGAGCTGATGAAAAAGTCCGACAT
CATCAGCTTGCACATTCCGCGTAACGCTGACACCGAAATTATCTTGACCGAGAAGCTGCTTTCTC
TGATGAAGCCGGACGCGGTGCTGATTAACACCTGCCGTGGCAATCTGATCGACGAGCAGGCAC
TCTATAGCGTTCTGAAGCAAAACCGCATCCGTGGCGCGGGCTTAGATGATCTGACCTACTACAAA
GACAACCCGATTATCGGCCTGAATAATGTCGTTCTGACACCGGGTTCGGCATGGTATTCTTAA

>*Pp*DPKA
ATGAGTGCTCCCTCTACATCAACTGTAGTTCGCGTTCCGTTCACCGAACTGCAAAGCCTGCTACA
AGCTATTTTTCAGCGTCATGGTTGTTCGGAAGCGGTTGCGCGCGTGCTGGCGCACAACTGCGCG
TCTGCCCAACGCGATGGAGCGCACAGCCACGGCGTGTTTCGTATGCCGGGCTACGTTTCCACC
CTGGCAAGCGGTTGGGTTGACGGCCAGGCAACGCCGCAGGTTAGCGACGTGGCTGCTGGCTAC
GTGCGCGTCGACGCTGCAGGCGGCTTTGCACAGCCGGCGTTGGCAGCGGCGAGAGAATTGTTG
GTTGCCAAGGCGCGTTCTGCAGGCATCGCGGTGCTGGCGATCCACAACAGCCATCATTTCGCTG
CTCTGTGGCCAGATGTGGAGCCGTTCGCGGAGGAGGGTCTGGTGGCTTTGTCCGTGGTTAATA
GCATGACGTGCGTTGTCCCGCATGGTGCGCGTAAACCGCTCTTCGGTACAAATCCGATCGCCTT
TGCGGCTCCGTGCGCGGAACACGATCCGATTGTTTTCGACATGGCGACGAGCGCTATGGCACA
CGGCGATGTACAGATTGCGGCTCGCGCAGGTCAGCAGCTGCCAGAAGGTATGGGTGTGGACGC
CGATGGCCAACCGACCACCGATCCGAAAGCCATCCTGGAAGGCGGCGCGCTGCTGCCGTTCGG
CGGCCACAAGGGTTCCGCGCTTAGCATGATGGTTGAGTTACTGGCGGCGGCATTAACTGGTGG
CCATTTCTCTTGGGAATTTGACTGGTCCGGTCATCCGGGTGCCAAGACCCCGTGGACCGGTCAA
CTGATTATCGTGATCAACCCGGGTAAGGCAGAGGGCGAACGTTTTGCGCAACGTAGCCGTGAGT
TGGTCGAGCACATGCAGGCGGTTGGTCTGACCCGTATGCCTGGTGAGCGTCGTTATCGTGAGC
GCGAGGTGGCCGAAGAGGAGGGGGTTGCGGTCACCGAACAGGAGTTACAAGGTCTGAAAGAAT
TGCTGGGTTAA

>*Cr*THAS
ATGGCTATGGCAAGTAAATCACCCTCTGAAGAGGTTTATCCGGTTAAGGCATTCGGCCTTGCCG
CGAAGGATTCCAGCGGTCTGTTTAGCCCGTTTAATTTCAGCCGCCGTGCAACCGGTGAGCACGA
TGTTCAACTGAAGGTGCTGTATTGTGGTACGTGCCAGTATGATCGTGAAATGAGCAAAAACAAAT
TCGGTTTTACCAGCTATCCGTACGTGCTGGGCCACGAAATAGTTGGTGAGGTAACTGAAGTGGG
CTCCAAAGTGCAGAAATTCAAAGTCGGAGATAAAGTCGGTGTTGCTAGCATTATCGAAACCTGCG
GTAAGTGCGAAATGTGCACCAATGAAGTCGAGAACTACTGCCCGGAGGCTGGCTCCATCGACTC
AAATTACGGTGCGTGTAGCAACATCGCCGTTATCAACGAGAACTTCGTCATTCGTTGGCCCGAGA
ATCTGCCGTTAGACTCTGGCGTTCCGCTGCTGTGTGCAGGGATTACCGCTTACAGCCCGATGAA
GAGATACGGCTTAGACAAACCGGGTAAACGCATTGGCATCGCGGGCCTGGGCGGCCTGGGTCA
TGTTGCGCTGCGTTTTGCAAAAGCCTTCGGCGCGAAGGTGACCGTGATTTCTTCGTCCTTGAAG
AAAAAGCGCGAGGCGTTCGAAAAATTTGGTGCCGATTCTTTTCTGGTTAGCTCCAACCCGGAGG
AAATGCAAGGTGCTGCTGGTACTCTCGACGGCATCATTGATACCATTCCGGGTAACCACAGCCT
GGAACCGCTGTTGGCGCTGTTGAAGCCATTGGGTAAGCTGATTATCCTTGGTGCGCCAGAGATG
CCGTTTGAAGTGCCGGCACCGAGCTTGCTGATGGGTGGCAAGGTGATGGCGGCGTCGACCGCA
GGTAGCATGAAAGAAATCCAGGAGATGATTGAGTTCGCGGCGGAACATAACATCGTGGCGGATG
TTGAGGTTATCTCGATCGACTACGTCAATACGGCTATGGAGCGTCTGGACAACAGCGACGTGCG
TTATCGTTTCGTGATCGACATCGGCAATACCCTGAAGAGCAACTAA

>*As*IRED
ATGACAGATCAAAATCTACCCGTTACTGTAGCTGGCCTGGGTCCGATGGGTGCGGCGCTCGCTG
CGGCGTTGCTGGACAGAGGCCACGATGTTACCGTTTGGAACCGTAGCCCGGGCAAGGCTGCTC
CGCTGGTGGCAAAAGGTGCGCGTCAAGCGGACGACATCGTTGACGCGGTTAGCGCGAGCCGTC
TGTTGGTGGTGTGCCTGGCGGATTACGATGCACTCTACTCTGCCCTGGGCCCAGCGCGCGAGG
CGTTGCGTGGTCGTGTGGTGGTAAATCTGAACAGCGGTACACCGAAAGAGGCCCGTGAAGCAG
CTCAGTGGGCAGAAGGTCACGGCATCGGTTACCTGGACGGCGCGATTATGGTTCCGCCACCTC

TCGTCGGTCACACTGGTTCCTTGTTTCTGTACAGCGGTTCCACCGAGATCTTTGAAACCCATAAA
GAAACTCTGGCGGACCTGGGTGATCCGGCGCATCTGGGCACGGATCCGGGTCTGGCGGTGCT
GTATAACACCGCATTGTTAAGCATGATGTATTCTAGCCTGAACGGCTTCTTGCATGCGGCCGCTC
TGGTGGGCTCTGCGGGTGTTGGTGCGGCAGAGTTCACCGAGATTGCAGTCGACTGGTTCCTGC
CGTCGGTCATCGGCGGTATCATCAAAGCCGAGGCTCCGACCATTGATAAGGGCGAATATCCGG
GTGAACTGGCGAGTTTGGAAATGAATGTTACGACCTTGAAGCACATTATCGGCACCAGCGATGA
ACAGCGTGTTGACGCTGGCATTCCGGCAGGAAACAAAGAGCTGCTGGACCGCGCAGTGGCCGC
GGGCTTTGGTAAGAGCGGCTACTCCTCCGTGATTGAGGTTCTTAAGCGCGGTGCGGCTTAA

>*Ec*SaRed
ATGCGCTGATAGTTCAAAAAAGTTAACAGTACTACTGAGCGGCGCATCCGGCTTGACTGGTTCTC
TGGCCTTCAAAAAACTGAAAGAACGTAGTGATAAATTCGAGGTACGCGGTTTAGTTAGATCCGAG
GCCAGCAAGCAGAAGCTGGGTGGCGGCGATGAGATCTTTATCGGCGACATCAGCGACCCAAAA
ACCCTGGAACCGGCAATGGAAGGTATTGACGCATTGATCATCCTGACCAGCGCAATTCCGCGTA
TGAAACCAACGGAGGAATTCACCGCGGAGATGATCAGCGGTGGCCGTAGCGAAGATGTCATCG
ACGCGTCGTTCTCCGGTCCGATGCCGGAGTTCTATTACGACGAGGGCCAATACCCGGAGCAAGT
CGATTGGATTGGTCAAAAGAACCAGATTGACACCGCGAAAAAGATGGGTGTTAAGCACATTGTTT
TGGTTGGTAGCATGGGTGGTTGTGATCCGGATCATTTTCTGAATCACATGGGCAATGGCAACATT
CTGATCTGGAAACGTAAAGCTGAACAGTACCTGGCGGACTCTGGCGTGCCGTATACCATTATCC
GCGCGGGTGGCCTGGATAACAAGGCTGGTGGTGTGCGCGAACTGTTAGTTGCTAAGGACGACG
TTCTGCTTCCGACGGAGAACGGCTTCATCGCCCGTGCGGACGTGGCAGAGGCGTGCGTTCAGG
CGTTGGAAATTGAAGAGGTGAAAAATAAGGCGTTCGATCTCGGCTCCAAGCCGGAAGGTGTGGG
TGAAGCGACCAAAGATTTTAAGGCGTTGTTTAGCCAGGTGACCACCCCGTTTTAA

>*Cg*EasG
ATGCAATTTTACTTACAGGGGGGAATGGCAAAACAGCTCGGCATATAGCCAGACTGCTGAAAGA
GGCTGATGTTCCATTTATAATCGGCTCTCGTTCTTCAACCTCGGAGATGATCGGTCACCACCGGA
GCTTTGACTGGCTGGACGAAGCGACCTTTGGGAACACACTTTCTGTCCATGGCGGCATGGAACC
GATCTCTATAGTTTGGCTGGTGCCCCCGCCGATCCTGGACCTGGCACCCCCAGTGATCTCATTT
ATTAACTTCGCAAATTCTAAAGGCGTGAAGCGTTTCGTACTTCTTTCGGGTAGTATTATTGATAAA
GGGGGTCCAGCCATGGGCCAGATTCATGCTCATCTGGATTCCCTGGAAGGAATCTCATATTCGG
TTCTTAGACCAACGTGGTTCATGGAAAATTTTTCAACACGTGGTGATTTACCCTTTGAAACAATTC
GGAAGGAAGGCAAATTCTACAGTGCCACGAAAGATGGCAAGATACCATTAATTTCAGTTATGGAT
ATTGCGCGTGTCGCTTTTCACGCACTGACCGCACCGGTGTTAGAAAAAGAGGAGCATATATTACT
GGGGCCTGAACTGTTGACGTATGACGATGTCGCAGAAACGCTGAGCCGCGTACTCGGGCGTAA
TATCATTCACGCCCGTGTAACGGAGAGTGAGCTGGCAGAAAAGCTGCAAGACAACGGTCTTACA
GCAGAAGATGCAGCAATGCAAGCATCTTTGGATCTTATTATCTCAGCAGGCGGCGATGAACGCC
TGAACACCGAGGTTCGTGACCTTACAGGCCAAGAGCCGCGACGTTTTAGTGACTTTGTGTCTGA
AAATAAGCATGCATGGATTTGCTAA

>*Pa*PchG
ATGCGGATGTGAGATCGGTAGTTGTCGCTGGATCGAGATTTGGCCAATTTTATGCCGCCGGTGT
GGCAGCCGATCCTCGCTTTGTTTTACGCGGGATCTTAGGACAGGGATCAAGACGTTCGCGAGCA
CTCGCGGAACGGCTTGGGGTTGAAACCTGGTGTGAAGTAGAAGCGTTACCGGATGACGTAAGA
CTGGCCTGCGTAGCGGTCGGAGGCGCTGCACGGGGGGAGCAGGGTCCGGCGCTGGCTGAAG
CTCTGATGGCACGCGGAATTGACGTCCTGATCGAACATCCCCTGCTGCCGCGAGAATGGCAAGA
TCTGTTACGATCTGCGGAAAGATTAGGCAGACGATGTCTTTTGAACACATTTTATCCCCAGCTGC
CAGCTGTCGCCCGGTTTATTGAACTTGGCCGTCAGTTACATCATCGGCGTGGCATTCGTCATCTC
GACGCCGCCTGTGGCGTGCAGGTTGGCTTTGCCACGCTTGACATTCTGGCAGCCTTGCTGGAG
GGTGTTGGTCCTTGGAGTCTTGAAAGCCCGTCCAATGATCTCAGCGCTATGCGTGAATTGTCCCT
TGTATTGGCCGAAGTGCCGCTGTCGCTGCACGTATTAAATGAACTGGCCGCAGCAGATGACGGT
CGCATGACGCTCCTCCAGCGTGTGTCCCTTACCACCGACCGGGGTACACTTAGCCTTCTTTCGC
CACATGGCCCCCTGTTGTGGACTCCGGCTGTCGCCGTGCCTGCGGAGGATGATGATGGCTTATT
TGCCCTGTTCGATGAAATCGCCGGAGAACCGTTGCCGTCCGCTCAATTGTGGTATGCCGAACCA
TGTAGTTGGGCTCAGGTGCATCAACGGCTGTGGCCAGCAGCAGCGGCCGAAGCACTGGCTCTG
CTGGCCGATGGAGATGAAGTGCGGCGACGCAATCAACGCTCTTTAGAAGTTGCCGCTCTGTGGC
AGCGCATCGGCGAGCGGCTTGGCTTCCCCGAAGCTCCCCCGGCATCACTGGCACCGGCTAGTT
TGGAGCAGGTTCTGGAGCAGGCGAGCTAA

>*Cp*IM1
ATGAAGGTCATTAGAGATAAAGATATTAAATCATTTCTTAACAAAAGGTTAACAAGGGAGTCCATT
TTTAGTCAATTCCAACCGGTATTGTTGCGTGGTTTAGCCACTTACGCAGCTAACCCAAATGCAAT
CGTTCCTCCAAGAATTGTCCAACAGTCGAATAATTCTGAGTCAGACACTACCCATGTGTTCATGC
CATGTATTTCACCTACCGAAGTGGGCATAAAAGTGATTAGTGGTGGTCCATCGAACAATACAAAG
GGGTTGGGGTTTCAAGGGTGTGTTATGATTTTGGATGAAGTAACTGGTGAATTGAATGCAATCTT
CAATGCCGCATGTTTAACTGCTTTCAGAACTGCATTAGCTAGTGTATTGGGTCTTACCAGAGTTGT
GCCTGTTGACTCTGTCGACGTTTTGCCGGAATTGTGTGTGTTTGGGGTAGGTCAACAAGCTTATT
GGCATGTCAAGTTGACTTTACTCTTGTATAAGGAGAAAATCGCGAAGGTGAACATACTAAATAGA
ACATTGGCAAATGCAGAAAAGTTGAAAGAGGAGTTGGGTAAAGAATTTGACAATGTTGAGTTCAG
AGCGTTCCTGTTTGAAGAGGATGAAAAGTTCAAACCGCATATGGAAAACAGTTCAATCATATACG
GATGCACTCCATCGACTTCAGCTGTGATAAAGAAAGACCATTTGAACAAAGATCCAAAATATCGA
AAGTTTATTTCTCTCATCGGTTCGTACAAGCCGCACATGATTGAGTTGGATTTGGAATTGATGAAT
GATTTCAAAAACAATGGCGTCAAAGTGATTGTTGACTCAAAGGAGCATACGTTACATGAAGCTGG
AGAATTGATACAATCAGGTTACACTAGTGACCAGTTGATTGAGATTCACGAATTGTATGAAACGG
AAGAGTTCAGTACGATTACAGATGCAACAACCGGTACAACCGTACAAAAGATTGTAGGATTATCA
ATCATGGACTTGTGCATGGGGAAGTACATTTATGAAAACATCCAAGACGATGATGCAGTTGTTGT
AAATGACTTTTAG

>*Pj*DHFR
AACCAGCAAAAGTCCCTCACCCTTATCGTGGCGCTTACGACGTCGTATGGCATCGGCCGTAGCA
ATAGTCTGCCATGGAAATTGAAGAAGGAAATCTCGTATTTTAAGCGCGTAACATCATTCGTCCCG
ACGTTTGATAGCTTTGAATCGATGAATGTCGTGCTCATGGGTCGTAAAACGTGGGAGTCAATTCC
GCTGCAGTTTCGTCCGCTGAAAGGTCGTATTAACGTTGTAATTACTCGTAATGAAAGCCTGGATT
TAGGTAACGGGATCCACTCGGCCAAAAGCCTGGATCATGCGCTGGAACTGCTGTACCGCACGTA
CGGTTCAGAGAGCAGCGTGCAAATCAACCGCATTTTTGTCATCGGCGGTGCCCAGTTATACAAA
GCCGCTATGGATCATCCGAAGTTGGATCGCATCATGGCCACAATTATTTACAAAGACATTCACTG
TGACGTCTTCTTCCCGTTGAAGTTTCGTGACAAAGAATGGAGCAGCGTCTGGAAAAAAGAAAAAC
ACTCGGATCTGGAGAGCTGGGTCGGTACTAAAGTGCCACATGGCAAAATTAACGAAGACGGTTT
CGATTATGAATTTGAGATGTGGACTCGTGACCTCTAA

>*Gg*DHFR
ATGGTGCGGTCCCTGAATAGTATCGTAGCAGTCTGTCAAAATATGGGTATTGGCAAAGACGGGA
ATCTGCCGTGGCCGCCGCTTCGTAACGAGTATAAATATTTTCAACGGATGACATCAACATCTCAC
GTGGAAGGAAAACAGAATGCCGTGATAATGGGCAAGAAAACCTGGTTTTCAATCCCGGAAAAAA
ATCGTCCCTTAAAAGATCGAATCAATATCGTGCTGTCCAGAGAATTGAAAGAAGCCCCGAAAGGT
GCACACTATCTGTCCAAAAGTCTGGACGACGCGTTAGCTTTATTGGACAGCCCAGAACTGAAATC
GAAAGTAGACATGGTGTGGATCGTAGGTGGCACCGCCGTTTACAAGGCGGCAATGGAGAAACC
GATAAACCATCGACTGTTTGTGACCAGAATCCTGCATGAATTTGAATCGGATACGTTCTTCCCAG
AAATAGATTATAAGGACTTCAAACTTCTGACCGAATACCCTGGAGTCCCTGCGGATATCCAGGAG
GAGAATGGTATTCAGTATAAATTTGAGGTCTATCAGAAATCTGTACTTGCACAATAA

>NAPW
ATGGAGAGAACACCCGATACGCCGGCTCCGGACTTACGCGGTAAGATTGCCCTGGTTGCTGGC
GCGACAAGAGGTGCAGGACGTGCGATAGCAGTTCAACTCGGCGCCGCGGGAGCTACAGTCTAC
GTAACTGGAAGAACTACCCGGGAACGTCGTTCAGAATACAATCGTAGTGAAACAATAGAGGAAA
CCGCAGAGTTGGTTACAGAGGCTGGTGGCACAGGTATTGCAGTCCCGACCGACCATCTGGTTCC
GGAGCAAGTGCGGGCCCTGGCTGATCGCGTGGACACTGAGCAAGGACGTCTCGATGTCTTAGT
AAACGATGTTTGGGGTGGTGAACGTCTGTTCGAATTCGACAAGAAAGTGTGGGAACATGATCTG
GACGCAGGGCTGCGTCTGATGCGTCTGGGAGTCGACACTCATGCAATTTCAAGCCACTTCTTGC
TGCCGCTCTTGGTGCGGCGTCCTGGTGGCTTAGTGGTTGAAATGACCGACGGAACGGCGGCAT
ACAATGGCAGTCATTATAGAAACTCCTATTTCTACGATCTTGTTAAGAACTCGGTATTACGTATGG
GCTACGTTCTGGCCCATGAGTTGGAGCCGTATGGCGGTACCGCTGTAACCTTAACTCCTGGATG
GATGCGTTCGGAGATGATGCTCGAAACATTAGGCGTTACTGAGGAGAACTGGAGAGACGCTTTG
ACGGAAGTCCCACATTTCTGTATTTCCGAGAGCCCATCGTACGTAGGACGTGCTGTAGCTGCTCT
CGCTGGTGACGCTGATGTTGCCAGATGGAATGGCCAATCCGTCAGCTCGGGTCAGCTCGCGCA
AGAATATGGCTTTACGGACCTGGACGGTAGCCGTCCCGATTGTTGGCGCTACCTTGTCGAGGTC
CAAGAGGCAGGTAAGCCTGCCGATCCGTCCGGTTACCGGTAA

>*Pb*SDR
ATGAAGCCTCTGCGTGGTAAAGTGGCTTTGGTGGCTGGCGCTACACGTGGTGCTGGTCGGGGC
ATAGCCGTTGAGCTTGGTGCCGCAGGTGCCACCGTGTATGTTACGGGCCGCACAACACGGCAG
CAGCGTAGTGAATATAACCGCCCAGAGACGATTGAAGAAACTGCAGAACTCGTTACCGCGGCAG
GCGGTCAGGGCATAGCCGTTCAGGTTGACCATTTACAGCCCGAACAGGTACAGGCTCTCATTGC
CCGAATTGAGAAGGAACAAGGCAGACTGGACGTTCTGGTTAATGACGTTTGGGGGGCGGAAAAT
CTTGCAGATTGGAATGTCCCTGTGTGGGATCATTCCCTTGAACGTGGATTCCGTATGCTTCGCTT
AGGCATCGATACGCACTTAATAACCTCCCATTTCGCGTTGCCCTTGCTTATTCGTAACAAGAATG
GCTTAGTAGTTGAGATGACAGATGGTACAGCGGAGTACAACTATAAAAACTACCGTATAAGCATG
TTTTATGATCTGGTCAAAAACTCCGTAATTCGCATGGCTCAAAGCCTGGCACATGAACTGGCGCC
ATATCAGTGTACTGCCGTGGCTATGACACCCGGTTGGATGCGGTCAGAGATTATGCTGGATCATT
TCGGAGTCAAAGAAGAAAATTGGCGCGACGCAGCGGAAAAGGAACCGCACTTTATCATTTCGGA
GTCCCCCCGTTACGTCGGTCGAGCGGTAGCAGCATTAGCTGGTGACCCCGAAGCTGCTAGATG
GAATGGAAAATCTCTGTCAAGTGGGCAGTTGGCTAAAGTCTATGGATTCACAGATCTTGATGGTT
CACAACCAGATTGCTGGCGCTACCTGGTCGAGGTACAGGAAGCTGGGAAGCCTGCAGATGCCA
GCGGCTATCGATAA

>*Mb*SDR
ATGAATGAAGATAGACGTCCTCTGGAAGGTAAGATCGCGTTGGTTGCCGGTGCTACCCGCGGTG
CCGGTCGTGGAATAGCAATCGAACTCGGTGCGGCGGGTGCAACGGTCTATTGTAGTGGGCGTT
CAAGCCGCGCAGATGTAGCGGGCCGTCGTGCGCCCGATGCACGTCCGTTTGAATTGTCTGGCC
GTCCCGAAACGATCGAGGAAACCGCAGAGCTGGTCACCGCAGCAGGGGGCACGGGTATTGCAA
TGCGCACCGATCATTTGGATGAAGATGCAGTAGCAGCGCTCGTTAAAAGAATCCGTGATGAACAT
GGGAGACTGGACGTCCTGGTGAACGATGTTTGGGGGGGAGATGCCCTGACTGAATGGGGAAAA
CCCTTTTGGGAGTTAGATCTGGAGCAGGGTCGAGTATTGCTTGATCGTGCCATCCGGACCCATG
TGGTTACAAGCCGTCATGCAGTACCCCTGCTGCTTGAACGACGCAGTCTTGAACGCCGGCTGAT
TGTTGAAATTACTGATGGGGATGCTATGTACTATCGCGGAAATTTTTTTTATGATATTGCGAAGAC
AACAGTCATTCGTCTGGCATTCGCAATGAGCGAGGAGTTACGCGAGCATGGAGTGGCGGCGGT
TGCCGTCACCCCGGGCTTCCTGCGCAGTGAAGCGATGCTGGAGCACTTTGGAGTAACTACAGA
GACTTGGCGTGACGGAGCGAAAAAAGACCCGCACTTTTTGTTCTCGGAAACACCGAGATTCGTG
GGTCGAGGAATTGCTGCACTGGCTTCTGATCCCGAGATCATGCGCCGCAGTGGAGGTCTCTTCT
CAAGTTGGCAACTGGCGGCTGAGTACGGGATAGATGACATTGACGGTACGAGACCCGATTGGG
GGAGCCATGCCGCCGGGTCTAGTTTTGCGGAAGAACATCGTGCTTCTCATGAGCGTTTTGTGCA
TGGAACGACCGCGCGGCATGCTGTTCGTGCTCCGGATGCCTCCGGCTCAGTTTAA

>*h*CRYM
ATGTCCAGAGTGCCGGCCTTCTTGAGCGCAGCTGAGGTAGAAGAACATTTGCGTTCCAGCTCAC
TTTTAATACCACCATTAGAGACGGCGTTGGCCAATTTCTCTTCTGGTCCTGAAGGTGGTGTGATG
CAGCCTGTCCGGACAGTGGTGCCCGTTACTAAGCACAGAGGATACTTAGGCGTCATGCCGGCAT
ATAGCGCGGCAGAGGATGCACTTACAACTAAATTGGTTACCTTTTACGAGGACCGTGGAATCAC
GTCTGTTGTCCCCTCTCATCAGGCGACTGTACTGCTGTTCGAACCCAGTAACGGCACTCTGCTTG
CCGTCATGGACGGCAACGTAATTACAGCAAAGCGGACAGCTGCTGTGTCGGCTATTGCGACCAA
GTTTCTCAAGCCACCTAGCTCTGAGGTACTGTGCATTCTTGGCGCCGGTGTGCAAGCGTACAGT
CACTATGAAATATTCACTGAACAGTTCAGCTTCAAGGAAGTCCGTATATGGAACCGAACCAAGGA
AAATGCTGAGAAATTTGCCGACACTGTGCAAGGTGAGGTGCGTGTGTGCAGTTCTGTCCAAGAG
GCCGTAGCCGGAGCAGACGTCATAATAACAGTGACTTTAGCAACCGAGCCGATACTGTTCGGTG
AGTGGGTCAAACCCGGCGCGCACATCAACGCCGTCGGGGCGTCCCGTCCTGATTGGCGTGAGT
TAGACGACGAGCTGATGAAGGAAGCAGTCCTGTATGTCGACAGCCAGGAAGCAGCCTTGAAGG
AGTCAGGTGATGTTCTGCTCTCAGGTGCCGAAATCTTTGCCGAGCTTGGTGAGGTTATAAAAGGA
GTTAAGCCCGCTCACTGCGAAAAGACAACTGTATTCAAAAGTCTGGGAATGGCGGTAGAGGACA
CGGTAGCAGCTAAATTAATATACGACAGCTGGAGCTCCGGGAAGTAA

>*Bt*CRYM
ATGTCATCTCGTCCTGTGTTTCTTAGCGCGGCAGACGTACAAGATCACCTGCGTAGCTCTTCATT
GCTGATCGCGCCCCTTGAGACTGCCTTGGCAAACTTTAGTTCTGGGCCTGATGGGGGTGTGGTG
CAGCCCGTTCGCACTGTTGTTCCTGTCGCAAAGCATCGTGGTTTTTTGGGCGTGATGCCGGCAT
ACTCGGCGGCAGAGGATGCACTGACTACCAAACTTGTCACTTTTTATGAAGACCACAGCGCAACT
TCTACGGTCCCCTCCCATCAGGCTACGGTACTGCTGTTTCAGCCGAGCAATGGGTCCCTGCTTG
CAGTGATGGATGGTAATGTTATAACTGCTAAACGTACCGCAGCCGTATCAGCCATCGCGACTAAA
TTTCTTAAACCTCCGAATTCCGAAGTACTGTGTATCCTGGGCGCTGGGGTCCAAGCTTATTCCCA

TTATGAAGTTTTTACTGAGCAATTTTTTTTTAAAGAAGTACGTATCTGGAACCGTACAAAAGAGAAT
GCAGAGAAATTTGTGAATACAGTTCCTGGAGAGGTTCGTATATGTTCGTCTGTTCAAGAAGCAGT
AACGGGTGCCGATGTAATAATCACCGTCACTATGGCGACCGAGCCAATATTGTTTGGAGAATGG
GTGAAACCGGGCGCTCACATCAACGCAATTGGCGCGTCGCGTCCAGATTGGCGAGAGCTGGAC
GACGAACTGATGAAACAGGCAGTGTTATATGTGGATAGCCAGGAAGCGGCTCTGAAAGAATCAG
GCGACGTACTGCTTTCAGGCGCCGAAATTTTTGCAGAGCTTGGAGAGGTTGTAAAGGGTGTAAA
GCCCGCGCACTGTGAAAAGACAACCGTTTTTAAAAGCCTGGGCATGGCCGTGGAAGATATGGTG
GCTGCCAAACTGGTCTATGATAGTTGGTCATCTGGGAAATAA

>*Tb*DHFR
ATGGTCTTTGACGCGCATTCTGCGTAAAAAAATCCCTGTACATGAACTGGCAGGTAAAATTTCTC
GCCCTCCCTTACGGCCTTTCAGCGTCGTAGTTGCCAGTGATGAGAAGGGGGGTATCGGTGATG
GAGGAACGATTCCATGGGAAATACCGGAAGATATGCAGTATTTCAGACGCGTAACCACGAACCT
GCGAGGGAAAAACGTGAAACCTTCTCCTAGCAAGCGTAACGCCGTAGTTATGGGTCGGAAAACT
TGGGATAGCTTGCCACCAAAATTTCGTCCCTTATCTAACCGGCTGAATGTGGTTCTGAGCCGGAG
TGCGACCAAAGAACAGCTGCTGGCTGGGATTCCCGACCCAATAAAACGTGCCGAAGCAGCCAAT
GATGTTGTCGCGGTTAATGGCGGTTTAGAAGACGCCCTGCGTATGTTGGTTTCTAAAGAACATAC
GTCTTCCATAGAAACAGTTTTCTGTATCGGTGGTGGGACGATTTATAAACAGGCCCTTTGTGCGC
CTTGCGTGAATGTGCTGCAGGCTATTCATCGTACCGTCGTCAGACCAGCTAGCAATTCTTGTTCC
GTGTTCTTTGATATTCCGGCGGCGGGGACGAAAACACCTGAAGGCCTGGAACTCGTTCGCGAGT
CGATCACAGATGAGCGGGTCAGCACGGGCGCGGGAGGTAAAAAGTATCAGTTCGAGAAGTTAG
TCCCGCGTAACAGTTAA

>*Tb*DHFR-TS
ATGCTTTCACTTACTCGGATTCTGCGCAAAAAAATTCCAGTGCATGAACTTGCGGGCAAAATATCT
CGGCCGCCGCTTCGCCCATTCTCGGTGGTAGTGGCCAGCGACGAGAAAGGGGGCATTGGCGAT
GGCGGTACTATCCCGTGGGAAATTCCGGAAGATATGCAATATTTTCGCCGCGTAACCACGAATCT
CAGAGGCAAAAATGTTAAGCCTAGTCCAAGCAAGCGGAATGCAGTAGTAATGGGCAGAAAAACG
TGGGATAGCCTTCCGCCAAAGTTCCGTCCCCTGTCAAACCGGCTGAACGTTGTACTTAGCCGCT
CTGCCACCAAGGAACAATTACTGGCCGGTATACCGGATCCTATCAAAAGAGCCGAAGCCGCTAA
TGATGTTGTGGCGGTTAATGGTGGGTTAGAAGATGCCTTGCGCATGCTTGTTAGTAAAGAACATA
CATCATCTATTGAAACAGTGTTTTGCATAGGAGGAGGGACAATATATAAGCAGGCCCTTTGTGCG
CCGTGCGTTAATGTGTTACAGGCTATCCATCGCACAGTCGTACGTCCCGCCTCTAATAGCTGCTC
TGTCTTCTTTGATATCCCTGCGGCAGGGACCAAAACTCCAGAAGGTTTAGAACTGGTTCGCGAAA
GTATTACAGACGAACGGGTTTCAACAGGCGCGGGTGGAAAAAGTATCAATTTGAGAAACTGGT
GCCTCGTAATTCTGAAGAGGAACAGTATCTGAATCTGGTGGGCCGAATTATAGATGAAGGGTGC
ACCAAATGTGATCGCACTGGTGTTGGGACCCGTAGCCTTTTTGGTGCTCAGATGCGCTTTAGCCT
TCGCAATAATCGCCTTCCCCTGCTTACGACCAAACGCGTGTTTTGGCGTGGTGTTTGTGAAGAAC
TGCTCTGGTTCTTACGTGGTGAAACTAACGCGAAACTGCTTAGCGATAAAGGTATTCACATATGG
GATGGGAATGGCTCTCGTGCGTTTCTTGATAGCCGAGGTTTAACGGATTACGACGAAATGGATTT
AGGTCCTGTCTATGGTTTTCAATGGCGTCACTTTGGTGCCGATTATATTAGCTGCAAAGTGGATA
GTGAGGGCAAAGGCGTTGATCAAATCGCAAATATCGTTAAGTCTCTGATTGAAAACCCGGATGAT
CGTCGGATGATTTGCACAGCATGGAATCCCGCCGCACTTCCGAGAATGGCTCTCCCACCATGTC
ATATGATGGCTCAGTTTTATGTCAGCAATGGCGAACTGAGTTGTATGTTGTACCAGCGCTCTTGC
GACATGGGCCTGGGTGTTCCATTCAATATAGCTTCGTATGCACTGTTGACTTTCCTGATGGCCAA
AGCCAGTGGTCTTCGCCCTGGGGAACTGGTGCATACTCTGGGGGATGCTCACGTATATAGCAAT
CATGTAGAACCATGCAGAAAACAACTTAAGCGCGTTCCGCGTCCATTTCCGTTCATCGTGTTTAA
GCAGGATAAAGAATTTCTGGAAGACTTCCAGGAATCTGATATTGAAGTAATCGATTATTCTCCATA
TCCTGTGATTTCTATGGAAATGGCAGTTTAA

>hDHFR
ATGGTGGGCTCACTTAACTGTATCGTCGCTGTGTCTCAAAACATGGGTATTGGCAAGAACGGGG
ACTTACCATGGCCACCGCTGCGTAACGAGTTCCGATATTTTCAACGTATGACGACGACGAGCTCT
GTTGAGGGCAAACAGAACCTCGTTATCATGGGCAAGAAGACCTGGTTCAGCATTCCGGAAAAGA
ACCGCCCTCTTAAGGGTCGCATCAACTTAGTACTCTCACGCGAGCTCAAGGAACCGCCACAGGG
TGCGCACTTCTTGTCCAGATCCTTGGACGATGCACTCAAGCTTACCGAGCAACCCGAGTTAGCTA
ACAAGGTGGACATGGTGTGGATCGTAGGTGGGAGTTCTGTTTACAAGGAAGCTATGAACCACCC
AGGACACCTGAAGCTGTTTGTGACCCGGATTATGCAAGACTTCGAATCAGATACATTCTTTCCGG
AAATAGATCTGGAGAAGTACAAGTTGCTTCCTGAATACCCCGGCGTGTTATCAGATGTGCAAGAG
GAGAAGGGGATCAAGTACAAATTTGAAGTATATGAGAAGAACGACTAA

>*At*Prua
ATGAAATGCCGGAGTGCGGTCAAGGAGCATATCCCCAAAACTGTGTTAATTACTGGTGCCGCTC
GGCGCTTGGGACGCGCAATCGCTTCAGATCTGGCCGCGCATGGTTTTGCGATTGCCGTGCATG
CAAATGAATCAATGGCTCAGGCAGAAGAATTTGCGAACGAAATTCGCCAGAAAGGGGGTCGTGC
CACGGCCGTCCAGGCCGATCTGACTCAATCCGCACCGACAATGGCATTAGTGGAAAAAGCTGCG
GCCGCCTTGGGACCTATCGGAGTTGTAGTGAATAATGCCTCGGTCTTCTTGGCAGACACAGCTG
AAACCCCCGATCCCGCTGTCTTCGATGCCCATTTTGCAGTTCATGTCCGGGCACCTAGTCTGATT
GCTGCCGCATTCGTAGAACAGTTGCCAGCTGAAAAGTCAGGGCTGATAGTGAACATTATTGATCA
GCGTGTGTTGGCGCTGACCCCACGCTTTTATAGTTATACCTTGTCAAAATCAACGTTGTGGACAG
CAACGCGCACCATGGCCCAGAGTTTCGCACCGCGGGTGCGGGTCAATGCCATCGGACCGGGC
CCGACCTTCAAATCCGAACGCCAGGCCCCTCAAGATTTTCAGGCACAAATTGATGGGTTAATTCT
GAAGAGAGGTCCTGCCCCGGACGAATTTGGGCGCACTATTCGATTTCTGTATGATACGCCAAGT
ATTACTGGCCAGATGATCGCCCTGGATGGGGGCCAACACCTGGGTTGGGAGACCCCTGACGTG
GCTGAAATCCCAGAGTAA

>*Pa*Prua
ATGGCGACAGCTCCGATTTTGATTACAGGGGCGAGCCAGCGGGTGGGTCTGCACTGCGCTCGC
CGGCTCCTCGCCGATGGCGAAAGCGTTATCGTATCTTATCGGAGTGAACGTCCGGCTCTGGATG
AGCTTCGTCAGGCAGGGGCCCTCACTCTGCATGCAGATTTCGCGTCTGAAGCGGGTATATTTGC
TTTTATTGGAGCACTTCGCCAGCATACGGATAGTTTACGTGCCATTGTACACAATGCATCCGACT
GGGTTGCAGAAACCCCAGGTCATGAAGCTGAAGCCTTCCAACAGCTTTTTTCTGTTCATATGTTG
GCCCCTTATCTGATTAATCTTCATTGTGCCGAACTGCTTGAGCGCTCGCAACCAGCAGATATAGT
ACATCTGACTGATGACGTTGCCCGCAAAGGAAGTGCGCGCCGGATTGCTTATTGTGCATCGAAA
GCGGGCCTGGATAATCTTACCTTATCATTCGCAGCTAGATTCGCGCCTCGTATTAAGGTGAACGC
AATTAGTCCGGCCCTGGTCATGTTTAACGACGGGGATGATGCCGAATATCGCGCCCGCACGCTC
GCCAAATCAGCACTGGGAATTGAACCAGGGCCGGAAGTGATTTATCAATCTCTCAGATACCTTCT
TGATAACCCATACGTTACGGGCACGACCCTGACAGTGAATGGCGGCCGGCACGTTAA

Supplemental References

1. Zallot, R., Oberg, N., and Gerlt, J.A. (2019). The EFI Web Resource for Genomic Enzymology Tools: Leveraging Protein, Genome, and Metagenome Databases to Discover Novel Enzymes and Metabolic Pathways. Biochemistry *58*, 4169–4182. https://doi.org/10.1021/acs.biochem.9b00735.

2. Shannon, P., Markiel, A., Ozier, O., Baliga, N.S., Wang, J.T., Ramage, D., Amin, N., Schwikowski, B., and Ideker, T. (2003). Cytoscape: A Software Environment for Integrated Models of Biomolecular Interaction Networks. *Genome Res.* ***13***, 2498–2504. https://doi.org/10.1101/gr.1239303.

3. Aleku, G.A., France, S.P., Man, H., Mangas-Sanchez, J., Montgomery, S.L., Sharma, M., Leipold, F., Hussain, S., Grogan, G., and Turner, N.J. (2017). A reductive aminase from *Aspergillus oryzae*. *Nat. Chem*. ***9***, 961–969. https://doi.org/10.1038/nchem.2782.
