# Supplementary material for: Expanding the repertoire of imine reductases by mining divergent biosynthetic pathways for promiscuous reactivity
Source: Chem Catal. 2024 Dec 19;4(12):101160. doi: 10.1016/j.checat.2024.101160 (PMC11876095; doi:10.1016/j.checat.2024.101160)
Supplement: Data S1. Chem Catalysis checklist [file mmc2.pdf]

Chem Catalysis checklists

Revision 1.1, Last updated: November 08, 2021

- *Chem Catalysis* has compiled these checklists to foster improved rigor and reproducibility in research and increased clarity and transparency in data reporting.
- Authors are encouraged to include the completed checklists as supplemental information at the time of submission. The checklists will be included in the supplemental information of published articles.
- Rather than check off all items on the list, authors should mark only those items that apply to their article.

The following checklists are relevant for this manuscript:

☒ **General catalysis checklist**

Please note: the general catalysis checklist should be completed for all submissions, including those with biocatalysts, electrochemistry, and photocatalysts

☒ **Biocatalysis checklist**

☐ **Electrochemistry checklist**

☐ **Photocatalysis checklist**

Sustainability remarks

☒ "Principles of green chemistry" have been considered in designing and conducting the research

For more information, please see https://www.acs.org/content/acs/en/greenchemistry/principles/12-principles-of-green-chemistry.html.

I verify that, to the best of my knowledge, this form is completed accurately in agreement with all co-authors ☒

Submitting author name: **Florian Hollfelder**

For general queries or feedback regarding this form, please email catalysis@cell.com

General catalysis checklist

Revision 1.1, Last updated: November 08, 2021

Catalyst synthesis

- [x] Novel methods are provided in full detail
- [x] Chemical vendor provided if catalyst was purchased
- [ ] Comprehensive literature references are included if the synthesis has been previously reported

Catalyst and new materials characterization

- [ ] Elemental analysis
- [ ] NMR spectroscopy
- [ ] High-resolution mass spectrometry (HRMS)
- [ ] Infrared spectroscopy
- [ ] Crystallography
- [ ] Phase and crystallinity
- [ ] Morphology
- [ ] Chemical composition of the catalyst
- [ ] Thickness analysis for two-dimensional materials
- [ ] Particle size and size distribution
- [ ] Characterization and analysis of pore size
- [ ] Exposed facets and orientation
- [ ] Defect structure
- [ ] Analysis of edge or vertex sites
- [ ] Analysis of valence state
- [x] Data are available in a repository

Catalyzed reaction

- [x] Reaction conditions and complete experimental procedure provided
- [ ] Size and type of reactor (e.g., flow, batch, semi-batch)
- [x] Operating temperature
- [ ] Operating pressure
- [x] Solvent
- [x] Catalyst loading (mass and/or concentration and reaction volume)
- [ ] Atmosphere
- [ ] Mass balance
- [x] Reactant concentration at the beginning of reaction
- [ ] Mass and/or heat transfer and mixing effects

Catalytic activity

- [ ] Reaction kinetics
- [x] Turnover frequency
- [x] Turnover number
- [x] Product selectivity
- [ ] Space-time yield
- [ ] Kinetics of deactivation

Catalyst stability assessment

- [ ] Long-term stability test, including test conditions
- [ ] Recyclability test
- [ ] Catalyst identity, loading, or purity were assessed post reaction (e.g. SEM, TEM, XRD, ICP, etc; details provided)

Control and benchmarking experiments

- [x] Reaction without catalyst
- [ ] Reaction without additives
- [x] Benchmarking table or figure (either other catalysts investigated in this study or previous literature reports with references)

Product or compound characterization

Identity

- [x] Integrated ^{1}H and ^{13}C NMR spectra provided
- [ ] Multiplicity and coupling constants provided in-text
- [ ] Other NMR experimentation provided
- [ ] High resolution mass spectral data
- [ ] Infrared (IR) absorption spectroscopy
- [ ] UV-vis spectroscopy
- [x] Chiral chromatography (GC and/or HPLC)
- [ ] X-ray diffraction (powder and/or single crystal)

Purity

- [x] Isolated yields
- [ ] High-field ^{1}H NMR spectra
- [ ] 1D proton-decoupled ^{13}C NMR spectra
- [ ] Combustion elemental analysis
- [ ] Quantitative GC or HPLC analytical data
- [ ] Electrophoretic analytical data
- [x] Sequence (biomacromolecules)
- [ ] Dispersity (polymers)

Quantification and statistical analysis

- [ ] The paper reports statistical analysis
- [x] There is a statement as to what (if any) methods were used to determine if the data met the assumptions of the statistical approach
- [ ] The statistical parameters (e.g., exact value of *n* samples, standard error of the mean, standard deviation) are reported in the paper

Computational analysis

- [ ] Calculations were conducted
- [ ] Software details, including version number
- [ ] Details of all basis sets and exchange-correlation functionals or wave function methods
- [ ] Force-field parameters
- [ ] Temperature and/or pressure (if non-standard conditions)
- [ ] Coordinates, calculated energies, and lowest frequency of all stationary points
- [ ] Intrinsic reaction coordinate to confirm transition states
- [ ] Data and code are available in a repository
- [ ] Convergence criteria of the force and energy
- [ ] Definitions of computed physical quantities and description of all corrections to electronic energies
- [ ] Ensemble
- [ ] k-point and supercell size
- [ ] Simulation cell details (if periodic calculations) or details if using molecular dynamics or Monte Carlo
- [ ] Pseudopotential

Other

- [x] The biocatalysis checklist is relevant for this work
- [ ] The electrochemistry checklist is relevant for this work
- [ ] The photocatalysis checklist is relevant for this work
- [ ] Other information is relevant for the general catalysis or general characterization reported in this manuscript (if so, please provide details below)

Biocatalysis checklist

CellPress

Revision 1.1, Last updated: November 08, 2021

General conditions

- [ ] The General Catalysis checklist has been completed

Catalyst identity

- [ ] The name from the IUBMB Enzyme List to identify the enzyme is provided
- [x] The NCBI Taxonomy ID is provided
- [x] A naturally occurring variant
- [ ] The localization within the cell
- [ ] Any post-translational modification are detailed
- [x] The full protein sequence and the appropriate NCBI GenBank or UniProt accession code is provided
- [x] Gene identifiers
- [x] Expression modules (i.e., regulatory sequences)
- [x] Plasmids used for expression
- [ ] Mutations within the gene or protein sequence (and an indication of whether the sequences are wild-type, synthetic and/or evolved)

Preparation

- [x] Novel methods are provided in full
- [ ] Metalloenzyme
- [x] Comprehensive literature references are included if the synthesis has been previously reported
- [ ] Artificial modification
- [x] Enzyme or protein purity

Storage and Propagation conditions

- [x] Storage solution
- [x] Storage temperature
- [ ] Atmosphere if not air
- [x] pH (if stored in solution)
- [x] Buffer and concentrations (including counter-ion)
- [ ] Metal salt(s) and concentrations
- [x] Enzyme or protein concentration
- [ ] Details regarding thawing procedure
- [x] Propogation medium
- [x] Propogation temperature
- [x] Antibiotic resistances
- [x] Statement about observed loss of activity under any of the preceeding conditions

Assay conditions

- [x] Substrate identity, purity, and concentrations
- [x] Buffer and concentrations
- [x] Metal salt(s) and concentrations
- [ ] Total ionic strength of assay mixture
- [x] Enzyme or protein concentration
- [x] Coupled assay components
- [x] Assay temperature, pressure, medium, and pH
- [ ] Atmosphere if not air
- [x] Culture vessel (e.g., flask, bioreactor, microtiter plate)
- [x] Measured reaction provided as stoichiometrically balanced equation

Activity/Performance

- [x] Measurements of initial rates of the reaction
- [ ] Specific substrate consumption rate q_S (in mol/g_{CDW}/h)
- [ ] Volumetric productivity Q_P (in kg/L/h or mol/L/hr)
- [ ] Proportionality between initial velocity and enzyme concentration
- [x] Turnover number
- [ ] Specific product formation rate q_P (in mol/g_{CDW}/h)
- [x] Enzyme activity expressed as k_{cat} (in s^{-1} or min^{-1}) or international unit (1 IU = 1 μmol min^{-1}); katal (mol/s) may alternatively be used as a unit of activity (conversion factor 1 unit = 16.67 nkat)

Methodology

- [x] Assay method
- [x] Type of assay
- [x] Reaction-stopping procedure
- [x] Direction of the assay
- [x] Reactant determined
- [x] Concentrations of free metal cations
- [ ] Reaction equilibrium constant
- [ ] Pathway intermediates
- [ ] By-products
- [x] Analytic methods for the detection of metabolites
- [x] *If applicable*: molecular cloning techniques
- [x] *If applicable*: recombinant DNA delivery techniques

Kinetic or physiological parameters

- [ ] k_{cat} (in s^{-1} or min^{-1})
- [x] V_{max}
- [ ] $S_{0.5}$ as concentration (e.g., mM)
- [ ] High-substrate inhibition, if observed, with K_i value
- [ ] Biomass yield on carbon substrate $Y_{X/S}$ (either g_{CDW}/g or g_{CDW}/mol)
- [ ] Substrate toxicity (minimum inhibitory concentration - MIC in g/L or mol/L)
- [ ] *If applicable*: tolerance to solvent concentrations (minimum inhibitory concentration - MIC in g/L or mol/L)
- [ ] K_m units or concentration necessary (e.g., mM)
- [ ] k_{cat}/K_m as concentration per time (e.g., mM^{-1} s^{-1})
- [x] Model used to determine the parameters
- [ ] Growth rate μ (in h^{-1}) or doubling time t_D in h)
- [ ] Hill coefficient, saturation ratio (RS), or other coefficients of cooperativity
- [ ] (By-)product toxicity (minimum inhibitory concentration - MIC in g/L or mol/L)

Inhibition or activation data

- [ ] Time dependence and reversibility
- [ ] Inhibition (K_i units necessary)

Other

- [ ] Other information is relevant for the biocatalysis reported in this manuscript (if so, please provide details below)

Electrochemistry checklist

Revision 1.1, Last updated: November 08, 2021

General conditions

- [ ] The General Catalysis checklist has been completed

Reaction conditions provided

- [ ] Cell type (H-cell, gas-diffusion type, etc.)
- [ ] Cell, electrode, and membrane material
- [ ] Electrode geometric area (cm^2)
- [ ] Scan rate for cyclic voltammograms
- [ ] Reactants
- [ ] Three-electrode or two-electrode configuration (half-cell or full cell, respectively)
- [ ] Bias potential and, for three-cell configuration, the reference electrode used
- [ ] Currents
- [ ] Dependence of current on scan or stir rate
- [ ] Treatment or polishing of the electrode
- [ ] pH for aqueous solutions (start, during reaction, end)
- [ ] Electrolyte
- [ ] Mass transfer conditions (rotation rate for rotating disc electrode; stir bar, flow rate in flow cells)

Data reported

- [ ] Vendor information, photographs, and/or schemes of any custom apparatus
- [ ] Normalized electrochemical surface area activity
- [ ] Electrochemical impedance spectroscopy (EIS)
- [ ] Mass activity
- [ ] Specific activity
- [ ] Mass balance
- [ ] Polarization plot (cell voltage versus current or current density)
- [ ] Electrochemically active surface area (ECSA, A/cm^2_{ECSA})
- [ ] Stability test conditions
- [ ] Current densities
- [ ] Faradaic efficiency
- [ ] Overpotential (including clear information about how the thermodynamic potential was determined, estimated, or calculated)

Other

- [ ] Other information is relevant for the electrochemistry reported in this manuscript (if so, please provide details below)

Photocatalysis checklist

CellPress

Revision 1.1, Last updated: November 08, 2021

General conditions

- [ ] The General Catalysis checklist has been completed

Reaction conditions provided

- [ ] Vendor information, photographs, and/or schemes of any custom apparatus and reaction setup
- [ ] Photocatalyst loading
- [ ] Substrate concentration
- [ ] Sacrificial donor
- [ ] Other additives
- [ ] Reaction vessel size, material, and thickness of glassware
- [ ] Total optical power impinging on the sample if liquid ($mW \cdot mL^{-1}$)
- [ ] Source and wavelength of light used for illumination
- [ ] Wavelength distribution of light
- [ ] Hole or electron scavengers
- [ ] Optical irradiance at the sample ($mW \cdot cm^{-2}$)

Data reported

- [ ] Quantum yields
- [ ] Photocatalytic efficiencies
- [ ] Apparent quantum yields or photonic efficiencies

Control experiments conducted

- [ ] Reaction without catalyst
- [ ] Reaction without light (on/off test and reaction conducted completely in the dark)
- [ ] Stern-Volmer or other quenching experiments

Other

- [ ] Other information is relevant for the photocatalysis reported in this manuscript (if so, please provide details below)
