# Supplementary material for: Expanding the repertoire of imine reductases by mining divergent biosynthetic pathways for promiscuous reactivity
Source: Chem Catal. 2024 Dec 19;4(12):101160. doi: 10.1016/j.checat.2024.101160 (PMC11876095; doi:10.1016/j.checat.2024.101160)
Supplement: Document S2. Article plus supplemental information [file mmc3.pdf]

Chem Catalysis

Article

Expanding the repertoire of imine reductases by mining divergent biosynthetic pathways for promiscuous reactivity

Graphical abstract

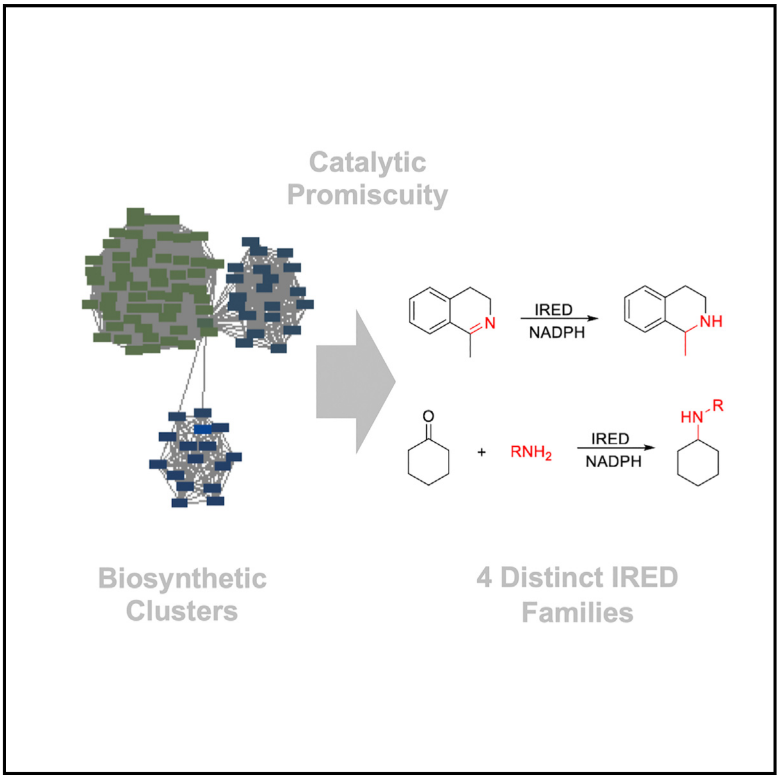

Highlights

- A functional genomics approach identifies new imine reductases (IREDs) for biocatalysis
- 44 enzymes from 6 non-homologous C=N bond reducing biosynthetic enzyme families screened
- 46% of these reduced synthetic imines, and some catalyze reductive carbonyl amination
- Prospecting unexplored sequence space is a route to imine reductases for synthesis

Authors

Godwin A. Aleku, Florian Hollfelder

Correspondence

godwin.aleku@kcl.ac.uk (G.A.A.),
fh111@cam.ac.uk (F.H.)

In brief

To identify useful biocatalytic enzymes in the vastness of sequence space, biosynthetic clusters with potential promiscuous activities for prospecting imine reductases (IREDs) like enzymes were defined. This approach guided the screening of 44 biosynthetic enzymes from six distinct non-homologous enzyme families, of which 46% showed activity and useful substrate scope for potential synthetic applications. These functional annotations based on experimental observations provide useful bridgeheads in previously unknown sequence space, provide practical guidelines for where to look for this type of activity, and increase confidence in further sequence-based annotations that are now more firmly anchored to actual observations.

Aleku & Hollfelder, 2024, Chem Catalysis *4*, 101160
December 19, 2024 © 2024 The Author(s).
Published by Elsevier Inc.
https://doi.org/10.1016/j.checat.2024.101160
CellPress

CellPress
OPEN ACCESS

Article

Expanding the repertoire of imine reductases by mining divergent biosynthetic pathways for promiscuous reactivity

Godwin A. Aleku[1,2,3,*] and Florian Hollfelder[1,*]
[1]Department of Biochemistry University of Cambridge, 80 Tennis Court Road, CB2 1GA Cambridge, UK
[2]Institute of Pharmaceutical Science, Franklin-Wilkins Building, King's College London, 150 Stamford Street, SE1 9NH London, UK
[3]Lead contact
*Correspondence: godwin.aleku@kcl.ac.uk (G.A.A.), fh111@cam.ac.uk (F.H.)
https://doi.org/10.1016/j.checat.2024.101160

THE BIGGER PICTURE Making enzymatic synthesis of active pharmaceutical ingredients (APIs) sustainable and attractive for industrial applications requires a repertoire of enzymes with activities and selectivities that surpass those of chemical synthesis methods. Reductive amination (RA) is one of the most frequently employed methods to synthesize amine-based APIs, so the use of imine reductases (IREDs) for this transformation is extremely attractive: catalytic reactions are performed with high efficiency and high stereoselectivity under mild conditions. Secondary and tertiary amines that are not accessible by other enzymatic methods can be synthesized in this way. However, the versatility of IRED-catalyzed reductive amination is limited by their substrate scope, so new IREDs to bridge gaps in specificity will increase the broad application of IREDs. Here, we explore a functional genomic approach to retrieve and characterize enzymes catalyzing unrelated physiological imine reduction reactions across several metabolic routes, revealing their promiscuity for the reductive amination of synthetic substrates. Our work has uncovered six useful enzyme families for imine reduction in previously unannotated sequence space, where their functional assignment creates "bridgeheads" for further searches for IREDs.

SUMMARY

Imine reductases (IREDs) are invaluable catalysts for enantioselective imine reduction and reductive amination of carbonyl compounds. Their synthetic versatility is, however, limited by their substrate scope, and new IREDs are needed. Current IREDs are closely related to the initially characterized enzymes, as their discovery has been driven by sequence homology searches. Here, we demonstrate a *functional* genomics approach based on biosynthetic promiscuity, guided by the identification of C=N reducing enzymes acting on large, complex substrates in biosynthetic pathways. These substrate-promiscuous biocatalysts share low homology to existing IREDs and fall into distinct functional enzyme families, yet they catalyze the hydrogenation of non-native imines as well as the reductive amination of simple ketones. Venturing further into sequence space without the constraints of close homology, but instead guided by functional promiscuity, has thus led us to distinct, previously unrecognized and unexplored areas of sequence space for mining IREDs for synthesis.

INTRODUCTION

Imine reductases (IREDs) have emerged as important members of the catalyst toolkit for the synthesis of chiral amine building blocks. IREDs (including the closely related subclass of reductive aminases [RedAms]) catalyze the NAD(P)H-dependent enantioselective reduction of imines and reductive amination of carbonyl compounds, allowing access to primary, secondary, and tertiary chiral amines.[1,2] Compared with other conventional methods (e.g., chemo-catalytic and transaminase-based routes), IRED-catalyzed reductive amination reactions often enable greener and shorter synthetic routes to 2° and 3° chiral amines by providing a direct and selective pathway to these amines without a further *N*-alkylation step.[3,4] A transaminase route, for instance, can only form 1° amines, and further *N*-alkylation step(s) and toxic reagents are required to access the corresponding 2° and 3° amine derivatives.[5] Abiotic reductive amination reactions, on the other hand, suffer from poor

 © 2024 The Author(s). Published by Elsevier Inc.
This is an open access article under the CC BY license (http://creativecommons.org/licenses/by/4.0/).

stereoselectivity and often yield the target products as racemic mixtures or in sub-optimal optical purity that require a further and tedious chiral resolution of the enantiomers. In addition, chemical reductive amination methods employing stoichiometric amounts of reducing reagents (such as $NaBH_4$, $NaBH(OAc)_3$, and $NaBH_3CN$) produce large quantities of undesired boronic or cyanide by-products.[6] Specialized and potentially hazardous equipment setups and high catalyst loadings are usually necessary in transition-metal-catalyzed reductive amination reactions where hydrogenation is performed with dihydrogen gas (using H_2/transition metal systems).

Since chiral amines are among the most important building blocks in the synthesis of active pharmaceutical ingredients (APIs), asymmetric amine-forming catalytic systems such as those afforded by IREDs are highly sought after. Recent examples of successful industrial exploitation of IRED/RedAm technology[7–10] demonstrate considerable interest in this enzyme family. This translational success fuels the motivation to establish the biocatalytic IRED route as a mainstream method of choice for the asymmetric reductive amination of carbonyl compounds. To realize this ambition, the narrow substrate scope of IREDs must be significantly expanded, as several frequently used reductive amination substrates, such as aromatic/bulky ketones and amines, are only poorly tolerated by existing IRED toolkits.[1,11,12]

This problem notwithstanding, the effort devoted to the characterization of synthetically useful IREDs has been remarkable, with several academic and industry groups contributing to the field. These efforts have, over the past decade, yielded hundreds of experimentally characterized IREDs (Figure 1A). The availability of *Streptomyces*' IREDs as query sequences, identified through the screening of microbial cultures,[13–15] paved the way for several genome mining IRED discovery projects in which putative bacterial homologs were identified by sequence homology and experimentally validated.[16–22] Through sequence homology search, several homologs of the characterized enzymes were assembled to construct the IRED Engineering Database.[23] Others have assessed the distribution of IREDs across different taxa, opening up fungal, plant, and metazoan IRED sequence space.[1,11,22] Lastly, a sequence-based metagenomic approach has recently been explored to further expand the IRED toolbox[24,25] (Figure 1A). The aforementioned IRED discovery strategies have relied on sequence homology to the extent that the vast majority of known IREDs are closely related, homologous proteins. The narrowly defined sequence window implies that they share similar functional, catalytic, or synthetic properties and limitations or potential for directed evolution. It is, therefore, not surprising that the hugely expanded IRED toolbox has not yet translated to a significant extension of the substrate scope of this enzyme class, highlighting the need for a discovery effort that is not driven by sequence homology alone but instead primarily by function. Exploration of alternative enzyme families that catalyze imine reduction has been considered with some promising results,[22,26,27] but an extensive functional investigation of multiple distinct enzyme families for biocatalytic application as IREDs is not on record.

This work addresses whether IREDs can be found using a sequence-independent strategy to reveal functional proteins in phylogenetically distinct sequence space that transcends the currently narrow sequence definition of contemporary IREDs. Specifically, our strategy explores enzymes that have been shown to reduce theC=N bond of biosynthetic intermediates in different biosynthetic/metabolic pathways across almost all clades of life. Even though biosynthetic enzymes are often perceived to display strict substrate specificity toward their native substrates,[28–31] catalytic and substrate promiscuity is a recognized, intrinsic property of many enzymes that allow them to act on alternative substrates, as an evolutionary "head start" en route to the acquisition of a new function after gene duplication.[32–34] Here, we systematically explore whether such C=N bond-reducing enzymes are promiscuous toward non-native imine substrates relevant for IRED applications and whether this search, only driven by a functional but not sequence-defined search profile, would uncover novel IREDs in areas of sequence space that is remote from the current cluster of known IREDs. Our approach involves (1) the selection of functionally and phylogenetically divergent enzyme families based on their recorded physiological role as NAD(P)H-dependent C=N reductases in the literature, regardless of sequence homology, (2) a preference for reductases that work on relatively large substrate molecules in the center of which the C=N reduction occurs (to bias for a large binding site able to accept bulky IRED substrates, for which few catalysts exist), and (3) experimental testing of recruited sequences per each selected functional family to determine the number of hits for each selected functional family, their activity, and their stereoselectivity. For each selected family, an average of seven sequences with varying degrees of sequence identity (35%–80%) were screened.

RESULTS AND DISCUSSION

Construction of a diverse enzyme collection

We identified a panel of 44 phylogenetically diverse C=N reducing enzymes covering a wide range of functional families, each with distinct native imine substrate specificity (Figures 1B and S1; Table S1; supplemental information). Specifically the panel comprises enzymes acting on pterin **1** and pteridine **2** such as (1) dihydrofolate reductases (DHFRs) and (2) pteridine reductases (PTR1s; PruAs), respectively; (3) short-chain dehydrogenases (SDRs) catalyzing C=N bond (instead of C=O) reduction in the biosynthesis of plant alkaloids, e.g., norcraugsodine **3**[35–39]; and (4) bacterial enzymes including naphthyridinomycin biosynthetic enzyme (NAPW) and homologs catalyzing C=N reduction of the iminium precursors of tetrahydroisoquinoline (THIQ) antibiotics, e.g., naphthyridinomycin iminium **4**.[40] Others include (5) imino acid reductases such as those catalyzing C=N reduction in the metabolism of acyclic imino acids, e.g., iminosuccinate **5**,[28,41] as well as those catalyzing the reduction of cyclic imino acids, e.g., 1-peperideine/pyrroline-2-carboxylate **6**.[42–44] (6) Lastly, representatives of the "classical" IRED family catalyzing imine reduction in biosynthetic pathways. For example, RedE, an uncharacterized metagenomic IRED-like biosynthetic enzyme acting on pyrrole/pyrrolinium indolocarbazole core **7**, a precursor of tryptophan dimer natural product,[45] as well as related homologs from Antarctica (from *Mortierella antarctica* [*Ma*RedAm]) and a tropical habitat (from a metagenomic bacterium [*Bac*RedAm]).

CellPress
OPEN ACCESS

A

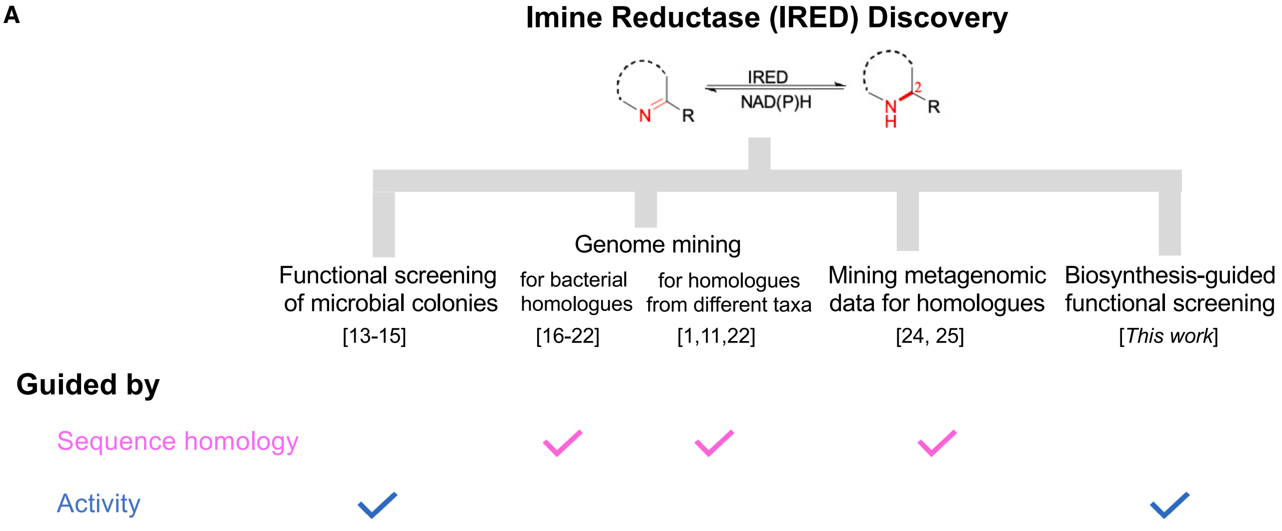

B

Enzyme family	No. of candidates screened	Native substrate
(i) Pteridine reductases (PTRs)	7	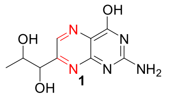
(ii) Dihydrofolate reductases (DHFRs)	7	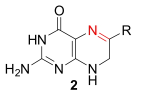
(iii) SDRs involved in plant alkaloid biosynthesis	9	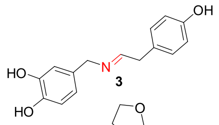
(iv) SDRs involved in bacterial alkaloid biosynthesis	3	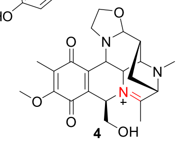
(v) (A)cyclic iminoacid reductases	8	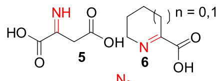
(vi) Classical IREDs	8	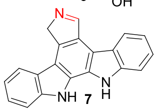

Figure 1. Past and present enzyme discovery approaches to identify IREDs

(A) Approaches are classified by exploratory principle: either by searching for sequences that are homologous to the few known IREDs or by testing candidates experimentally (regardless of homology to known IREDs), with references to exemplify these.

(B) In this work, a systematic functional exploration of potentially promiscuous biosynthetic C=N bond-reducing enzymes toward the reduction of non-native imine substrates was performed. The summary shows enzyme classes as named by their diverse known reactions with their native substrates. Compounds: pteridine **1**, dihydropterin **2,** norcraugsodine **3**, naphthyridinomycin iminium **4**, imminosuccinate **5**, (1)-piperideine/pyrroline-2-carboxylate **6,** and pyrrole indolocarbazole core **7**.

See also the supplemental information, Table S1, and Figure S1.

A cladogram of these divergent sequences reveals distinct clusters (Figure 2A), namely clades 1–6, with each clade containing further monophyletic group(s) (e.g., clades 3a, 3b, and 3c and clades 6a, 6b, and 6c). A sequence similarity network analysis[46,47] showed a similar pattern of clustering (Figure S2; supplemental information). Using clade 1 (containing classical

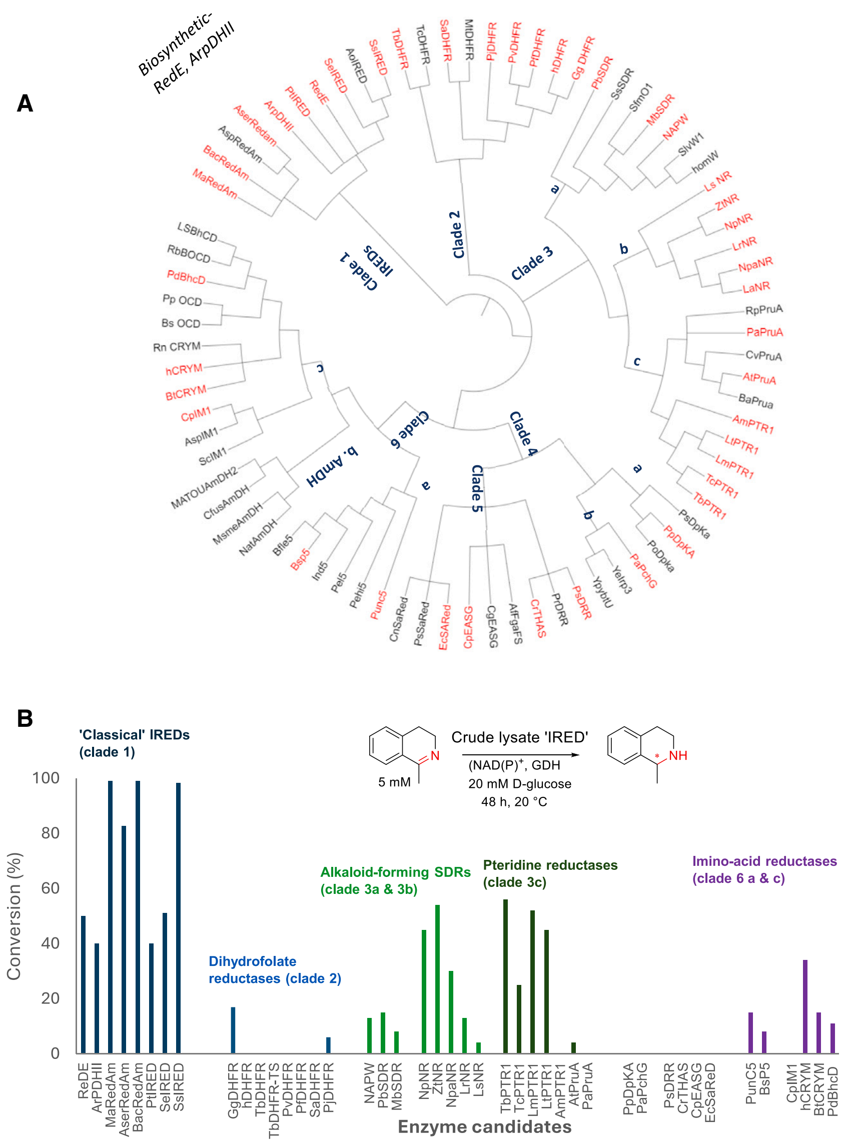

Figure 2. Systematic evaluation of NAD(P)H-dependent C=N bond-reducing (biosynthetic) enzymes for promiscuous IRED activity

(A) A cladogram showing highly divergent (biosynthetic) enzymes recruited for this study (in red) from distant or unrelated clades.

(B) HPLC conversion obtained from analysis of biotransformation reactions using recombinantly expressed (in *E. coli*) (biosynthetic) C=N bond-reducing enzyme/homolog against a model non-native isoquinoline imine substrate. For NAPW, *Pb*SDR, and *Mb*SDR, conversion values for the reduction of the imine myosmine are presented. Hit rate (%) = (number of enzyme candidates affording detectable product formation as monitored by reverse phase-HPLC/total number enzymes screened in that family) × 100. Hit rate (%) for clade 1 = 100%; clade 2 = 25%; clade 3 = 87%; clades 4 and 5 = 0%; and clades 6a and 6c = 66%.

IREDs) as a reference clade, generated percent sequence identity (PID) matrices clearly show each clade as, indeed, a unique sequence space with significantly low PIDs of 10%–17% to known IREDs (Figures S3–S5; supplemental information).

Screening for NADPH-dependent imine-reducing activity on non-native substrates

Representative sequences for each clade were chosen and cloned into pET-based vectors (pET28a, pET15b, and pET22b) (see supplemental information for gene sequences). These plasmids were each expressed in *E. coli* BL21(DE3) to produce recombinant whole-cell biocatalysts (supplemental information; Figures S20 and S21). A preliminary screening for NAD(P)H-dependent imine-reducing activity was performed by monitoring the high-performance liquid chromatography (HPLC) conversion of the imine 1-methyl-3,4-dihydroisoquinoline **8** to the corresponding amine **9** and/or bioreduction of myosmine **10** to nornicotine **11** following 48 h incubation with lysates of recombinant IRED-expressing *E. coli* cells in lysozyme-based lysis buffer (pH 7.0). These substrates have been chosen for an initial affirmation of IRED function because, empirically, the majority of synthetically useful IREDs have been found to reduce isoquinoline and pyrroline imines. In addition, isoquinolines and pyrrolidines are prevalent motifs in many pharmaceuticals.

Analysis of biotransformation reactions revealed that ~60% of the biosynthetic enzymes from functionally diverse classes displayed imine-reducing activity on one or both non-native imine substrates **8** and **10** (Figure 2B), although group-specific hit rates ranged from 0% to 100%. Of the eight members investigated from clade 1 (REDE homologs, the classical IREDs), all displayed imine-reducing activity toward **8** or **10**, representing a 100% hit rate. In contrast, only two members, *Pj*DHFR from *Pneumocystis jirovecii* (fungal) and *Gg*DHFR from *Gallus gallus* (avian), of the eight screened from clade 2 (DHFRs) displayed weak imine-reducing activity toward **8**, equivalent to a 25% hit rate. 87% (13) of the 15 members of clade 3 (PTR1s, norcraugsodine reductases [NRs], and NAPW-like enzymes) converted either **8** or **10** to the corresponding amine. The protozoan PTR1s and NRs from plants afforded moderate conversions, with the other hits in this clade only displaying weak activity (Figure 2B).

Six members of clades 4 and 5 were screened, but no detectable product was observed under the biotransformation conditions based on HPLC analysis. For clades 6a and 6c (imino acid reductases), six members were also screened, of which 66% displayed weak imine-reducing activity toward **8**. These include the acyclic imino acid reductase PunC5/BsP5 from *Paenibacillus* sp./*Bacillus* sp., iminosuccinate reductase *Pb*BcHD from *Paracoccus denitrificans*, and the mammalian ketimine reductase mu-crystallin (CRYM). It is worth mentioning that

some of these enzymes (e.g., DHFRs, NAPW, PunC5, BsP5) were unstable and enzymatic imine-reducing activity appears to vary with each enzyme batch. Native amine dehydrogenases (NatAmDHs) that form a distinct branch (6b) within clade 6 were excluded from this study, as significant effort has recently been devoted to their biocatalytic exploitation.[24,48]

Using semi-purified enzyme preparations, we examined the stereoselectivity of selected representatives of clades 1, 2, 3, and 6 toward the reduction of imines **8** and **10** (Table 1). RedE displayed weak activity toward **8** but efficiently reduced myosmine **10** to the corresponding *S*-nornicotine **11** (90% conv., >99% enantiomeric excess [*e.e.*]), while related homologs *Ma*RedAm, *Bac*RedAm, and and the reductive aminase from *Aspergillus sergii (Aser*RedAm) efficiently reduced **8** to the (*R*)-amine **9** (>99% conv., 48%–92% *e.e.*). *Bac*RedAm and *Ma*RedAm also efficiently converted **10** to the (*S*)-amine (>99% conv.), whereas *Aser*RedAm displayed weaker activity toward this substrate.

Protozoan PTR1s from kinetoplastid parasites, including from *Trypanosoma brucei* (*Tb*PTR1), *T. cruzi* (*Tc*PTR1), *Leishmania major* (*Lm*PTR1), and *L. tarentolae* (*Lt*PTR1), reduced both **8** and **10.** While *Lm*PTR1 and *Tb*PTR1 reduced **8** to the corresponding (*S*)-**9**, albeit in moderate to low *e.e.* (13%–54% *e.e.*), *Tc*PTR1 and *Lt*PTR1 produced the corresponding (*R*)-amine in excellent enantioselectivity (up 96% *e.e.*). The PTR1s all yielded the (*R*)-**11** from the reduction of **10,** irrespective of their selectivity toward amine **9**. The avian DHFR (*Gg*DHFR) reduced **8** to (*R*)**-9** (97% conv., >98% *e.e.*), but no conversion of **9** to **10** could be detected.

The plant alkaloid-forming biosynthetic enzyme NR (*Np*NR from *Narcissus pseudonarcissus*), which has been previously investigated for imine reduction of other cyclic imine substrates,[19] and homologs from *Zephyranthes treatiae* (*Zt*NR) and *Lycoris radiata* (*Lr*NR) afforded high conversion of **8** to (*R*)-**9,** albeit with poor *e.e.* values. However, *Zt*NR and *Np*NR displayed stereo-complementary selectivity for the reduction of imine **10**, yielding (*S*)-**11** (30% *e.e.*) and (*R*)-**11** (28% *e.e.*), respectively. An alkaloid-forming bacterial dehydrogenase, NAPW-like protein from *Paenibacillus* sp. (*Pb*SDR), formed (*S*)-**11** from **10** (68% conv., 91% *e.e.*). Representatives of (a)cyclic imino acid reductase, including *Homo sapiens ketimine reductase mu-crystallin* (*hs*CRYM), Punc5, and imminosuccinate reductase (β- hydroxyaspartate cyclodeaminase from *Paracoccus denitrificans, Pd*BhCD), yielded (*R*)-**9** with near-perfect selectivity (>98% *e.e.*); however, substrate **10** was barely converted by *hs*CRYM and PunC5 (<3% conv.). Control reactions with stoichiometric amounts of NAD(P)H confirmed the IRED activity and showed a similar trend to the observed activity when using a glucose dehydrogenase (GDH)-recycling system (see supplemental information and Table S2).

All the enzymes investigated in this work displayed a preference for NADPH, although NADH was also accepted, and in some cases, e.g., *Ma*RedAm, *Lt*PTR1, *Zt*NR, and *Am*IRED, showed comparable conversion with both cofactors. The pH profile for the imine-reducing activity of selected enzymes showed optimal activity at pH between 6 and 7, with most of the enzymes also maintaining high activity at pH 5 (see supplemental information and Figure S6). Activity at weakly acidic pH can be useful when handling substrates that are labile at basic pH[7] and in cascade reactions involving (de)carboxylases.[50,51]

To further examine the synthetic potential of members of PTR1 and NR families and benchmarking against members of the classical IRED family, we performed biotransformations at higher substrate loading for the reduction of cyclic imines **8** (100 mg preparative scale, 25 mM, 28 mL reaction) and **10** (25 mM, 1 mL reaction), as well as for bulkier imines, salsolidine imine **12** (10 mM, 1 mL reaction) and harmaline **14** (10 mM, 1 mL reaction) (Table 1B). At this elevated substrate loading, the PTR1s, NRs, and IREDs retained moderate to high conversion values for imines **8** and **10** (32% to >99% conv.) to the corresponding enantioenriched products **9** and **11** (Table 1B). Isolated yields and *e.e.* values for the preparative-scale reactions for the reduction **8** to (*R*)-**9** were 92% yield, 95% *e.e.*; 25% yield, 98% *e.e.*; and 30% yield, 24% *e.e.* for *Ma*RedAm-catalyzed, *Lt*PTR1-catalyzed, and *Zt*NR-catalyzed reactions, respectively. For the reduction of imines **12** and **14**, a difference in substrate tolerance could be observed within members of the same family. For example, *Lt*PTR1 showed moderate conversion (41%) toward **12**, yielding (*R*)-**13** (>98% *e.e.*), but only weak or trace activity could be detected with *Tb*PTR1 and *Lm*PTR1 with the same substrate. Harmaline **14** was reduced to the corresponding amine (*S*)-**15,** albeit in low conversion values (6%–18%).

The plant enzyme *Zt*PTR1 was efficient toward the reduction of salsolidine imine **12** (>99% conv., 38% e.e. [*S*]) and harmaline **14** (97% conv., 89% *e.e.* [*R*]), while *Np*NR displayed a slower conversion rate with these substrates. Similar trends were observed with the classical IREDs. *Ma*RedAm efficiently reduced imine **12,** affording the corresponding amine (*R*)-**13** (>99% conv., >98% *e.e.*), but displayed poor activity toward imine **25** (6% conv.). In contrast, *Bac*RedAm reduced **14** to form (*S*)-**15** in excellent conversion and *e.e.* (93% conv., 98% *e.e.*) but only showed weak activity toward the reduction of imine **12.**

The protein sequence space shown to harbor enzymes with imine-reducing activity from this work extends far beyond the classical definition of the IRED sequence space. For example, sequences included in the IRED Engineering Database (www.ired.biocatnet.de), which provides the most extensive coverage of currently known IREDs (>1,400 sequences), fall in their entirety under clade 1 (Figure 2A).[23] Indeed, a BLAST search against this database using any member of clade 1 as a query sequence returns hits with significant homology scores. However, a similar homology search using representative sequences of all other clades 2, 3, 4, 5, and 6 scanned against the IRED Engineering Database did not return any hits, indicating that the five other distinct enzyme families (clades) described here cover new ground: i.e., are sequence diverse and phylogenetically distinct from known IREDs. This test suggests that the new clades will provide the basis for a significant extension of IRED diversity by functional annotation of proteins in sequence space that act on synthetic imine substrates as IREDs or provide a starting point for their directed evolution.

Application of representative members in reductive amination

Encouraged by the activity of members of clade 3 on isoquinoline and pyrroline imines and the high imine-reducing hit rate of this

Table 1. Biotransformation for the enantioselective reduction of non-native imines catalyzed by a panel of highly divergent (biosynthetic) enzymes

(a)

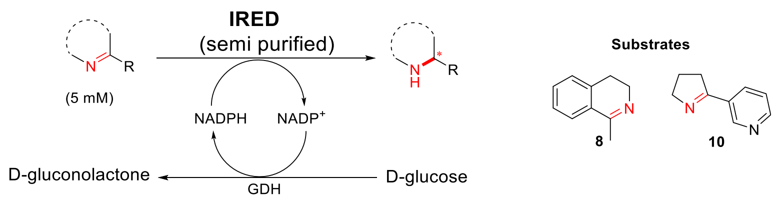

Entry	Enzymes	8		10	
Enzymes	Functional family	Conv. (%)	*e.e.*% (Abs. conf.)	Conv. (%)	*e.e.*% (Abs. conf.)
ReDE	classical IRED (clade 1)	<5	N/D	79	70 (*S*)
*Ma*RedAm	classical IRED (clade 1)	>99	48 (*R*)	>99	31 (*S*)
*Bac*RedAm	classical IRED (clade 1)	>99	92 (*R*)	>99	98 (*S*)
*Aser*RedAm	classical IRED (clade 1)	>99	89 (*R*)	15	95 (S)
*Gg*DHFR	dihydrofolate reductase (clade 2)	48	>98 (*R*)	N/D	N/D
*Lt*PTR1	pteridine reductases (clade 3a)	>99	96 (*R*)	79	90 (*R*)
*Tb*PTR1	pteridine reductases (clade 3a)	56	13 (*S*)	58	84 (*R*)
*Lm*PTR1	pteridine reductases (clade 3a)	78	54 (*S*)	>99	98 (*R*)
*Tc*PTR1	pteridine reductases (clade 3a)	67	94 (*R*)	n.t.	n.t.
*Zt*NR	alkaloid-forming SDR (clade 3b)	>99	25 (*R*)	75	30 (*R*)
*Np*NR	alkaloid-forming SDR (clade 3b)	89	20 (*R*)	60	29 (*S*)
*Lr*NR	alkaloid-forming SDR (clade 3b)	65	93 (*R*)	45	96 (*R*)
*Pb*SDR	alkaloid-forming SDR (clade 3b)	<3	n.d.	68	91 (*S*)
*Hs*Crym	imino acid reductase (clades 6a and 6c)	68	>98 (*R*)	<3	n.d.
PunC5	imino acid reductase (clades 6a and 6c)	76	>98 (*R*)	<3	n.d.
*Pd*BhcD	imino acid reductase (clades 6a and 6c)	85	>98 (*R*)	8	n.d.

(b) **Substrates**

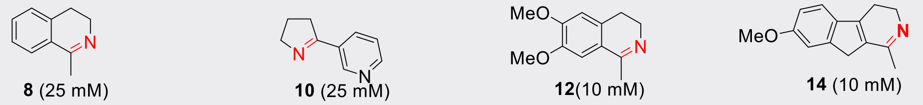

Products

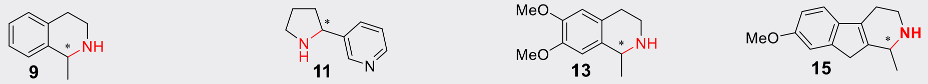

	9	11	13	15
**BacRedAm**	**>99% conv., 98% *e.e.* (*R*)**	**57% conv., >98% *e.e.* (*S*)**	**<3% conv., n.d.**	**93% conv., >98% *e.e.* (*R*)**
**MaRedAm**	**>99% conv., 95% *e.e.* (*R*)**	**99% conv., >98% *e.e.* (*S*)**	**>99% conv., >98% *e.e.* (*R*)**	**6% conv., >97% e.e. (*R*)**
***Lt*PRT1**	**>99% conv., 98% *e.e.* (*R*)**	**79% conv., 90% *e.e.* (*R*)**	**41% conv., >98% *e.e.* (*R*)**	**18% conv., >98% *e.e.* (*R*)**
***Lm*PTR1**	**32% conv., 54% *e.e.* (S)**	**>99% conv., >98% *e.e.* (*R*)**	**<1% conv., n.d.**	**6% conv., 11% e.e. (*S*)**
***Tb*PTR1**	**35% conv., 13% e.e. (*S*)**	**58% conv., 84% e.e. (*R*)**	**6% conv., >97% e.e. (*S*)**	**18% conv., 67% e.e. (*R*)**
***Zt*NR**	**>99% conv., 24% e.e. (*R*)**	**75% conv., 30% e.e. (*R*)**	**>99% conv., 38% e.e. (*S*)**	**97% conv., 89% e.e. (*S*)**
***Np*NR**	**89% conv., 20% e.e. (*R*)**	**55% conv., 29% e.e. (*S*)**	**25% conv., 2% e.e. (*S*)**	**34% conv., 8% e.e. (*R*)**

N.B. Reaction conditions for scheme **a**: 5 mM imine, 20 mM D-glucose, 0.5 mM $NADP^+$, 0.25–1 mg mL^{-1} IRED (reactions were performed with semi-purified IRED preparation), 0.25 mg mL^{-1} glucose dehydrogenase (GDH; cell-free extract), in 0.5 mL phosphate buffer (100 mM with 100 mM NaCl, pH 7.0). The reaction was incubated at 20°C for 12–48 h. Reaction conditions for scheme **b**: 10–25 mM imine, 30–50 mM glucose, 1 mM $NADP^+$, 1–2 mg mL^{-1} IRED, 0.5 mg mL^{-1} GDH (lyophilized cell-free extract), in phosphate buffer (100 mM with 100 mM NaCl, pH 7.0). The reaction was incubated at 25°C for 48 h. The absolute configurations of biotransformation products were determined by comparing the selectivity observed here to previously characterized *Ao*IRED[49] and *Asp*RedAm[1] under the same reaction conditions and using the same HPLC chiral columns and screening methods. n.t., not tested; N/D, product not detected; n.d., not determined; Abs. conf., absolute configuration.

CellPress
OPEN ACCESS

Table 2. Reductive amination of carbonyl compounds catalyzed by (biosynthetic) IREDs

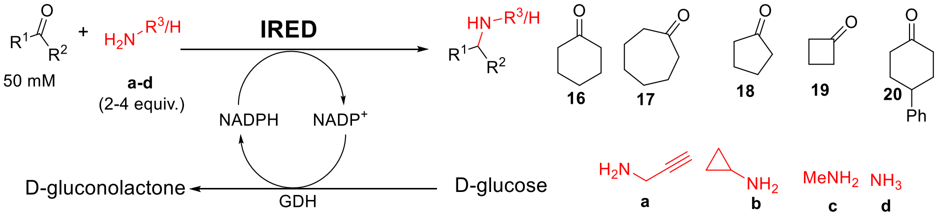

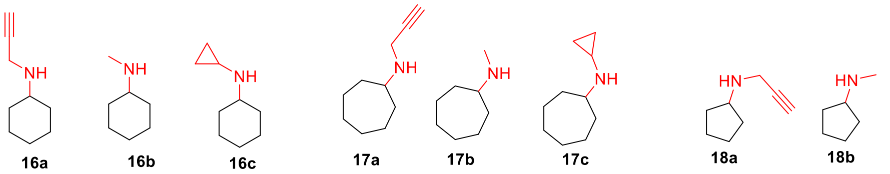

Enzyme	Conversion (%) 16a	16b	16c	17a	17b	17c	18a	18b
*Ma*RedAm	>99	95	>99	98	61	88	94	53
*Bac*RedAm	>99	>99	>99	86	50	66	80	66
*Lt*PTR1	78	62	55	87	29	81	82	15
*Tb*PTR1	28	5	23	20	n.t.	16	30	N/D
*Lm*PTR1	41	24	24	22	n.t.	15	25	8
*Zt*NR	22	N/D	6	21	n.t.	n.t.	18	n.t.
*Np*NR	16	N/D	N/D	20	n.t.	n.t.	15	n.t.

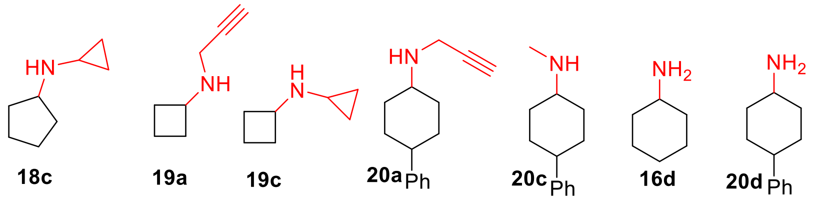

Enzyme	Conversion (%) 18c	19a	19c	20a	20c	16d	20d
*Ma*RedAm	80	94	90	93	71	90	30
*Bac*RedAm	62	83	74	89	80	15	32
*Lt*PTR1	46	45	23	53	56	8	14
*Tb*PTR1	11	15	n.t.	n.t.	n.t.	N/D	n.t.
*Lm*PTR1	5	21	n.t.	n.t.	n.t.	N/D	n.t.
*Zt*NR	n.t.	N/D	N/D	n.t.	8%	N/D	N/D
*Np*NR	n.t.	N/D	N/D	n.t.	N/D	N/D	N/D

N.B. Reaction conditions: [carbonyls]: **16**, **17**, **18**, and **19** (50 mM) and **20** (10 mM); [amine nucleophile]: **a** and **b** (2 equiv) and **c** and **d** (4 equiv). 100 mM glucose, 0.5 mM $NADP^+$, 0.5–1 mg mL^{-1} (semi-)pure IRED, 0.5 mg mL^{-1} glucose dehydrogenase (GDH; cell-free extract), 7.5 mM $MgCl_2$ in 0.5 mL 100 mM Tris-HCl (100 mM NaCl, pH 9.0). The reaction was incubated at 20$^{\circ C}$ for 12–48 h. n.t., not tested; N/D, product not detected.

group, we became interested in the prospect of these biosynthetic enzymes for the reductive amination of ketones with alkylamines, given the synthetic usefulness of this transformation. Hence, alongside members of clade 1, we examined the performance of representative members of clade 3 toward the reductive amination of simple cyclic ketones (cyclohexanone **16**, cycloheptanone **17**, cyclopentanone **18**, cyclobutanone **19**, 4-phenylcyclohexanone **20**) with alkylamines (propargylamine **a**, cyclopropylamine **b**, methylamine **c)** and ammonia, **d** (Table 2).

*Ma*RedAm-catalyzed reductive amination of carbonyls **16–19** (50 mM) with alkylamines **a**, **b**, and **c** (2 equiv.) afforded high conversion (**16a–16c,** 95% to >99% conv.; **17a–17c,** 50%–98%

conv., **18a–18c,** 53%–94% conv., **19a** and **19c**, 74%–89% conv.), and comparable conversion values were obtained with *Bac*RedAm (Table 2). Similar conversion values were also observed with 4-phenylcyclohexanone **20** (10 mM), with *Ma*RedAm and *Bac*RedAm efficiently coupling amines **a** and **c** to form the corresponding amine products **20a** (89%–93% conv.) and **20c** (71%–80% conv.), respectively.

Members of the PTR1s (clade 3c) displayed similar substrate tolerance to *Ma*RedAm and *Bac*RedAm. *Lt*PTR1, in many cases, afforded comparable conversion values with the prototype RedAms (e.g., **16a**, 78% conv.; **17a**, 87% conv.; **18a**, 82% conv.; **19a**, 45% conv.; **20a**, 53% conv.), whereas *Tb*PTR1 and *Lm*PTR1 yielded these products but in lower conversion values. Other alkylamines, such as cyclopropylamine **b** and methylamine **c**, were also accepted by *Lt*PTR1 as amine nucleophiles, furnishing the corresponding amine products in modest conversion values. *Ma*RedAm-catalyzed amination of **16** (50 mM) with NH_3 **d** (supplied as 200 mM NH_4Cl) afforded 90% conv. to the primary amine **16d**, while conversion values were significantly lower with *Bac*RedAm (**16d**, 15% conv.) and *Lt*PTR1 (**16d**, 8% conv.). These enzymes also formed 4-phenylcyclohexylamine **20d** from 4-phenylcyclohexanone **20** and NH_3, **d** (**20d**, *Ma*RedAm, 32% conv.; *Bac*RedAm, 30% conv.; and *Lt*PR1, 14% conv.).

The plant enzymes (clade 3b) *Zt*NR and *Np*NR displayed activity toward the amination of **16**–**18** with propargylamine **a**, affording the corresponding *N*-alkylated propargylamine products **16a**–**18a**, albeit with significantly lower conversion values. However, both enzymes could not use ammonia or the small alkylamine $MeNH_2$ and showed weak activity toward amination of **16** with cyclopropylamine **b** (*Zt*NR, **16b**, 5% conv.) (Table 2).

Preparative-scale biotransformation reactions for the amination of cyclohexanone **16** (100 mg, 1.02 mmol, total reaction volume, 41 mL) with 2 equiv of propargylamine **a** were performed, yielding the corresponding secondary amine **16a** (84% yield for *Ma*RedAm-catalyzed reaction and 63% yield for *Lt*PTR1-catalyzed reaction).

To examine the pattern of substrate specificity for both carbonyl and amine coupling partners, we carefully constructed a small substrate panel containing ketones **16** and **22**, aldehyde **21**, α-keto ester **23**, and α-keto acids **24** and **25** as carbonyl acceptors, and propargylamine **a**, cyclopropylamine **b**, methylamine **c,** and ammonia **d** were included as the amine coupling partners (Table 3). Using purified enzyme preparation, representative members of clades 1, 2, 3, 5, and 6 were each screened against the various substrate combinations of this substrate panel, monitoring the NADPH-dependent reductive amination initial rate at 340 nm using a microtiter plate reader (Table 3).

*Ma*RedAm (clade 1) was the most versatile of these catalysts, displaying high to moderate activity for the ketone/aldehyde and alkylamine combinations, with specific activity of up to 11.6 U mg^{-1}. *Ma*RedAm-catalyzed reductive amination activity was one order of magnitude lower when ammonia was used instead of alkylamines (Table 3). *Ma*RedAm also exhibited reductive amination activity toward the α-keto ester **23** and α-keto acids, **24** and **25**, albeit two orders of magnitude slower relative to the rates observed with cyclohexanone/hydrocinnamaldehyde and alkylamines. *Lt*PR1, a representative from clade 3c, showed similar amine specificity to *Ma*RedAm. However, reaction velocities were 10- to 100-fold slower when compared with *Ma*RedAm for the same substrate combinations. Activity toward α-keto acids was not detected under the screening conditions. Pseudomonas putida ketimine reductase (*Pp*Dpka) showed similar amine substrate specificity to *Ma*RedAm but distinct specificity for the carbonyl acceptor. *Pp*Dpka exhibited high specific activity toward the amination of α-keto ester **23** and α-keto acids **24** and **25** with alkylamines (up to 5 U mg^{-1}); activity toward ketones **16** and **22** and aldehyde **21** was not detected under the conditions of this assay. *Hs*CRYM (clade 6c), PunC5 (clade 6a), and the avian DHFR (*Gg*DHFR, clade 6a) displayed activity for α-keto acids/esters with alkylamines and, in some cases, ammonia (e.g., PunC5); however, methylamine **c** was the preferred alkylamine nucleophile for these substrates.

The screening against this carefully designed, albeit small, substrate panel has revealed that several non-homologous enzyme families investigated can catalyze reductive amination with primary amines to form *N*-alkylamines, *N*-alkylamino esters, and *N*-alkylamino acids (Table 3). Hence constructing IRED panels to contain representative members from these distinct and non-homologous enzyme families should significantly extend the amine product scope that can be accessed compared to conventional IRED kits.

To demonstrate the utility of observed activities for biotransformation reactions, we focused on PTR1s and NRs (again benchmarking against the classical IREDs in clade 1) for the reductive amination of hydrocinnamaldehyde **21** (30 mM), benzaldehyde **26** (10 mM), and a prochiral ketone, 4-phenyl-2-butanone **22** (10 mM). PTR1s efficiently catalyzed the amination of hydrocinnamaldehyde with propargylamine **a** to form the corresponding secondary amine coupling product **21a** (54%–95% conv.) as well as the coupling of benzaldehyde **17** with **a**, yielding norpargyline **26a** (up to >99% conv.). However, PTR1s only showed low conversion (up to 6%) when tasked with the amination 4-phenyl-2-butanone **22** with propargylamine **a** or allylamine **e**. Similarly, NRs catalyzed the amination benzaldehyde **26** with **a** to afford the corresponding amine **26a** in high conversion (up >99%) but displayed weak amination for hydrocinnamaldehyde **21** and only trace activity for the amination of **22**. In contrast, the classical IREDs/RedAms performed well across these substrates, affording high conversion of up to >99%. For the reductive amination of ketone **22** with amines **a** or **e**, the (*R*)-configured amine products ((*R*)**-22a**, and (*R*)-**22e**) were generated with moderate to high enantioselectivity of up to 95% *e.e.* Although this preliminary screen shows that PTR1s and NRs are suitable for the amination of aromatic aldehydes and simple cyclic ketones and less efficient for the amination of (aromatic) ketones, an extensive substrate profiling study is needed to map out the distinctive substrate specificities of PTR1s and NRs, as well as the other enzyme families described in this work.

The classical IREDs emerged as the most versatile of these enzyme families for synthetic application in the reductive amination of ketones. Hence, we further investigated the efficiency of the novel IREDs/RedAms identified in this work (Table S1) toward the amination of difficult-to-aminate bicyclic aromatic ketones such as 1-indanone **27** and 1-tetralone **28**. The synthesis of α-secondary amines from bicyclic aromatic ketones represents one of the most challenging reactions for existing

CellPress
OPEN ACCESS

Table 3. Comparison of substrate specificity of representative members from distinct enzyme families using a small substrate panel

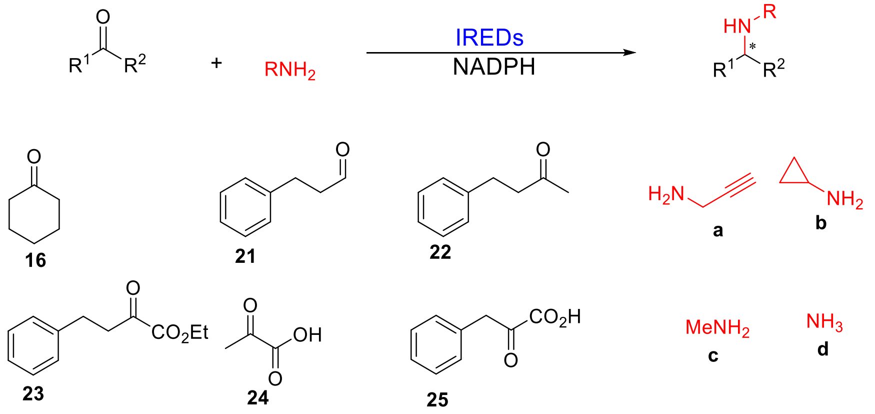

Specific activity (U mg^{-1})

Amine nucleophile	Carbonyl acceptor	*Ma*RedAm (clade 1)	*Lt*PTR1 (clade 3c)	*Pp*Dpka (clade 4)	*h*CRYM (clade 6c)	Avian DHFR (clade 2)	PunC5 (clade 6a)
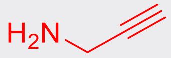 **a**	16	7.726	0.021	–	–	–	–
	**21**	9.794	0.041	–	–	–	–
	**22**	0.266	0.015	–	–	–	–
	**23**	0.052	–	3.975	–	–	–
	**24**	0.029	–	4.326	0.055	0.048	0.133
	**25**	0.041	–	0.216	–	–	–
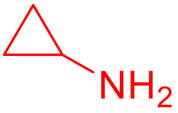 **b**	**16**	10.300	0.034	–	–	–	–
	**21**	11.629	0.034	–	–	–	–
	**22**	0.732	0.012	–	–	–	–
	**23**	0.079	–	4.602	–	–	–
	**24**	0.018	–	4.713	0.039	0.047	0.097
	**25**	0.044	–	0.215	–	–	–
$MeNH_2$ **c**	**16**	6.976	0.025	–	–	–	–
	**21**	1.972	0.013	–	–	–	–
	**22**	0.127	0.005	–	–	–	–
	**23**	0.044	–	4.770	0.022	0.005	0.024
	**24**	–	–	4.752	0.113	0.097	0.168
	**25**	0.031	–	0.015	–	0.023	0.096
NH_3 **d**	**16**	0.434	0.007	–	–	–	–
	**21**	0.324	0.009	–	–	–	–
	**22**	–	–	–	–	–	–
	**23**	–	–	2.799	–	–	–
	**24**	–	–	2.906	–	–	0.028
	**25**	–	–	0.554	–	–	–

N.B. Cells with en dashes mean that activity was not detected under the screening conditions. The following screening conditions were used: reactions contain 5–10 mM ketones, 60 mM amine nucleophile (100 mM for ammonia) added to reaction mixture from 1 M pH adjusted stock solution (pH 9), and 0.5 mM NADPH. The reaction was performed in Tris-HCl buffer (100 mM, pH 9, supplemented with 7.5 mM $MgCl_2$), and 0.05–0.6 mg mL^{-1} IRED was added to start the reaction. The initial reaction rate was monitored at 340 nm using a microtiter plate reader.

IREDs/RedAms.[52] Achieving this transformation remains hugely attractive, as corresponding amine products formed from this reaction are prevalent in pharmaceutical drugs. To this end, we screened 9 members of clade 1 (classical IREDs) for the amination of **27** with methylamine **c**, revealing *Bac*RedAm, *Ma*RedAm, and *Aser*RedAm as the best-performing members of this clade for these substrates.

The amination of 1-indanone with methylamine **c** formed the corresponding α-secondary amine product **27c** with modest conversion values (54%–60%). *Ma*RedAm and *Aser*RedAm

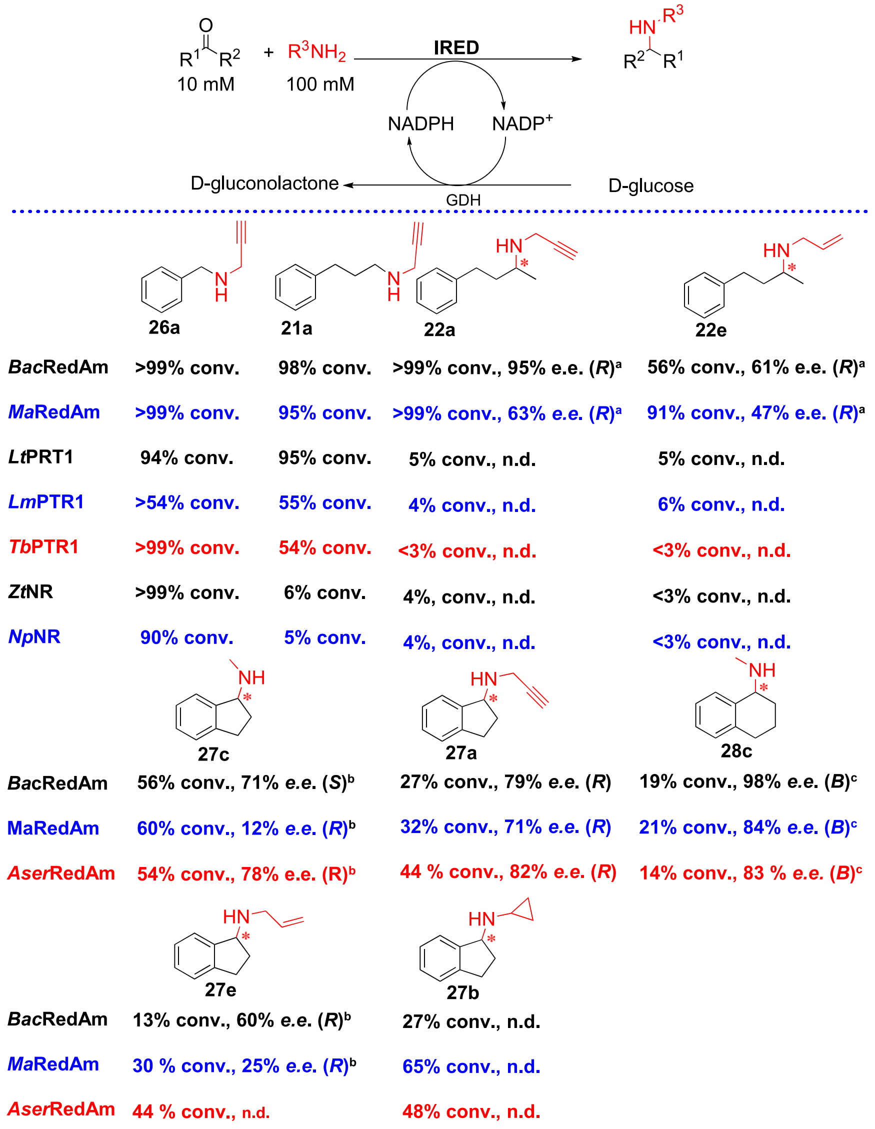

Figure 3. Investigation of the performance of PTR1s, NRs, and classical IREDs for the reductive amination of aromatic ketones and aldehydes

Pteridine reductases (PTR1s) and norcraugsodine reductases (NRs) were able to catalyze the amination of aromatic aldehydes but showed only weak/trace activity toward the amination of aromatic ketones. Novel reductive aminases identified from this work catalyzed the stereoselective reductive amination of aromatic ketones for the synthesis of α-chiral secondary amines. A glucose dehydrogenase (GDH)-based cofactor recycling system was employed, with glucose used as a sacrificial oxidant. Reaction conditions: ketone substrate (10 mM), amine nucleophile (100 mM), 0.5 mM $NAD(P)^+$, 20 mM D-glucose, 0.3 mg mL^{-1} GDH lyophilized cell-free extract, purified RedAm/IRED 1 mg mL^{-1}, 2% v/v DMSO. The reaction was performed in Tris-HCl buffer (100 mM, pH 9) at 30°C and 230 rpm for 24 h. Conversion and enantiomeric excess (*e.e.*) values were determined by normal-phase HPLC analysis using chiral columns.

[a]Absolute configuration assigned based on comparison of the elution pattern of the *N*-methylated analog.

[b]Absolute configurations were assigned based on comparisons of the elution pattern of the *N*-propargyl analog.

[c]Absolute configuration not determined; designation of “A” or “B” represents the order of the elution of the enantiomers on chiral HPLC, with enantiomer “A” eluting first.

produced the (*R*)-**27c** with *e.e.* values of 12% and 78%, respectively, while *Bac*RedAm generated (*S*)-**27c** (71% *e.e.*) (Figure 3; see also supplemental information and Figure S7). Interestingly, *Bac*RedAm-catalyzed reductive amination of **27** with propargylamine **a** afforded the (*R*)-configured amine product rasagaline **27a** (27% conv., 79% *e.e.*), indicating that the amine nucleophile can play a role in the stereochemical outcome of RedAm-catalyzed reductive amination. Both *Ma*RedAm and *Aser*RedAm also afforded rasagiline (*R*)-**27a** (*Ma*RedAm: 32% conv., 71% *e.e.*; *Aser*RedAm: 44% conv., 82% *e.e.*), retaining the selectivity observed with *N*-methylated product **27c** (Figure 3). The RedAm-catalyzed amination with other amine nucleophiles, namely cyclopropylamine **b** and allylamine **e**, yielded the corresponding amine products **27a** and **27e** in moderate conversions of up to 65%. These enzymes also catalyzed the reductive amination of 1-tetralone **28** with methylamine to yield **28c**, a key intermediate of sertraline, albeit in low conversion values (14%–19%) but with good to excellent *e.e.* values of up to 98% (Figure 3).

Conclusion

In summary, the functional exploration of highly divergent, sequence-unrelated enzyme families based on their C=N reducing biosynthetic roles has identified promiscuous catalysts with IRED activity toward non-native substrates. While accidental enzyme discovery in biosynthetic pathways has been the source of many useful catalysts in the past, here we provide a rationale for IRED mining with high hit rates (average: ~46%; successful in 4 of 6 clades) via the substrate promiscuity of C=N reducing enzymes. Specifically, we have mapped and annotated hitherto unexplored sequence space by uncovering distinct, unrelated enzyme families for prospecting biocatalysts for enantioselective imine reduction and reductive amination. Clade 3, which comprised PTR1s and alkaloid-forming plants and bacterial SDRs, represents a unique and promising functional annotation of sequence space to find novel enzymes for enantioselective imine reduction and reductive amination with a hit rate >87% for imine-reducing activity for this clade. We showed that members of PTR1s and NRs were able to catalyze the reductive amination of simple cyclic ketones and aromatic aldehydes.

Clade 6 is another promising group to retrieve novel IRED-like biocatalysts; members of this group such as the mammalian ketimine reductase mu-crystallin (CRYM) and iminosuccinate reductases (e.g., *Pb*BhCD) exhibit promiscuous (albeit weak) imine-reducing activity toward non-native cyclic imines, e.g., isoquinoline imine. Importantly, clade 6 also features NatAmDHs, which have been shown to catalyze the IRED-like reductive amination reaction.[7] Our approach allows the identification of alternative sets of evolutionarily unrelated, non-homologous "isofunctional" enzymes.

A comparative evaluation of the performance of these non-homologous enzyme families in the reductive amination of carbonyl compounds with primary amines using a small panel of carbonyl compounds including ketones, aldehydes, α-keto esters, and α-keto acids highlights differences in substrate specificity. Classical IREDs emerged as the most versatile enzyme class, displaying activity across all the investigated substrate groups and enabling the synthesis of α-secondary amines from difficult-to-aminate aromatic ketones.

Several of the other enzyme classes characterized in this work act on native substrates that can be considered large and hydrophobic, providing spacious hydrophobic binding sites, which should warrant future evaluation of their usefulness in the reductive amination of bulky substrates. Even more desirable is an extensive substrate profiling study using large and structurally diverse carbonyl and amine substrate panels to map out the unique synthetic scope of each of these diverse enzyme families. Catalytic efficiency can further be optimized through protein engineering to provide a versatile toolbox for biocatalytic imine reduction and reductive amination of challenging substrates. In this context, sequence network models[53] and convolutional neural networks using deep learning models[54] can serve as useful tools to investigate the sequence and structural features peculiar and common to these divergent enzyme families. Such studies may provide useful mechanistic insights into enzyme promiscuity to allow (semi)-rational enzyme engineering of these enzymes.

EXPERIMENTAL PROCEDURES

Materials and methods

For more details on the experimental procedures, including materials and chemicals, procedures for the synthesis of chemical standards, procedures for cloning, enzyme expression and purification, and biotransformation reactions, see the supplemental information.

RESOURCE AVAILABILITY

Lead contact

Requests for further information and resources should be directed to and will be fulfilled by the lead contact, Godwin Aleku (godwin.aleku@kcl.ac.uk).

Materials availability

All materials generated in this study are available from the lead contact without restriction.

Data and code availability

This study did not generate any datasets.

ACKNOWLEDGMENTS

This work received support from the Leverhulme Trust through a Leverhulme Early Career Fellowship to G.A.A. (ECF-2020-694). G.A.A. also received support through the Isaac Newton Trust Early Career Fellowship and The Royal Society (RGS\R1\231514). Further support was provided by the BBSRC (BB/T003545/1). F.H. is an ERC Advanced Investigator (695669) and member of the EU Horizon consortium BlueRemediomics (101082304) with support from UKRI. The authors would like to thank Dr. Melanie A. Higgins for kindly providing us with Bsp5 and Punc5 plasmids and Dr. Liisa van Vliet for kindly providing us with the *Pv*DHFR and *Pf*DHFR plasmids.

AUTHOR CONTRIBUTIONS

G.A.A. conceived and designed the experiments with input and guidance from F.H. G.A.A. performed all experiments and analyzed the data. G.A.A. and F.H. wrote the paper.

DECLARATION OF INTERESTS

The authors declare no competing interests.

SUPPLEMENTAL INFORMATION

Supplemental information can be found online at https://doi.org/10.1016/j.checat.2024.101160.

Received: April 10, 2024
Revised: August 11, 2024
Accepted: September 25, 2024
Published: October 24, 2024

REFERENCES

1. Aleku, G.A., France, S.P., Man, H., Mangas-Sanchez, J., Montgomery, S.L., Sharma, M., Leipold, F., Hussain, S., Grogan, G., and Turner, N.J. (2017). A reductive aminase from *Aspergillus oryzae*. Nat. Chem. *9*, 961–969. https://doi.org/10.1038/nchem.2782.
2. Gilio, A.K., Thorpe, T.W., Turner, N., and Grogan, G. (2022). Reductive aminations by imine reductases: from milligrams to tons. Chem. Sci. *13*, 4697–4713. https://doi.org/10.1039/D2SC00124A.
3. Aleku, G.A., Titchiner, G.R., Roberts, G.W., Derrington, S.R., Marshall, J.R., Hollfelder, F., Turner, N.J., and Leys, D. (2022). Enzymatic N-Allylation of Primary and Secondary Amines Using Renewable Cinnamic Acids Enabled by Bacterial Reductive Aminases. ACS Sustain. Chem. Eng. *10*, 6794–6806. https://doi.org/10.1021/acssuschemeng.2c01180.
4. Ramsden, J.I., Heath, R.S., Derrington, S.R., Montgomery, S.L., Mangas-Sanchez, J., Mulholland, K.R., and Turner, N.J. (2019). Biocatalytic N-Alkylation of Amines Using Either Primary Alcohols or Carboxylic Acids via Reductive Aminase Cascades. J. Am. Chem. Soc. *141*, 1201–1206. https://doi.org/10.1021/jacs.8b11561.
5. Slabu, I., Galman, J.L., Lloyd, R.C., and Turner, N.J. (2017). Discovery, Engineering, and Synthetic Application of Transaminase Biocatalysts. ACS Catal. *7*, 8263–8284. https://doi.org/10.1021/acscatal.7b02686.
6. Afanasyev, O.I., Kuchuk, E., Usanov, D.L., and Chusov, D. (2019). Reductive Amination in the Synthesis of Pharmaceuticals. Chem. Rev. *119*, 11857–11911. https://doi.org/10.1021/acs.chemrev.9b00383.
7. Schober, M., MacDermaid, C., Ollis, A.A., Chang, S., Khan, D., Hosford, J., Latham, J., Ihnken, L.A.F., Brown, M.J.B., Fuerst, D., et al. (2019). Chiral synthesis of LSD1 inhibitor GSK2879552 enabled by directed evolution of an imine reductase. Nat. Catal. *2*, 909–915. https://doi.org/10.1038/s41929-019-0341-4.
8. Kumar, R., Karmilowicz, M.J., Burke, D., Burns, M.P., Clark, L.A., Connor, C.G., Cordi, E., Do, N.M., Doyle, K.M., Hoagland, S., et al. (2021). Biocatalytic reductive amination from discovery to commercial manufacturing

applied to abrocitinib JAK1 inhibitor. Nat. Catal. *4*, 775–782. https://doi.org/10.1038/s41929-021-00671-5.

9. Ma, E.J., Siirola, E., Moore, C., Kummer, A., Stoeckli, M., Faller, M., Bouquet, C., Eggimann, F., Ligibel, M., Huynh, D., et al. (2021). Machine-Directed Evolution of an Imine Reductase for Activity and Stereoselectivity. ACS Catal. *11*, 12433–12445. https://doi.org/10.1021/acscatal.1c02786.

10. Duan, S., Widlicka, D.W., Burns, M.P., Kumar, R., Hotham, I., Desrosiers, J.-N., Bowles, P., Jones, K.N., Nicholson, L.D., Buetti-Weekly, M.T., et al. (2022). Application of Biocatalytic Reductive Amination for the Synthesis of a Key Intermediate to a CDK 2/4/6 Inhibitor. Org. Process Res. Dev. *26*, 879–890. https://doi.org/10.1021/acs.oprd.1c00255.

11. Montgomery, S.L., Pushpanath, A., Heath, R.S., Marshall, J.R., Klemstein, U., Galman, J.L., Woodlock, D., Bisagni, S., Taylor, C.J., Mangas-Sanchez, J., et al. (2020). Characterization of imine reductases in reductive amination for the exploration of structure-activity relationships. Sci. Adv. *6*, eaay9320. https://doi.org/10.1126/sciadv.aay9320.

12. Matzel, P., Krautschick, L., and Höhne, M. (2017). Photometric Characterization of the Reductive Amination Scope of the Imine Reductases from Streptomyces tsukubaensis and Streptomyces ipomoeae. Chembiochem *18*, 2022–2027. https://doi.org/10.1002/cbic.201700257.

13. Mitsukura, K., Suzuki, M., Tada, K., Yoshida, T., and Nagasawa, T. (2010). Asymmetric synthesis of chiral cyclic amine from cyclic imine by bacterial whole-cell catalyst of enantioselective imine reductase. Org. Biomol. Chem. *8*, 4533–4535. https://doi.org/10.1039/C0OB00353K.

14. Mitsukura, K., Suzuki, M., Shinoda, S., Kuramoto, T., Yoshida, T., and Nagasawa, T. (2011). Purification and Characterization of a Novel (*R*)-Imine Reductase from *Streptomyces* sp. Biosci. Biotechnol. Biochem. *75*, 1778–1782. https://doi.org/10.1271/bbb.110303.

15. Mitsukura, K., Kuramoto, T., Yoshida, T., Kimoto, N., Yamamoto, H., and Nagasawa, T. (2013). A NADPH-dependent (S)-imine reductase (SIR) from Streptomyces sp. GF3546 for asymmetric synthesis of optically active amines: purification, characterization, gene cloning, and expression. Appl. Microbiol. Biotechnol. *97*, 8079–8086. https://doi.org/10.1007/s00253-012-4629-4.

16. France, S.P., Howard, R.M., Steflik, J., Weise, N.J., Mangas-Sanchez, J., Montgomery, S.L., Crook, R., Kumar, R., and Turner, N.J. (2018). Identification of Novel Bacterial Members of the Imine Reductase Enzyme Family that Perform Reductive Amination. ChemCatChem *10*, 510–514. https://doi.org/10.1002/cctc.201701408.

17. Li, H., Luan, Z.-J., Zheng, G.-W., and Xu, J.-H. (2015). Efficient Synthesis of Chiral Indolines using an Imine Reductase from Paenibacillus lactis. Adv. Synth. Catal. *357*, 1692–1696. https://doi.org/10.1002/adsc.201500160.

18. Roiban, G.-D., Kern, M., Liu, Z., Hyslop, J., Tey, P.L., Levine, M.S., Jordan, L.S., Brown, K.K., Hadi, T., Ihnken, L.A.F., and Brown, M.J.B. (2017). Efficient Biocatalytic Reductive Aminations by Extending the Imine Reductase Toolbox. ChemCatChem *9*, 4475–4479. https://doi.org/10.1002/cctc.201701379.

19. Roth, S., Präg, A., Wechsler, C., Marolt, M., Ferlaino, S., Lüdeke, S., Sandon, N., Wetzl, D., Iding, H., Wirz, B., et al. (2017). Extended Catalytic Scope of a Well-Known Enzyme: Asymmetric Reduction of Iminium Substrates by Glucose Dehydrogenase. Chembiochem Eur. J. Chem. Biol *18*, 1703–1706. https://doi.org/10.1002/cbic.201700261.

20. Scheller, P.N., Fademrecht, S., Hofelzer, S., Pleiss, J., Leipold, F., Turner, N.J., Nestl, B.M., and Hauer, B. (2014). Enzyme Toolbox: Novel Enantiocomplementary Imine Reductases. Chembiochem *15*, 2201–2204. https://doi.org/10.1002/cbic.201402213.

21. Wetzl, D., Berrera, M., Sandon, N., Fishlock, D., Ebeling, M., Müller, M., Hanlon, S., Wirz, B., and Iding, H. (2015). Expanding the Imine Reductase Toolbox by Exploring the Bacterial Protein-Sequence Space. Chembiochem *16*, 1749–1756. https://doi.org/10.1002/cbic.201500218.

22. Yao, P., Xu, Z., Yu, S., Wu, Q., and Zhu, D. (2019). Imine Reductase-Catalyzed Enantioselective Reduction of Bulky α,β-Unsaturated Imines en Route to a Pharmaceutically Important Morphinan Skeleton. Adv. Synth. Catal. *361*, 556–561. https://doi.org/10.1002/adsc.201801326.

23. Fademrecht, S., Scheller, P.N., Nestl, B.M., Hauer, B., and Pleiss, J. (2016). Identification of imine reductase-specific sequence motifs. Proteins *84*, 600–610.

24. Caparco, A.A., Pelletier, E., Petit, J.L., Jouenne, A., Bommarius, B.R., de Berardinis, V., Zaparucha, A., Champion, J.A., Bommarius, A.S., and Vergne-Vaxelaire, C. (2020). Metagenomic Mining for Amine Dehydrogenase Discovery. Adv. Synth. Catal. *362*, 2427–2436. https://doi.org/10.1002/adsc.202000094.

25. Marshall, J.R., Yao, P., Montgomery, S.L., Finnigan, J.D., Thorpe, T.W., Palmer, R.B., Mangas-Sanchez, J., Duncan, R.A.M., Heath, R.S., Graham, K.M., et al. (2021). Screening and characterization of a diverse panel of metagenomic imine reductases for biocatalytic reductive amination. Nat. Chem. *13*, 140–148. https://doi.org/10.1038/s41557-020-00606-w.

26. Stockinger, P., Schelle, L., Schober, B., Buchholz, P.C.F., Pleiss, J., and Nestl, B.M. (2020). Engineering of Thermostable β-Hydroxyacid Dehydrogenase for the Asymmetric Reduction of Imines. Chembiochem *21*, 3511–3514. https://doi.org/10.1002/cbic.202000526.

27. Roth, S., Kilgore, M.B., Kutchan, T.M., and Müller, M. (2018). Exploiting the Catalytic Diversity of Short-Chain Dehydrogenases/Reductases: Versatile Enzymes from Plants with Extended Imine Substrate Scope. Chembiochem *19*, 1849–1852. https://doi.org/10.1002/cbic.201800291.

28. Guo, J., Higgins, M.A., Daniel-Ivad, P., and Ryan, K.S. (2019). An Asymmetric Reductase That Intercepts Acyclic Imino Acids Produced in Situ by a Partner Oxidase. J. Am. Chem. Soc. *141*, 12258–12267. https://doi.org/10.1021/jacs.9b03307.

29. Mangas-Sanchez, J., France, S.P., Montgomery, S.L., Aleku, G.A., Man, H., Sharma, M., Ramsden, J.I., Grogan, G., and Turner, N.J. (2017). Imine reductases (IREDs). Curr. Opin. Chem. Biol. *37*, 19–25. https://doi.org/10.1016/j.cbpa.2016.11.022.

30. Schrittwieser, J.H., Velikogne, S., and Kroutil, W. (2015). Biocatalytic Imine Reduction and Reductive Amination of Ketones. Adv. Synth. Catal. *357*, 1655–1685. https://doi.org/10.1002/adsc.201500213.

31. Zumbrägel, N., Merten, C., Huber, S.M., and Gröger, H. (2018). Enantioselective reduction of sulfur-containing cyclic imines through biocatalysis. Nat. Commun. *9*, 1949. https://doi.org/10.1038/s41467-018-03841-5.

32. Babtie, A., Tokuriki, N., and Hollfelder, F. (2010). What makes an enzyme promiscuous? Curr. Opin. Chem. Biol. *14*, 200–207. https://doi.org/10.1016/j.cbpa.2009.11.028.

33. Bornscheuer, U.T., and Kazlauskas, R.J. (2004). Catalytic Promiscuity in Biocatalysis: Using Old Enzymes to Form New Bonds and Follow New Pathways. Angew. Chem. Int. Ed. *43*, 6032–6040. https://doi.org/10.1002/anie.200460416.

34. Tawfik, O.K., and S, D. (2010). Enzyme Promiscuity: A Mechanistic and Evolutionary Perspective. Annu. Rev. Biochem. *79*, 471–505. https://doi.org/10.1146/annurev-biochem-030409-143718.

35. Farrow, S.C., Hagel, J.M., Beaudoin, G.A.W., Burns, D.C., and Facchini, P.J. (2015). Stereochemical inversion of (S)-reticuline by a cytochrome P450 fusion in opium poppy. Nat. Chem. Biol. *11*, 728–732. https://doi.org/10.1038/nchembio.1879.

36. Kilgore, M.B., Holland, C.K., Jez, J.M., and Kutchan, T.M. (2016). Identification of a Noroxomaritidine Reductase with Amaryllidaceae Alkaloid Biosynthesis Related Activities. J. Biol. Chem. *291*, 16740–16752. https://doi.org/10.1074/jbc.M116.717827.

37. Matuschek, M., Wallwey, C., Xie, X., and Li, S.-M. (2011). New insights into ergot alkaloid biosynthesis in Claviceps purpurea: an agroclavine synthase EasG catalyses, via a non-enzymatic adduct with reduced glutathione, the conversion of chanoclavine-I aldehyde to agroclavine. Org. Biomol. Chem. *9*, 4328–4335. https://doi.org/10.1039/c0ob01215g.

38. Stavrinides, A., Tatsis, E.C., Foureau, E., Caputi, L., Kellner, F., Courdavault, V., and O'Connor, S.E. (2015). Unlocking the Diversity of Alkaloids in Catharanthus roseus: Nuclear Localization Suggests Metabolic

Channeling in Secondary Metabolism. Chem. Biol. *22*, 336–341. https://doi.org/10.1016/j.chembiol.2015.02.006.

39. Vogel, M., Lawson, M., Sippl, W., Conrad, U., and Roos, W. (2010). Structure and Mechanism of Sanguinarine Reductase, an Enzyme of Alkaloid Detoxification. J. Biol. Chem. *285*, 18397–18406. https://doi.org/10.1074/jbc.M109.088989.

40. Wen, W.-H., Zhang, Y., Zhang, Y.-Y., Yu, Q., Jiang, C.-C., Tang, M.-C., Pu, J.-Y., Wu, L., Zhao, Y.-L., Shi, T., et al. (2021). Reductive inactivation of the hemiaminal pharmacophore for resistance against tetrahydroisoquinoline antibiotics. Nat. Commun. *12*, 7085. https://doi.org/10.1038/s41467-021-27404-3.

41. Schada von Borzyskowski, L., Severi, F., Krüger, K., Hermann, L., Gilardet, A., Sippel, F., Pommerenke, B., Claus, P., Cortina, N.S., Glatter, T., et al. (2019). Marine Proteobacteria metabolize glycolate via the β-hydroxyaspartate cycle. Nature *575*, 500–504. https://doi.org/10.1038/s41586-019-1748-4.

42. Hallen, A., Cooper, A.J.L., Smith, J.R., Jamie, J.F., and Karuso, P. (2015). Ketimine reductase/CRYM catalyzes reductive alkylamination of α-keto acids, confirming its function as an imine reductase. Amino Acids *47*, 2457–2461. https://doi.org/10.1007/s00726-015-2044-8.

43. Muramatsu, H., Mihara, H., Kakutani, R., Yasuda, M., Ueda, M., Kurihara, T., and Esaki, N. (2005). The putative malate/lactate dehydrogenase from Pseudomonas putida is an NADPH-dependent delta1-piperideine-2-carboxylate/delta1-pyrroline-2-carboxylate reductase involved in the catabolism of D-lysine and D-proline. J. Biol. Chem. *280*, 5329–5335. https://doi.org/10.1074/jbc.M411918200.

44. Uma Mahesh, V.N.M., and Chadha, A. (2021). Imine reduction by an Ornithine cyclodeaminase/μ-crystallin homolog purified from Candida parapsilosis ATCC 7330. Biotechnol. Rep. *31*, e00664. https://doi.org/10.1016/j.btre.2021.e00664.

45. Chang, F.-Y., Ternei, M.A., Calle, P.Y., and Brady, S.F. (2015). Targeted Metagenomics: Finding Rare Tryptophan Dimer Natural Products in the Environment. J. Am. Chem. Soc. *137*, 6044–6052. https://doi.org/10.1021/jacs.5b01968.

46. Zallot, R., Oberg, N., and Gerlt, J.A. (2019). The EFI Web Resource for Genomic Enzymology Tools: Leveraging Protein, Genome, and Metagenome Databases to Discover Novel Enzymes and Metabolic Pathways. Biochemistry *58*, 4169–4182. https://doi.org/10.1021/acs.biochem.9b00735.

47. Shannon, P., Markiel, A., Ozier, O., Baliga, N.S., Wang, J.T., Ramage, D., Amin, N., Schwikowski, B., and Ideker, T. (2003). Cytoscape: A Software Environment for Integrated Models of Biomolecular Interaction Networks. Genome Res. *13*, 2498–2504. https://doi.org/10.1101/gr.1239303.

48. Mayol, O., Bastard, K., Beloti, L., Frese, A., Turkenburg, J.P., Petit, J.-L., Mariage, A., Debard, A., Pellouin, V., Perret, A., et al. (2019). A family of native amine dehydrogenases for the asymmetric reductive amination of ketones. Nat. Catal. *2*, 324–333. https://doi.org/10.1038/s41929-019-0249-z.

49. Aleku, G.A., Man, H., France, S.P., Leipold, F., Hussain, S., Toca-Gonzalez, L., Marchington, R., Hart, S., Turkenburg, J.P., Grogan, G., and Turner, N.J. (2016). Stereoselectivity and Structural Characterization of an Imine Reductase (IRED) from Amycolatopsis orientalis. ACS Catal. *6*, 3880–3889. https://doi.org/10.1021/acscatal.6b00782.

50. Aleku, G.A., Saaret, A., Bradshaw-Allen, R.T., Derrington, S.R., Titchiner, G.R., Gostimskaya, I., Gahloth, D., Parker, D.A., Hay, S., and Leys, D. (2020). Enzymatic C–H activation of aromatic compounds through CO 2 fixation. Nat. Chem. Biol. *16*, 1255–1260. https://doi.org/10.1038/s41589-020-0603-0.

51. Aleku, G.A., Roberts, G.W., Titchiner, G.R., and Leys, D. (2021). Synthetic Enzyme-Catalyzed CO2 Fixation Reactions. ChemSusChem *14*, 1781–1804. https://doi.org/10.1002/cssc.202100159.

52. Aleku, G.A. (2024). Imine Reductases and Reductive Aminases in Organic Synthesis. ACS Catal *14*, 14308–14329. https://doi.org/10.1021/acscatal.4c04756.

53. Copp, J.N., Anderson, D.W., Akiva, E., Babbitt, P.C., and Tokuriki, N. (2019). Chapter Twelve - Exploring the sequence, function, and evolutionary space of protein superfamilies using sequence similarity networks and phylogenetic reconstructions. In Methods in Enzymology New Approaches for Flavin Catalysis, B.A. Palfey, ed. (Academic Press), pp. 315–347. https://doi.org/10.1016/bs.mie.2019.03.015.

54. Taujale, R., Zhou, Z., Yeung, W., Moremen, K.W., Li, S., and Kannan, N. (2021). Mapping the glycosyltransferase fold landscape using interpretable deep learning. Nat. Commun. *12*, 5656. https://doi.org/10.1038/s41467-021-25975-9.

Chem Catalysis, Volume *4*

Supplemental information

Expanding the repertoire of imine reductases by mining divergent biosynthetic pathways for promiscuous reactivity

Godwin A. Aleku and Florian Hollfelder

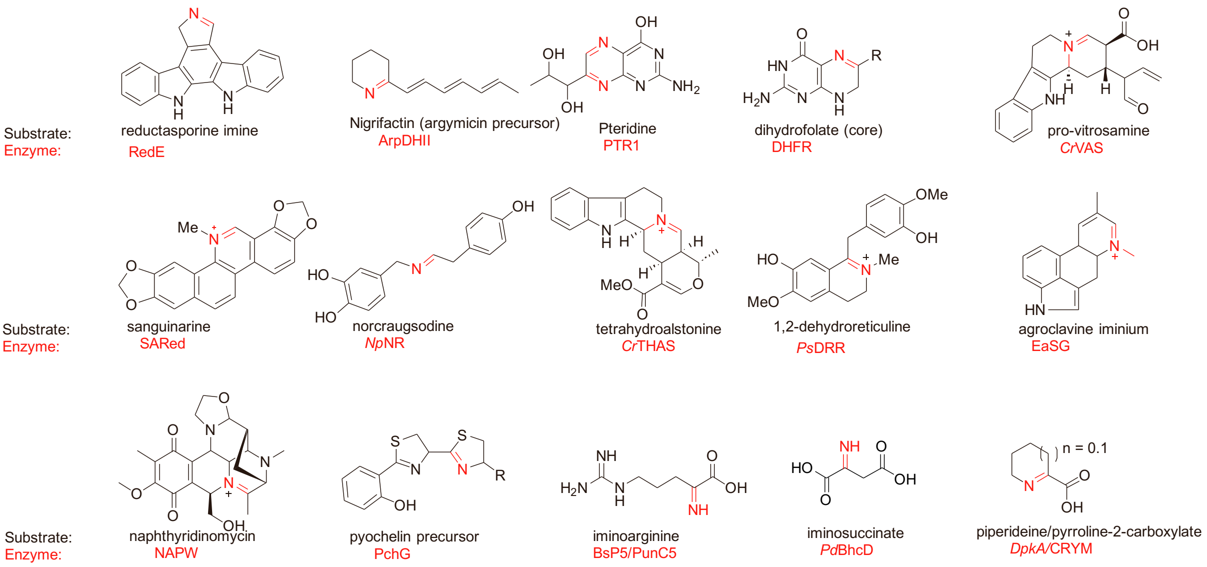

Figure S1. Examples of structurally diverse native imine/iminium substrates and their respective biosynthetic C=N reducing enzymes. RedE, tryptophan dimer biosynthetic enzyme; ArpDHII, Argimycins P dehydrogenase; PTR1, pteridine reductase 1; DHFR, dihydrofolate reductase; *Cr*VAS vitrosamine synthase from *Catharanthus roseus*; SaRed, sanguinarine reductase; NpNR noroxomaritidine/norcraugsodine reductase from *Narcissus pseudonarcissus*; *Cr*THAS, tetrahydroalstonine synthase from C. roseus; *Ps*DRR, 1,2-dehydroreticuline reductase from *Papaver sp*; EaSG agroclavine synthase; NAPW, naphthyridinomycin dehydrogenase; PchG, pyochelin biosynthesis thiazoline reductase; BsP5/PunC5 acyclic imino acid reductase; *Pb*BhcD, Imminosuccinate reductase from *Paracoccus denitriicans.* DpkA/CRYM-ketimine reductase.

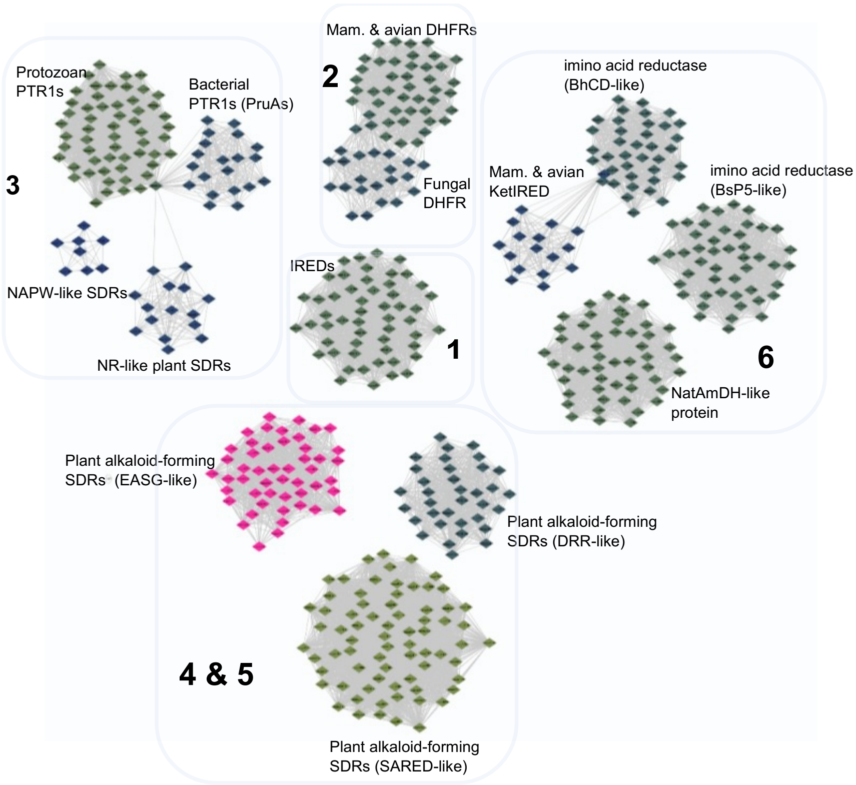

Figure S2. Sequence similarity networks of biosynthetic C=N reducing enzymes from different functional families. Networks were generated using the Enzyme Similarity Tool (EFI-EST)[1] and visualised with Cytoscape.[2] Edges displayed correspond to a median of 27% identity over an alignment length of 350 amino acids. Representatives' clades. IREDs, imine reductases; DHFR, dihydrofolate reductase; PTR1, pteridine reductases; PruAs, bacterial pteridine reductase; SDR, short-chain dehydrogenase/reductase; KetIRED, ketimine reductases/CRYMs; NR, *norcraugsodine reductase, NAPW,* naphthyridinomycin dehydrogenase; NatAmDH, native amine dehydrogenase; DRR, dihydroreticuline reductase; EaSG, agroclavine synthase (ergot alkaloid biosynthesis). A comprehensive list of the enzymes included in this study is presented in Table S1 and a phylogenetic tree (cladogram) is presented in main manuscript, Figure 2.

		IREDs						DHFRs							
		ArpDHII	RedE	SeIRED	BacRedAm	MaRedAm	AserRedam	TbDHFR	PjDHFR	hDHFR	GgDHFR	SaDHFR	PvDHFR		
IREDs	ArpDHII	100	39	43	42	36	39	16	15	13	13	16	12	ArpDHII	IREDs
	RedE	39	100	45	44	34	35	12	12	14	14	16	13	RedE	
	SeIRED	43	45	100	45	37	38	15	13	13	13	15	11	SeIRED	
	BacRedAm	42	44	45	100	53	51	14	16	16	16	14	10	BacRedAm	
	MaRedAm	36	34	37	53	100	53	16	13	16	16	16	12	MaRedAm	
	AserRedam	39	35	38	51	53	100	15	10	13	13	15	10	AserRedam	
DHFRs	TbDHFR	16	12	15	14	16	15	100	30	31	32	27	24	TbDHFR	DHFRs
	PjDHFR	15	12	13	16	13	10	30	100	36	36	31	27	PjDHFR	
	hDHFR	13	14	13	16	16	13	31	36	100	75	30	31	hDHFR	
	GgDHFR	13	14	13	16	16	13	32	36	75	100	31	30	GgDHFR	
	SaDHFR	16	16	15	14	16	15	27	31	30	31	100	32	SaDHFR	
	PvDHFR	12	13	11	10	12	10	24	27	31	30	32	100	PvDHFR	
		ArpDHII	RedE	SeIRED	BacRedAm	MaRedAm	AserRedam	TbDHFR	PjDHFR	hDHFR	GgDHFR	SaDHFR	PvDHFR		
		IREDs						DHFRs							

Figure S3. Percent sequence identity matrix comparing classical imine reductases (IREDs)/reductive aminases (RedAms) with dihydrofolate reductases (DHFRs, clade 2). The percent identity matrix was generated using ClustalW.

		IREDS							NAPW-like SDRs			NR-like SDRs						PTR1s				
		ArpDHII	RedE	SsIRED	SeIRED	BacRedAm	MaRedAm	AserRedam	MbSDR	PbSDR	NAPW	LsNR	NpaNR	LaNR	LrNR	NpNR	ZtNR	AmPTR1	LtPTR1	LmPTR1	TbPTR1	TcPTR1
IREDS	ArpDHII	100	38	40	42	41	35	38	12	12	12	11	12	12	12	11	17	15	15	14	11	16
	RedE	38	100	43	45	43	33	34	16	14	15	13	10	11	10	11	12	14	14	12	13	14
	SsIRED	40	43	100	57	46	39	38	14	12	12	12	10	10	12	12	11	15	14	13	15	16
	SeIRED	42	45	57	100	45	37	38	12	10	10	11	11	12	13	12	12	14	13	12	11	16
	BacRedAm	41	43	46	45	100	53	50	14	11	11	16	12	12	11	11	13	17	15	14	14	17
	MaRedAm	35	33	39	37	53	100	52	14	12	12	11	11	12	11	12	12	17	14	11	14	15
	AserRedam	38	34	38	38	50	52	100	10	11	12	13	14	14	12	13	14	14	13	12	14	14
NAPW-like SDRs	MbSDR	12	16	14	12	14	14	10	100	55	51	22	23	24	22	24	23	22	21	20	19	21
	PbSDR	12	14	12	10	11	12	11	55	100	74	23	22	23	22	23	22	21	21	21	21	21
	NAPW	12	15	12	10	11	12	12	51	74	100	22	21	22	22	22	22	21	19	19	20	19
NR-like SDRs	LsNR	11	13	12	11	16	11	13	22	23	22	100	45	46	**45**	48	49	32	27	28	29	26
	NpaNR	12	10	10	11	12	11	14	23	22	21	45	100	90	70	75	70	33	27	26	29	26
	LaNR	12	11	10	12	12	12	14	24	23	22	46	90	100	71	73	68	33	27	26	32	25
	LrNR	12	10	12	13	11	11	12	22	22	22	45	70	71	100	83	75	34	27	24	27	23
	NpNR	11	11	12	12	11	12	13	24	23	22	48	75	73	83	100	85	36	27	26	30	26
	ZtNR	17	12	11	12	13	12	14	23	22	22	49	70	68	75	85	100	36	29	27	30	26
PTR1s	AmPTR1	15	14	15	14	17	17	14	22	21	21	32	33	33	34	36	36	100	45	44	44	41
	LtPTR1	15	14	14	13	15	14	13	21	21	19	27	27	27	27	27	29	45	100	80	52	48
	LmPTR1	14	12	13	12	14	11	12	20	21	19	28	26	26	24	26	27	44	80	100	51	48
	TbPTR1	11	13	15	11	14	14	14	19	21	20	29	29	32	27	30	30	44	52	51	100	56
	TcPTR1	16	14	16	16	17	15	14	21	21	19	26	26	25	23	26	26	41	48	48	56	100

Figure S4. Percent sequence identity matrix comparing classical imine reductases (IREDs)/reductive aminases (RedAms) with members of clade 3 (pteridine reductases, PTR1s; naphthyridinomycin dehydrogenases, NAPW; norcraugsodine reductases, NRs. The percent identity matrix was generated using ClustalW.

		CRYMs							BHCDs									IREDs									
		GcCRYM	SvCRYM	AcCRYM	CmCRYM	RnCRYM	CRYM	BtCRYM	VsBHCD	GaBhCD	PaBhCD	PsBHCD	OtBhCD	AeBhCD	RbBOCD	LSBhCD	RaBhCD	MaRedAm	BacRedAm	AdRedAm	AtRedAm	AspRedAm	AsRedam	SeIRED	AoIRED	SpRedAm	RedE
CRYMs	GcCRYM	100	91	91	92	80	80	79	29	30	27	28	27	28	27	29	29	10	11	10	12	13	13	14	14	14	10
	SvCRYM	91	100	94	95	79	80	78	29	29	27	29	27	28	27	29	30	10	11	11	11	13	15	14	13	14	11
	AcCRYM	91	94	100	98	77	78	78	28	28	27	28	26	27	27	29	29	10	11	11	12	13	14	14	13	14	11
	CmCRYM	92	95	98	100	81	80	81	29	29	28	29	27	28	28	30	30	10	11	11	12	13	13	14	13	14	11
	RnCRYM	80	79	77	81	100	88	89	28	27	26	27	25	26	27	27	27	10	10	10	11	13	13	13	12	12	10
	HsCRYM	80	80	78	80	88	100	90	27	26	26	27	25	25	26	27	27	10	11	10	12	13	13	11	14	12	10
	BtCRYM	79	78	78	81	89	90	100	27	26	26	26	24	25	27	27	27	10	11	10	11	12	12	12	13	12	10
BHCDs	VsBHCD	29	29	28	29	28	27	27	100	42	38	39	38	39	39	38	38	12	11	12	13	14	14	13	13	14	12
	GaBhCD	30	29	28	29	27	26	26	42	100	61	61	60	66	64	63	62	12	14	12	14	13	13	13	13	15	11
	PaBhCD	27	27	27	28	26	26	26	38	61	100	79	64	70	69	70	70	13	13	12	13	13	12	13	13	15	12
	PsBHCD	28	29	28	29	27	27	26	39	61	79	100	63	68	68	68	68	13	14	14	14	14	14	14	16	16	12
	OtBhCD	27	27	26	27	25	25	24	38	60	64	63	100	72	67	67	66	10	14	12	11	11	11	11	11	12	10
	AeBhCD	28	28	27	28	26	25	25	39	66	70	68	72	100	74	71	70	13	15	13	15	14	13	12	12	16	11
	RbBOCD	27	27	27	28	27	26	27	39	64	69	68	67	74	100	75	74	12	13	12	13	12	12	14	11	15	11
	LSBhCD	29	29	29	30	27	27	27	38	63	70	68	67	71	75	100	99	12	12	12	11	13	13	12	14	14	12
	RaBhCD	29	30	29	30	27	27	27	38	62	70	68	66	70	74	99	100	12	12	12	12	14	14	13	14	14	12
IREDs	MaRedAm	10	10	10	10	10	10	10	12	12	13	13	10	13	12	12	12	100	53	50	54	51	53	37	38	39	34
	BacRedAm	11	11	11	11	10	11	11	11	14	13	14	14	15	13	12	12	53	100	53	54	50	50	45	45	49	45
	AdRedAm	10	11	11	11	10	10	10	12	12	12	14	12	13	12	12	12	50	53	100	54	52	54	39	41	39	38
	AtRedAm	12	11	12	12	11	12	11	13	14	13	14	11	15	13	11	12	54	54	54	100	59	60	42	42	42	37
	AspRedAm	13	13	13	13	13	13	12	14	13	13	14	11	14	12	13	14	51	50	52	59	100	92	37	36	37	34
	AsRedam	13	15	14	13	13	13	12	14	13	12	14	11	13	12	13	14	53	50	54	60	92	100	37	38	37	35
	SeIRED	14	14	14	14	13	11	12	13	13	13	14	11	12	14	12	13	37	45	39	42	37	37	100	57	49	46
	AoIRED	14	13	13	13	12	14	13	13	13	13	16	11	12	11	14	14	38	45	41	42	36	38	57	100	48	44
	SpRedAm	14	14	14	14	12	12	12	14	15	15	16	12	16	15	14	14	39	49	39	42	37	37	49	48	100	55
	RedE	10	11	11	11	10	10	10	12	11	12	12	10	11	11	12	12	34	45	38	37	34	35	46	44	55	100

Figure S5. Percent sequence identity matrix comparing classical imine reductases (IREDs)/reductive aminases (RedAms) against members of clade 6c (imino acid reductases, CRYMs and BhCD-like enzyme). The percent identity matrix was generated using ClustalW.

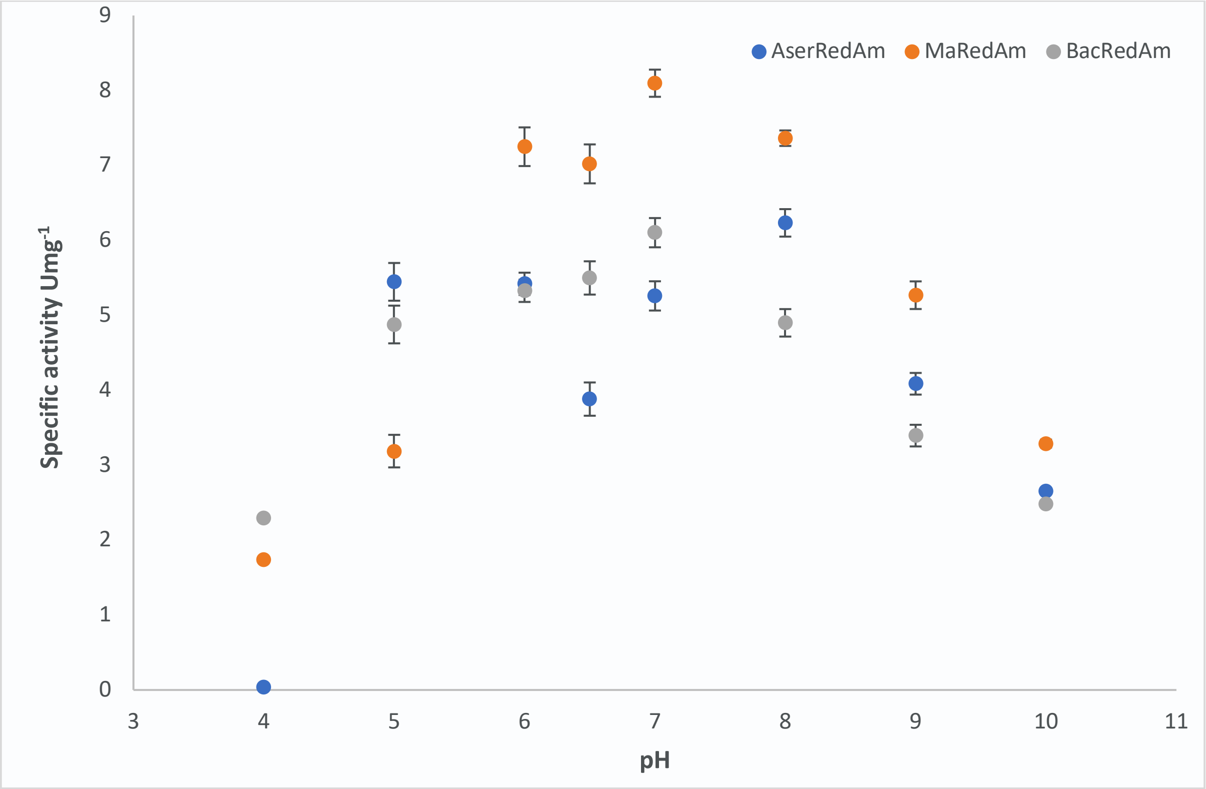

(b)

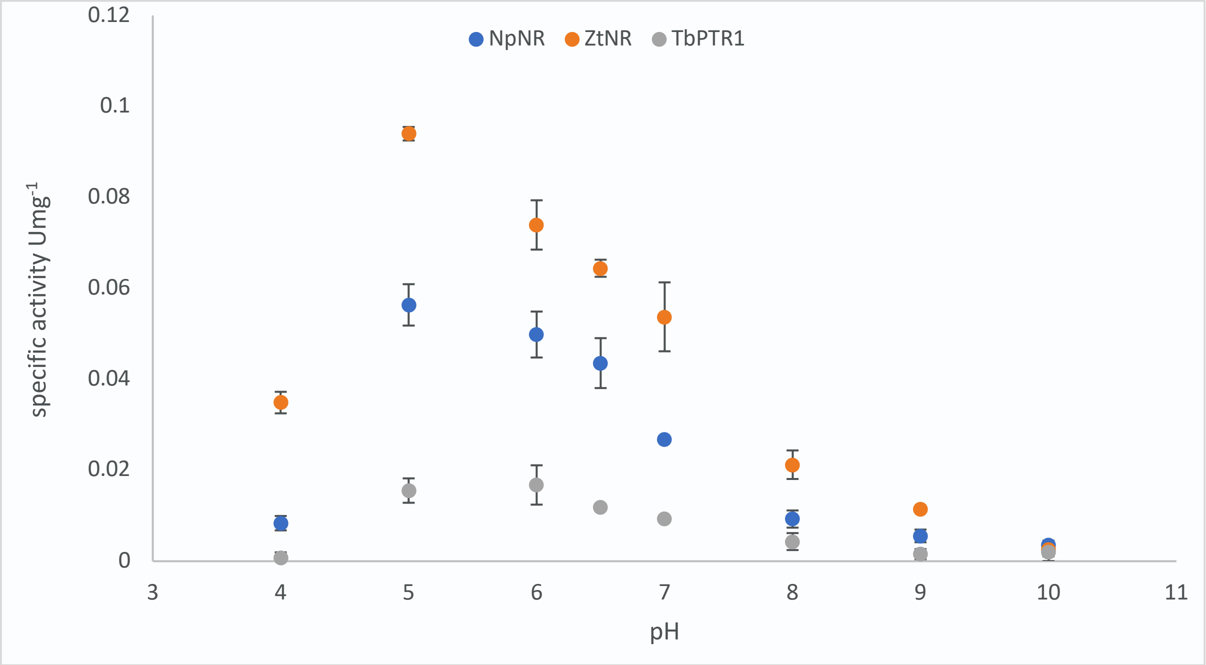

pH	Buffer	pH	Buffer
pH4-6	Citrate-Phosphate Buffer	pH 8-9	Tris-HCl
pH 6.5-7.5	Phosphate buffer	pH 10-11	Glycine-NaOH

Figure S6. pH profiles for the NADPH-dependent reduction of 1-methyl-3,4-dihydroisoquinoline imine for selected representatives of imine reducing enzymes.

Supplemental Representative chromatograms

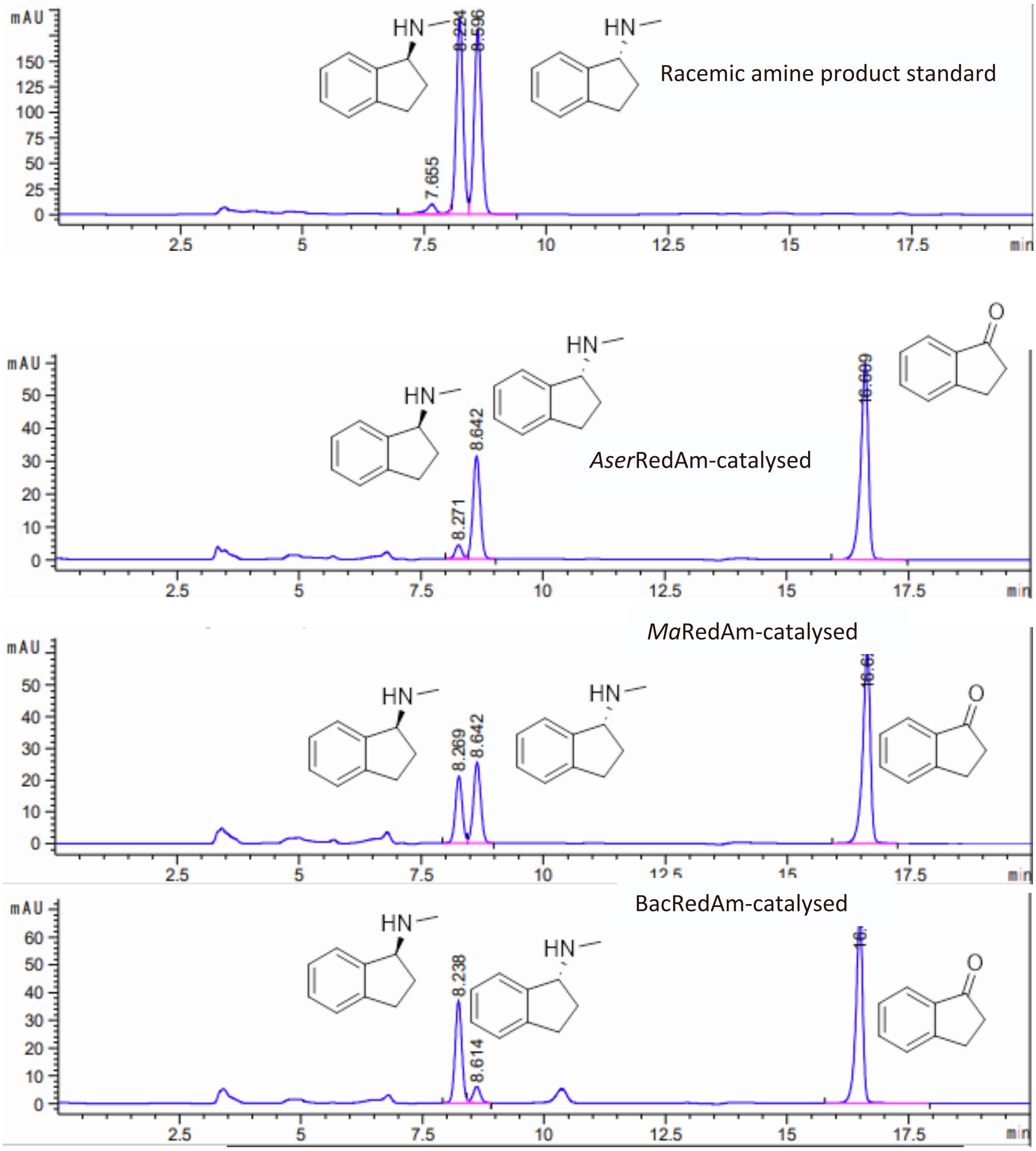

Figure S7. An example of products obtained from IRED-catalysed amination of aromatic ketones: Chiral HPLC analysis of IRED-catalysed reductive amination of 1-indanone **27** with methylamine **c** to afford enantioenriched alpha secondary amine product **27c**.

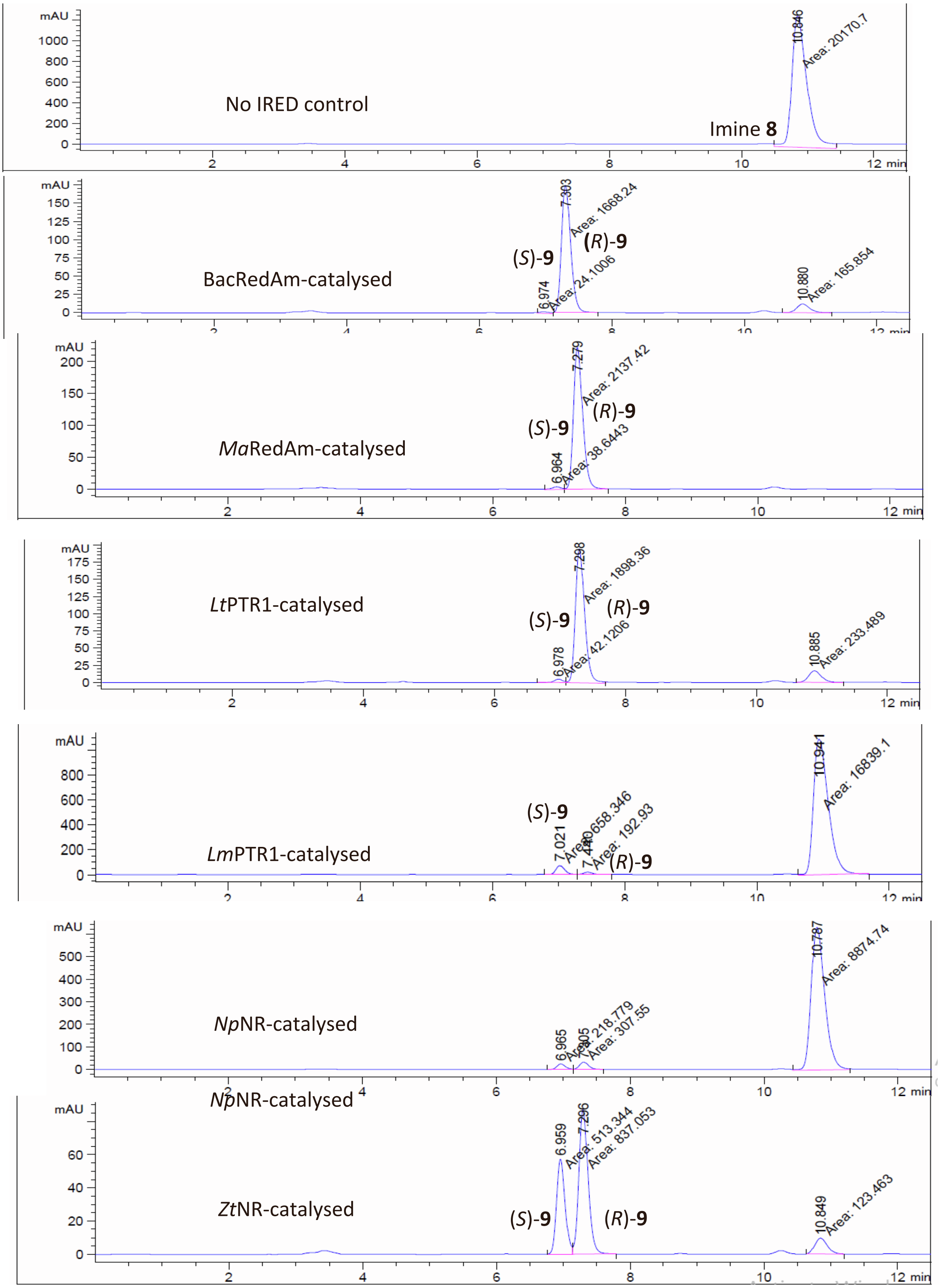

Figure S8. Chiral HPLC analysis of biotransformation reaction for the reduction of 1-methyl-3,4-dihydroisoquinoline imine **8** to yield.

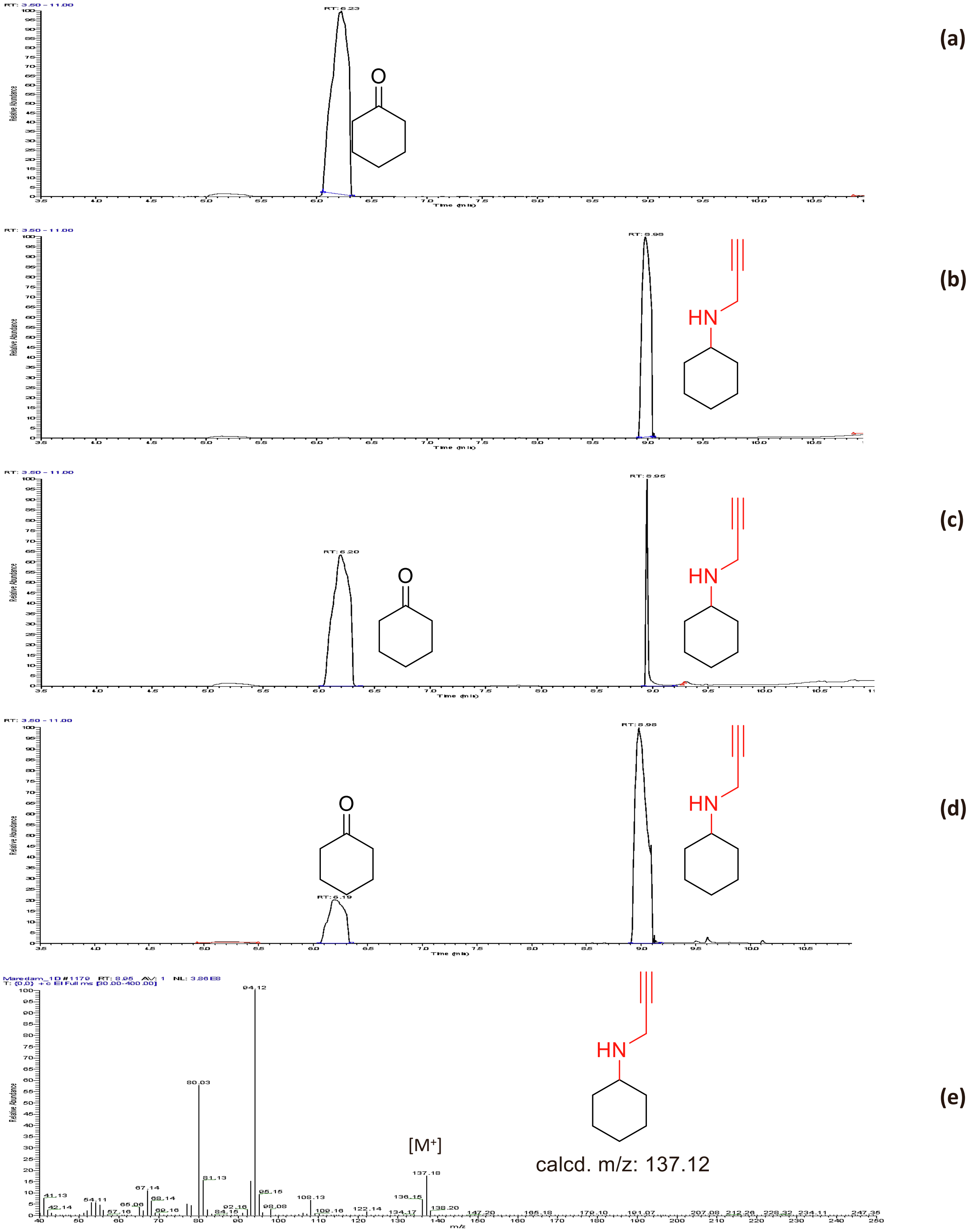

Figure S9. IRED-catalysed Reductive amination of cyclohexanone and propargylamine. (a) Control reaction containing all reaction components but lacking IRED. (b) Biotransformation catalysed by *Ma*RedAm. (c). Biotransformation catalysed by *Zt*NR (d). Biotransformation catalysed by *Lt*PTR1. (e) EI spectrum of the product obtained from MaRedAm-catalysed biotransformation. GCMS (EI) m/z = 137.18.

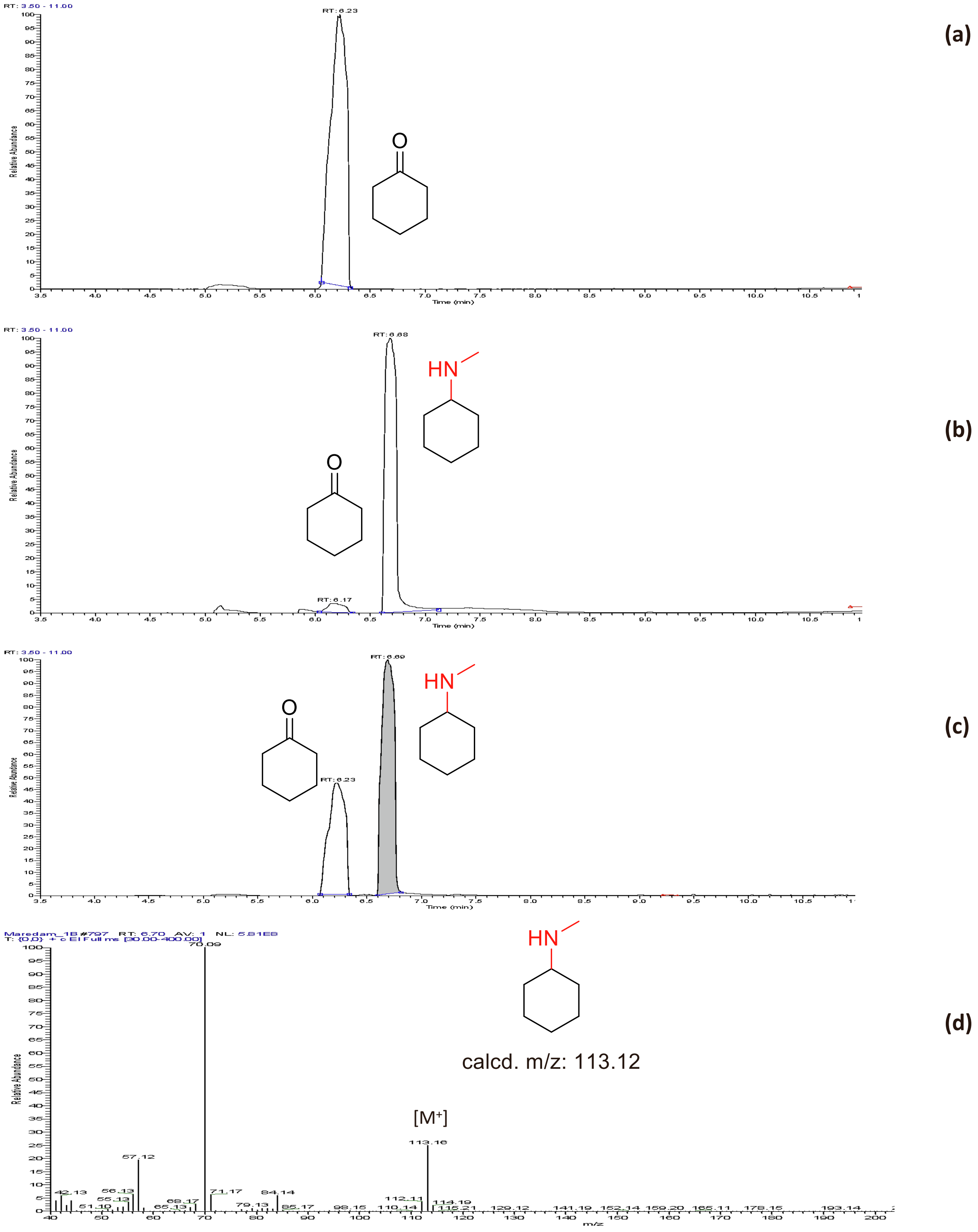

Figure S10. IRED-catalysed reductive amination of cyclohexanone and methylamine. (a) Control reaction containing all reaction components but lacking IRED. (b) Biotransformation catalysed by *Ma*RedAm. (c). Biotransformation catalysed by *Lt*PTR1, (d) EI spectrum of product obtained from MaRedAm-catalysed biotransformation. GCMS (EI) m/z found= 113.16.

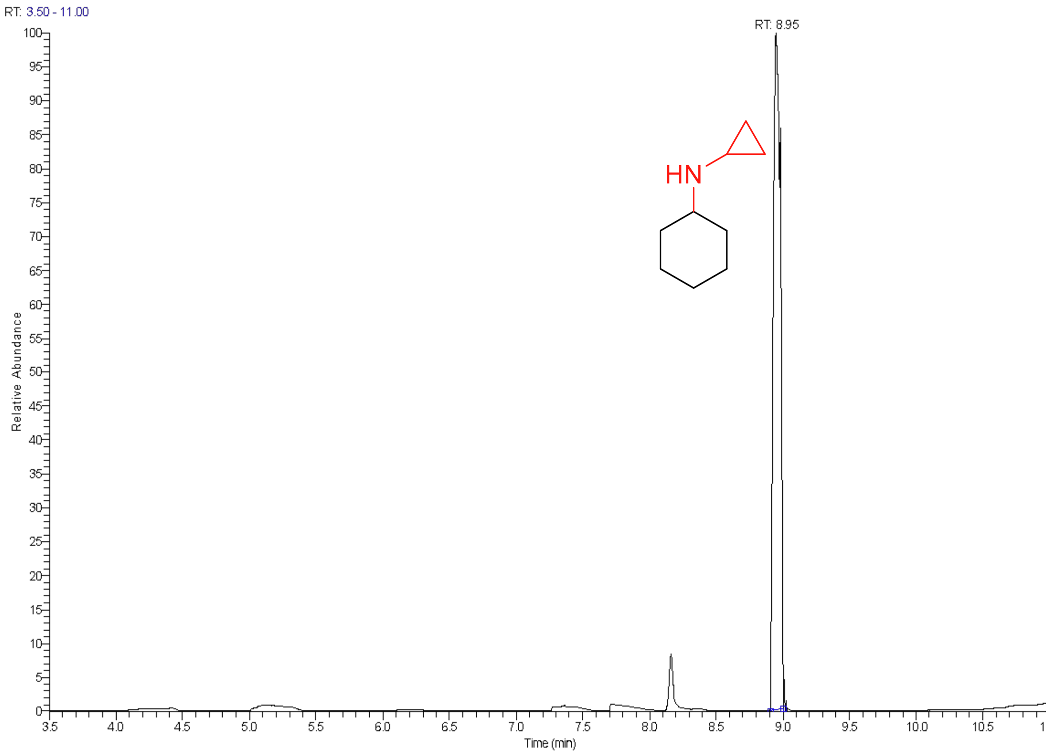

(a)

(b)

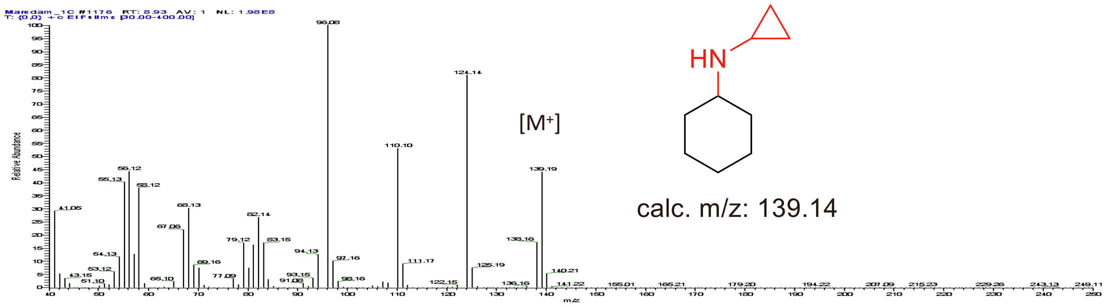

Figure S11. IRED-catalysed reductive amination of cyclohexanone and cyclopropylamine. (a) Biotransformation catalysed by *Ma*RedAm. (b) EI spectrum of the product obtained from MaRedAm-catalysed biotransformation. GCMS (EI) m/z found = 139.19.

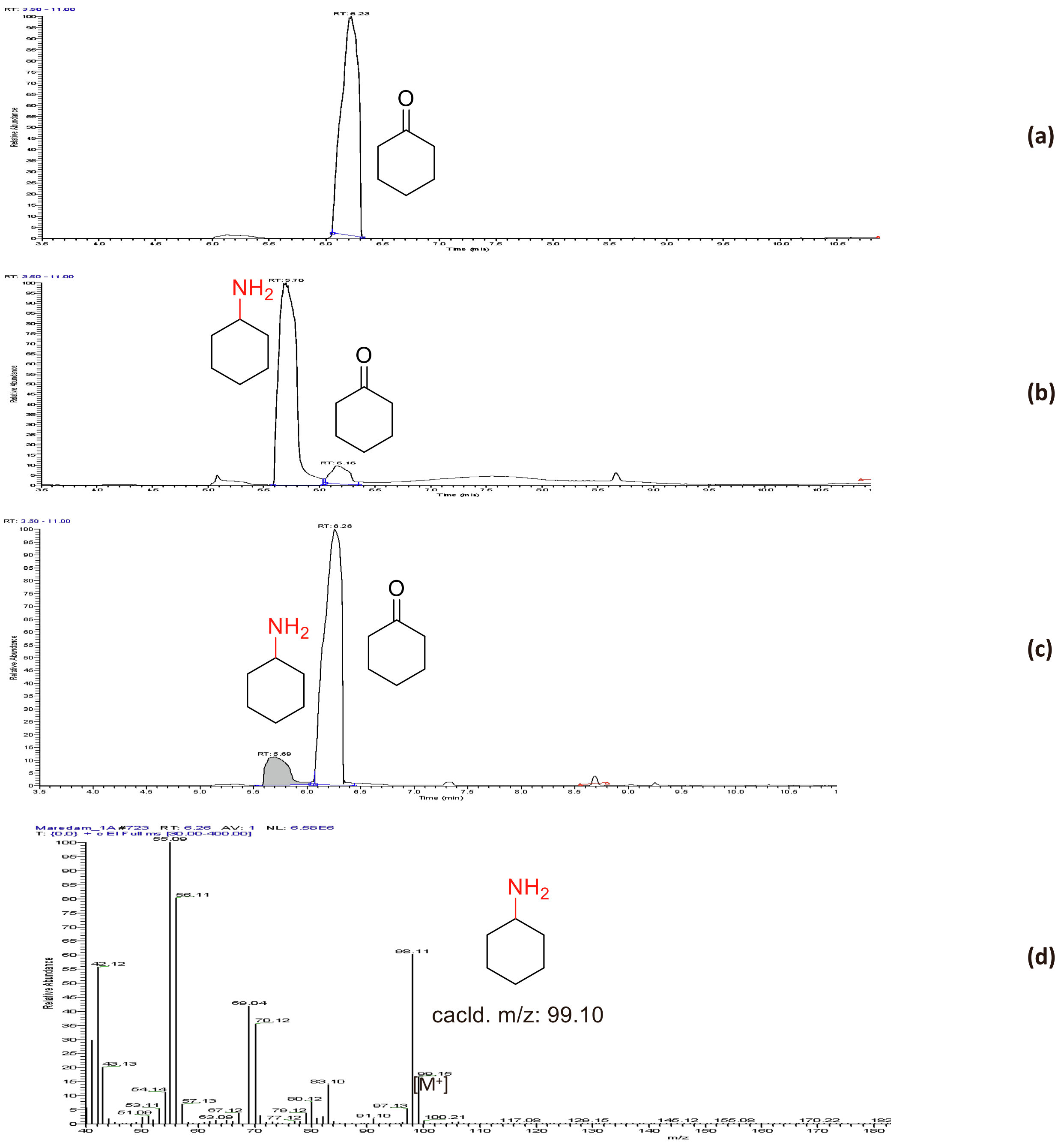

Figure S12. IRED-catalysed reductive amination of cyclohexanone and methylamine. (a) Control reaction containing all reaction components but lacking IRED. (b) Biotransformation catalysed by *Ma*RedAm. (c). Biotransformation catalysed by BacRedAm, (d) EI spectrum of product obtained from MaRedAm-catalysed biotransformation. GCMS (EI) m/z found= 99.15

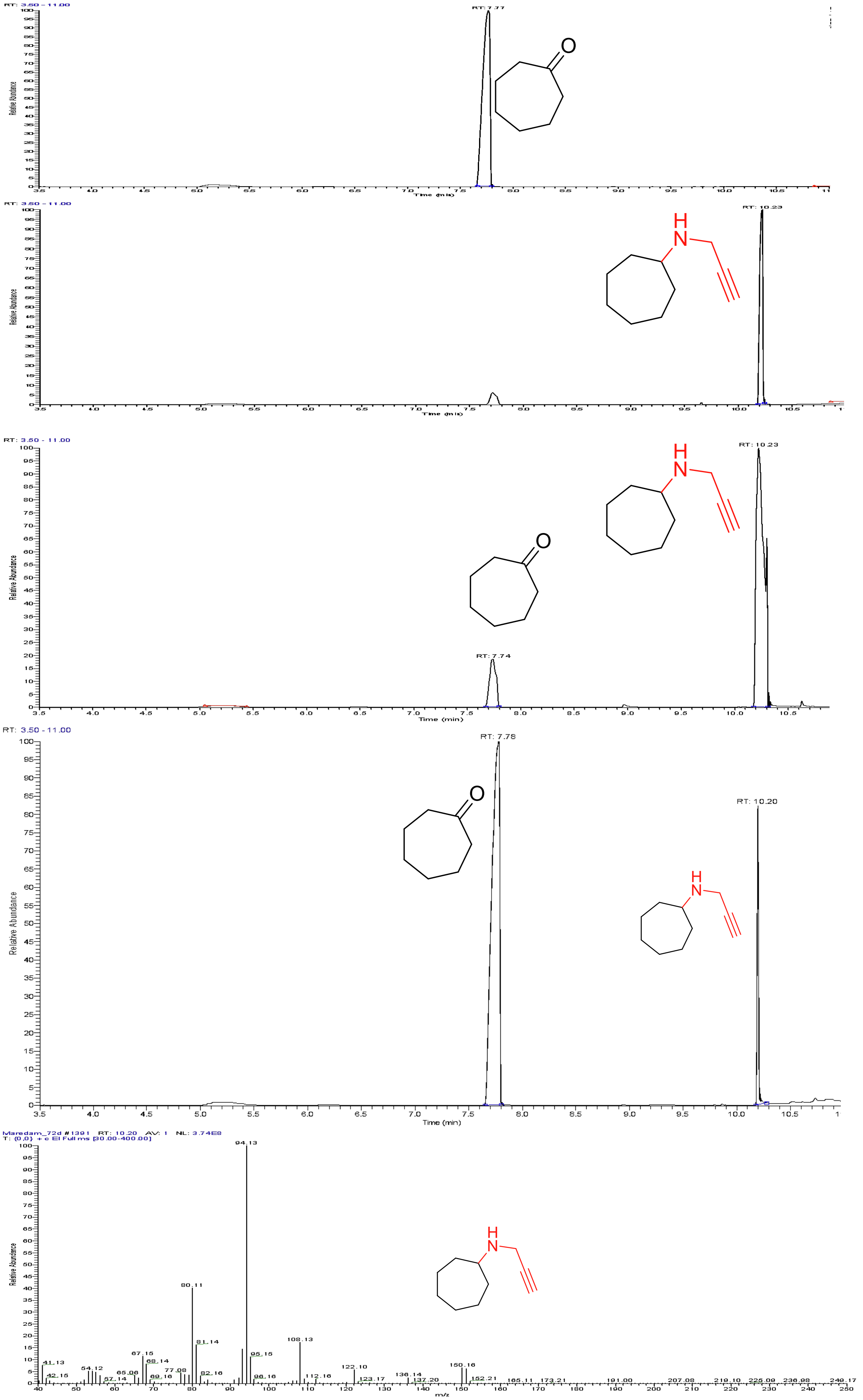

(a)

(b)

(c)

(d)

(e)

Figure S13. IRED-catalysed reductive amination of cycloheptanone and propargylamine. (a) Control reaction containing all reaction components but lacking IRED. (b) Biotransformation catalysed by

*Ma*RedAm. (c). Biotransformation catalysed by *Lt*PTR1 (d). Biotransformation catalysed by *Zt*NR. (e) EI spectrum of product obtained from MaRedAm-catalysed biotransformation.

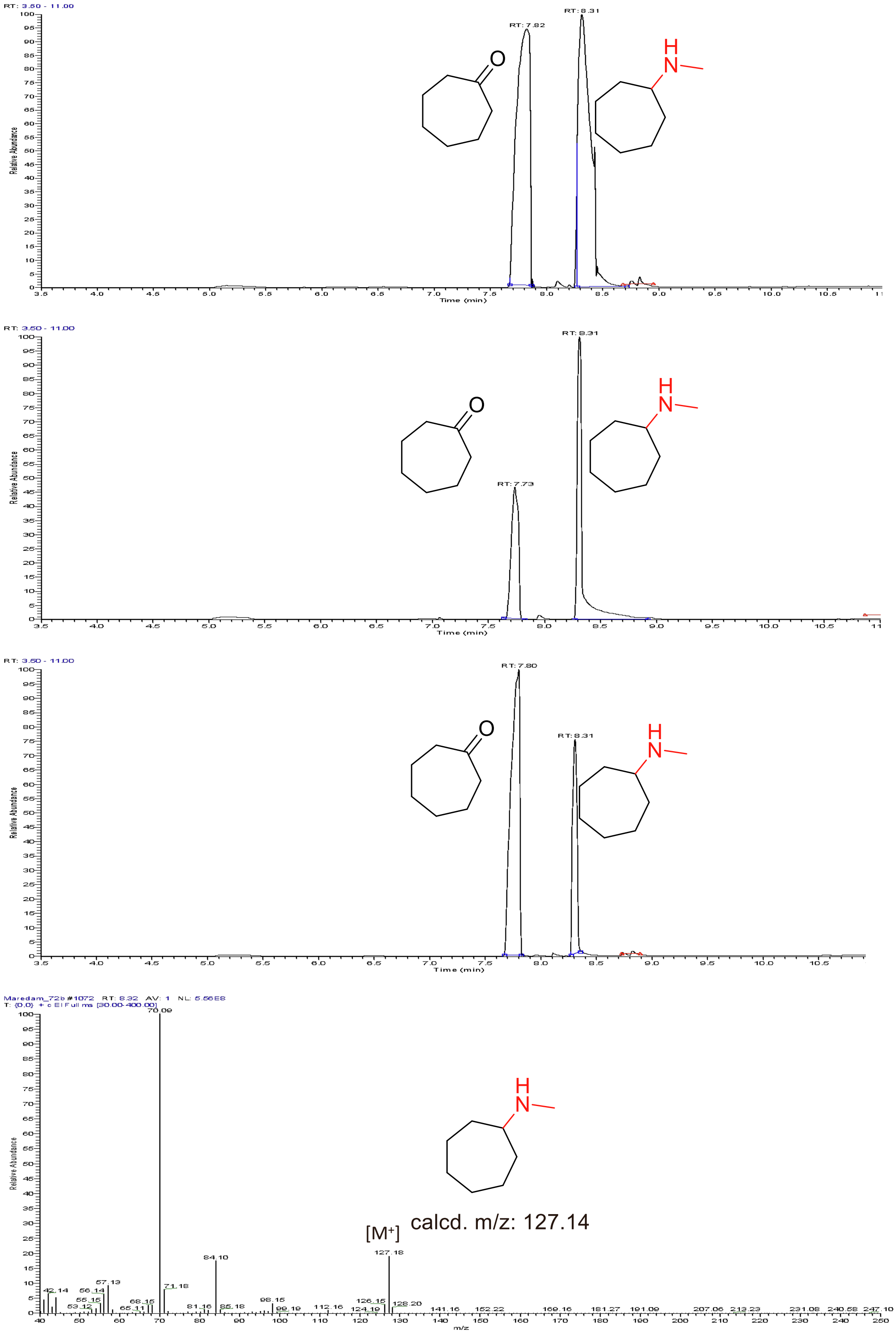

(a)

(b)

(c)

(d)

Figure S14. IRED-catalysed reductive amination of cycloheptanone and methylamine. (a) Biotransformation catalysed by *Ba*RedAm. (a) Biotransformation catalysed by *Ma*RedAm. (c).

Biotransformation catalysed by *Lt*PTR1, (d) EI spectrum of product obtained from MaRedAm-catalysed biotransformation. GCMS (EI) m/z found= 127.18

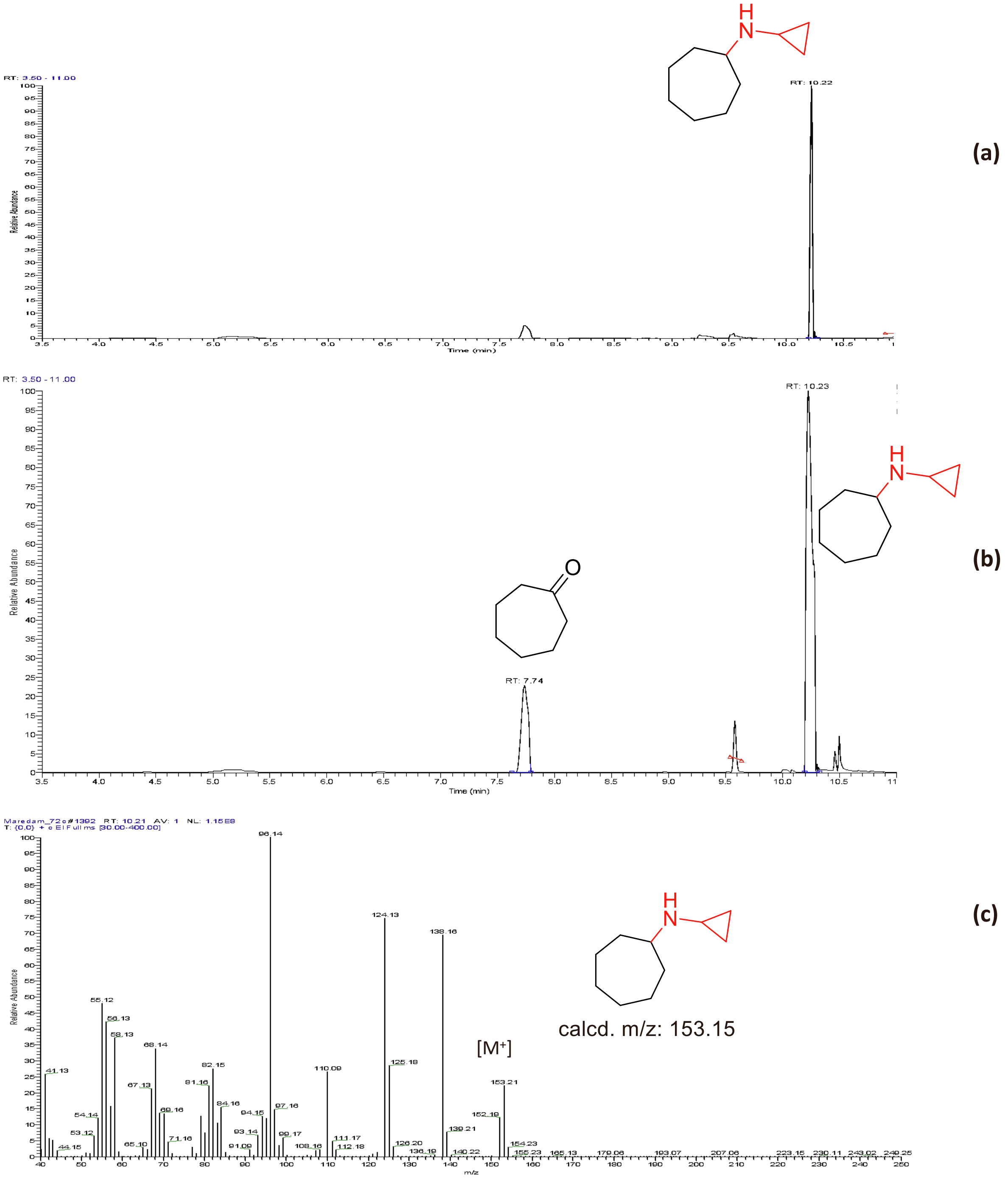

Figure S15. IRED-catalysed reductive amination of cycloheptanone and cyclopropylamine. (a) Biotransformation catalysed by *Ma*RedAm. (b). Biotransformation catalysed by *Lt*PTR1, (d) EI spectrum of product obtained from MaRedAm-catalysed biotransformation. GCMS (EI) m/z found= 153.2.

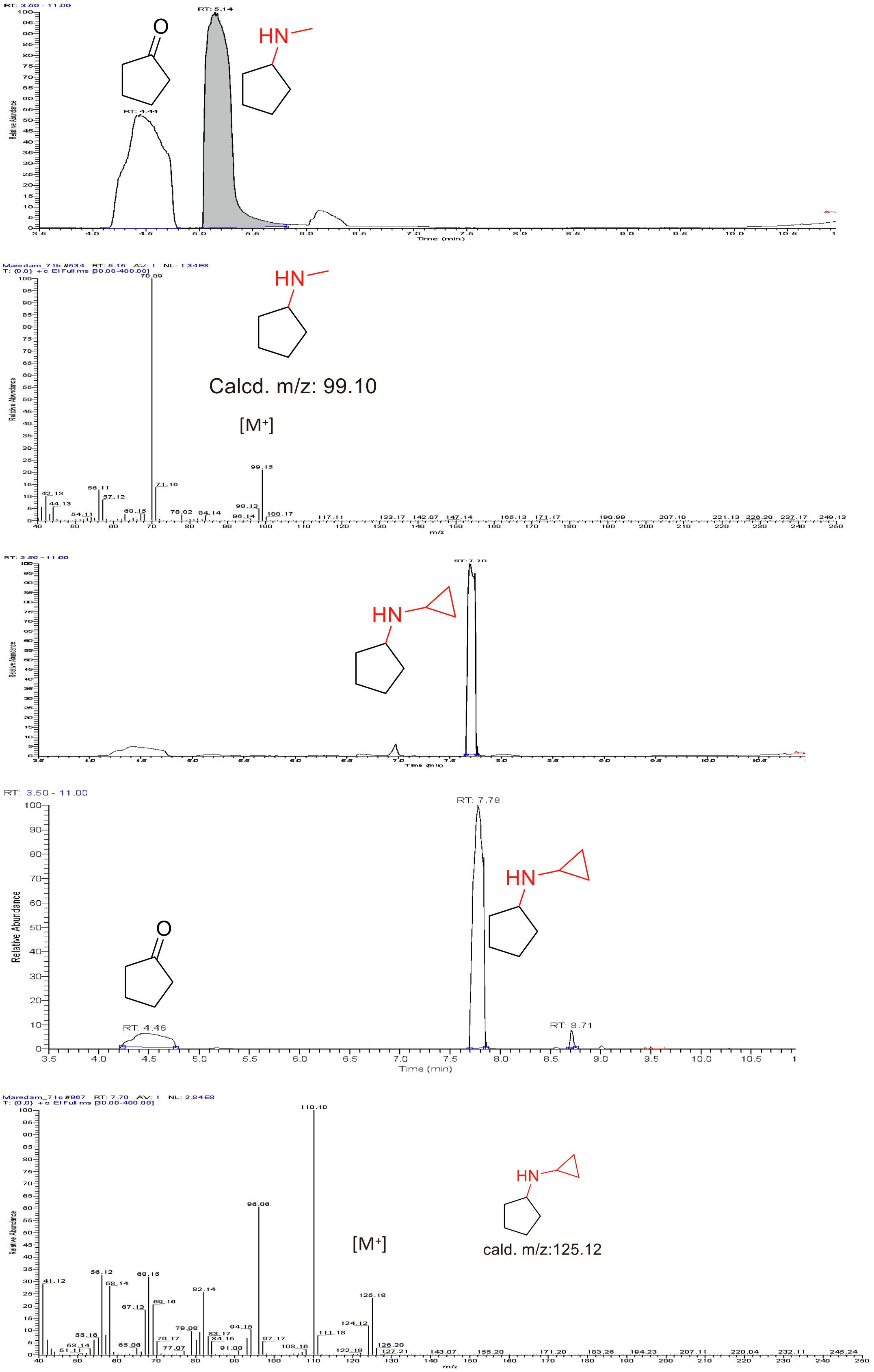

(a)

(b)

(c)

(d)

(e)

Figure S16. IRED-catalysed reductive amination of cyclopentanone. (a, b) Bioamination of cyclopentanone with methylamine catalysed by *Ma*RedAm and the associated EI spectrum of the biotransformation product, GCMS (EI) m/z found= 99.15. (c-e), Bioamination of cyclopentanone with propargylamine catalysed *Ma*RedAm (row c) and LtPTR1 (row d) and the associated EI spectrum of

the biotransformation product obtained from *Ma*RedAm-catalysed reaction (row e). GCMS (EI) m/z found= 125.18.

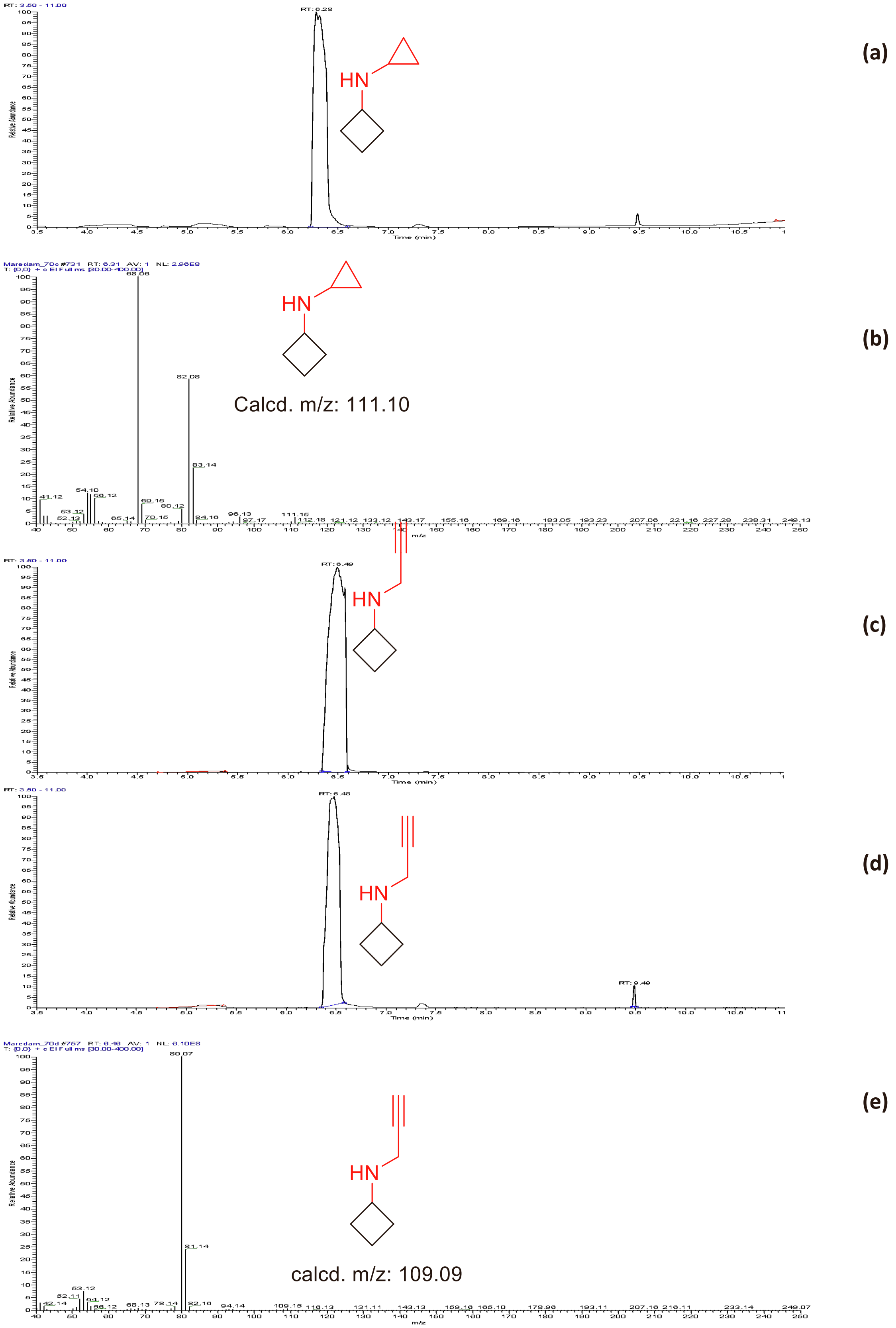

Figure S17. IRED-catalysed reductive amination of cyclobutanone. (a, b) Bioamination of cyclobutanone with cyclopropylamine catalysed by *Ma*RedAm and (b) the associated EI spectrum of the biotransformation product, GCMS (EI) m/z found= 111.15. (c-e), Bioamination of cyclobutanone

with propargylamine catalysed *Ma*RedAm (row c) and LtPTR1 (row d) and the associated EI spectrum of the biotransformation product obtained from *Ma*RedAm-catalysed reaction (row e). GCMS (EI) m/z found= 109.15

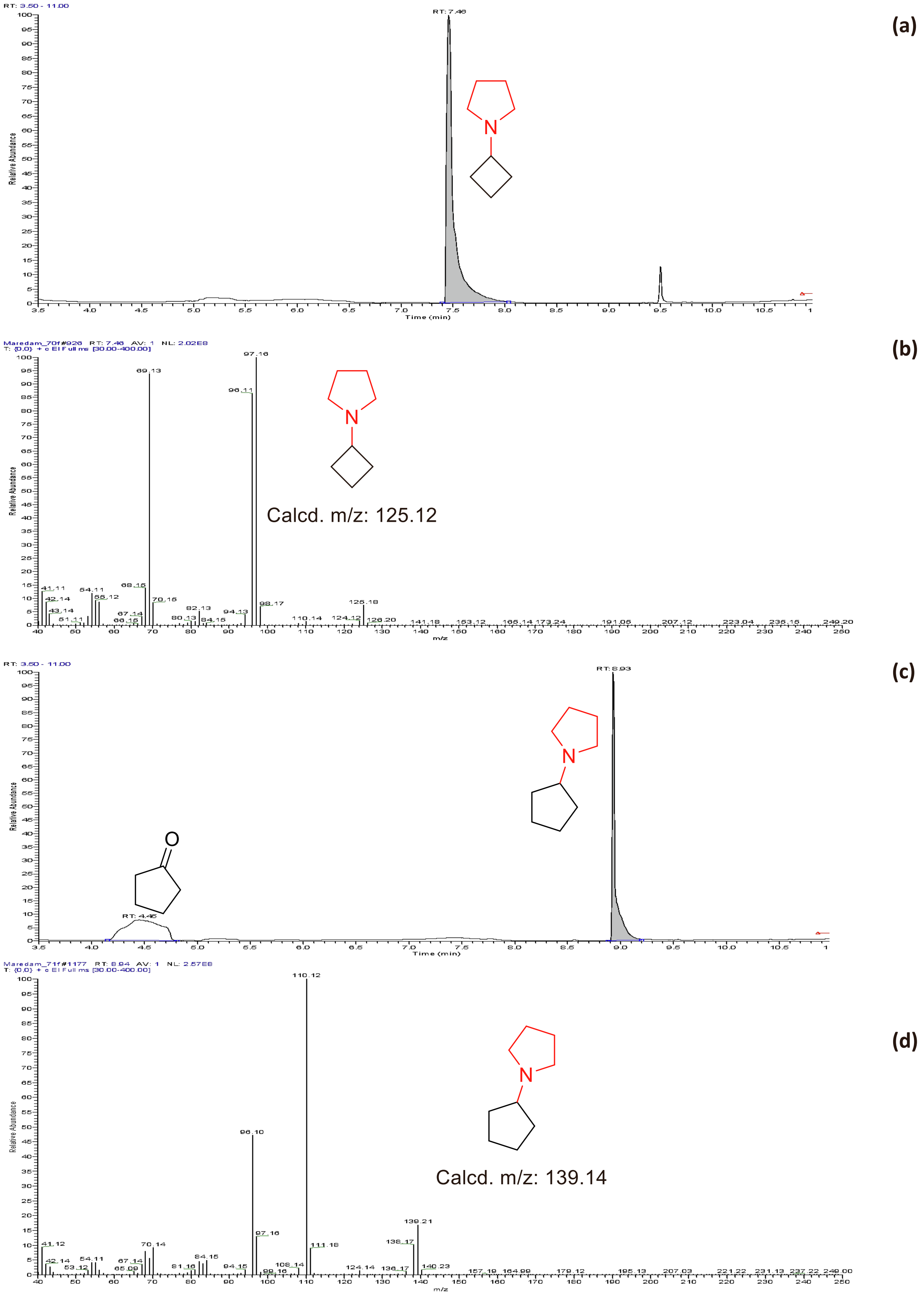

Figure S18. IRED-catalysed reductive amination of ketones with pyrrolidine. (a) Bioamination of cyclobutanone with pyrrolidine catalysed by *Ma*RedAm and (b) the associated EI spectrum of the

biotransformation product, GCMS (EI) m/z found= 125.18. (c-e), Bioamination of cyclopentanone with propargylamine catalysed *Ma*RedAm (row c) and the associated EI spectrum of the biotransformation product obtained from MaRedam-catalysed reaction. GCMS (EI) m/z found= 139.21.

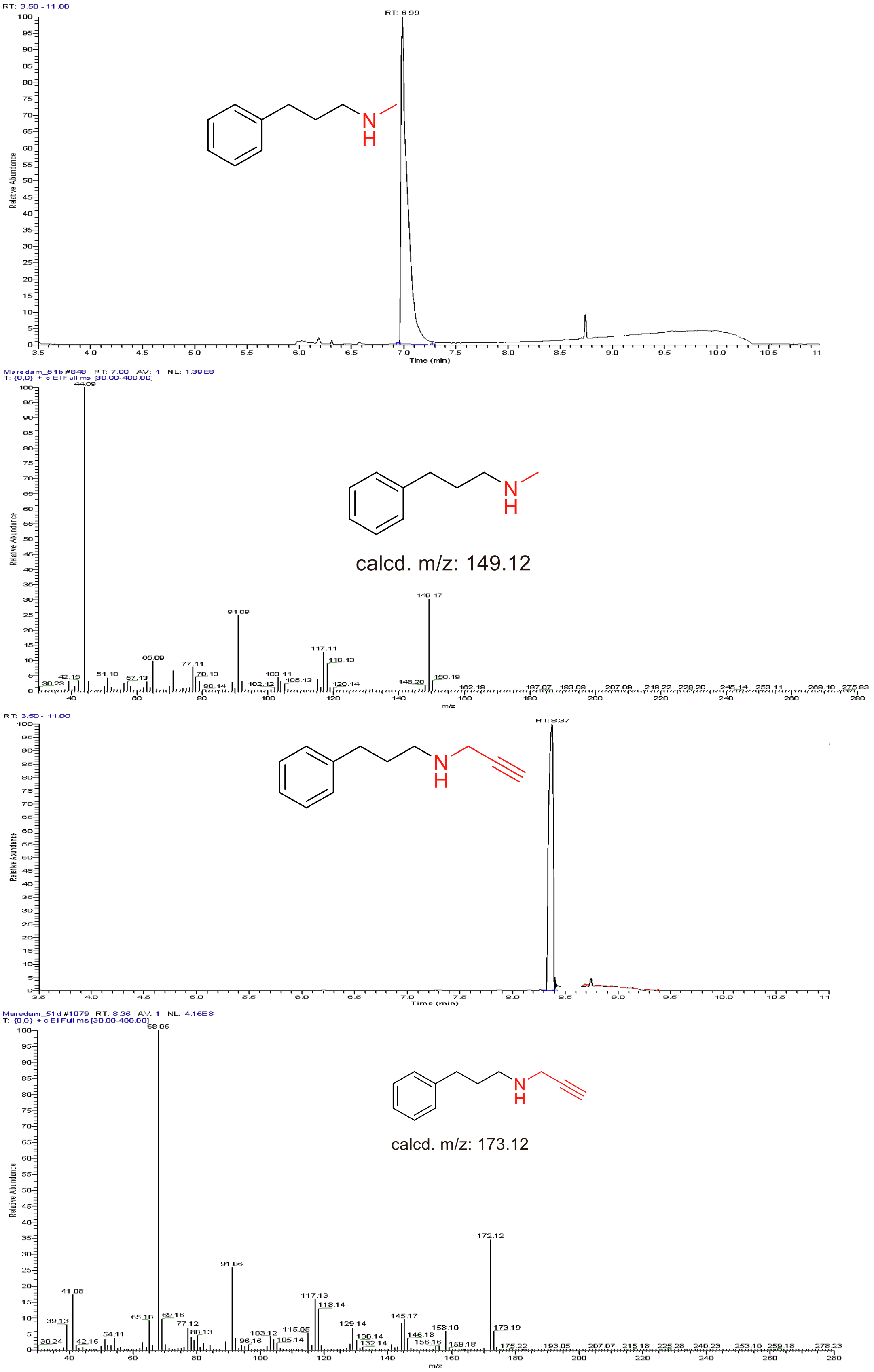

(a)

(b)

(c)

(d)

Figure S19. IRED-catalysed reductive amination of hydrocinnamaldehyde. (a) Bioamination of

hydrocinnamaldehyde with methylamine catalysed by *Ma*RedAm and (b) the associated EI spectrum of the biotransformation product, GCMS (EI) m/z found= 149.17. (c) Bioamination of hydrocinnamaldehyde with propargylamine catalysed by *Ma*RedAm and (b) the associated EI spectrum of the biotransformation product, GCMS (EI) m/z found= 173.19.

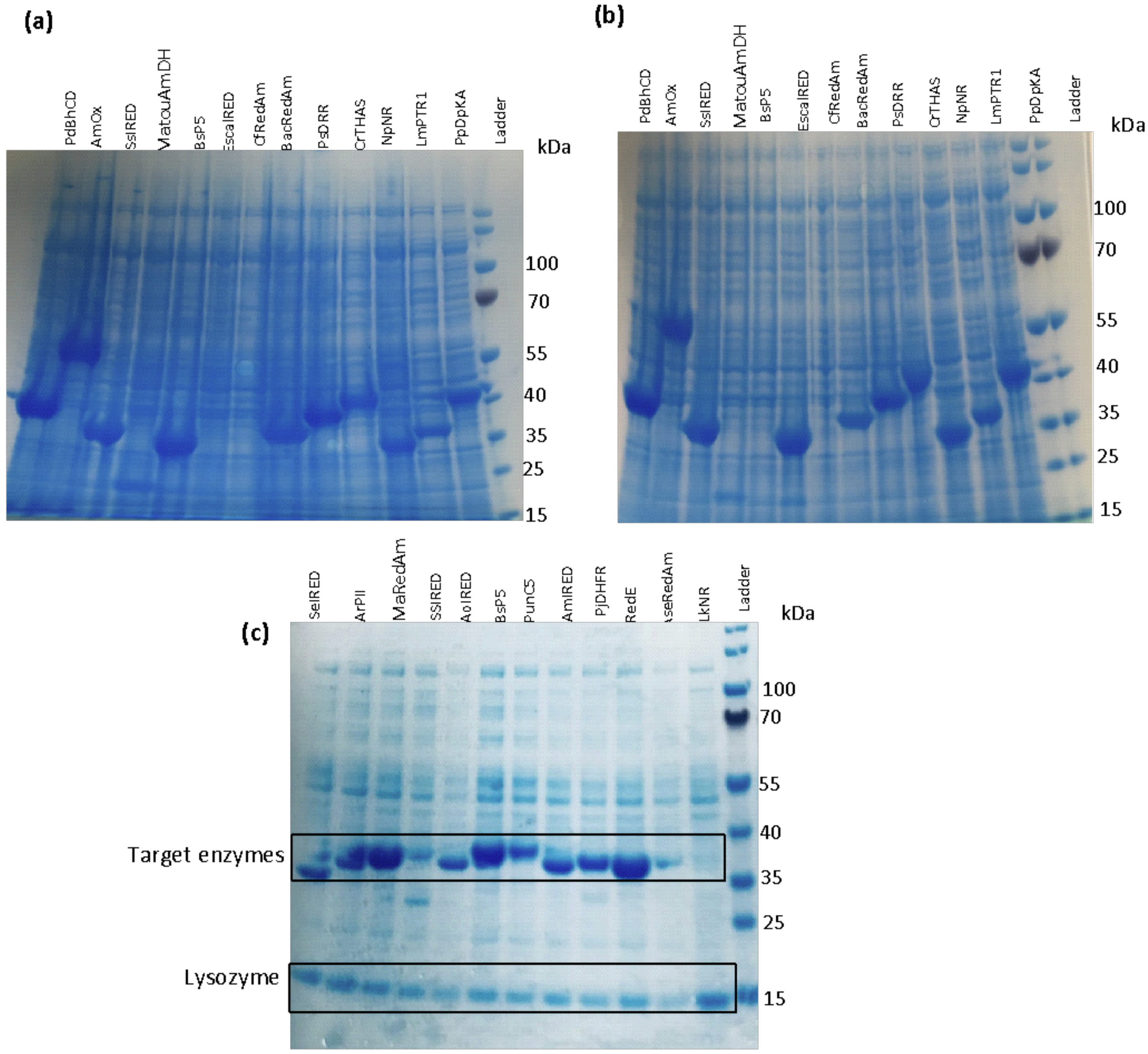

Figure S20. SDS page gels to detect soluble expression by analysis of clarified lysates obtained from recombinant *E. coli* expression. Expression was trialled with **(a)** Lysogeny broth (LB) autoinduction media (initial cultivation for 5 h at 37 °C then continued for 40 h at 24 °C) *versus* **(b,c)** LB media with IPTG induction (initial cultivation at 37 °C until OD_{600} of between 0.6 and 0.8, then induction with IPTG (0.4 mM), cultivation continued at 22°C). For gels (a) and (b), cell disruption was performed using ultrasonication. For gel (c), lysis was performed with lysozyme-based lysis buffer (100 mM Tris-HCl, 100 mM NaCl, pH 7.5, 1 mg ml^{-1} lysozyme, 0.3 mg ml^{-1} polymyxin B), incubated at 20 °C for 1h 30 min. Both IPTG induction and autoinduction conditions were suitable for the expression of most of the investigated enzymes. Similarly, cell disruption with either ultrasonication or lysozyme-based cell lysis was both effective. The enzymatic lysis was employed for small-volume cultures, while ultrasonication was used for larger-volume cultures.

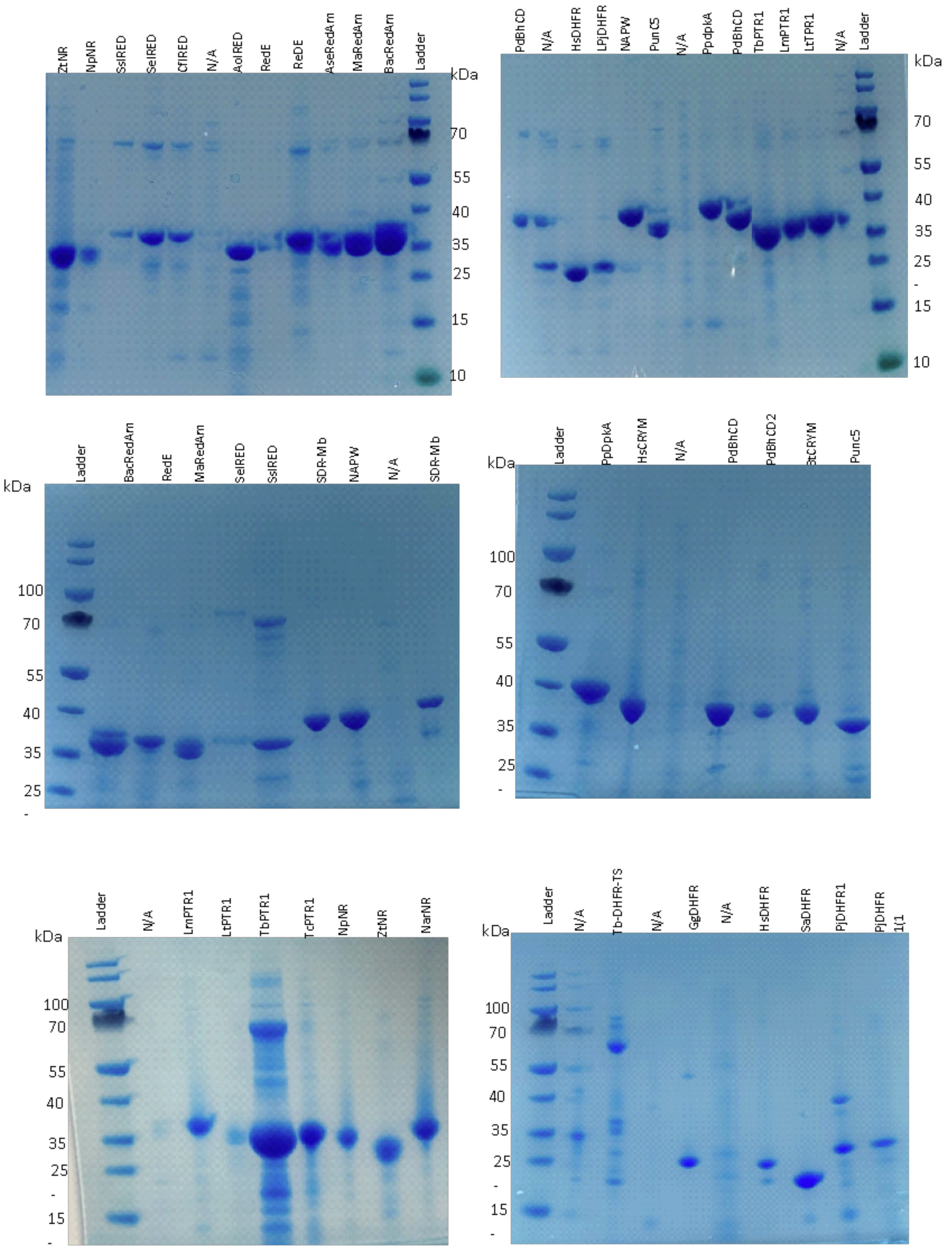

Figure S21. Protein gel (SDS page) analysis of semi-purified IREDs. For quick (semi)-purification, cells were resuspended in buffer A (50 mM Tris-HCl buffer, 300 mM NaCl, 25 mM imidazole pH 7.5) and were disrupted by ultrasonication at 4°C employing 5 × 20 s bursts with 40 s intervals at 4 °C. Following centrifugation of the lysed cells, the clarified lysate was purified by Ni-affinity chromatography using gravity columns. Clarified lysate was loaded onto the column, followed by a single-step wash with buffer A, and eluted with buffer B (50 mM Tris-HCl buffer, 300 mM NaCl, 300 mM imidazole, pH 7.5). The elute was desalted using 10 kDa cut-off Centricon filters (15 ml) and the buffer was exchanged to Tris-HCL buffer (50 mM Tris, 100 mM NaCl, pH 7.5) or HEPES (30 mM, 100 ml NaCl).

Table S1. A panel of highly divergent/non-homologous (biosynthetic) imine-reducing enzymes investigated in this study

Entry		Organismal source	*Physiological function (Pathway)*	*Genbank ID*
Classical IREDs involved in biosynthesis and homologues				
1	RedE	*Metagenomic*	tryptophan dimer (TD) biosynthesis	>AKG47111.1
2	ArpDHII	*Streptomyces argillaceus*	Argimycins P dehydrogenase	SCO70303.1
3	*Bac*RedAm	Metagenomic (bacterial)	Not known	PZN88780.1
4	*Mared*Am	*Mortierella antarctica*	Not known	KAF9989289.1
5	*Aser*RedAm	*Aspergillus sergii*	Not known	KAE8332418.1
6	*Pt*IRED	*Pyxidicoccus trucidator*	Not known	WP_164011897.1
7	*Ss*IRED	*Streptomyces sp. WAC 05977*	Not known	RSN12190.1
8	*Se*IRED	*Saccharopolyspora erythraea*	Not known	WP_211898848.1
9	*ArIRED*	*Amycolatopsis roodepoortensis*	Not known	WP_257488618.1
Pteridine reductases (PTR1s)				
10	*Lm*PTR1	*Leishmania major*	Protozoan Pteridine reductase (pterins salvage)	sp|Q01782.2
11	*Lt*PTR1	*Leishmania tarentolae*	Protozoan Pteridine reductase (pterins salvage)	sp|P42556.1
12	*Tc*PTR1	*Trypanosoma cruzi*	Protozoan Pteridine reductase (pterins salvage)	AAC38850.1
13	*Tb*PTR1	*Trypanosoma brucei equiperdum]*	Protozoan Pteridine reductase (pterins salvage)	RHW70915.1
14	*Am*PTR1	*Alteromonas mediterranea*	PTR1-like protein	WP_071968609.1
15	*At*PruA	*Agrobacterium tumefaciens*	Bacterial pteridine reductase	WP_006312872.1
16	*Pa*PruA	*Pseudomonas aeruginosa*	Bacterial pteridine reductase	WP_003091895.1
Dihydrofolate reductases (DHFRs)				
17	*Pj*DHFR/PcDHFR	*Pneumocystis jirovecii*	Dihydrofolate reductase (folate metabolism)	1VJ3_A
18	*Gg*DHFR	*Gallus gallus*	Dihydrofolate reductase (folate metabolism)	NP_001006584.2
19	*h*DHFR	*Homo sapien*	Dihydrofolate reductase (folate metabolism)	NP_000782.1
20	*Tb*DHFR-TS	*Trypanosoma brucei*	Bifunctional dihydrofolate reductase-thymidylate synthase	Q27783.1
22	*Tb*DHFR	*Trypanosoma brucei*	Dihydrofolate reductase	3QFX_A
23	*PvDHfR*	*Plasmodium vivax*	Dihydrofolate reductase	WAN11905.1

24	*PfDHFR*	*Plasmodium falciparum*	Dihydrofolate reductase	PDB: 1J3I_A
Short-chain dehydrogenases/reductases *(SDRs) involved in plant alkaloid biosynthesis*				
25	*Np*NR	*Narcissus pseudonarcissus*	noroxomaritidine/norcraugsodine reductase (NR)	AUG71944.1
26	*NpaNR*	*Narcissus papyraceus*	noroxomaritidine/norcraugsodine reductase	AXU39908.1
27	*Zt*NR	*Zephyranthes treatiae*	NR-like plant protein	6Y4D_A
28	LrNR	*Lycoris radiata*	NR-like plant protein	QFQ50502.1
29	LsNR	*Larkinella sp. BK230*	NR-like bacterial protein	WP_208327519.1
30	*Cr*THAS	*Catharanthus roseus*	tetrahydroalstonine synthase	AKF02528.1
31	*EcSaRed*	*Eschscholzia californica*	Sanguinarine reductase (benzophenanthridine detoxification)	>sp|D5JWB3.1
32	*Ps*DRR	*Papaver Sp*	1,2-dehydroreticuline reductase (morphine biosynthesiss)	ACM44068.1
33	*Cp*EaSG	*Claviceps gigantea*	agroclavine synthase (ergot alkaloid biosynthesis)	ATW01297.1
SDRs in bacterial alkaloids biosynthesis				
34	NAPW	*Streptomyces sp.*	naphthyridinomycin biosynthetic	WP_121719702.1
35	*Mb*SDR	*Myxococcales bacterium*	NAPW-like protein	OJY30843.1
36	*Pb*SDR	*Paenibacillus sp.*	NAPW-like protein	WP_076164526.1
(A)cyclic imino-acid reductases				
37	PunC5	*Paenibacillus sp.*	Imino acid reductase	WP_090636782.1
38	*BsP5*	*Bacillus sp.*	Imine acid reductase	pdb|6P2I|A
39	*Pd*BhcD	*Paracoccus denitrificans*	Imminosuccinate reductase (β-hydroxyaspartate cycle)	pdb|6RQA|A
40	*Pp*DpKA	Pseudomonas putida	Amino acid biosynthesis	Q5FB93.1
41	*Hs*CRYM	*Human sapiens*	ketimine reductase mu-crystallin	NP_001363185.1
42	*Bt*DHFR	*Bos taurus*	ketimine reductase mu-crystallin	Leucojum aestivum
43	*Cp*IM1	*Candida parapsilosis*	Ketimine reductase-like protein	XP_036665050.1
44	PchG	*Pseudomonas aeruginosa*	pyochelin biosynthesis thiazoline reductase	: WP_250024055.1

Table S2. Biotransformation for the imine reduction of 1-methyl-3,4-dihydroisoquinoline **8** using stoichiometric amounts of NAD(P)H.

Enzyme	Cofactor	Conversion (%)	e.e. (%)	Absolute conf.
*Am*IRED	NADPH	98	71	(S)
	NADH	>99	79	(S)
*Bac*Redam	NADPH	>98	94	(*R*)
	NADH	93	89	(*R*)
MaRedAm	NADPH	98	86	(*R*)
	NADH	>99	90	(*R*)
*Lt*PTR1	NADPH	>99	92	(*R*)
	NADH	>99	92	(*R*)
*Lm*PTR1	NADPH	>99	49	(S)
	NADH	59	70	(S)
*Tb*PTR1	NADPH	79	1	(S)
	NADH	45	11	(S)
*Np*NR	NADPH	>99	15	(*R*)
	NADH	91	19	(*R*)
*Np*NR	NADPH	>99	19	(*R*)
	NADH	>99	20	(*R*)

Reaction condition: 5 mM imine, 10 mM NAD(P)H, 0.25-1 mg ml^{-1} IRED in 0.5 ml phosphate buffer (100 mM with 100 mM NaCl, pH 7.0). The reaction was incubated at 25 °C for 24 h.

Table S3. HPLC methods and retention times

Method A: *n*-hexane/isopropanol/diethylamine (90/10/0.1).
Method B: *n*-hexane/isopropanol/diethylamine (80/20/0.1).
Method C: *n*-hexane/isopropanol/diethylamine (98/02/0.1).
Method D: *n*-hexane/isopropanol/diethylamine (97/03/0.1).
Flow rate: 1 ml min^{-1}, monitored at wavelength of 265 nm.
Columns: CHIRALPAK®IC 250 mm × 4.6 mm, 5 μm; and CHIRALPAK® IB N-5, 250 mm × 4.6 mm, 5 μm.

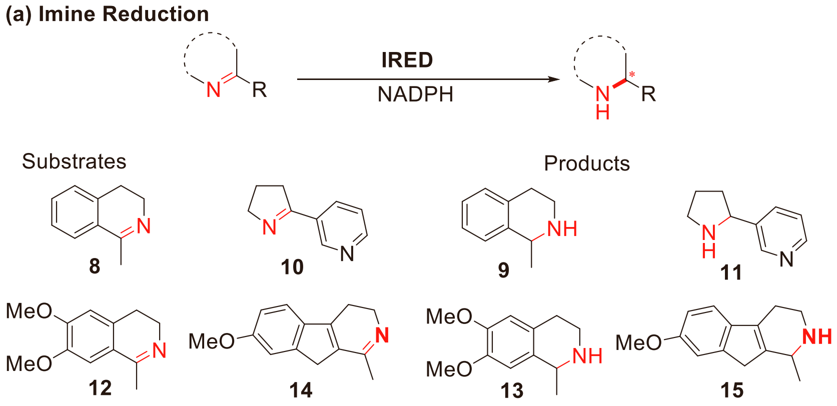

Imine Substrate	Amine product	Column	n-hexane/IPA/DEA	Retention time		
				Imine	Amine product	
					T1	T2
8	9	CHIRALPAK®IC	Method A	11.0	7.0 (*S*)	7.4 (*R*)

**10**	**11**	CHIRALPAK®IB N-5	Method A	12.7	16.2 (*R*)	19.4 (S)
**12**	**13**	CHIRALPAK®IB N-5	Method A	12.4	16.2 (*R*)	19.4 (*S*)
**14**	**15**	CHIRALPAK®IC	Method B	6.7	12.0 (S)	14.6 (*R*)

(b) Reductive Amination

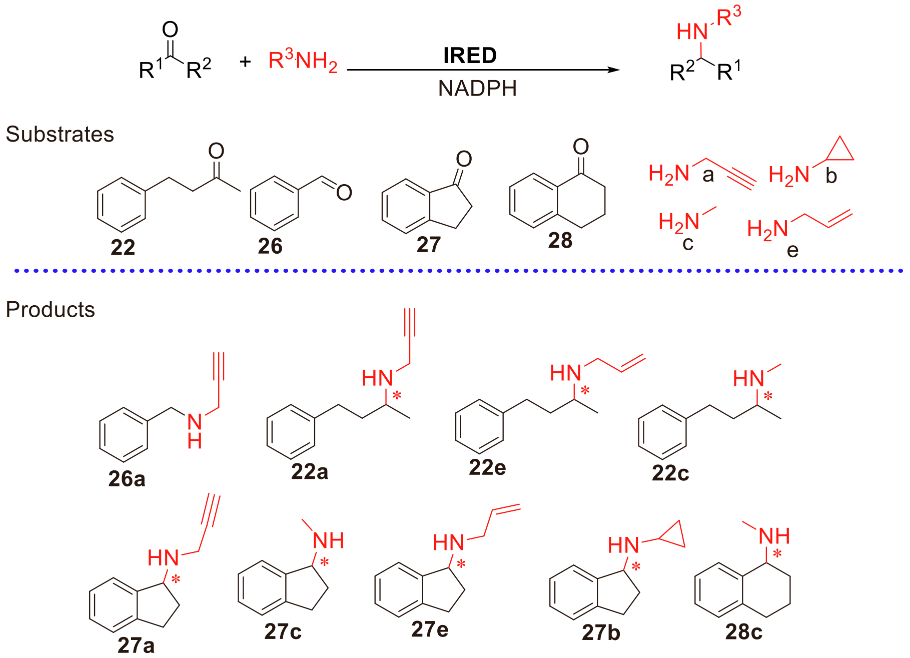

Substrates		Amine product	Column	n-hexane/IPA/DEA	Retention time		
					Ketone/aldehyde	Amine product	
Carbonyl	Amine					T1	T2
**22**	**c**	**22c**	CHIRALPAK®IB N-5	Method C	6.3	5.4 (*R*)	5.9 (*S*)
**22**	**b**	**22b**	CHIRALPAK®IB N-5	Method C	6.3	3.9 [a]	
**22**	**e**	**22e**	CHIRALPAK®IB N-5	Method C	6.3	4.2 (*R*)	4.4 (*S*)
**22**	**a**	**22a**	CHIRALPAK®IB N-5	Method C	6.3	5.2 (*R*)	5.4 (*S*)
**26**	**a**	**26a**	CHIRALPAK®IC	Method C	7.8	6.3[b]	
**27**	**c**	**27c**	CHIRALPAK®IC	Method C	21.4	8.2 (*S*)	8.6 (*R*)
**27**	**c**	**27c**	CHIRALPAK®IC	Method D	17	8.2 (*S*)	8.6 (*R*)
**27**	**b**	**27b**	CHIRALPAK®IC	Method C	21.4	4.2[a]	
**27**	**e**	**27e**	CHIRALPAK®IC	Method C	21.4	4.7 *S*)	5.0 (*R*)
**27**	**a**	**27a**	CHIRALPAK®IC	Method C	21.4	6.1 *S*)	6.7 (*R*)
**28**	**c**	**28c**	CHIRALPAK®IC	Method C	14.0	6.5 (*S*)	7.2 (*R*)

[a] HPLC method did not resolve enantiomers. [b] product is non-chiral.

Table S4. GC-MS analysis: retention times for ketones/aldehydes and reductive amination products

Column: CD-5MS Capillary Column 30 m x 0.25 mm x 0.25µm (P/N MOD-GC-CD5MSU-5, S/N CD17506. Method: inlet temperature = 250°C, detector temperature = 250°C, MS source= 230 °C, gas flow = 1.0 ml min^{-1}; oven temperature between 60 - 280°C, 20°C min^{-1} or 15 °C min^{-1}

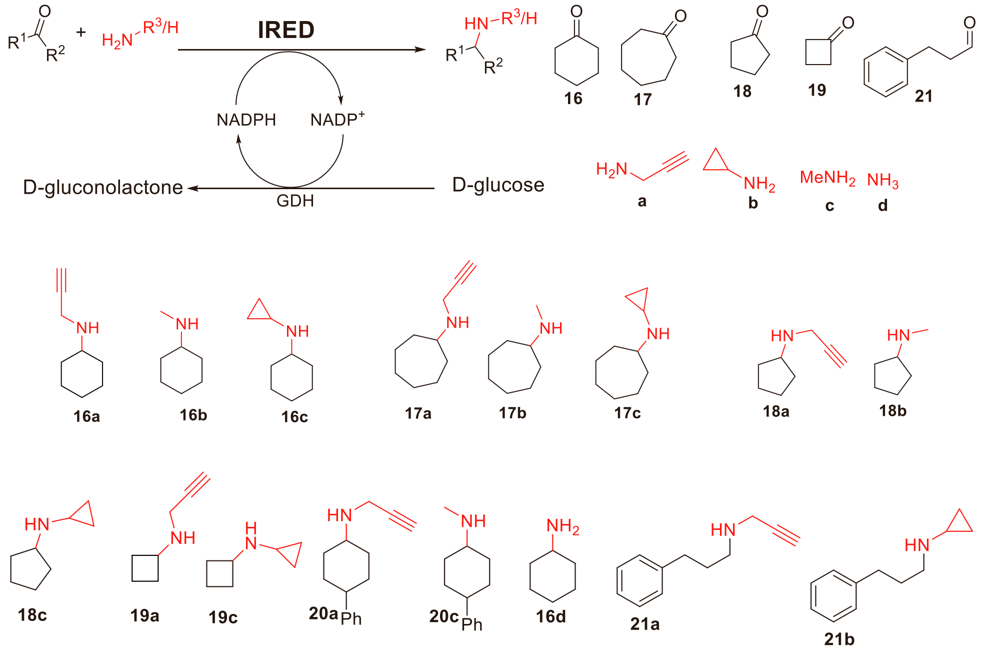

Ketone	Amine donor	Amine product	**Ketone retention time** [min]	**Amine retention time** [min]
**16**	**a**	**16a**	6.2	9.0
**16**	**b**	**16b**	6.2	8.9
**16**	**c**	**16c**	6.2	6.7
**16**	**d**	**16d**	6.2	5.7
**17**	**a**	**17a**	7.7	10.2
**17**	**b**	**17b**	7.7	10.2
**17**	**c**	**17c**	7.7	8.3
**17**	**d**	**17d**	7.7	-
**18**	**a**	**18a**	4.4	7.7
**18**	**b**	**18b**	4.4	7.7
**18**	**c**	**18c**	4.4	5.1
**18**	**d**	**18d**	4.4	-
**19**	**a**	**19a**	n.d.	6.5
**19**	**b**	**19b**	n.d.	6.3
**21**	**a**	**21a**	7.7	8.7
**21**	**b**	**21c**	7.7	8.3
**21**	**c**	**21d**	7.7	7.0

Supplemental Experimental Procedures

Chemicals

Commercially available chemicals and reagents of the highest purity were purchased from Sigma-Aldrich (Poole, Dorset, UK), Fluorochem (Hadfield, Derbyshire, UK), or Thermo Fisher Scientific unless stated otherwise. Enzyme nicotinamide cofactors $NAD(P)^+$ and NAD(P)H were purchased from Cambridge Bioscience (Cambridge, UK). Media were purchased from Formedium (Hunstanton, UK).

General Procedure for chemical reductive amination. Secondary amine product standards were prepared as previously reported.[3] Briefly, 2 equiv. of $Ti(OiPr)_4$ was added to a solution containing 1 mmol ketone and 4 mmol amine in EtOH, and the reaction was stirred under Argon at room temperature for 18 h. The reaction was cooled on ice, and 2 equiv. $NaBH_4$ was added and stirred at room temperature for 1 h, after which 3 M HCl was added to quench the reaction. Then, 5 ml of water was added, and the solution was basified to pH 12 with 10 M aqueous NaOH. 10 ml of EtOAc was added to the basified solution and mixed vigorously, and the organic layer was collected. The aqueous fraction was further extracted with EtOAc, and the organic fractions were combined. 20 ml of water was added to the organic fraction, acidified to pH 1. The aqueous fraction was collected, basified to pH 12 with 10 M NaOH, and the amine product was extracted twice into dichloromethane (DCM). The DCM fraction was dried with anhydrous $MgS0_4$, and DCM was removed to recover the amine product. NMR data are in agreement with previously reported data.[3]

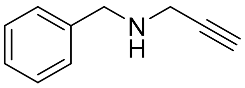

26a

Yellow oil isolated, 70% yield. **^{1}H NMR** (400 MHz, $CDCl_3$) δ 7.30 – 7.13 (m, 4H), 3.80 (s, 2H), 3.35 (d, *J* = 2.4 Hz, 3H), 2.18 (t, *J* = 2.4 Hz, 1H), 0.84 – 0.71 (m, 1H). **^{13}C NMR** (101 MHz, $CDCl_3$) δ 139.40, 128.46, 128.43, 127.19, 82.07, 71.58, 52.29, 37.35.

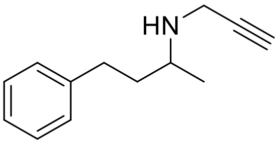

22a

Brownish-yellow oil isolated, 62% yield. **^{1}H NMR** (400 MHz, $CDCl_3$) δ 7.35 – 7.23 (m, 2H), 7.19 (ddt, *J* = 10.8, 6.3, 1.6 Hz, 3H), 7.16 (s, 1H), 3.53 – 3.35 (m, 2H), 2.99 – 2.86 (m, 1H), 2.79 – 2.58 (m, 2H), 2.19 (t, *J* = 2.4 Hz, 1H), 1.84 – 1.56 (m, 2H), 1.11 (d, *J* = 6.3 Hz, 3H). **^{13}C NMR** (101 MHz, $CDCl_3$) δ 142.29, 128.38, 128.36, 125.79, 82.37, 71.15, 51.15, 38.47, 35.61, 32.16, 19.81.

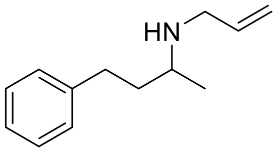

22e

Yellowish oil isolated, 73% yield. **^{1}H NMR** (400 MHz, $CDCl_3$) δ 7.41 – 7.28 (m, 2H), 7.28 – 7.19 (m, 3H), 5.97 (ddt, *J* = 17.1, 10.2, 6.0 Hz, 1H), 5.22 (dq, *J* = 17.2, 1.7 Hz, 1H), 5.20 – 5.09 (m, 1H), 3.35 (ddt, *J* = 13.8, 5.9, 1.5 Hz, 1H), 3.27 (ddt, *J* = 13.8, 6.2, 1.4 Hz, 1H), 2.84 – 2.63 (m, 4H), 1.86 (dddd, *J* = 13.5, 9.8, 6.4, 5.6 Hz, 1H), 1.78 – 1.63 (m, 1H), 1.17 (d, *J* = 6.3 Hz, 3H). **^{13}C NMR** (101 MHz, $CDCl_3$) δ 142.71, 137.48, 128.67, 128.65, 126.05, 115.98, 52.41, 50.13, 39.05, 32.65, 20.57.

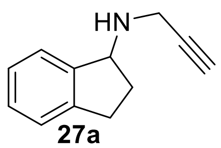

27a

Brow oil isolated, 58% yield. **^{1}H NMR** (400 MHz, $CDCl_3$) δ 7.40 (ddt, *J* = 6.7, 1.4, 0.8 Hz, 1H), 7.33 – 7.18 (m, 3H), 4.50 – 4.43 (m, 1H), 4.17 (q, *J* = 7.1 Hz, 1H), 3.57 (t, *J* = 2.6 Hz, 2H), 3.17 – 3.03 (m, 1H), 2.94 – 2.80 (m, 1H), 2.59 – 2.38 (m, 1H), 2.31 (t, *J* = 2.5 Hz, 1H), 2.04 – 1.85 (m, 1H), 1.35 – 1.22 (m, 1H). **^{13}C NMR** (101 MHz, $CDCl_3$) δ 144.48, 143.84, 126.69, 126.28, 124.89, 124.21, 82.46, 71.44, 61.89, 36.15, 33.31, 30.47

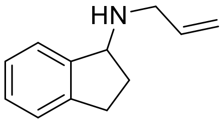

27e

Brown oil isolated, 67% yield. **^{1}H NMR** (400 MHz, $CDCl_3$) δ 7.40 – 7.30 (m, 1H), 7.27 – 7.14 (m, 3H), 5.98 (ddt, *J* = 17.1, 10.2, 5.9 Hz, 1H), 5.32 – 5.18 (m, 1H), 5.11 (dq, *J* = 10.2, 1.4 Hz, 1H), 4.27 (t, *J* = 6.6 Hz, 1H), 4.12 (q, *J* = 7.1 Hz, 1H), 3.45 – 3.27 (m, 2H), 3.01 (ddd, *J* = 15.9, 8.5, 4.9 Hz, 1H), 2.87 – 2.75 (m, 1H), 2.41 (dddd, *J* = 12.9, 8.3, 7.0, 4.9 Hz, 1H), 1.84 (dddd, *J* = 12.8, 8.5, 7.1, 6.1 Hz, 1H). **^{13}C NMR** (101 MHz, $CDCl_3$) δ 145.62, 144.05, 137.52, 127.84, 126.65, 125.19, 124.55, 116.23, 60.80, 50.47, 34.06, 30.78

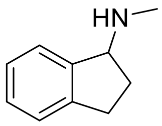

27c

Brown oil isolated, 56% yield. **^{1}H NMR** (400 MHz, $CDCl_3$) δ 7.29 – 7.20 (m, 1H), 7.20 – 7.03 (m, 3H), 4.11 – 3.95 (m, 1H), 2.90 (ddd, *J* = 16.0, 8.5, 5.2 Hz, 1H), 2.77 – 2.61 (m, 1H), 2.38 (s, 3H), 2.35 – 2.21 (m, 1H), 1.85 – 1.68 (m, 1H**).** ^{13}C NMR (101 MHz, $CDCl_3$) δ 144.19, 143.47, 127.30, 125.99, 124.53, 124.00, 64.37, 33.23, 32.29, 30.08

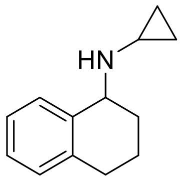

28a

Yellow oil isolated, 65% yield. **^{1}H NMR** (400 MHz, $CDCl_3$) δ 7.44 – 7.35 (m, 1H), 7.25 – 7.14 (m, 2H), 7.17 – 7.08 (m, 1H), 3.92 (t, *J* = 4.5 Hz, 1H), 2.93 – 2.73 (m, 2H), 2.36 – 2.27 (m, 1H), 2.12 – 1.92 (m, 3H), 1.88 – 1.75 (m, 1H), 0.65 – 0.38 (m, 5H). **^{13}C NMR** (101 MHz, $CDCl_3$) δ 139.73, 137.52, 129.37, 129.31, 126.93, 125.96, 56.08, 29.77, 29.08, 29.06, 19.32, 7.85, 6.59

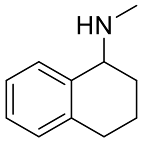

28c

Brownish-yellow isolated 52% yield**. ^{1}H NMR** (400 MHz, $CDCl_3$) δ 7.38 – 7.29 (m, 1H), 7.23 – 7.11 (m, 2H), 7.14 – 7.05 (m, 1H), 3.69 (t, *J* = 4.8 Hz, 1H), 2.89 – 2.66 (m, 3H), 2.51 (s, 3H), 2.07 – 1.92 (m, 1H), 1.92 (ddd, *J* = 6.3, 3.0, 1.7 Hz, 1H), 1.92 – 1.79 (m, 1H), 1.83 – 1.69 (m, 1H). **^{13}C NMR** (101 MHz, $CDCl_3$) δ 139.12, 137.72, 129.97, 129.45, 129.22, 127.09, 126.02, 57.40, 34.24, 29.68, 19.19.

Gene synthesis, cloning, expression, and protein purification.

Each gene sequence was optimised for *E. coli* expression, synthesised by Twist Biosciences, (South San Francisco, USA), and cloned into pET28a+ unless otherwise stated. Plasmid constructs of Bsp5 and Punc5 in pET22b have been kindly given to us by Dr Melanie A. Higgins. *Pv*DHFR, *Pf*DHFR, and *Sa*DHFR were each cloned into pET15b and were sourced from our in-house gene collections. A single colony of recombinant *E. coli* BL21 (DE3) containing pET28a/pET22b/pET15b-IRED was inoculated into 10 ml lysogeny broth (LB) (1% tryptone, 0.5% yeast extract, 1% NaCl, containing kanamycin 30 μg ml.−1 or ampicillin 50 μg ml−1) and incubated overnight at 37 °C in an orbital shaker at 200 r.p.m. This starter culture was used as the inoculum for subsequent growth.

Expression trial. Soluble expression of the target protein was trialled with isopropyl β-D-1-thiogalactopyranoside (IPTG) induction and LB autoinduction media (Formedium, Hunstanton, UK). A 250 ml flask containing 50 ml LB was supplemented with (30 μg ml.−1) or ampicillin (50 μg ml−1), depending on the construct, and inoculated with 0.5 ml of starter culture. For expression in LB media using IPTG induction, initial cultivation was performed at 37 °C in an orbital shaker with shaking at 180 r.p.m. At an optical density (OD600) of between 0.6 and 0.8, IPTG was added to a final concentration of 0.4 mM to induce protein expression. Incubation was continued at 22 °C with shaking at 180 r.p.m. for 18 h. For expression using autoinduction media, cultivation was initially performed at 37 °C in an orbital shaker with shaking at 180 r.p.m for 5 h, after which further cultivation was continued at 24°C for a further 40 h. Soluble expression was comparable for most of the investigated enzymes when using IPTG induction or autoinduction conditions.

Cell lysis was performed either by ultrasonication (5 × 20 s bursts with 40 s intervals at 4 °C) or incubation in lysozyme-based lysis buffer (100 mM Tris-HCl, 100 mM NaCl, pH 7.5, supplemented with 1 mg ml^{-1} lysozyme from chicken egg white, 0.3 mg ml^{-1} polymyxin B) incubated at 20 °C for 1h 30 min. Cell debris was removed by centrifugation (12,000 r.p.m, 4 °C, 30 min), and the clarified soluble cell-free extract was analysed by SDS-page gel electrophoresis, purified, or used for biotransformation.

Protein production and (semi)-purification. A 2-l flask containing 500 ml LB was supplemented with kanamycin (30 μg ml.−1) or ampicillin (50 μg ml^{-1}) and inoculated with 5 ml of starter culture. Cultivation was performed at 37 °C in an orbital shaker with shaking at 180 r.p.m. At an optical density (OD600 nm) between 0.6 and 0.8, isopropyl β-D-1-thiogalactopyranoside (IPTG) was added to give a final

concentration of 0.4 mM to induce protein expression. Incubation was continued at 22 °C and 180 r.p.m. for 18 h. Cells were then harvested by centrifugation and washed in Tris-HCl buffer (100 mM, pH 7.5).

For quick (semi)-purification, cells were resuspended in buffer A (50 mM Tris-HCl buffer, 300 mM NaCl, 25 mM imidazole, pH 7.5) and were disrupted by ultrasonication at 4°C employing 5 × 20 s bursts with 40 s intervals at 4 °C. Following centrifugation of the lysed cells (12,000 r.p.m, 4 °C, 1 h), the clarified lysate was purified by Ni-affinity chromatography using gravity columns. Clarified lysate was loaded onto the column, followed by a single-step wash with buffer A, and eluted with buffer B (50 mM Tris-HCl buffer, 300 mM NaCl, 300 mM imidazole, pH 7.5). The elute was desalted using 10 kDa cut-off centricon filters (15 ml) and the buffer was exchanged to Tris-HCL buffer (50 mM Tris, 100 mM NaCl, pH 7.5) or HEPES (30 mM, 100 ml NaCl, for BsP5, Punc5, NAPW).

Biotransformation Reactions. *For imine reduction using crude lysed cells*. An initial biotransformation reaction to assess imine-reducing activity was performed using crude lysed *E. coli* cells expressing an IRED. Biotransformation reactions typically contained *E. coli* BL21(DE3) resting cells containing expressed IRED (OD600 nm of 50), 0.3 mM NADP+/0.3 mM NAD+, 1-2% (v/v) DMSO, 5 mM imine substrate, 20 mM D-glucose, 0.3 mg ml^{-1} glucose dehydrogenase (GDH) as lyophilised cell free extract (CFE), 0.5 mg ml^{-1} lysozyme, and 0.25 mg ml-1 polymyxin B in sodium phosphate buffer (100 mM, pH 7.0, 100 ml NaCl). Reactions were incubated in an orbital shaker at 20 °C with shaking at 200 rpm for 48 h.
Biotransformation reactions using cell-free extract or (semi)purified enzyme preparation were performed employing glucose dehydrogenase (GDH)/NADP+ as a cofactor recycling system. A typical 500 µl reaction mixture contained 20 mM D-glucose, 0.3 mg ml^{-1} GDH (lyophilised CFE), 0.3 mM NADP+, 0.1-1 mg ml^{-1} IRED, 5 mM imine and 2% (v/v) DMSO. The reaction volume was made up to 500 µl with sodium phosphate buffer (100 mM, pH 7, 100 mM NaCl). Reactions were incubated at 25°C with shaking at 200 r.p.m. for 24 h.

For IRED-catalysed reductive amination of ketones or aldehydes. A 500 µl reaction mixture contained 50 mM ketone (30 mM for hydrocinnamaldehyde, and 10 mM for other carbonyl substrates), 30-100 mM D-glucose, 0.3 mg ml^{-1} GDH (lyophilised cell-free extract), 0.5 mM NADP+ in Tris-HCl buffer (100 mM, pH 9.0) containing 2% (v/v) DMSO. 2-10 equivalents of amine (from 1M buffered amine nucleophile solution stock solution, pH 9) were then added, and the reaction was initiated with the addition of 0.2-1 mg ml^{-1} IRED. The reaction volume was made up to 500 µl with Tris-HCl buffer (100 mM, pH 9.0). Reactions were incubated at 25 °C with shaking at 200 r.p.m. for 24 h.

For analysis, reactions were quenched by adding 60 µl of 5 M NaOH and extracted twice with 500 µl tert-butyl methyl ether. The organic fractions were combined and dried over anhydrous MgSO4 and analysed on HPLC using chiral columns (CHIRALPAK®IC 250 mm × 4.6 mm, 5 µm; and CHIRALPAK® IB N-5, 250 mm × 4.6 mm, 5 µm). Samples were run at 1 ml min^{-1} and monitored at 265 nm or by GC-MS (CD-5MS Capillary Column 30 m x 0.25 mm x 0.25µm (P/N MOD-GC-CD5MSU-5, S/N CD17506).

Preparative Biotransformation for the imine reduction of 1-methyl-3.4-dihydroisoquinoline (using MaRedAm, ZtNR, and LtPR1 as representative IREDs). Preparative biotransformation reactions for the reduction of the imine, 1-methyl 3,4-dihydroisoquinoline **8** (100mg, 0.69 mmol, total reaction volume, 28 ml) were performed in a 250 ml glass flask. The reaction mixture contained 100 mg imine **8** (25 mM), D-glucose (2.0 equiv., 50 mM), 0.6 mM $NADP^{+}$, and 2% v/v DMSO. The mixture was dissolved in 18 ml NaP*i* buffer (100 mM, pH 7), and the pH of the reaction mixture was adjusted to pH 7. The enzymatic components, including purified IRED (1 mg ml^{-1} *Ma*RedAm or 2 mg ml^{-1} *Zt*NR, or 2 mg ml^{-1} *Lt*PR1) and GDH as CFE (0.5 mg ml^{-1}), were then added to this reaction mixture. The reaction volume was then made up to 28 ml with NaP*i* buffer (100 mM, pH 7.0). The reaction flask was sealed and incubated at 25 °C in an orbital shaker with 140 rpm shaking for 24 h (*Ma*RedAm) or 48 h (for *Lt*PTR1 and *Zt*NR).

The biotransformation reaction was quenched by the addition of 5 M NaOH (basified to pH ~12) and extracted twice into EtOAc (2 x 20 ml) with intermediate centrifugation (4000 r.p.m, 5min, 10 °C). To the combined organic fractions, 20 ml of water was added, the pH was adjusted to pH 1 with 3 M HCl, and the aqueous layer was collected. The pH of the aqueous fraction was then readjusted to pH 12 with 5 M aqueous NaOH. To extract the amine product from the organic layer, 20 ml of dichloromethane (DCM) was added to the basified aqueous layer; this step was repeated. The combined DCM fractions were dried with anhydrous MgSO4, and the clarified DCM solution was recovered. DCM was then removed under reduced pressure to afford the corresponding amine **9.** Data from isolated product were

compared to commercial racemic amine standard **9** and *e.e.* values were determined using chiral HPLC, See Figure S8. *R*, *S*-configurations were assigned from comparison of isolated product with the enantiomer product obtained with IREDs of known selectivity (*Ao*IRED for the *S*-amine product and AspRedAm for the *R*-amine product.

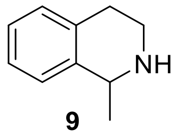

9

Yellow oil isolated. **^{1}H NMR** (400 MHz, $CDCl_3$) δ 7.41 – 7.22 (m, 3H), 4.35 – 4.25 (m, 1H), 3.45 (dt, *J* = 12.5, 5.1 Hz, 1H), 3.33 – 2.98 (m, 2H), 2.98 – 2.86 (m, 1H), 2.84 (s, 1H), 1.65 (d, *J* = 6.7 Hz, 3H), 1.03 (td, *J* = 13.7, 7.6 Hz, 1H). **^{13}C NMR** (101 MHz, $CDCl_3$) δ 138.43, 134.64, 127.53, 127.28, 125.93, 125.91, 51.55, 41.66, 27.86, 22.60.

Preparative biotransformation for the reductive amination of cyclohexanone with propargylamine (using MaRedAm, and LtPR1 as representative IREDs). Preparative scale biotransformation reactions for amination of cyclohexanone **1** (100mg, 1.02 mmol, total reaction volume, 41 ml with 2 equiv. of propargylamine **a,** were performed in 250 ml glass flasks. The 41 ml reaction mixture contained 100 mg cyclohexanone (25 mM), amine (2 equiv., 50 mM), D-glucose (2.0 equiv., 50 mM), 0.6 mM NADP+ and 2% v/v DMSO. The mixture was dissolved in 30 ml Tris-HCl buffer (100 mM, pH 8), and the pH of the reaction mixture was adjusted to pH 8.

The enzymatic components, including purified IRED (0.3 mg ml^{-1} MaRedAm or 1 mg ml^{-1} *Lt*PR1) and GDH as CFE (0.3 mg ml^{-1}), were then added to this reaction mixture. The reaction volume was then made up to 41 ml with Tris-HCl buffer (100 mM, pH 8.0). The reaction flask was sealed and incubated at 25 °C in an orbital shaker with 140 rpm shaking for 24 h or 48 h.

The biotransformation reaction was quenched by the addition of 5 M NaOH (basified to pH ~12) and extracted twice into EtOAc (2 x 20 ml) with intermediate centrifugation (4000 r.p.m, 5min, 10 °C). To the combined organic fractions, 20 ml of water was added, and the pH was adjusted to pH 1 with the addition of 3 M HCl, and the aqueous layer was collected. The pH of the aqueous fraction was then readjusted to pH 12 with 10 M aqueous NaOH. To extract the amine product from the organic layer, 20 ml of dichloromethane (DCM) was added to the basified aqueous layer; this step was repeated. The combined DCM fractions were dried with anhydrous $MgSO_4$, and the clarified DCM solution was recovered. DCM was then removed under reduced pressure to afford amine **16a.**

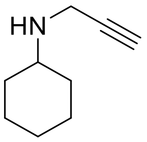

16a. Yellow oil isolated, (84% yield for MaRedAm-catalysed reaction and 63% yield for *Lt*PTR1-catalysed reaction) **^{1}H NMR** (400 MHz, $CDCl_3$) δ 3.42 (d, *J* = 2.5 Hz, 2H), 2.62 (tt, *J* = 10.3, 3.7 Hz, 1H), 2.16 (t, *J* = 2.4 Hz, 1H), 1.87 – 1.76 (m, 2H), 1.76 – 1.63 (m, 2H), 1.63 – 1.53 (m, 1H), 1.33 – 0.97 (m, 6H). **^{13}C NMR** (101 MHz, $CDCl_3$) δ 82.52, 70.96, 54.95, 35.07, 33.00, 26.07, 24.78. GCMS (EI) m/z = 137.18.

Supplemental: Gene Sequences

>LmPTR1
ATGACAGCTCCCACTGTACCAGTTGCACTAGTGACCGGCGCTGCTAAACGTCTGGGCAGAAGCA
TTGCTGAGGGCTTGCACGCCGAGGGCTACGCAGTTTGTCTGCACTACCATCGTAGCGCGGCGG
AGGCGAACGCGCTGTCTGCGACCTTGAATGCGCGTCGTCCGAATAGCGCGATCACCGTTCAAG
CAGACCTGAGCAACGTGGCCACGGCTCCGGTGAGCGGTGCGGACGGCTCTGCTCCGGTCACCT
TATTCACCCGTTGCGCAGAGCTGGTCGCCGCGTGCTATACCCATTGGGGTCGCTGCGACGTGCT
GGTGAACAACGCTAGCTCGTTCTATCCGACCCCGTTGCTGCGTAATGATGAAGATGGTCACGAA
CCGTGTGTTGGTGATCGTGAAGCCATGGAAACCGCGACCGCGGACCTGTTCGGCTCTAACGCC
ATCGCACCGTATTTTCTGATTAAAGCGTTTGCACATCGCTTCGCTGGTACACCGGCGAAACACCG
AGGTACGAACTATTCCATCATCAACATGGTTGATGCTATGACCAACCAGCCGCTGCTGGGCTACA
CGATTTATACCATGGCAAAGGGTGCGCTTGAGGGCTTGACTCGCAGCGCGGCGCTGGAGCTGG
CACCACTGCAAATTCGTGTTAATGGTGTCGGTCCGGGTTTGTCCGTGTTGGTAGACGACATGCC
GCCTGCGGTGTGGGAAGGCCACCGCAGTAAGGTTCCGCTGTACCAGCGTGATAGCTCCGCGGC
TGAAGTTTCCGATGTTGTTATTTTTCTTTGCAGCTCAAAGGCAAAGTACATCACTGGTACGTGCGT
CAAAGTGGACGGCGGTTACAGCCTGACCCGCGCATAA

>*Tb*PTR1
ATGGAAGCCCCTGCCGCAGTAGTAACGGGTGCCGCAAAACGTATTGGTCGTGCCATCGCGGTTA
AATTACATCAGACGGGTTATCGCGTCGTCATCCATTATCATAACTCGGCGGAAGCAGCCGTCTCG
CTGGCTGATGAACTCAATAAAGAACGTTCCAATACCGCCGTTGTTTGCCAAGCAGATCTTACCAA
TTCCAACGTTTTACCGGCATCCTGTGAGGAGATCATCAATTCGTGCTTTCGCGCGTTTGGTCGTT
GTGATGTCTTAGTGAATAACGCGTCCGCGTTTTACTCAACGCCCCTGGTGCAGGGTGATCACGA
AGACAACTCTAACGGCAAGACGGTGGAGACACAAGTCGCGGAATTAATCGGTACGAATGCAATT
GCCCCCTTTCTGCTCACCATGTCTTTTGCTCAGCGTCAGAAAGGGACGAATCCAAATTGCACATC
TTCAAACCTGTCTATCGTAAACCTGTGTGACGCGATGGTCGACCAACCTTGTATGGCGTTTTCTT
TATATAATATGGGCAAGCATGCGCTGGTCGGGCTTACCCAAAGCGCCGCCTTAGAACTCGCTCC
CTATGGTATCCGCGTAAATGGCGTAGCCCCGGGAGTTTCGTTACTGCCGGTAGCTATGGGCGAG
GAAGAGAAAGACAAGTGGCGTCGCAAAGTGCCACTGGGCCGCCGCGAAGCGTCTGCCGAGCA
GATTGCGGACGCCGTGATCTTCCTCGTGTCAGGCTCGGCACAATATATTACAGGCAGTATTATTA
AGGTTGATGGCGGCCTGTCATTAGTCCACGCGTAA

>*Tc*PTR1
ATGAACGAGACGTCTCACGAAGCAAGTGAATGCCCGGCCGCCATCATTACGGGTGGTGCCCGT
CGCATCGGCCATAGCATTGCGGTTCGTCTTCACCAACAGGGCTTTCGTGTGGTGGTTCACTACC
GCCACTCAGAGGGCGCTGCCCAACGTCTCGTGGCAGAGCTGAATGCCGCACGCGCTGGATCCG
CGGTTCTGTGCAAAGGAGATCTGAGCCTTGGATCATCGTTGTTAGATTGCTGTGAAGATATCATC
GATTGTAGTTTCCGTGCGTTTGGTCGCTGCGACGTACTGGTTAATAACGCCTCGGCGTACTATCC
CACTCCGCTGCTCCCAGGTGATGATACTAATGGAGCCGCAGATGCAAAGCCCATTGATGCTCAA
GTGGCCGAACTCTTCGGCAGCAATGTCGTGGCCCCTCTTTTCCTGATCCGTGCTTTTGCGCGCC
GTCAAGGCGAAGGCGATTCTTGGCGCAGTCGCAACCTTTCAGTGGTTAATCTGTGCGATGCGAT
GACCGACCTGCCTTTACCTGGATTTTGTGTATATACAATGGCGAAACACGCCCTGGGTGGGCTTA
CTCGCGCAGCAGCGTTAGAGTTGGCGCCGCGTCATATTCGCGTAAACGCGGTTGCGCCAGGCC
TGTCATTATTTCCGCCGGCAATGCCACAAGAAACGCAAGAGGAATATCGCCATAAAGTTCCACTG
GGACAAAGTGAAGCGAGCGCGGCGCAGATCGCTGATGCTATTGCCTTTCTGGTCAGCAAGGAC
GCAGGGCACATCACTGGGATCACCCTGAAAGTTGATGGCGGTTTGATTTTGGCTCGCGCGTAA

>*Lt*PTR1
ATGACGACCAGCCCGACGGCCCCTGTAGCACTTGTGACTGGAGCAGCTAAGCGTCTGGGTTCTA
GTATTGCGGAAGCGCTGCACGCTGAGGGATATACCGTATGCTTGCACTATCATCGTTCAGCGGC
CGACGCCAGCACGTTAGCAGCAACCTTAAATGCCCGTCGCCCTAATAGCGCCATCACCGTCCAG
GCCGACTTAAGTAATGTCGCAACGGCATCGTTTTCTGAGACGGATGGCTCCGTCCCAGTTACAC
TCTTCTCGCGCTGTAGCGCGTTGGTTGACGCCTGTTATATGCACTGGGGTCGTTGCGACGTTCT
TGTTAATAACGCATCAAGTTTCTACCCAACGCCGTTGCTGCGTAAGGACGCAGGTGAGGGCGGT
TCGAGCGTAGGTGACAAAGAAAGCCTGGAAGTAGCCGCTGCGGATCTCTTTGGCTCGAATGCCA
TTGCACCGTACTTCCTGATCAAGGCCTTCGCACAGCGCGTGGCCGACACCCGCGCAGAACAAC
GTGGAACGTCATACTCGATCGTGAATATGGTAGATGCCATGACCAGCCAGCCATTATTGGGCTA
CACCATGTACACCATGGCGAAAGAGGCACTTGAGGGCCTTACTCGCTCAGCAGCGTTGGAGCTT
GCAAGTCTTCAGATCCGTGTAAATGGTGTCTCGCCGGGCTTAAGTGTTTTACCTGATGATATGCC

TTTCTCGGTTCAAGAGGATTATCGTCGCAAGGTCCCCCTTTACCAACGCAACTCGTCGGCCGAG
GAAGTGAGCGACGTAGTTATCTTCCTGTGTTCACCGAAGGCCAAGTACATTACCGGCACTTGCAT
CAAGGTGGACGGCGGGTATTCGCTCACACGTGCGTAA

>*Am*PTR1
ATGACTAACTCGGGTCCGGTTGCTTTAATCACTGGTGCGGCGAAACGTATTGGCGCGGCCATGG
CCATGAAGCTCCATAATGAGGGCTACCGCGTTATCATCCACTATGGCCATAGCGAGAACGATGC
TCTTTCCCTGGCGGCTCGCTTAAACCAGAAACGTGCGAATTCAGCATTCTGTTTGCAGGCCGAC
CTGTGTGACACGCATGCCGTCAGTGCGCTGGGTGAAGAGGCTGTGAATGTGTGGGGTCGCTTG
GACGTCCTTGTAAACAATGCTTCCTCATTTTATCCAACGCCGGTGGGAGACATTACAGAAGAGGA
TTGGACTAGCTTAGTAGGTTCAAACGTTAAGGGGCCACTGTTCCTCAGTCAGGCCCTGACCCCC
GCCTTAAAGAAGTCAAACGGGTGCATTGTTAACATGGTAGACATGCATATTGATCGTCCCCTGCC
GAAGCATTCTGTGTATCTCCTTGCCAAGTCAGGCCTGGCTTCTCTGACACAGTCACTCGCAATTG
ACCTTGCGCCAAACATTCGTGTGAACGGTATTGGACCAGGGGCGATTCTCTGGCCTGAACGCGA
GATGGAAGATGCAGAGAAGGACACTCTGCTGTCGTCTATCCCTCTGGGAGAGCTTGGCACACCG
GATGACATCGCCAATACACTGTGGTTCCTGATCAGCGCGCCCTACATTACGGGCCAGATCATCTT
CGTGGACGGCGGCCGCTCACTGCACACAGGAGCGTCAGCATAA

>NpNR
ATGAGTTTAGAAAAAAGGTGGTCACTAGAGGGAACCACGGCTCTGGTTACCGGTGGCACCAAGG
GTATTGGCCGTGCGATCGTGGAAGAGTTGGTGGGTTTTGGTGCTCGTGTCTATACGTGCAGCCG
TAACGAAGCTGAGCTGCGTAAGTGCCTCCAAGAGTGGGAAAATCTGAAATACGACGTGACAGGC
TCCGTGTGTGATGTTTCGAGCCGCACCGAGCGCGAGAAGTTGGCCGAGGACGTCTCCTCTGTTT
TTAACGGCAAATTGAACATCCTGATTAATAACGCGGGTGGTTATGTGAACAAACCGATTGACGGC
TTCACCGCGGAGGACTTCAGCTTCCTGGTGGCCGTTAACTTGGAAAGCGCGTTTCATCTGTGCC
AGCTGGCCCACCCGATGCTGAAGGCCTCAGGCACGGGTTCTATTGTGCACATCAGCTCCAGCTG
CGCACAAATTGCGATTCCGGGTCATAGCATCTACAGTAGCACCAAAGGTGCGATCAACCAGCTG
ACCCGTAACTTGGCGTGTGAATGGGCAAAGGACAACATCCGCACCAATAGCATCGCACCGGGTG
CGATCCGTACCCCGGGCACCGAACCATTTGTTAATGATAAAGATGCGCTGGATCGTGAGGTTAG
CCGTGTTCCGAGCGGTCGCATTGGTGAACCGGAAGAGGTCGCGTCTCTGGCGGCTTTCTTATGC
ATGCCGTCCGCAAGCTACATCACTGGCCAGGTAATTTGTGTTGATGGTGGGAGAGCTATCAATG
GCTAA

>*NpaNR*
ATGAGACGGCGCGAAGAGGAATCACTGTCAATGCCAATGGAGAAAAAACGCTGGTCGCTGGTG
GGTGCAACGGCGCTTGTAACGGGGGGAACAAAGGGAATTGGCAGAGCTATCGTCGAGGAACTG
GCCGGATTTGGGGCGAGAGTGCATACGTGCTCGCGCAATAAAGCGGAATTGAACGTGTGCCTG
CAGGAATGGGAGAAACTGAAGTTGGATGTGACAGGATCAGTCTGCGATGTCTCTTCTCGTACAG
AACGAGAAAAACTGATGGAAGAGGTCTCTTCGGTATTTAATGGTAAATTGAATATTCTGATAAACA
ATGCCGGCACAGCGATCCTGAAGCCAATTCTTGATTTCACCGATGAAGATTGTTCCTTTTTAGTA
GCGACTAACTTTGAATCTGCGTTTCACTTGAGTCAGCTGTCGCATCCAATGCTGAAGGCGTCCG
GAGTTGGCAGCATTGTGCATATCAGTACAGTATGCACATTTATTGGCCTCGAAGGCCACTGTATT
TACAGCGCCACCAAAGGTGCTATGTCGCAATTGACGAGAGATTTTGCTTGTGAATGGGCACGTG
ATGGTATTCGTACGAACTGCATCGCTCCGGGTATTACCCGCACCGTACAGGTCCAACCGTTCCT
GGACGATAAAGACGCTGCGGCGAAGGAAATGAGCCGCATCCCGAATGGTCGCCCAGGTGAGCC
AGAAGAAATGGCTTCTCTTGCTGCGTTTTTATGCATGCCAGCCGCTTCATATATAAATGGACAGG
TTATTTGCGTCGATGGTGGGCGAGGAATCAATGGTTAA

>*Lr*NR
ATGCGTTGGAGTCTTCGCGGGCGCACTGCCTTAGTTACATCAGGTACGAAGGGTATCGGCCACG
CGATCGTTGAGGAGCTGGCGGGCTTAGGCGCTGTGGTATATACGTGTTCGCGCAATGAGACCG
AACTGAATAAGTGCCTGCAAGAATGGAAGAATCTTAAACTGAATATTAGCGGCAGCGTATGCGAC
GTTTCATCACGCCCCGAACGTAAGAAGTTAATGGAAAACGTGACGTCAATCTTCGACGGAAAAGT
TAATATTCTGATCAATAATGCCGCGTCTACACTGTATAAGGCGGTGGTGGACTGTACGGCTGAGG
ATTACAGCTTTATTATGGCGGCAAATTTTGAAAGCGCCTTTCACCTGTGTCAGCTGGCGCATCCG
ATGCTGAAAGCCAGTGGTACCGGGTCCATTGTGCATATTTCCAGCGCGTGTGCGGGTATCGCGA
TTCCTGGACATACGATCTACTCCAGCACCAAAGGGGCCATTAACCAACTGACCCGCAACCTCGC
GTGCGAATGGGCCAAGGACAACATTCGCACGAATAGCATTGCTCCTGGCGCCATTCGCACTCCC
GGGACTGAGAGCTTCGTCGAAGACAAAGACGCCCTGGACCGCGAAGTGTCGCGCATCCCTTTT

GGTCGTATTGGGGAACCCGAGGAAGTGGCATCGCTGGCAGCCTTTCTGTGTATGCCGAGCGCG
AGCTATATTACCGGCCAGGTGATTTGTGTTGACGGTGGCCGCACGATTAACGGGTAA

>*Zt*NR
ATGCGTTGGAGTCTTAAAGGTACTACAGCGCTTGTTACGGGCGGCACGAAGGGGATCGGTCAC
GCGATCGTGGAAGAATTGGCTGGTTTTGGGGCTCGCGTGTATACTTGTAGCCGCAACGAAGCCG
AGCTGACCAAATGCTTGCAAGAATGGGAAAATTTAAATTTCGACGTGGCAGGAAGCGTGTGTGA
CATTGCCTCACGTACCGAACGCGAGGAGCTGATGGAGCGTGTATCAAGCGTGTTCAACGGAAAC
CTGAATATCTTAATTAATAATGCGGGTGGTTACGTGAACAAACCAATCGACGATGTTACCGCCGA
AGATTTTAGTTTTCTGGTGGCGGTAAATCTCGAAAGCGCATTTCATCTCTGCCAGCTCGCGCACC
CTATGCTCAAAGCATCGGGTCGTGGTAGCATTGTCCATGTGAGCTCGTGCTGCGCTCAGATCGC
GTTACCTGGGCATTCGATGTATTCTGCCACCAAGGGAGCAATTAATCAGTTGACCCGTAATCTCG
CGTGTGAGTGGGCGAAAGATAACATTCGCACCAACACCGTAGCTCCGGGAGCCATTCGCACGC
CGAGTTCGGAACCGTTTGTCAATGACAAAGATGCGGTGGCTAAGGAAGTCGCGCGCGTACCGCT
CGGCCGTATTGGGGAGCCGGAAGAGGTAGCTGCCATCACGGTATTTCTGTGTATGCCGGCTGC
CAGTTATATTACAGGTCAGGTCATTTGTGTCGATGGCGGCCGTACGATCAACGGCTAA

>*Ls*NR-like
ATGGATTTCAGCTGGCAGTTAACCCATCAACGCGCCCTGGTCACGGGTGGCACCAAAGGTATCG
GTCGTGCAATCGTCCAGCAATTGCTTCAGTTCGGTGCTTCTATCTTTATCGTTGCACGCGATAAC
GCCCTTCTGCAACAGCAATTACAGGAATACCGTCAAAACGGCTTTTCGGTCGACGGCCTTGCCA
CGGATATTTCGCAGCCTGGAGCCGCACAACGCTTAGTTGAAACCGTCCAAGACCGTTGGGGTTC
ATTAGACATCCTGATCAACAACGCGGGAACCAACATCCGCAAACCTACCACCGACTACCGCCCG
GATGAATTTGACCAGATTCTGAACACTAACCTTCGCAGCGCCTACGAGCTGTGCCAGGCTGCGT
ATCCACTGCTGAAAAGTAGCGGAAAATCTCGCATTGTGTTTGTGAGCTCAGTAAGCGGCCTCAC
GCACACCTCTTCTGGTTCGATCTACGGGATGACTAAGGCAGCCCTGCACCAACTGACTCGCAAC
CTGGCCGTGGAGTGGGCACCGGACGGAATTCGTGTAAATGCAGTGGCACCCTGGTACATCCGT
ACGCCGCTTGCCGAACCGGTTCTTTCAGACCCGGAACGTCTCAATCGCATTCTGAGTCGCACGC
CGCTGGGCCGCATCGGCGAGCCAGAAGAGGTCGCAGCAACCGTCGCGTTTCTGTGTCTTCCCG
CAGCAGGTTACATCACCGGTCAGACACTGGCTGTAGACGGTGGAATGACTGCTTGGGGAATGTA
A

>*Pt*IRED
ATGTAAACCCGCTATAGCAGTACTAGGAGTTGGTCGTATGGGCAGCGCGCTGGTCAGCGCATTC
CTGAAACAGGGCTACGGCGTTGATATCTGGAACCGTACCCGTGCGAAGTGCGAACCGCTGGCG
GCTCAAGGCGCTCGCATTGCAGCGACGGTAAGAGATGCGGTGGCAGCTGCGGACATCGTGGTG
GTCAACGTAAACGATTATGGTACTTCCGACTCTCTGTTACGTCCGGACGAAGTTACCCGTGCATT
GCGCGGTAAACTGCTCGTCCAGCTGACCTCCGGCTCCCCGAGCCAGGCCCGTGAGCAAGCGAC
GTGGGCACGCCAGCACGGCATTCCATACCTGGACGGCGCGATTATGGGTACTCCGGACTTCAT
CGGACAGCCGGGTGGCACCCTGCTGTACAGCGGTCCGTCGGAACTGTTCGAGCAATATAAACC
GGTGTTGTTGACCTTAGGTGGTAATACCCAGCACGTGGGCGCGGACGTTGGCCACGCAGCGGC
TCTCGACAGCGCGCTGCTGGTGTATTTATGGGGTGCTATGTTCGGCGTTTTGCAAGGCGCGGCT
GTGTGTGAAGCGGAAAAGGTGCCGCTGGAGAGCTTTATGGGTTACGTCAAGGCTACCAGCCCT
GTGGTTGAAGGTGCCGTGACCGATGTTCTTATGCGTGTTCAGCAAGGCCGTTTTGGTTCTGACG
CGACCACCCTGGCCACGTTGGAGATCCACCATGGTGCGTTGCGCCACCTGCTGGAGCTGTGCC
GTGAGCGCGGTCTGCATCGTGAGATGCCGGAAGCATTTGATCGTCTGTTCCAGAAAGCGCTGCA
AGCCGGTCATGCCCAAGATGATTTTGCAGTTCTATCACGTTTTATGCGCTAA

>*Bac*RedAm
ATGAGGGAACCCATAGTAAGTGCTCACACAGAGCGCGCAGTCGAGTCTCGTGGCGCGGACCGT
GGTTCTGCGGTTACCGTCATCGGCTTGGGTTCCATGGGTTCAGCCCTCGCCGGCGCGGTGCTG
GAAGCGGGCTATCCGACGACCGTTTGGAACCGCACGGCTGGTAAGGCAGAACCATTGGTGCGT
AGAGGCGCGGCTCGCGCGGCGACGGTGGCGGAGGCGGTGAGCGCGTCCCCGACCGTGATCG
CCTGCGTGCTGGATTATCGTGCGTTACGTGAGATCCTGAGCACCGCGGGCGACGCACTCGCTG
GCCGTACCGTTGTTAATCTGACCAACGGTACACCGACCGAGGCCCGTGAAACCGCTGCTTGGGT
CGAGGGTCATGGTGCTCGTTACCTGGACGGCGGCATCATGGCAGTTCCGGAAATGATTGGTGG
CGCGGAAAGCCTTGTTCTGTATAGCGGTAGCGCCGAGGCGTTTGAAACCGTAGAGCCGGTTCTG
CGTCGCTTCGGCAGCGCTATGTACCTGGGTGCGGACCCGGGTTTGGCCTCGCTGCACGATCTG
GCATTGCTGGCGGGGATGTACGGCCTGTTTGCAGGCTTCCTGCACGCAGTGGCCTTAGTGGGT
ACGGAAGGTGTTCGTGCCACCGAGTTCACCAGCTCCCTGCTGATTCCGTGGCTGCAGGCCATGA

CCGCGACTCTGCCTGAAGCTGCGGCGCAAATTGATGCAGGCGACTACGCAGCGACTGGTTCTC
GCCTGGACATGCAAGCGGTCGCGTTGGCGAACATTGTTGAGGCGAGCAGAAGCCAGGGTATCC
GTCCGGATCTTATGCTGCCGATTCAGGCATTGGTTGAGCGCCGTGTGGCAAAAGGTGGTGGCG
GTGAAGATATCGCAGCGGTGGTGGAGGAAGTTCGCGGATAA

>*Ma*RedAm
ATGACTTCTTCCTCCACTGTTAGCATTATCGGCCTTGGCGCCATGGGCTTGGCCCTTGCTGCCAA
GTTTGTGGAGAAGGGCTACACGACCACTGTTTGGAACAGATCCACCGAGAAGGCACTCAAGTTT
GCTGCAGAGCACGAGAACGCGCATGCCGCAACCACCGTGGCTCAAGGTCTAGAGGCTAGCAAC
CTGGTGATCATCTGTCTTCTCGACAACAAGGCCGTTCGCGATACCATTGATCAAGCCCTTCCCTC
CTTGGCGGGACGCATCGTTGTCAACCTGACTAACGGTACCCCTGATGAGGGACGAGAGACTGG
GGCCCTTGTGGCGGCTCAGGAAGGATCCAAGTACGTGCACGGCGGGATCATGGCAACTCCTTC
GATGGTCGGCTCTCCGGCATCGGTGCTGCTGTACAGCGGATCTCTAGAAGCTTACACAGCGGTG
GAGAAGGATCTCGAGATCCTGGGTGCTGGCAAGTACCTCGGAGCCGACTCTGGATCGGCTTCG
CTGCATGATCTGGCGCTCCTGAGCGGGATGTATGGCCTCTTCTCTGGCTTTACGCATGCAGTGT
CGCTGGTGCGGAACGAGAAGCGGTCGACCACGGAATTCCTGTCGCTTTTGGTGCCCTGGCTGA
CGGCGATGACGGGCTACCTGCACGTGCTGGGCAAGCAGATTGATGAGGGCGACTTCTCGTCTC
TTGGGTCGAGTATTGAGATGCAGGTGCCTGCGATCAACAACATTGTGAAGACAAGCGAGGCACA
GGGCGTGTCTGCGGATCTCATCCGGCCCATCCAAGGCTTGCTGGAGCGTGCGGTGGCGGTCG
GACGAGGTGGCGAGGAGATCTCGGCGCTGGTGGGCCTGAATGTGCTGGCGAGGAAGGCAGAG
TAA

>AserRedAm
ATGTCGAAGCACATAAGCATATTCGGCCTCGGTGCTATGGGAACAGCTCTCGCAGCCAAGTATC
TCAGACATGGCTATAACACAACCGTTTGGAATCGAACCACTGCAAAGGCAACTCCGCTCGTTGA
GCAGGGTGCCAAGCTAGCCTCTACAATCTCGAAGGGGGTAGACGCCAGCGACCTCATCGTTATA
TGCCTCCTTAACAATCAAGTTGTCGAGAACACTCTACGGGATGCATTACACATTCTGTCCAGTAA
GACCATTGTCAACCTTACCAATGGGACACCAAACCAGGCTCGTAAGCTCGCAGATTTCGTCACCT
CCCACGGAGCACGGTACATCCACGGCGGCATTATGGCGGTTCCCACCATGATTGGCTCTCCACA
CGCTGTCCTGCTTTACAGTGGAGAGTCTCTTGAGCTATTCAAGAGCATCGAAAGTCATCTGTCTC
TCCTTGGGATGTGCAAATATCTCGGCACCGATGCAGGGTCTGCCTCTTTGCATGACTTGGCTCT
GTTGTCGGGAATGTACGGTCTCTTCTCCGGGTTCCTGCATGCGGTTGCTCTGATCAGGTCCGAG
CAGGATACCAGTACTGCCGCTACCGGGTTATTGCCGCTTCTGACTCCGTGGTTGTCGGCAATGA
CGGGATATCTTAGTTCTATCGCGAAACAGATCGACGAGGGTGACTATGCCACACAGGGATCTAA
CCTGGGAATGCAATTGGCCGGAATGGAAAACATAATCAGAGCTGGCGAGGAGCAGGGGGTCTC
CTCACAGATGATCCTTCCGATAAAAGCGTTGATAGAGCAAGCAGTGGGTGAAGGGCATGGGGGT
GAGGATCTATCAGCGCTGATTGAATACTTTGAGGCGGGAAAAAATGTGGATTAA

>RedE
ATGGGCGCGAAAGTAACCGTCCTGGGTCTCGGCCCGATGGGCGCGGCCCTGGCCGGAGCGTT
CCTCGCGGCTGGTCATCGTACGACCGTTTGGAACCGTACGCCGGGTAAAGGTGGCTCTCTGGC
TGGTGAAGGGGCAACCGAAGTTGCGAGCGCCGCAGAAGCGGTGGCAGCGAGCCCGCTGGTCG
TAGTCTGTTTGGCAACGTACGAAGCAGTACATGAGGTACTCGACCCTCTTGCTGATGAACTGGC
CGGTCGCACTGTTGTCAACCTTACCTCGGGTTCCCCGGTCCACGCCCGTGAGACCGCCAACTG
GGCTCAGCAACACGGCGCCGAATATCTGGACGGTGTTATTATGACGACGCCAAGTGGAATTGGG
AAGCCGGATTACCTGCTGCTGTATAGTGGCAGCCAGGCAGCGTTCGATGGCTCACGTGGTACG
CTGTGTGCATTGGGCGAACCAATGAATCTGGGTACTGATGCAGCGATGGCCAGCGTGTACGATA
CCGCATTACTCGGCTTAATGTGGGGTACTCTGACGGGCTGGCTTCATGGTGTGGCCTTGATGGG
TGCCGATGGGCCAGGCGGTAATGTCACTGCGACTGCGTTCACCGAAGTCGCTAATCGCTGGAT
GAAGACGGTGGGTGTGTTTATGAACACTTACGCACCCCATGTAGACGCGGGCCACTATCCCGGG
GATGAATTTACCTTACATCTGCATCATCGGACTATGAACATTCTGGCCCACGCATCGGAACTGCG
CGGTGTAGTCAGCGGTTTACCGGAGTTACTGACCGAGTTGACGGGGCGCGCAATCACGGCGGG
TCACGGCAATGACTCTTATGCGCGGCTGGTCGAATTCATTCGCAAAGATGGTAGTCCTATTTAA

>SeIRED
ATGAATAACGGCTTCGCTGCTCCTGTTACCGTCGTTGGACTCGGACCCATGGGGTGTGCGCTTG
CAGATGCGTTTCTCGCCGCTGGTCACCCGACTACAGTGTGGAATCGTTCGGCCCATAAGGCAGA
CCCGCTTGTGGCAAAGGGAGCCGTTCGTGCGGCTACCGCTGCTGAAGCACTCGCCGCCAGCGA
CTTAGTGGTGGTTTGTGTCGCGGATTATGCTGCGATGCGTGCAGCCCTCGATCGCACCGGCGCA
GAACTGAGTGGTAAAGTTCTCGTCAACCTCTGTTCCGGTACACCTCGCGAGGCCCGCGAGGCTC

TTACGTGGGCGACCGCGCACGGGGCTGGATATCTTGACGGTGCTATCATGGTGCCGGTTGAAG
TGATTGGTACCCCTAGTAGTGTTGTGTTTTATTCCGGTGCCCGTGAGCCATTTGACGCACACCGC
TCAACGTTAGATGCCCTGGGTGGGGTGCCTCGCTACCTGGGAGATGACGCTGGGCTGGCAGTA
TTACACAATACCGCGCTTTTGGGCTTGATGTGGGCTACGGTAAATGGGTTTCTTCACGCAGCCG
CGCTCGTGGAAAGCGCGGGTGTCGGAGTGGCTGACTTCGCCGAGACGGCAGTGGATTGGTTTC
TGCCCTCGGTTATTGGCGAGATCTTACGTGCTGAGGCCGCTCGCATTGACCGCGGTGAGTTCCC
TGGCGATGGCGGGACCCTTGCGATGTGTTTAACAGCAATTGAACATATTGTTCGTACCTCGCGC
GATGCTGGAATCAGCGACGAGGTGCCGAGCCAGTTGAAGACATTGGGCGATCGTGCCGTAGCG
GCAGGTCACGGCGACGAGAACTACATGTCACTTATCAAGGTACTGCGCGTGCCTTCGACTGGTG
CTCATCGTTAA

>*Ss*IRED
ATGAGTGAGAAGAAGCTGGCAGTTACCGTAGCAGGACTGGGGCCAATGGGTTCAGCCCTGGCC
GCGGCACTGCTTGATCACGGCCACGAAGTCACGGTATGGAATCGTAGTCCTGGTAAGGCTGCG
CCGTTAGTAGCACGCGGTGCCCGCGAAGCGGATGGCGTGGCTAGCGCAGTGAGCGCGTCTGA
AGTAACTGTGGTGTGCCTTGCCGACTACAACGCGTTATACTCGGCGTTACGCCCAGCAGAGGCG
GCTCTTCGTGGACGTGTGGTTGTGAATCTTAATAGCGGCACCCCCAAAGAGGCACACGAAGCAG
TCCGTTGGGCGGAAGACCGTGGTATCGGTTACTTGGACGGAGCTATTATGGTTCCACCCGCGAT
GGTCGGACGCCCAGGCAGCGTATTCCTGTATTCTGGTGCAGAGGATGTATTTGATGCACACAAG
GCGACCCTGGCGGTTCTGGGTGAGGCAACGTACCTTGGTGCAGACCCAGGCCTTGCCGTACTT
TACAATACGGCACTTCTGTCTTTGATGTACAGCAGTATGAACGGCTTTCTGCACGCTGCGGCGCT
GGTTGGCTCGGCGGGAGTTGCAGCGACGGACTTCACAAAGCTGGCTGTTGACTGGTTCTTACCT
AGTGTGGTCGGCGGCATTTTAGAAGTTGAGGCTCCAGCGATCGACAATGGTGTTTACCCTGGTG
ATTTAGGCTCATTAGAGATGAACTTAACGGCCTTGACCCATATTGTAGGTACATCCGGCGAACAA
GGCGTAGACACGGAAATTCCTGCCCGCAATAAAGAGTTAGCGGAGCGTGCAATTGCGGCCGGT
TTCGGTAAGTCTAGCTACTCTTCAATTATCGAAGTGCTGAAGAAATAA
>*Ar*IRED
ATGAGTGAGAAGAAGCTGGCAGTTACCGTAGCAGGACTGGGGCCAATGGGTTCAGCCCTGGCC
GCGGCACTGCTTGATCACGGCCACGAAGTCACGGTATGGAATCGTAGTCCTGGTAAGGCTGCG
CCGTTAGTAGCACGCGGTGCCCGCGAAGCGGATGGCGTGGCTAGCGCAGTGAGCGCGTCTGA
AGTAACTGTGGTGTGCCTTGCCGACTACAACGCGTTATACTCGGCGTTACGCCCAGCAGAGGCG
GCTCTTCGTGGACGTGTGGTTGTGAATCTTAATAGCGGCACCCCCAAAGAGGCACACGAAGCAG
TCCGTTGGGCGGAAGACCGTGGTATCGGTTACTTGGACGGAGCTATTATGGTTCCACCCGCGAT
GGTCGGACGCCCAGGCAGCGTATTCCTGTATTCTGGTGCAGAGGATGTATTTGATGCACACAAG
GCGACCCTGGCGGTTCTGGGTGAGGCAACGTACCTTGGTGCAGACCCAGGCCTTGCCGTACTT
TACAATACGGCACTTCTGTCTTTGATGTACAGCAGTATGAACGGCTTTCTGCACGCTGCGGCGCT
GGTTGGCTCGGCGGGAGTTGCAGCGACGGACTTCACAAAGCTGGCTGTTGACTGGTTCTTACCT
AGTGTGGTCGGCGGCATTTTAGAAGTTGAGGCTCCAGCGATCGACAATGGTGTTTACCCTGGTG
ATTTAGGCTCATTAGAGATGAACTTAACGGCCTTGACCCATATTGTAGGTACATCCGGCGAACAA
GGCGTAGACACGGAAATTCCTGCCCGCAATAAAGAGTTAGCGGAGCGTGCAATTGCGGCCGGT
TTCGGTAAGTCTAGCTACTCTTCAATTATCGAAGTGCTGAAGAAATAA

>*Ps*DRR
ATGGAATCAAATGGAGTACCCATGATAACACTATCCAGCGGTATCCGCATGCCGGCTCTGGGTA
TGGGTACTGTTGAAACCATGGAGAAGGGCACTGAACGCGAGAAGCTCGCGTTTCTGAAGGCGAT
CGAGGTGGGCTATCGTCACTTCGATACCGCGGCCGCCTACCAGACCGAAGAGTGCCTTGGCGA
GGCGATCGCTGAAGCTCTGCAACTGGGTCTGATCAAAAGCCGTGAAGAGCTGTTCATCGCGTCG
AAGTTGTGGTGTACCGATGCGCACGCAGACCTGGTGCTGCCGGCACTGCAGAATTCTCTGCGCA
ATCTGAAGCTGGAATACTTGGACCTGTATTTGATTCATTTTCCGGTGTCCCTTAAGCCGGGTCGT
ATTGTTAGCGATATTCCAAAAGACCAGATGCTGCCTATGGACTACAAATCTGTTTGGGCTGCTAT
GGAGGAGTGCCAGACGCTGGGCTTTACCCGTGCAATTGGCGTTTCTAATTTCAGCTGCAAGAAA
TTGCAAGAGCTGATGGCTACCGCGAACAGCCATCCGGTTGTTAACGAAGTGGAAATGTCCCCGG
TCTTTCAGCAAAAAAACTTGCGTGCGTACTGCAAGGCCAACAACATTATGATTACCGCATACAGC
GTTTTAGGCGCGCGTGGTGCGGCGTGGGGTAGCAACGCAGTAATGGACAGTAAAGTGCTGCAC
GAAATCGCGGTCGCCAGAGGCAAAAGCGTGGCACAGGCATCCATGCGTTGGGTTTATCAACAAG
GTGCGTGTTTGGTCGTGAAATCGTTCAACGAGGAGCGCATGAAAGAAAATTTGAAAATCTTCGAT
TGGGAATTAAGCGCGGAAGATATGGAGAAGATCAGCGAAATCCCGCAGTGCCGTACCTCAAGCG
CTGACTTCCTGCTGAGCCCGACGGGTCCGTTTAAAACGGAGGAGGAGTTCTGGGATGAAAAGGA
CTAA

>BsP5
ATGAAGATAACATATATTGATAAACCCACTTACTTGCCAAGCTGGGTCATCAACAAGATTAACGAA
TATGGTGATTTCGAGGTATTCTACGACTTCCCGAACGAAGAAGAGGCGATTAATAGACTGTCGAG
CACCGACATCGCGATTGTTGAATGGACCAGCATCACGAAAGAAATGATTGAGAAGATCAGTCGT
CTGAAGTACCTGATAACCATTACCACCAGCTACGATTATATCGACGTGAACAGCCTAAAGGACAA
CGAAATCATGGTTAGCAACTGCCCGCAGTATTCCAAACAGGCTGTGGCGGAGCACGTGTTTGCA
CTGTTGTTTGCGGTGAATCGCAAAATCCTGCAGGCCGATGAGACGTGCCGTAAAGGTTTGTCCC
ATATCTACCCGCCTTTTCTGTGTAGCGAGATTCGTGATAAAACCATTGGTCTTATCGGCATTGGTC
AGATTGGCCAAACTGTTGCCGAAATCGCTAATGCGTTCCAAATGAAAGTTATTGGTCTCAACAAG
TCCAAGCGCAACGTGAAAGGTATTCAACAGGTTGATATCACGGAGCTGATGAAAAAGTCCGACAT
CATCAGCTTGCACATTCCGCGTAACGCTGACACCGAAATTATCTTGACCGAGAAGCTGCTTTCTC
TGATGAAGCCGGACGCGGTGCTGATTAACACCTGCCGTGGCAATCTGATCGACGAGCAGGCAC
TCTATAGCGTTCTGAAGCAAAACCGCATCCGTGGCGCGGGCTTAGATGATCTGACCTACTACAAA
GACAACCCGATTATCGGCCTGAATAATGTCGTTCTGACACCGGGTTCGGCATGGTATTCTTAA

>*Pp*DPKA
ATGAGTGCTCCCTCTACATCAACTGTAGTTCGCGTTCCGTTCACCGAACTGCAAAGCCTGCTACA
AGCTATTTTTCAGCGTCATGGTTGTTCGGAAGCGGTTGCGCGCGTGCTGGCGCACAACTGCGCG
TCTGCCCAACGCGATGGAGCGCACAGCCACGGCGTGTTTCGTATGCCGGGCTACGTTTCCACC
CTGGCAAGCGGTTGGGTTGACGGCCAGGCAACGCCGCAGGTTAGCGACGTGGCTGCTGGCTAC
GTGCGCGTCGACGCTGCAGGCGGCTTTGCACAGCCGGCGTTGGCAGCGGCGAGAGAATTGTTG
GTTGCCAAGGCGCGTTCTGCAGGCATCGCGGTGCTGGCGATCCACAACAGCCATCATTTCGCTG
CTCTGTGGCCAGATGTGGAGCCGTTCGCGGAGGAGGGTCTGGTGGCTTTGTCCGTGGTTAATA
GCATGACGTGCGTTGTCCCGCATGGTGCGCGTAAACCGCTCTTCGGTACAAATCCGATCGCCTT
TGCGGCTCCGTGCGCGGAACACGATCCGATTGTTTTCGACATGGCGACGAGCGCTATGGCACA
CGGCGATGTACAGATTGCGGCTCGCGCAGGTCAGCAGCTGCCAGAAGGTATGGGTGTGGACGC
CGATGGCCAACCGACCACCGATCCGAAAGCCATCCTGGAAGGCGGCGCGCTGCTGCCGTTCGG
CGGCCACAAGGGTTCCGCGCTTAGCATGATGGTTGAGTTACTGGCGGCGGCATTAACTGGTGG
CCATTTCTCTTGGGAATTTGACTGGTCCGGTCATCCGGGTGCCAAGACCCCGTGGACCGGTCAA
CTGATTATCGTGATCAACCCGGGTAAGGCAGAGGGCGAACGTTTTGCGCAACGTAGCCGTGAGT
TGGTCGAGCACATGCAGGCGGTTGGTCTGACCCGTATGCCTGGTGAGCGTCGTTATCGTGAGC
GCGAGGTGGCCGAAGAGGAGGGGGTTGCGGTCACCGAACAGGAGTTACAAGGTCTGAAAGAAT
TGCTGGGTTAA

>*Cr*THAS
ATGGCTATGGCAAGTAAATCACCCTCTGAAGAGGTTTATCCGGTTAAGGCATTCGGCCTTGCCG
CGAAGGATTCCAGCGGTCTGTTTAGCCCGTTTAATTTCAGCCGCCGTGCAACCGGTGAGCACGA
TGTTCAACTGAAGGTGCTGTATTGTGGTACGTGCCAGTATGATCGTGAAATGAGCAAAAACAAAT
TCGGTTTTACCAGCTATCCGTACGTGCTGGGCCACGAAATAGTTGGTGAGGTAACTGAAGTGGG
CTCCAAAGTGCAGAAATTCAAAGTCGGAGATAAAGTCGGTGTTGCTAGCATTATCGAAACCTGCG
GTAAGTGCGAAATGTGCACCAATGAAGTCGAGAACTACTGCCCGGAGGCTGGCTCCATCGACTC
AAATTACGGTGCGTGTAGCAACATCGCCGTTATCAACGAGAACTTCGTCATTCGTTGGCCCGAGA
ATCTGCCGTTAGACTCTGGCGTTCCGCTGCTGTGTGCAGGGATTACCGCTTACAGCCCGATGAA
GAGATACGGCTTAGACAAACCGGGTAAACGCATTGGCATCGCGGGCCTGGGCGGCCTGGGTCA
TGTTGCGCTGCGTTTTGCAAAAGCCTTCGGCGCGAAGGTGACCGTGATTTCTTCGTCCTTGAAG
AAAAAGCGCGAGGCGTTCGAAAAATTTGGTGCCGATTCTTTTCTGGTTAGCTCCAACCCGGAGG
AAATGCAAGGTGCTGCTGGTACTCTCGACGGCATCATTGATACCATTCCGGGTAACCACAGCCT
GGAACCGCTGTTGGCGCTGTTGAAGCCATTGGGTAAGCTGATTATCCTTGGTGCGCCAGAGATG
CCGTTTGAAGTGCCGGCACCGAGCTTGCTGATGGGTGGCAAGGTGATGGCGGCGTCGACCGCA
GGTAGCATGAAAGAAATCCAGGAGATGATTGAGTTCGCGGCGGAACATAACATCGTGGCGGATG
TTGAGGTTATCTCGATCGACTACGTCAATACGGCTATGGAGCGTCTGGACAACAGCGACGTGCG
TTATCGTTTCGTGATCGACATCGGCAATACCCTGAAGAGCAACTAA

>*As*IRED
ATGACAGATCAAAATCTACCCGTTACTGTAGCTGGCCTGGGTCCGATGGGTGCGGCGCTCGCTG
CGGCGTTGCTGGACAGAGGCCACGATGTTACCGTTTGGAACCGTAGCCCGGGCAAGGCTGCTC
CGCTGGTGGCAAAAGGTGCGCGTCAAGCGGACGACATCGTTGACGCGGTTAGCGCGAGCCGTC
TGTTGGTGGTGTGCCTGGCGGATTACGATGCACTCTACTCTGCCCTGGGCCCAGCGCGCGAGG
CGTTGCGTGGTCGTGTGGTGGTAAATCTGAACAGCGGTACACCGAAAGAGGCCCGTGAAGCAG
CTCAGTGGGCAGAAGGTCACGGCATCGGTTACCTGGACGGCGCGATTATGGTTCCGCCACCTC

TCGTCGGTCACACTGGTTCCTTGTTTCTGTACAGCGGTTCCACCGAGATCTTTGAAACCCATAAA
GAAACTCTGGCGGACCTGGGTGATCCGGCGCATCTGGGCACGGATCCGGGTCTGGCGGTGCT
GTATAACACCGCATTGTTAAGCATGATGTATTCTAGCCTGAACGGCTTCTTGCATGCGGCCGCTC
TGGTGGGCTCTGCGGGTGTTGGTGCGGCAGAGTTCACCGAGATTGCAGTCGACTGGTTCCTGC
CGTCGGTCATCGGCGGTATCATCAAAGCCGAGGCTCCGACCATTGATAAGGGCGAATATCCGG
GTGAACTGGCGAGTTTGGAAATGAATGTTACGACCTTGAAGCACATTATCGGCACCAGCGATGA
ACAGCGTGTTGACGCTGGCATTCCGGCAGGAAACAAAGAGCTGCTGGACCGCGCAGTGGCCGC
GGGCTTTGGTAAGAGCGGCTACTCCTCCGTGATTGAGGTTCTTAAGCGCGGTGCGGCTTAA

>*Ec*SaRed
ATGCGCTGATAGTTCAAAAAAGTTAACAGTACTACTGAGCGGCGCATCCGGCTTGACTGGTTCTC
TGGCCTTCAAAAAACTGAAAGAACGTAGTGATAAATTCGAGGTACGCGGTTTAGTTAGATCCGAG
GCCAGCAAGCAGAAGCTGGGTGGCGGCGATGAGATCTTTATCGGCGACATCAGCGACCCAAAA
ACCCTGGAACCGGCAATGGAAGGTATTGACGCATTGATCATCCTGACCAGCGCAATTCCGCGTA
TGAAACCAACGGAGGAATTCACCGCGGAGATGATCAGCGGTGGCCGTAGCGAAGATGTCATCG
ACGCGTCGTTCTCCGGTCCGATGCCGGAGTTCTATTACGACGAGGGCCAATACCCGGAGCAAGT
CGATTGGATTGGTCAAAAGAACCAGATTGACACCGCGAAAAAGATGGGTGTTAAGCACATTGTTT
TGGTTGGTAGCATGGGTGGTTGTGATCCGGATCATTTTCTGAATCACATGGGCAATGGCAACATT
CTGATCTGGAAACGTAAAGCTGAACAGTACCTGGCGGACTCTGGCGTGCCGTATACCATTATCC
GCGCGGGTGGCCTGGATAACAAGGCTGGTGGTGTGCGCGAACTGTTAGTTGCTAAGGACGACG
TTCTGCTTCCGACGGAGAACGGCTTCATCGCCCGTGCGGACGTGGCAGAGGCGTGCGTTCAGG
CGTTGGAAATTGAAGAGGTGAAAAATAAGGCGTTCGATCTCGGCTCCAAGCCGGAAGGTGTGGG
TGAAGCGACCAAAGATTTTAAGGCGTTGTTTAGCCAGGTGACCACCCCGTTTTAA

>*Cg*EasG
ATGCAATTTTACTTACAGGGGGGAATGGCAAAACAGCTCGGCATATAGCCAGACTGCTGAAAGA
GGCTGATGTTCCATTTATAATCGGCTCTCGTTCTTCAACCTCGGAGATGATCGGTCACCACCGGA
GCTTTGACTGGCTGGACGAAGCGACCTTTGGGAACACACTTTCTGTCCATGGCGGCATGGAACC
GATCTCTATAGTTTGGCTGGTGCCCCCGCCGATCCTGGACCTGGCACCCCCAGTGATCTCATTT
ATTAACTTCGCAAATTCTAAAGGCGTGAAGCGTTTCGTACTTCTTTCGGGTAGTATTATTGATAAA
GGGGGTCCAGCCATGGGCCAGATTCATGCTCATCTGGATTCCCTGGAAGGAATCTCATATTCGG
TTCTTAGACCAACGTGGTTCATGGAAAATTTTTCAACACGTGGTGATTTACCCTTTGAAACAATTC
GGAAGGAAGGCAAATTCTACAGTGCCACGAAAGATGGCAAGATACCATTAATTTCAGTTATGGAT
ATTGCGCGTGTCGCTTTTCACGCACTGACCGCACCGGTGTTAGAAAAAGAGGAGCATATATTACT
GGGGCCTGAACTGTTGACGTATGACGATGTCGCAGAAACGCTGAGCCGCGTACTCGGGCGTAA
TATCATTCACGCCCGTGTAACGGAGAGTGAGCTGGCAGAAAAGCTGCAAGACAACGGTCTTACA
GCAGAAGATGCAGCAATGCAAGCATCTTTGGATCTTATTATCTCAGCAGGCGGCGATGAACGCC
TGAACACCGAGGTTCGTGACCTTACAGGCCAAGAGCCGCGACGTTTTAGTGACTTTGTGTCTGA
AAATAAGCATGCATGGATTTGCTAA

>*Pa*PchG
ATGCGGATGTGAGATCGGTAGTTGTCGCTGGATCGAGATTTGGCCAATTTTATGCCGCCGGTGT
GGCAGCCGATCCTCGCTTTGTTTTACGCGGGATCTTAGGACAGGGATCAAGACGTTCGCGAGCA
CTCGCGGAACGGCTTGGGGTTGAAACCTGGTGTGAAGTAGAAGCGTTACCGGATGACGTAAGA
CTGGCCTGCGTAGCGGTCGGAGGCGCTGCACGGGGGGAGCAGGGTCCGGCGCTGGCTGAAG
CTCTGATGGCACGCGGAATTGACGTCCTGATCGAACATCCCCTGCTGCCGCGAGAATGGCAAGA
TCTGTTACGATCTGCGGAAAGATTAGGCAGACGATGTCTTTTGAACACATTTTATCCCCAGCTGC
CAGCTGTCGCCCGGTTTATTGAACTTGGCCGTCAGTTACATCATCGGCGTGGCATTCGTCATCTC
GACGCCGCCTGTGGCGTGCAGGTTGGCTTTGCCACGCTTGACATTCTGGCAGCCTTGCTGGAG
GGTGTTGGTCCTTGGAGTCTTGAAAGCCCGTCCAATGATCTCAGCGCTATGCGTGAATTGTCCCT
TGTATTGGCCGAAGTGCCGCTGTCGCTGCACGTATTAAATGAACTGGCCGCAGCAGATGACGGT
CGCATGACGCTCCTCCAGCGTGTGTCCCTTACCACCGACCGGGGTACACTTAGCCTTCTTTCGC
CACATGGCCCCCTGTTGTGGACTCCGGCTGTCGCCGTGCCTGCGGAGGATGATGATGGCTTATT
TGCCCTGTTCGATGAAATCGCCGGAGAACCGTTGCCGTCCGCTCAATTGTGGTATGCCGAACCA
TGTAGTTGGGCTCAGGTGCATCAACGGCTGTGGCCAGCAGCAGCGGCCGAAGCACTGGCTCTG
CTGGCCGATGGAGATGAAGTGCGGCGACGCAATCAACGCTCTTTAGAAGTTGCCGCTCTGTGGC
AGCGCATCGGCGAGCGGCTTGGCTTCCCCGAAGCTCCCCCGGCATCACTGGCACCGGCTAGTT
TGGAGCAGGTTCTGGAGCAGGCGAGCTAA

>*Cp*IM1
ATGAAGGTCATTAGAGATAAAGATATTAAATCATTTCTTAACAAAAGGTTAACAAGGGAGTCCATT
TTTAGTCAATTCCAACCGGTATTGTTGCGTGGTTTAGCCACTTACGCAGCTAACCCAAATGCAAT
CGTTCCTCCAAGAATTGTCCAACAGTCGAATAATTCTGAGTCAGACACTACCCATGTGTTCATGC
CATGTATTTCACCTACCGAAGTGGGCATAAAAGTGATTAGTGGTGGTCCATCGAACAATACAAAG
GGGTTGGGGTTTCAAGGGTGTGTTATGATTTTGGATGAAGTAACTGGTGAATTGAATGCAATCTT
CAATGCCGCATGTTTAACTGCTTTCAGAACTGCATTAGCTAGTGTATTGGGTCTTACCAGAGTTGT
GCCTGTTGACTCTGTCGACGTTTTGCCGGAATTGTGTGTGTTTGGGGTAGGTCAACAAGCTTATT
GGCATGTCAAGTTGACTTTACTCTTGTATAAGGAGAAAATCGCGAAGGTGAACATACTAAATAGA
ACATTGGCAAATGCAGAAAAGTTGAAAGAGGAGTTGGGTAAAGAATTTGACAATGTTGAGTTCAG
AGCGTTCCTGTTTGAAGAGGATGAAAAGTTCAAACCGCATATGGAAAACAGTTCAATCATATACG
GATGCACTCCATCGACTTCAGCTGTGATAAAGAAAGACCATTTGAACAAAGATCCAAAATATCGA
AAGTTTATTTCTCTCATCGGTTCGTACAAGCCGCACATGATTGAGTTGGATTTGGAATTGATGAAT
GATTTCAAAAACAATGGCGTCAAAGTGATTGTTGACTCAAAGGAGCATACGTTACATGAAGCTGG
AGAATTGATACAATCAGGTTACACTAGTGACCAGTTGATTGAGATTCACGAATTGTATGAAACGG
AAGAGTTCAGTACGATTACAGATGCAACAACCGGTACAACCGTACAAAAGATTGTAGGATTATCA
ATCATGGACTTGTGCATGGGGAAGTACATTTATGAAAACATCCAAGACGATGATGCAGTTGTTGT
AAATGACTTTTAG

>*Pj*DHFR
AACCAGCAAAAGTCCCTCACCCTTATCGTGGCGCTTACGACGTCGTATGGCATCGGCCGTAGCA
ATAGTCTGCCATGGAAATTGAAGAAGGAAATCTCGTATTTTAAGCGCGTAACATCATTCGTCCCG
ACGTTTGATAGCTTTGAATCGATGAATGTCGTGCTCATGGGTCGTAAAACGTGGGAGTCAATTCC
GCTGCAGTTTCGTCCGCTGAAAGGTCGTATTAACGTTGTAATTACTCGTAATGAAAGCCTGGATT
TAGGTAACGGGATCCACTCGGCCAAAAGCCTGGATCATGCGCTGGAACTGCTGTACCGCACGTA
CGGTTCAGAGAGCAGCGTGCAAATCAACCGCATTTTTGTCATCGGCGGTGCCCAGTTATACAAA
GCCGCTATGGATCATCCGAAGTTGGATCGCATCATGGCCACAATTATTTACAAAGACATTCACTG
TGACGTCTTCTTCCCGTTGAAGTTTCGTGACAAAGAATGGAGCAGCGTCTGGAAAAAAGAAAAAC
ACTCGGATCTGGAGAGCTGGGTCGGTACTAAAGTGCCACATGGCAAAATTAACGAAGACGGTTT
CGATTATGAATTTGAGATGTGGACTCGTGACCTCTAA

>*Gg*DHFR
ATGGTGCGGTCCCTGAATAGTATCGTAGCAGTCTGTCAAAATATGGGTATTGGCAAAGACGGGA
ATCTGCCGTGGCCGCCGCTTCGTAACGAGTATAAATATTTTCAACGGATGACATCAACATCTCAC
GTGGAAGGAAAACAGAATGCCGTGATAATGGGCAAGAAAACCTGGTTTTCAATCCCGGAAAAAA
ATCGTCCCTTAAAAGATCGAATCAATATCGTGCTGTCCAGAGAATTGAAAGAAGCCCCGAAAGGT
GCACACTATCTGTCCAAAAGTCTGGACGACGCGTTAGCTTTATTGGACAGCCCAGAACTGAAATC
GAAAGTAGACATGGTGTGGATCGTAGGTGGCACCGCCGTTTACAAGGCGGCAATGGAGAAACC
GATAAACCATCGACTGTTTGTGACCAGAATCCTGCATGAATTTGAATCGGATACGTTCTTCCCAG
AAATAGATTATAAGGACTTCAAACTTCTGACCGAATACCCTGGAGTCCCTGCGGATATCCAGGAG
GAGAATGGTATTCAGTATAAATTTGAGGTCTATCAGAAATCTGTACTTGCACAATAA

>NAPW
ATGGAGAGAACACCCGATACGCCGGCTCCGGACTTACGCGGTAAGATTGCCCTGGTTGCTGGC
GCGACAAGAGGTGCAGGACGTGCGATAGCAGTTCAACTCGGCGCCGCGGGAGCTACAGTCTAC
GTAACTGGAAGAACTACCCGGGAACGTCGTTCAGAATACAATCGTAGTGAAACAATAGAGGAAA
CCGCAGAGTTGGTTACAGAGGCTGGTGGCACAGGTATTGCAGTCCCGACCGACCATCTGGTTCC
GGAGCAAGTGCGGGCCCTGGCTGATCGCGTGGACACTGAGCAAGGACGTCTCGATGTCTTAGT
AAACGATGTTTGGGGTGGTGAACGTCTGTTCGAATTCGACAAGAAAGTGTGGGAACATGATCTG
GACGCAGGGCTGCGTCTGATGCGTCTGGGAGTCGACACTCATGCAATTTCAAGCCACTTCTTGC
TGCCGCTCTTGGTGCGGCGTCCTGGTGGCTTAGTGGTTGAAATGACCGACGGAACGGCGGCAT
ACAATGGCAGTCATTATAGAAACTCCTATTTCTACGATCTTGTTAAGAACTCGGTATTACGTATGG
GCTACGTTCTGGCCCATGAGTTGGAGCCGTATGGCGGTACCGCTGTAACCTTAACTCCTGGATG
GATGCGTTCGGAGATGATGCTCGAAACATTAGGCGTTACTGAGGAGAACTGGAGAGACGCTTTG
ACGGAAGTCCCACATTTCTGTATTTCCGAGAGCCCATCGTACGTAGGACGTGCTGTAGCTGCTCT
CGCTGGTGACGCTGATGTTGCCAGATGGAATGGCCAATCCGTCAGCTCGGGTCAGCTCGCGCA
AGAATATGGCTTTACGGACCTGGACGGTAGCCGTCCCGATTGTTGGCGCTACCTTGTCGAGGTC
CAAGAGGCAGGTAAGCCTGCCGATCCGTCCGGTTACCGGTAA

>*Pb*SDR
ATGAAGCCTCTGCGTGGTAAAGTGGCTTTGGTGGCTGGCGCTACACGTGGTGCTGGTCGGGGC
ATAGCCGTTGAGCTTGGTGCCGCAGGTGCCACCGTGTATGTTACGGGCCGCACAACACGGCAG
CAGCGTAGTGAATATAACCGCCCAGAGACGATTGAAGAAACTGCAGAACTCGTTACCGCGGCAG
GCGGTCAGGGCATAGCCGTTCAGGTTGACCATTTACAGCCCGAACAGGTACAGGCTCTCATTGC
CCGAATTGAGAAGGAACAAGGCAGACTGGACGTTCTGGTTAATGACGTTTGGGGGGCGGAAAAT
CTTGCAGATTGGAATGTCCCTGTGTGGGATCATTCCCTTGAACGTGGATTCCGTATGCTTCGCTT
AGGCATCGATACGCACTTAATAACCTCCCATTTCGCGTTGCCCTTGCTTATTCGTAACAAGAATG
GCTTAGTAGTTGAGATGACAGATGGTACAGCGGAGTACAACTATAAAAACTACCGTATAAGCATG
TTTTATGATCTGGTCAAAAACTCCGTAATTCGCATGGCTCAAAGCCTGGCACATGAACTGGCGCC
ATATCAGTGTACTGCCGTGGCTATGACACCCGGTTGGATGCGGTCAGAGATTATGCTGGATCATT
TCGGAGTCAAAGAAGAAAATTGGCGCGACGCAGCGGAAAAGGAACCGCACTTTATCATTTCGGA
GTCCCCCCGTTACGTCGGTCGAGCGGTAGCAGCATTAGCTGGTGACCCCGAAGCTGCTAGATG
GAATGGAAAATCTCTGTCAAGTGGGCAGTTGGCTAAAGTCTATGGATTCACAGATCTTGATGGTT
CACAACCAGATTGCTGGCGCTACCTGGTCGAGGTACAGGAAGCTGGGAAGCCTGCAGATGCCA
GCGGCTATCGATAA

>*Mb*SDR
ATGAATGAAGATAGACGTCCTCTGGAAGGTAAGATCGCGTTGGTTGCCGGTGCTACCCGCGGTG
CCGGTCGTGGAATAGCAATCGAACTCGGTGCGGCGGGTGCAACGGTCTATTGTAGTGGGCGTT
CAAGCCGCGCAGATGTAGCGGGCCGTCGTGCGCCCGATGCACGTCCGTTTGAATTGTCTGGCC
GTCCCGAAACGATCGAGGAAACCGCAGAGCTGGTCACCGCAGCAGGGGGCACGGGTATTGCAA
TGCGCACCGATCATTTGGATGAAGATGCAGTAGCAGCGCTCGTTAAAAGAATCCGTGATGAACAT
GGGAGACTGGACGTCCTGGTGAACGATGTTTGGGGGGGAGATGCCCTGACTGAATGGGGAAAA
CCCTTTTGGGAGTTAGATCTGGAGCAGGGTCGAGTATTGCTTGATCGTGCCATCCGGACCCATG
TGGTTACAAGCCGTCATGCAGTACCCCTGCTGCTTGAACGACGCAGTCTTGAACGCCGGCTGAT
TGTTGAAATTACTGATGGGGATGCTATGTACTATCGCGGAAATTTTTTTTATGATATTGCGAAGAC
AACAGTCATTCGTCTGGCATTCGCAATGAGCGAGGAGTTACGCGAGCATGGAGTGGCGGCGGT
TGCCGTCACCCCGGGCTTCCTGCGCAGTGAAGCGATGCTGGAGCACTTTGGAGTAACTACAGA
GACTTGGCGTGACGGAGCGAAAAAAGACCCGCACTTTTTGTTCTCGGAAACACCGAGATTCGTG
GGTCGAGGAATTGCTGCACTGGCTTCTGATCCCGAGATCATGCGCCGCAGTGGAGGTCTCTTCT
CAAGTTGGCAACTGGCGGCTGAGTACGGGATAGATGACATTGACGGTACGAGACCCGATTGGG
GGAGCCATGCCGCCGGGTCTAGTTTTGCGGAAGAACATCGTGCTTCTCATGAGCGTTTTGTGCA
TGGAACGACCGCGCGGCATGCTGTTCGTGCTCCGGATGCCTCCGGCTCAGTTTAA

>*h*CRYM
ATGTCCAGAGTGCCGGCCTTCTTGAGCGCAGCTGAGGTAGAAGAACATTTGCGTTCCAGCTCAC
TTTTAATACCACCATTAGAGACGGCGTTGGCCAATTTCTCTTCTGGTCCTGAAGGTGGTGTGATG
CAGCCTGTCCGGACAGTGGTGCCCGTTACTAAGCACAGAGGATACTTAGGCGTCATGCCGGCAT
ATAGCGCGGCAGAGGATGCACTTACAACTAAATTGGTTACCTTTTACGAGGACCGTGGAATCAC
GTCTGTTGTCCCCTCTCATCAGGCGACTGTACTGCTGTTCGAACCCAGTAACGGCACTCTGCTTG
CCGTCATGGACGGCAACGTAATTACAGCAAAGCGGACAGCTGCTGTGTCGGCTATTGCGACCAA
GTTTCTCAAGCCACCTAGCTCTGAGGTACTGTGCATTCTTGGCGCCGGTGTGCAAGCGTACAGT
CACTATGAAATATTCACTGAACAGTTCAGCTTCAAGGAAGTCCGTATATGGAACCGAACCAAGGA
AAATGCTGAGAAATTTGCCGACACTGTGCAAGGTGAGGTGCGTGTGTGCAGTTCTGTCCAAGAG
GCCGTAGCCGGAGCAGACGTCATAATAACAGTGACTTTAGCAACCGAGCCGATACTGTTCGGTG
AGTGGGTCAAACCCGGCGCGCACATCAACGCCGTCGGGGCGTCCCGTCCTGATTGGCGTGAGT
TAGACGACGAGCTGATGAAGGAAGCAGTCCTGTATGTCGACAGCCAGGAAGCAGCCTTGAAGG
AGTCAGGTGATGTTCTGCTCTCAGGTGCCGAAATCTTTGCCGAGCTTGGTGAGGTTATAAAAGGA
GTTAAGCCCGCTCACTGCGAAAAGACAACTGTATTCAAAAGTCTGGGAATGGCGGTAGAGGACA
CGGTAGCAGCTAAATTAATATACGACAGCTGGAGCTCCGGGAAGTAA

>*Bt*CRYM
ATGTCATCTCGTCCTGTGTTTCTTAGCGCGGCAGACGTACAAGATCACCTGCGTAGCTCTTCATT
GCTGATCGCGCCCCTTGAGACTGCCTTGGCAAACTTTAGTTCTGGGCCTGATGGGGGTGTGGTG
CAGCCCGTTCGCACTGTTGTTCCTGTCGCAAAGCATCGTGGTTTTTTGGGCGTGATGCCGGCAT
ACTCGGCGGCAGAGGATGCACTGACTACCAAACTTGTCACTTTTTATGAAGACCACAGCGCAACT
TCTACGGTCCCCTCCCATCAGGCTACGGTACTGCTGTTTCAGCCGAGCAATGGGTCCCTGCTTG
CAGTGATGGATGGTAATGTTATAACTGCTAAACGTACCGCAGCCGTATCAGCCATCGCGACTAAA
TTTCTTAAACCTCCGAATTCCGAAGTACTGTGTATCCTGGGCGCTGGGGTCCAAGCTTATTCCCA

TTATGAAGTTTTTACTGAGCAATTTTTTTTTAAAGAAGTACGTATCTGGAACCGTACAAAAGAGAAT
GCAGAGAAATTTGTGAATACAGTTCCTGGAGAGGTTCGTATATGTTCGTCTGTTCAAGAAGCAGT
AACGGGTGCCGATGTAATAATCACCGTCACTATGGCGACCGAGCCAATATTGTTTGGAGAATGG
GTGAAACCGGGCGCTCACATCAACGCAATTGGCGCGTCGCGTCCAGATTGGCGAGAGCTGGAC
GACGAACTGATGAAACAGGCAGTGTTATATGTGGATAGCCAGGAAGCGGCTCTGAAAGAATCAG
GCGACGTACTGCTTTCAGGCGCCGAAATTTTTGCAGAGCTTGGAGAGGTTGTAAAGGGTGTAAA
GCCCGCGCACTGTGAAAAGACAACCGTTTTTAAAAGCCTGGGCATGGCCGTGGAAGATATGGTG
GCTGCCAAACTGGTCTATGATAGTTGGTCATCTGGGAAATAA

>*Tb*DHFR
ATGGTCTTTGACGCGCATTCTGCGTAAAAAAATCCCTGTACATGAACTGGCAGGTAAAATTTCTC
GCCCTCCCTTACGGCCTTTCAGCGTCGTAGTTGCCAGTGATGAGAAGGGGGGTATCGGTGATG
GAGGAACGATTCCATGGGAAATACCGGAAGATATGCAGTATTTCAGACGCGTAACCACGAACCT
GCGAGGGAAAAACGTGAAACCTTCTCCTAGCAAGCGTAACGCCGTAGTTATGGGTCGGAAAACT
TGGGATAGCTTGCCACCAAAATTTCGTCCCTTATCTAACCGGCTGAATGTGGTTCTGAGCCGGAG
TGCGACCAAAGAACAGCTGCTGGCTGGGATTCCCGACCCAATAAAACGTGCCGAAGCAGCCAAT
GATGTTGTCGCGGTTAATGGCGGTTTAGAAGACGCCCTGCGTATGTTGGTTTCTAAAGAACATAC
GTCTTCCATAGAAACAGTTTTCTGTATCGGTGGTGGGACGATTTATAAACAGGCCCTTTGTGCGC
CTTGCGTGAATGTGCTGCAGGCTATTCATCGTACCGTCGTCAGACCAGCTAGCAATTCTTGTTCC
GTGTTCTTTGATATTCCGGCGGCGGGGACGAAAACACCTGAAGGCCTGGAACTCGTTCGCGAGT
CGATCACAGATGAGCGGGTCAGCACGGGCGCGGGAGGTAAAAAGTATCAGTTCGAGAAGTTAG
TCCCGCGTAACAGTTAA

>*Tb*DHFR-TS
ATGCTTTCACTTACTCGGATTCTGCGCAAAAAAATTCCAGTGCATGAACTTGCGGGCAAAATATCT
CGGCCGCCGCTTCGCCCATTCTCGGTGGTAGTGGCCAGCGACGAGAAAGGGGGCATTGGCGAT
GGCGGTACTATCCCGTGGGAAATTCCGGAAGATATGCAATATTTTCGCCGCGTAACCACGAATCT
CAGAGGCAAAAATGTTAAGCCTAGTCCAAGCAAGCGGAATGCAGTAGTAATGGGCAGAAAAACG
TGGGATAGCCTTCCGCCAAAGTTCCGTCCCCTGTCAAACCGGCTGAACGTTGTACTTAGCCGCT
CTGCCACCAAGGAACAATTACTGGCCGGTATACCGGATCCTATCAAAAGAGCCGAAGCCGCTAA
TGATGTTGTGGCGGTTAATGGTGGGTTAGAAGATGCCTTGCGCATGCTTGTTAGTAAAGAACATA
CATCATCTATTGAAACAGTGTTTTGCATAGGAGGAGGGACAATATATAAGCAGGCCCTTTGTGCG
CCGTGCGTTAATGTGTTACAGGCTATCCATCGCACAGTCGTACGTCCCGCCTCTAATAGCTGCTC
TGTCTTCTTTGATATCCCTGCGGCAGGGACCAAAACTCCAGAAGGTTTAGAACTGGTTCGCGAAA
GTATTACAGACGAACGGGTTTCAACAGGCGCGGGTGGGAAAAAGTATCAATTTGAGAAACTGGT
GCCTCGTAATTCTGAAGAGGAACAGTATCTGAATCTGGTGGGCCGAATTATAGATGAAGGGTGC
ACCAAATGTGATCGCACTGGTGTTGGGACCCGTAGCCTTTTTGGTGCTCAGATGCGCTTTAGCCT
TCGCAATAATCGCCTTCCCCTGCTTACGACCAAACGCGTGTTTTGGCGTGGTGTTTGTGAAGAAC
TGCTCTGGTTCTTACGTGGTGAAACTAACGCGAAACTGCTTAGCGATAAAGGTATTCACATATGG
GATGGGAATGGCTCTCGTGCGTTTCTTGATAGCCGAGGTTTAACGGATTACGACGAAATGGATTT
AGGTCCTGTCTATGGTTTTCAATGGCGTCACTTTGGTGCCGATTATATTAGCTGCAAAGTGGATA
GTGAGGGCAAAGGCGTTGATCAAATCGCAAATATCGTTAAGTCTCTGATTGAAAACCCGGATGAT
CGTCGGATGATTTGCACAGCATGGAATCCCGCCGCACTTCCGAGAATGGCTCTCCCACCATGTC
ATATGATGGCTCAGTTTTATGTCAGCAATGGCGAACTGAGTTGTATGTTGTACCAGCGCTCTTGC
GACATGGGCCTGGGTGTTCCATTCAATATAGCTTCGTATGCACTGTTGACTTTCCTGATGGCCAA
AGCCAGTGGTCTTCGCCCTGGGGAACTGGTGCATACTCTGGGGGATGCTCACGTATATAGCAAT
CATGTAGAACCATGCAGAAAACAACTTAAGCGCGTTCCGCGTCCATTTCCGTTCATCGTGTTTAA
GCAGGATAAAGAATTTCTGGAAGACTTCCAGGAATCTGATATTGAAGTAATCGATTATTCTCCATA
TCCTGTGATTTCTATGGAAATGGCAGTTTAA

>hDHFR
ATGGTGGGCTCACTTAACTGTATCGTCGCTGTGTCTCAAAACATGGGTATTGGCAAGAACGGGG
ACTTACCATGGCCACCGCTGCGTAACGAGTTCCGATATTTTCAACGTATGACGACGACGAGCTCT
GTTGAGGGCAAACAGAACCTCGTTATCATGGGCAAGAAGACCTGGTTCAGCATTCCGGAAAAGA
ACCGCCCTCTTAAGGGTCGCATCAACTTAGTACTCTCACGCGAGCTCAAGGAACCGCCACAGGG
TGCGCACTTCTTGTCCAGATCCTTGGACGATGCACTCAAGCTTACCGAGCAACCCGAGTTAGCTA
ACAAGGTGGACATGGTGTGGATCGTAGGTGGGAGTTCTGTTTACAAGGAAGCTATGAACCACCC
AGGACACCTGAAGCTGTTTGTGACCCGGATTATGCAAGACTTCGAATCAGATACATTCTTTCCGG
AAATAGATCTGGAGAAGTACAAGTTGCTTCCTGAATACCCCGGCGTGTTATCAGATGTGCAAGAG
GAGAAGGGGATCAAGTACAAATTTGAAGTATATGAGAAGAACGACTAA

>*At*Prua
ATGAAATGCCGGAGTGCGGTCAAGGAGCATATCCCCAAAACTGTGTTAATTACTGGTGCCGCTC
GGCGCTTGGGACGCGCAATCGCTTCAGATCTGGCCGCGCATGGTTTTGCGATTGCCGTGCATG
CAAATGAATCAATGGCTCAGGCAGAAGAATTTGCGAACGAAATTCGCCAGAAAGGGGGTCGTGC
CACGGCCGTCCAGGCCGATCTGACTCAATCCGCACCGACAATGGCATTAGTGGAAAAAGCTGCG
GCCGCCTTGGGACCTATCGGAGTTGTAGTGAATAATGCCTCGGTCTTCTTGGCAGACACAGCTG
AAACCCCCGATCCCGCTGTCTTCGATGCCCATTTTGCAGTTCATGTCCGGGCACCTAGTCTGATT
GCTGCCGCATTCGTAGAACAGTTGCCAGCTGAAAAGTCAGGGCTGATAGTGAACATTATTGATCA
GCGTGTGTTGGCGCTGACCCCACGCTTTTATAGTTATACCTTGTCAAAATCAACGTTGTGGACAG
CAACGCGCACCATGGCCCAGAGTTTCGCACCGCGGGTGCGGGTCAATGCCATCGGACCGGGC
CCGACCTTCAAATCCGAACGCCAGGCCCCTCAAGATTTTCAGGCACAAATTGATGGGTTAATTCT
GAAGAGAGGTCCTGCCCCGGACGAATTTGGGCGCACTATTCGATTTCTGTATGATACGCCAAGT
ATTACTGGCCAGATGATCGCCCTGGATGGGGGCCAACACCTGGGTTGGGAGACCCCTGACGTG
GCTGAAATCCCAGAGTAA

>*Pa*Prua
ATGGCGACAGCTCCGATTTTGATTACAGGGGCGAGCCAGCGGGTGGGTCTGCACTGCGCTCGC
CGGCTCCTCGCCGATGGCGAAAGCGTTATCGTATCTTATCGGAGTGAACGTCCGGCTCTGGATG
AGCTTCGTCAGGCAGGGGCCCTCACTCTGCATGCAGATTTCGCGTCTGAAGCGGGTATATTTGC
TTTTATTGGAGCACTTCGCCAGCATACGGATAGTTTACGTGCCATTGTACACAATGCATCCGACT
GGGTTGCAGAAACCCCAGGTCATGAAGCTGAAGCCTTCCAACAGCTTTTTTCTGTTCATATGTTG
GCCCCTTATCTGATTAATCTTCATTGTGCCGAACTGCTTGAGCGCTCGCAACCAGCAGATATAGT
ACATCTGACTGATGACGTTGCCCGCAAAGGAAGTGCGCGCCGGATTGCTTATTGTGCATCGAAA
GCGGGCCTGGATAATCTTACCTTATCATTCGCAGCTAGATTCGCGCCTCGTATTAAGGTGAACGC
AATTAGTCCGGCCCTGGTCATGTTTAACGACGGGGATGATGCCGAATATCGCGCCCGCACGCTC
GCCAAATCAGCACTGGGAATTGAACCAGGGCCGGAAGTGATTTATCAATCTCTCAGATACCTTCT
TGATAACCCATACGTTACGGGCACGACCCTGACAGTGAATGGCGGCCGGCACGTTAA

Supplemental References

1. Zallot, R., Oberg, N., and Gerlt, J.A. (2019). The EFI Web Resource for Genomic Enzymology Tools: Leveraging Protein, Genome, and Metagenome Databases to Discover Novel Enzymes and Metabolic Pathways. Biochemistry *58*, 4169–4182. https://doi.org/10.1021/acs.biochem.9b00735.

2. Shannon, P., Markiel, A., Ozier, O., Baliga, N.S., Wang, J.T., Ramage, D., Amin, N., Schwikowski, B., and Ideker, T. (2003). Cytoscape: A Software Environment for Integrated Models of Biomolecular Interaction Networks. *Genome Res.* ***13***, 2498–2504. https://doi.org/10.1101/gr.1239303.

3. Aleku, G.A., France, S.P., Man, H., Mangas-Sanchez, J., Montgomery, S.L., Sharma, M., Leipold, F., Hussain, S., Grogan, G., and Turner, N.J. (2017). A reductive aminase from *Aspergillus oryzae*. *Nat. Chem*. ***9***, 961–969. https://doi.org/10.1038/nchem.2782.
